# Plastid phylogenomics of the cool-season grass subfamily: clarification of relationships among early-diverging tribes

**DOI:** 10.1093/aobpla/plv046

**Published:** 2015-05-02

**Authors:** Jeffery M. Saarela, William P. Wysocki, Craig F. Barrett, Robert J. Soreng, Jerrold I. Davis, Lynn G. Clark, Scot A. Kelchner, J. Chris Pires, Patrick P. Edger, Dustin R. Mayfield, Melvin R. Duvall

**Affiliations:** 1 Botany Section, Research and Collections, Canadian Museum of Nature, PO Box 3443 Stn. D, Ottawa, ON, Canada K1P 3P4; 2 Biological Sciences, Northern Illinois University, 1425 W. Lincoln Hwy, DeKalb, IL 60115-2861, USA; 3 Department of Biological Sciences, California State University, 5151 State University Dr., Los Angeles, CA 90032-8201, USA; 4 Department of Botany, National Museum of Natural History, Smithsonian Institution, Washington, DC 20013-7012, USA; 5 Section of Plant Biology, Cornell University, 412 Mann Library, Ithaca, NY 14853, USA; 6 Ecology, Evolution and Organismal Biology, Iowa State University, 251 Bessey Hall, Ames, IA 50011-1020, USA; 7 Biological Sciences, Idaho State University, 921 S. 8th Ave, Pocatello, ID 83209, USA; 8 Division of Biological Sciences, University of Missouri, 1201 Rollins St, Columbia, MO 65211, USA; 9 Department of Plant and Microbial Biology, University of California – Berkeley, Berkeley, CA 94720, USA

**Keywords:** Chloroplast genome, core Pooideae, phylogenetics, phylogenomics, plastome, Poeae, *Schedonorus arundinaceus*

## Abstract

Whole plastid genomes are being sequenced rapidly from across the green plant tree of life, and phylogenetic analyses of these are increasing resolution and support for relationships that have varied among or been unresolved in earlier single- and multi-gene studies. Pooideae, the cool-season grass lineage, is the largest of the 12 grass subfamilies and includes important temperate cereals, turf grasses and forage species. Although numerous studies of the phylogeny of the subfamily have been undertaken, relationships among some ‘early-diverging’ tribes conflict among studies, and some relationships among subtribes of Poeae have not yet been resolved. To address these issues, we newly sequenced 25 whole plastomes, which showed rearrangements typical of Poaceae. These plastomes represent 9 tribes and 11 subtribes of Pooideae, and were analysed with 20 existing plastomes for the subfamily. Maximum likelihood (ML), maximum parsimony (MP) and Bayesian inference (BI) robustly resolve most deep relationships in the subfamily. Complete plastome data provide increased nodal support compared with protein-coding data alone at nodes that are not maximally supported. Following the divergence of *Brachyelytrum*, Phaenospermateae, Brylkinieae–Meliceae and Ampelodesmeae–Stipeae are the successive sister groups of the rest of the subfamily. Ampelodesmeae are nested within Stipeae in the plastome trees, consistent with its hybrid origin between a phaenospermatoid and a stipoid grass (the maternal parent). The core Pooideae are strongly supported and include Brachypodieae, a Bromeae–Triticeae clade and Poeae. Within Poeae, a novel sister group relationship between Phalaridinae and Torreyochloinae is found, and the relative branching order of this clade and Aveninae, with respect to an Agrostidinae–Brizinae clade, are discordant between MP and ML/BI trees. Maximum likelihood and Bayesian analyses strongly support Airinae and Holcinae as the successive sister groups of a Dactylidinae–Loliinae clade.

## Abstract (precis)

Whole plastid genomes (plastomes) are being sequenced rapidly from across the green plant tree of life, and phylogenetic analyses of these are increasing resolution and support for relationships that were unresolved in earlier studies. The cool-season grass subfamily, Pooideae, includes important temperate cereals, turf grasses and forage species, yet some aspects of deep phylogeny in the lineage are unresolved. We newly sequenced 25 Pooideae plastomes, and conducted phylogenomic analyses of these and 20 existing plastomes from the subfamily. Most aspects of deep relationship in Pooideae are maximally supported in our analyses, including those among early-diverging tribes.

## Introduction

Advances in next-generation sequencing technologies (Moore *et al.* 2006; Cronn *et al.* 2008; Parks *et al.* 2009; Wysocki *et al.* 2014) have resulted in a rapid increase in completed plastid genomes (Jansen and Ruhlman 2012) sampled widely across the green plant tree of life. The use of whole plastomes to infer phylogenies (i.e. phylogenomics) provides opportunities to potentially increase resolution and support for relationships that have varied among or been unresolved and/or poorly supported in earlier single- and multi-gene studies. Plastomes have been used to address diverse phylogenetic questions at deep (Ruhfel *et al.* 2014) to shallow (Parks *et al.* 2009) hierarchical levels, and to characterize plastid genome evolution (e.g. patterns of gene loss and organization, GC content, microstructural events, evolutionary rates) (e.g. Barrett and Davis 2012; Jansen and Ruhlman 2012).

Grasses are the fourth largest family of flowering plants in the world, with some 11 000 species and 600–900 genera. At the family level, numerous phylogenetic studies have been conducted and the deep phylogenetic framework for Poaceae is well established. The family contains three small, deeply diverging subfamilies (Anomochlooideae, Pharoideae, Puelioideae) that are the successive sister groups of a large clade comprising two major lineages, the Bambusoideae, Ehrhartoideae, Pooideae (BEP) and the Panicoideae, Arundinoideae, Chloridoideae, Micrairoideae, Aristidoideae, Danthonioideae (PACMAD) clades (Grass Phylogeny Working Group 2001; Duvall *et al.* 2007; Sanchez-Ken and Clark 2007; Bouchenak-Khelladi *et al.* 2008; Saarela and Graham 2010; Grass Phylogeny Working Group II 2012).

The BEP clade includes the bamboo (Bambusoideae), rice (Ehrhartoideae) and cool-season (Pooideae) grass subfamilies. The origin of its crown clade has been dated at ∼40–53 to 70.7–72.6 million years ago, depending on which fossil calibration points are used (Christin *et al.* 2014). Earlier analyses inferred varying relationships among the subfamilies of the BEP clade (Hsiao *et al.* 1998; Hilu *et al.* 1999; Zhang 2000; Grass Phylogeny Working Group 2001; Duvall *et al.* 2007) and a few studies found that Pooideae may be the sister group of the PACMAD clade (Soreng and Davis 1998; Duvall *et al.* 2007), but there is now consensus from plastid multi-gene analyses that Bambusoideae and Pooideae are sister taxa (Bouchenak-Khelladi *et al.* 2008; Saarela and Graham 2010; Grass Phylogeny Working Group II 2012; Wu and Ge 2012). This is consistent with some symplesiomorphic bambusoid macro- and micromorphological characters in ‘early-diverging’ lineages within Pooideae that were traditionally included in Bambusoideae (Clark *et al.* 1995). This topology has been confirmed by a phylogenomic study of 121 orthologous nuclear genes (Zhao *et al.* 2013) and a species tree inferred from >18 000 maximum likelihood (ML) gene trees (Burleigh *et al.* 2011), but it was not recovered in a nuclear analysis of 43 putative orthologous cDNA sequences (Peng *et al.* 2010).

Pooideae, the cool-season grass lineage, is the largest in the BEP clade and the largest of the 12 grass subfamilies, with some 189 genera, 10 nothogenera and 3900 species (Grass Phylogeny Working Group 2001; Clayton *et al.* 2006 onwards; Soreng *et al.* 2014). It includes important temperate cereals like wheat (*Triticum*), barley (*Hordeum*) and oats (*Avena*), cool-season turf grasses in the genera *Festuca*, *Poa*, *Agrostis*, *Lolium* and *Schedonorus* (Beard 2012), and numerous temperate forage species. Since the establishment of Pooideae in the mid-19th century by Bentham (1861), its circumscription has undergone radical realignment as understanding of the evolutionary history of grasses advanced (reviewed in Soreng and Davis 2000; Soreng *et al.* 2007). Pooideae, in its current circumscription, was first recognized as a natural group in cladistic studies of morphological data (Kellogg and Campbell 1987), and this hypothesis has been corroborated by numerous molecular phylogenetic studies (Soreng *et al.* 1990; Davis and Soreng 1993, 2007, 2010; Cummings *et al.* 1994; Nadot *et al.* 1994; Clark *et al.* 1995; Catalán *et al.* 1997; Hsiao *et al.* 1998; Soreng and Davis 1998; Hilu *et al.* 1999; Mathews *et al.* 2000; Grass Phylogeny Working Group 2001; Duvall *et al.* 2007; Bouchenak-Khelladi *et al.* 2008; Grass Phylogeny Working Group II 2012; Blaner *et al.* 2014 [*mat*K analyses]).

Multiple phylogenetic analyses based on plastid and nuclear ribosomal data have clarified the major evolutionary lineages within Pooideae (e.g. Davis and Soreng 2007; Döring *et al.* 2007; Soreng *et al.* 2007; Bouchenak-Khelladi *et al.* 2008; Schneider *et al.* 2009). These are recognized as supertribes, tribes and/or subtribes in two recent classifications that differ only in the ranks chosen for some lineages. The classification by Soreng *et al.* (2014), modified from Soreng *et al.* (2003, 2007), recognizes 2 supertribes, 14 tribes and 1 subtribe, while that of Röser and colleagues (Döring *et al.* 2007; Schneider *et al.* 2009, 2011) recognizes 9 tribes, 9 subtribes and 1 tribal complex (Table 1). Here we follow the classification of Soreng *et al.* (2014) **[see Supporting Information]**.

**Table 1.** Comparison of the Pooideae classifications of Soreng *et al*. (2014) and Schneider *et al*. (2009, 2011). Parallel taxa (taxon names in boldface) are equivalent in circumscription but may differ in rank. Numbers in square brackets are the number of genera in each taxon according to Soreng *et al*. (2014). Schneider *et al*. (2009, 2011) do not provide a subtribal classification for their Aveneae/Poeae complex.

Soreng *et al*. (2014)	Schneider *et al.* (2009, 2011)
Tribe **Brachyelytreae** Ohwi [1]	Tribe **Brachyelytreae**
	Tribe **Nardeae**
Tribe **Nardeae** W.D.J. Koch. [1]	Subtribe **Nardinae** Kromb.
Tribe **Lygeae** J. Presl [1]	Subtribe **Lygeinae** Röser
Tribe **Phaenospermateae** Renvoize & Clayton [3]	Tribe **Phaenospermateae**
Subtribe **Duthieinae** Pilg. ex Potztal [5–6]	Tribe **Duthieae** Röser & J. Schneider
	Tribe **Stipeae**
Tribe **Stipeae** Dumort. [∼29]	Subtribe **Stipinae** Griseb.
Tribe **Ampelodesmeae** Tutin [1]	Subtribe **Ampelosdesminae** Conert
	Tribe **Meliceae**
Tribe **Meliceae** Link ex Endl. [6]	Subtribe **Melicinae** Fr.
Tribe **Brylkinieae** Tateoka [2]	Subtribe **Brylkiniinae** Ohwi
Tribe **Diarrheneae** C.S. Campb. [2]	Tribe **Diarrheneae**
Tribe **Brachypodieae** Harz [1]	Tribe **Brachypodieae**
Supertribe **Triticodae** T.D. Macfarl. & L. Watson	Tribe **Hordeae** Kunth ex Spenn.
***Littledalea*** Hemsl. [1]	Subtribe **Littledaleinae** Röser
Tribe **Bromeae** Dumort. [1]	Subtribe **Brominae** Dumort.
Tribe **Triticeae** Dumort. [∼25] [this name, published in 1824, has priority over Hordeae Kunth ex Spenn., published in 1825]	Subtribe **Hordeinae** Dumort.
Supertribe **Poodae** L. Liu	
Tribe **Poeae** R.Br. [∼120]	**Aveneae/Poeae** complex [unranked]
Poeae chloroplast group 1 (Aveneae type) [= Poeae clade 1 in current study]	
Subtribe **Torreyochloinae** Soreng	
Subtribe **Aveninae** J. Presl	
Subtribe **Phalaridinae** Fr.	
Subtribe **Anthoxanthinae** A. Gray	
Subtribe **Brizinae** Tzvelev s.s.	
Subtribe **Brizinae** s.l. “Calotheca Clade”	
Subtribe **Agrostidinae** Fr.	
Poeae chloroplast group 2 (Poeae type) [= Poeae clade 2 in current study]	
Subtribe **Scolochloinae** Tzvelev	
Subtribe **Sesleriinae** Parl.	
Subtribe **Coleanthinae** Rouy	
Subtribe **Miliinae** Dumort.	
Subtribe **Poinae** Dumort.	
Subtribe **Holcinae** Dumort.	
Subtribe **Airinae** Fr.	
Subtribe **Loliinae** Dumort.	
Subtribe **Dactylidinae** Stapf	
Subtribe **Cynosurinae** Fr.	
Subtribe **Ammochloinae** Tzvelev	
Subtribe **Parapholiinae** Caro	

Pooideae comprise numerous, mostly [with the exception of Meliceae (150 species) and Stipeae (557); Clayton *et al.* 2006 onwards] species-poor ‘early-diverging’ lineages (those resulting from the earliest or deepest splits in the clade), and the core Pooideae, which as defined by Soreng *et al.* (2007), includes the tribes Brachypodieae, Bromeae, Poeae, Triticeae and the genus *Littledalea*, and includes the majority of species in the subfamily. Most studies identify *Brachyelytrum* [= Brachyelytreae], a small enigmatic genus that has been variously recognized as pooid or bambusoid (reviewed in Saarela *et al.* 2003), as the sister group of the rest of the subfamily [Catalán *et al.* 1997; Grass Phylogeny Working Group 2001; Davis and Soreng 2007 (*matK* analyses); Duvall *et al.* 2007; Bouchenak-Khelladi *et al.* 2008; Davis and Soreng 2010; Grass Phylogeny Working Group II 2012; Blaner *et al.* 2014], although in a few studies *Brachyelytrum* falls outside the Pooideae clade [Davis and Soreng 1993; Soreng and Davis 1998; Blaner *et al.* 2014 (topoisomerase 6 analysis)]. After the divergence of *Brachyelytrum*, a clade of *Lygeum* [= Lygeeae] and *Nardus* [= Nardeae] is the next successive sister group of the rest of the subfamily in multiple studies [Catalán *et al.* 1997; Soreng and Davis 1998, 2000; Mathews *et al.* 2000; Grass Phylogeny Working Group 2001; Davis and Soreng 2007, 2010; Döring *et al.* 2007 (Nardeae not sampled); Duvall *et al.* 2007; Bouchenak-Khelladi *et al.* 2008; Schneider *et al.* 2011; Grass Phylogeny Working Group II 2012; Blaner *et al.* 2014], with the exception of two in which *Brachyelytrum* and *Nardus* form a moderately supported clade that is sister to rest of the subfamily (Hsiao *et al.* 1998; Hilu and Alice 1999).

The tribes Meliceae, Brylkinieae, Phaenospermateae, Stipeae and Ampelodesmeae are the next emerging branches in the phylogeny. Brylkinieae has been included only in a few analyses, where it has been placed as the sister group of Meliceae (Schneider *et al.* 2009, 2011; Davis and Soreng 2010; Romaschenko *et al.* 2010, 2012; Rodionov *et al.* 2013; Blaner *et al.* 2014). The monotypic *Ampelodesmos* (*A. mauritanicus*) [= Ampelodesmeae] has consistently been nested in Stipeae in plastid and nuclear ribosomal analyses (Hsiao *et al.* 1998; Davis and Soreng 2007, 2010; Grass Phylogeny Working Group II 2012; Romaschenko *et al.* 2012), although it differs dramatically in spikelet morphology, being multi-flowered rather than single-flowered like Stipeae. Using a low copy nuclear gene, Romaschenko *et al.* (2014) recently demonstrated that *Ampelodesmos* originated via hybridization between a phaenospermatoid and an ‘early-diverging’ stipoid grass (the maternal parent), explaining its placement in Stipeae in plastid trees, and leading Soreng *et al.* (2014) to accept Ampelodesmeae as a monotypic tribe.

The interrelationships among Stipeae–Ampelodesmeae, Meliceae and Phaenospermateae with respect to the rest of Pooideae have varied substantially among studies based on different taxon samplings, molecular markers and methods of phylogenetic inference. Three studies infer these to be successively diverging lineages with strong support in Bayesian analyses for at least two of the three relevant deep nodes, but in each of these studies the position of at least one of the tribes/lineages differs with respect to the others. The best supported topology is presented in a tree based on two plastid genes, in which Phaenospermateae (not monophyletic in the analysis), Stipeae–Ampelodesmeae and Meliceae are strongly supported as successively diverging lineages (Duvall *et al.* 2007). In contrast, a tree based on three plastid genes identifies Stipeae–Ampelodesmeae, Phaenospermateae and Meliceae as successively diverging lineages (in other words, the branching order of the first two lineages is reversed), but with weak support for the clade that includes Phaenospermateae, Meliceae and the rest of Pooideae (Grass Phylogeny Working Group II 2012). A nine-region plastid tree supports Phaenospermateae, Brylkinieae–Meliceae and Stipeae–Ampelodesmeae as successively diverging lineages, but with weak support for the clade including Stipeae–Ampelodesmeae and the rest of Pooideae (Romaschenko *et al.* 2012).

Diarrheneae and Brachypodieae are resolved as the next successively diverging lineages in most analyses, sister to a clade including Bromeae, *Littledalea*, Poeae and Triticeae [Clark *et al.* 1995 (Brachypodieae not sampled); Catalán *et al.* 1997; Hilu *et al.* 1999 (Diarrheneae not sampled); Grass Phylogeny Working Group 2001; Davis and Soreng 2007; Döring *et al.* 2007; Duvall *et al.* 2007 (Bayesian analysis); Schneider *et al.* 2009 (Diarrheneae not sampled); Davis and Soreng 2010; Schneider *et al.* 2011 (ITS, but weak support for the Diarrheneae + Brachypodieae + rest of Pooideae clade); Grass Phylogeny Working Group II 2012 (Diarrheneae not sampled)]. In other studies, different topologies were found: the two tribes were resolved as a clade [Bouchenak-Khelladi *et al.* 2008; Blaner *et al.* 2014 (matK analysis)], Brachypodieae were inferred to have diverged prior to Diarrheneae (Mathews *et al.* 2000), the two lineages comprised a polytomy with the rest of the core Pooideae (Schneider *et al.* 2011) and Diarrheneae were part of an unresolved polytomy with Stipeae, Meliceae, Phaenospermateae, Ampelodesmeae and the core Pooideae [Blaner *et al.* 2014 (based on sequences of the nuclear gene *topoisomerase* 6)]. In one ITS analysis, Diarrheneae were weakly supported as the sister group of the rest of Pooideae (Hsiao *et al.* 1998).

Among the core Pooideae, Triticeae and *Brachypodium* are estimated to have diverged 32–39 Mya, based on comparisons of their nuclear genomes (The International *Brachypodium* Initiative 2010); this represents the origin of the crown core Pooideae. An independent estimate places the origin of the Triticeae–Poeae split at ∼26–33.5 Mya (Sandve and Fjellheim 2010). There is consensus that Triticeae and Bromeae are sister taxa (Catalán *et al.* 1997; Hsiao *et al.* 1998; Soreng and Davis 1998, 2000; Hilu *et al.* 1999; Mathews *et al.* 2000; Grass Phylogeny Working Group 2001; Davis and Soreng 2007, 2010; Döring *et al.* 2007; Duvall *et al.* 2007; Bouchenak-Khelladi *et al.* 2008; Schneider *et al.* 2009, 2011; Grass Phylogeny Working Group II 2012; Blaner *et al.* 2014) and all studies that have sampled *Littledalea* have found this genus to be the sister group of the Bromeae–Triticeae clade (Davis and Soreng 2007, 2010; Döring *et al.* 2007; Soreng *et al.* 2007; Schneider *et al.* 2009, 2011; Blaner *et al.* 2014), with the exception of one in which cloned *topoisomerase 6* sequences of *Littledalea tibetica* formed a polytomy with the Bromeae–Triticeae clade (Blaner *et al.* 2014).

Poeae is the most species-rich of the tribes of Pooideae, with some 2258 species distributed in cool-temperate, Mediterranean and arctic climates (Clayton *et al.* 2006 onwards). Taxa now included in Poeae have been variously arranged in multiple smaller tribes and subtribes (reviewed by Soreng *et al.* 2007; see also Quintanar *et al.* 2007; Gillespie *et al.* 2008). Of these, the Aveneae (the oat tribe) and the Poeae s.s. have been recognized most widely, distinguished on the basis of several morphological characters (e.g. Clayton and Renvoize 1986), some of which are quite homoplasious (Soreng *et al.* 2007). Multiple studies have demonstrated that Aveneae and Poeae s.s. are not monophyletic in any of their traditional circumscriptions (e.g. Soreng and Davis 2000; Davis and Soreng 2007; Döring *et al.* 2007; Quintanar *et al.* 2007; Soreng *et al.* 2007; Schneider *et al.* 2009). Within Poeae s.l. two major clades have been identified in analyses of plastid deoxyribonucleic acid (DNA), which have been variously recognized informally (Soreng and Davis 2000; Saarela *et al.* 2010; Grass Phylogeny Working Group II 2012; Soreng *et al.* 2014); we refer to these simply as Poeae clades 1 and 2, as in Schneider *et al.* (2009). Six subtribes are recognized in Poeae clade 1 and 12 subtribes in Poeae clade 2 (Table 1). Evolutionary relationships within and among these lineages have been addressed in numerous studies across the tribe (Soreng *et al.* 2007; Schneider *et al.* 2009, 2011, 2012) and within and among tribes: Airinae and Holcinae (Chiapella 2007), Aveninae (Grebenstein *et al.* 1998; Röser *et al.* 2001; Rodionov *et al.* 2005; Winterfeld *et al.* 2009*a*, *b*, 2014; Romero-Zarco 2011), Brizinae (Essi *et al.* 2008), Loliinae (Torrecilla and Catalán 2002; Catalán *et al.* 2004; Torrecilla *et al.* 2004; Inda *et al.* 2008), Phalaridinae (Voshell *et al.* 2011), Poinae (Gillespie *et al.* 2007, 2008, 2009, 2010; Hoffmann *et al.* 2013), Poeae clade 1 (Quintanar *et al.* 2007, 2010; Saarela *et al.* 2010). However, numerous aspects of the relationships within and among the subtribes of Poeae remain unclear.

### Grass plastomes

The economically important grasses were among the first organisms to have their plastid genomes sequenced. The plastome of rice (Hiratsuka *et al.* 1989) was the third species, after tobacco and a liverwort, and the first monocot completed; and the maize plastome (Maier *et al.* 1995) was the sixth one completed (Jansen *et al.* 2005). There are now (as of 25 September 2014) some 106 plastid genomes publicly available for grasses, representing 43 genera,78 species and 8 subfamilies: Anomochlooideae (1 species) (Givnish *et al.* 2010; Morris and Duvall 2010; Jones *et al.* 2014), Pharoideae (2 species) (Jones *et al.* 2014), Puelioideae (1 species) (Jones *et al.* 2014), Bambusoideae (33 species) (Wu *et al.* 2009; Zhang *et al.* 2011; Burke *et al.* 2012, 2014; Wu and Ge 2012; Gao and Gao 2014; Ma *et al.* 2014), Ehrhartoideae (7 species) (Hiratsuka *et al.* 1989; Shahid Masood *et al.* 2004; Tang *et al.* 2004; Wu and Ge 2012; Lin *et al.* 2014), Panicoideae (7 species) (Maier *et al.* 1995; Asano *et al.* 2004; Calsa Júnior *et al.* 2004; Saski *et al.* 2007; Diekmann *et al.* 2009; Leseberg and Duvall 2009; Young *et al.* 2011; Besnard *et al.* 2013), Pooideae (48 species) (Ogihara *et al.* 2000; Saski *et al.* 2007; Diekmann *et al.* 2009; Hand *et al.* 2013; Gornicki *et al.* 2014; Middleton *et al.* 2014) and Chloridoideae (1 species) (Wysocki *et al.* 2014) **[see Supporting Information]**.

Several studies have generated whole plastomes for grasses, and phylogenetic analyses of these plastomes have resulted in increased resolution and support for relationships within and among some grass subfamilies compared with trees in earlier single- and multi-gene studies. For example, Jones *et al.* (2014) found the two-genus subfamily Anomochlooideae to be monophyletic, a result found in some (Clark *et al.* 1995; Grass Phylogeny Working Group 2001; Duvall *et al.* 2007; Grass Phylogeny Working Group II 2012) but not all [Hilu *et al.* 1999; Zhang 2000; Bouchenak-Khelladi *et al.* 2008; Blaner *et al.* 2014 (*matK* analyses)] previous analyses, and they reconstructed species-level relationships in Pharoideae. Several plastome studies support a sister group relationship between Bambusoideae and Pooideae (Zhang *et al.* 2011; Wu and Ge 2012; Burke *et al.* 2014; Jones *et al.* 2014—but see the ML tree in Young *et al.* 2011) and in Bambusoideae plastomes have substantially improved resolution and support within and among species of the temperate woody bamboo tribe Arundinarieae (Zhang *et al.* 2011; Ma *et al.* 2014) compared with earlier multi-locus plastid phylogenies (Triplett and Clark 2010; Zeng *et al.* 2010). In Triticeae, plastomes have clarified relationships among closely related species of wheat (*Triticum*) and goatgrass (*Aegilops*) (Gornicki *et al.* 2014; Middleton *et al.* 2014). These examples indicate that whole plastomes hold much promise for resolving relationships among grass clades that have previously been problematic. Here, we report 25 new plastomes of taxa of Pooideae and use these in combination with previously published plastomes to infer phylogenetic relationships among the major lineages in the subfamily.

## Methods

### Taxon sampling and DNA extraction

Silica-dried leaf tissue was obtained from 25 species of pooid grasses (Table 2). Tissue was homogenized manually in liquid nitrogen before extraction. The DNA extraction protocol was followed using the Qiagen DNeasy Plant Mini Kit (Qiagen Inc., Valencia, CA, USA).

**Table 2.** Voucher specimen information and GenBank accession numbers for newly sequenced plastomes, place of publication of previously published plastomes and Illumina library preparation methods (TruSeq, Nextera or TruSeq Nano) used to produce reads of the newly sequenced taxa. The tribal/subtribal classification follows Soreng *et al*. (2014).

Tribe/subtribe	Species	Voucher specimens and GenBank accession numbers [ ] for newly sequenced plastomes, or place of plastome publication for previously sequenced plastomes	Illumina library preparation method
Brachyelytreae	*Brachyelytrum aristosum* (Michx.) P. Beauv. ex Branner & Coville	USA New York: *J.I. Davis 777* (BH) [KM974735]	TruSeq Nano
Phaenospermateae	*Phaenosperma globosum* Munro ex Benth.	USA: *J.I. Davis 779* (BH) [KM974745]	TruSeq Nano
Ampelodesmeae	*Ampelodesmos mauritanicus* (Poir.) T. Durand & Schinz	Germany. *Royl & Schiers s.n.* (B) [KM974731]	Nextera
Stipeae	*Achnatherum hymenoides* (Roem. & Schult.) Barkworth	Canada. British Columbia: *J.M. Saarela, C.J. Sears & J.R. Maze 725* (CAN-590407) [KM974729]	TruSeq
	*Oryzopsis asperifolia* Michx.	Canada. British Columbia: *J.M. Saarela & D.M. Percy 430* (CAN-590301) [KM974744]	TruSeq
	*Piptochaetium avenaceum* (L.) Parodi	USA Maryland: *R.J. Soreng & K. Romaschenko* (US) [KM974748]	TruSeq Nano
Meliceae	*Melica mutica* Walter	USA Maryland: *W.J. Kress & M. Butts 04-7461* (US) [KM974742]	TruSeq Nano
	*Melica subulata* (Griseb.) Scribn.	Canada. British Columbia: *J.M. Saarela, D.M. Percy & Y.M. Chang 836* (CAN 590495) [KM974743]	Nextera
Diarrheneae	*Diarrhena obovata* (Gleason) Brandenburg	USA: *J.I. Davis 756* (BH) [KM974739]	TruSeq Nano
Brachypodieae	*Brachypodium distachyon* (L.) P. Beauv.	Bortiri *et al.* (2008)	
Bromeae	*Bromus vulgaris* (Hook.) Shear	Canada. British Columbia: *J.M. Saarela, D.M. Percy & Y.M. Chang* 822 (CAN-590469) [KM974737]	TruSeq
Triticeae	*Aegilops cylindrica* Host	Middleton *et al.* (2014)	
	*Aegilops geniculata* Roth	Middleton *et al.* (2014)	
	*Aegilops speltoides* Tausch	Middleton *et al.* (2014)	
	*Aegilops tauschii* Coss.	Middleton *et al.* (2014)	
	*Hordeum jubatum* L.	Canada. Yukon: *P.M. Peterson, J.M. Saarela & S.F. Smith 18478* (CAN-591095) [KM974741]	TruSeq
	*Hordeum vulgare* L. subsp. *vulgare*	Saski *et al.* (2007)	
	*Hordeum vulgare* subsp. *spontaneum* (K. Koch) Asch. & Graebn.	Middleton *et al.* (2014)	
	*Secale cereale* L.	Middleton *et al.* (2014)	
	*Triticum aestivum* L.	Bahieldin *et al.* (2014)	
	*Triticum boeoticum* Boiss.	Middleton *et al.* (2014)	
	*Triticum monococcum* L.	Middleton *et al.* (2014)	
	*Triticum urartu* Thumanjan ex Gandilyan	Middleton *et al.* (2014)	
Poeae			
Agrostidinae	*Agrostis stolonifera L.*	Saski *et al.* (2007)	
	*Ammophila breviligulata* Fernald	USA New York: *P.M. Peterson & J.M. Saarela 20867* (CAN) [KM974730]	Nextera
Airinae	*Helictochloa hookeri* (Scribn.) Romero Zarco	Canada. Saskatchewan: *P.M. Peterson, J.M. Saarela & S.F. Smith 18359* (CAN-590913) [KM974734]	Nextera
Anthoxanthinae	*Anthoxanthum odoratum* L.	Canada. British Columbia: *J.M. Saarela 500* (CAN-591412) [KM974732]	TruSeq
	*Hierochloe odorata* (L.) P. Beauv.	USA Massachusetts: *E.A Kellogg s.n.* (A) [KM974740]	Nextera
Aveninae	*Avena sativa* L.	Canada. British Columbia: *J.M. Saarela & D.M. Percy 775* (CAN-590451) [KM974733]	Nextera
	*Trisetum cernuum* Trin. subsp. *cernuum*	Canada: British Columbia: *J.M. Saarela, D.M. Percy & Y.M. Chang 876* (CAN-0591575) [KM974753]	Nextera
Brizinae s.s.	*Briza maxima* L.	Canada. Alberta [cultivated]: *J.M. Saarela 284* (CAN) [KM974736]	Nextera
Coleanthinae	*Puccinellia nuttalliana* (Schult.) A.S. Hitchc.	Canada. British Columbia: *J.M. Saarela, C.J. Sears & J.R. Maze 713* (CAN 591508) [KM974750]	TruSeq
Dactylidinae	*Dactylis glomerata* L.	Canada. British Columbia. *J.M. Saarela 496* (CAN 591411) [KM974738]	TruSeq
Holcinae	*Deschampsia antarctica* E. Desv.	Lee *et al.* (2014)	
Loliinae	*Festuca altissima* All.	Hand *et al.* (2013)	
	*Festuca ovina* L.	Hand *et al.* (2013)	
	*Lolium multiflorum* Lam.	Hand *et al.* (2013)	
	*Lolium perenne L.*	Diekmann *et al.* (2009)	
	*Schedonorus arundinaceus* (Schreb.) Dumort.	Canada. British Columbia: *J.M. Saarela 331* (CAN-591322) [KM974751]	TruSeq
	*Schedonorus arundinaceus* (Schreb.) Dumort. (as *Lolium arundinaceum* (Schreb.) Darbysh.)	Cahoon *et al.* (2010)	
	*Schedonorus pratensis* (Huds.) P. Beauv. (as *Festuca pratensis* Huds.)	Hand *et al.* (2013)	
Phalaridinae	*Phalaris arundinacea* L.	Canada. British Columbia: *J.M. Saarela, D.M. Percy & Y.M. Chang 973* (CAN-590547) [KM974746]	TruSeq
Poinae	*Phleum alpinum* L.	Canada. British Columbia: *J.M. Saarela & D.M. Percy 1234* (CAN-590642) [KM974747]	TruSeq
	*Poa palustris* L.	Canada. British Columbia: *J.M. Saarela & D.M. Percy 1080* (CAN-591589) [KM974749]	TruSeq
Torreyochloinae	*Torreyochloa pallida* var. *pauciflora* (J. Presl) J.I. Davis	Canada. British Columbia: *J.M. Saarela & D.M. Percy 1110* (CAN-591622) [KM974752]	TruSeq

### Plastome sequencing

Two micrograms of total genomic DNA from *Brachyelytrum aristosum*, *Phaenosperma globosum*, *Piptochaetium avenaceum*, *Melica mutica* and *Diarrhena obovata* were used in each library preparation. Libraries were prepared using the TruSeq Nano DNA sample preparation kit (Illumina, San Diego, CA, USA) and sequenced paired-end at Cold Spring Harbor Laboratory, Cold Spring, NY, USA. Libraries were prepared for the remaining taxa using the TruSeq and Nextera library preparation kits (Table 2). Detailed protocols for TruSeq, Nextera and TruSeq Nano are provided in Wysocki *et al.* (2014), Burke *et al.* (2014) and Barrett *et al.* (2013, 2014), respectively.

All Illumina-sequenced reads were first quality filtered using DynamicTrim v2.1 from the SolexaQA software package (Cox *et al.* 2010) with default settings, and then sequences <25 bp in length (default setting) were removed with LengthSort v2.1 in the same package.

### Plastome assembly, annotation and alignment

Plastome assembly was performed entirely *de novo*. The Velvet software package (Zerbino and Birney 2008) was run iteratively following the methods from Wysocki *et al.* (2014). Contigs were scaffolded using the anchored conserved region extension method (Wysocki *et al.* 2014). Any remaining gaps in the plastomes were repaired by locating overlapping regions of 20 bp or higher using contigs or raw reads until the circular map was complete. Fully assembled plastomes were annotated by aligning to a previously published and annotated reference plastome in Geneious Pro (Biomatters Ltd, Auckland, New Zealand) and copying the reference annotations to the assembled plastome when the annotation shared a minimum similarity of 70 %. The banked plastome from *Lolium perenne* (NC009950) was used as an annotation reference.

Plastomes were arranged with the large single-copy (LSC) region followed by the inverted repeat region B (IRb) and ending with the short single-copy (SSC) region. Inverted repeat region A (IRa) was omitted from the matrix to prevent overrepresentation of the inverted repeat sequence. All newly assembled plastomes were then aligned, along with 20 previously published pooid plastomes and one bamboo outgroup plastome, *Bambusa bambos* (KJ870988) (Table 2), using the MAFFT alignment software (Katoh *et al.* 2005). We used the corrected sequence of *Triticum aestivum* (Bahieldin *et al.* 2014), as the earlier plastome (Ogihara *et al.* 2000) contained sequences from the rice plastome. The alignment was then inspected for structural mutations and adjusted manually to preserve tandem repeat boundaries and to identify inversions. Regions that contained inversion mutations were deleted from the matrix to remove false homology inferences. The alignment file may be obtained from TreeBase (http://purl.org/phylo/treebase/phylows/study/TB2:S16741).

### Indels

Indel mutations were scored in the plastome alignment as in Leseberg and Duvall (2009). We scored indels that (i) were ≥2 bp long, excluding mono-nucleotide repeats regardless of length; (ii) could be attributed to slipped-strand mispairing, identified by the presence of a perfect or near-perfect repeated sequence; (iii) were unambiguous and did not overlap with other indels in the alignment; and (iv) were present in two or more individuals (i.e. autapomorphic indels were not scored). These criteria allowed us to focus on indels that could be interpreted as single evolutionary events. Indels were not included in our analyses. Based on the topology of the ML tree, scored indels were interpreted as representing putative synapomorphies or putative homoplasy.

### Phylogeny estimation

We conducted maximum parsimony (MP), ML and Bayesian inference (BI) analyses with two different taxon sets and two subsets of the data. The first taxon set included 45 of the 46 taxa in our matrix, excluding a previously published plastome of *Schedonorus arundinaceus* that has been reported as having some sequencing errors (Hand *et al.* 2013), and the second included this plastome (46 taxa). For each of these matrices, we conducted analyses of complete plastomes (non-coding and protein coding) and protein-coding sequence (cds). The procedures, analyses and parameters noted below were used for all analyses. To exclude the potential alignment ambiguity from the analyses, nucleotide positions that contained one or more gaps introduced by the alignment were omitted from the matrix. The Akaike Information Criterion (AIC) was used in jModelTest v 2.1.3 (Guindon and Gascuel 2003; Darriba *et al.* 2012) to compare models of character evolution in each of the gap-free matrices. The General Time Reversible (GTR) model of substitution incorporating invariant sites and a γ distribution (GTR + I + G) was among the best-fit models and was used in subsequent analyses. Maximum likelihood analysis was performed using RAxML v 8.0.5 (Stamatakis 2006) with 1000 non-parametric bootstrap replicates. Non-parametric bootstrap values were generated using the Consense function in Phylip (Felsenstein 2005). MrBayes 3.2.2 (Ronquist and Huelsenbeck 2003) was used to perform the BI analyses. A Dirichlet prior was used for base frequencies and the rate matrix, and a uniform prior was used for the shape parameter (α), proportion of invariable sites (I) and topology; these are the default prior settings. Branch lengths were unconstrained and a GTR + I + G model was used with four discrete rate categories. The Metropolis-coupled Markov chain Monte Carlo (MCMCMC) search/sampling analysis was run for 2 × 10 000 000 generations with four chains. Average standard deviation of split frequencies remained <0.001 after the 50 % burn-in. Heuristic parsimony [1000 random addition sequence (RAS) replicates; TBR branch swapping; best trees kept] and parsimony bootstrap analysis with 1000 pseudoreplicates, 10 RAS each, were performed with PAUP* v4.0b10 (Swofford 2003). All results are presented on ML phylograms.

### Testing outgroup selection effects

The set of 45 pooid plastomes was analysed with a randomized set of outgroup taxa to test the effect of outgroup selection on the tree topology. Outgroup taxa were selected from a pool of previously published plastome sequences from Bambusoideae [*Acidosasa purpurea* (NC015820), *Arundinaria gigantea* (NC020341), *A. fargesii* (NC024712), *Bambusa bambos* (KJ870988), *Dendrocalamus latiflorus* (NC013088), *Fargesia nitida* (NC024715), *Ferrocalamus rimosivaginus* (NC015831), *Indocalamus longiauritus* (NC015803), *Phyllostachys nigra* (NC015826), *Olyra latifolia* (KF515509)], Ehrhartoideae [*Leersia tisserantii* (NC016677), *Oryza sativa* (NC001320), *Rhynchoryza subulata* (NC016718)] and the PACMAD clade [*Coix lacryma-jobi* (NC013273), *Neyraudia reynaudiana* (NC024262), *Panicum virgatum* (NC015990), *Sorghum bicolor* (NC008602), *Saccharum* hybrid (NC006084) and *Zea mays* (NC001666)]. A representative species from each of the three lineages was chosen randomly using a custom Python script (available on request), and aligned with the pooid plastomes with the MAFFT method. Phylogeny was estimated in an ML framework using RAxML. This was repeated 16 times and the tree with the highest likelihood from each iteration was tested for congruence using the Consense function of the Phylip software package.

## Results

### Plastome sequencing

Complete plastomes were newly sequenced for 25 pooid grass species. Plastome lengths ranged from 134 287 to 137 897 bp. All lengths of plastomes and their sub-regions are reported in Table 3. After the removal of one inverted repeat region, the 46-taxon alignment included 135 838 nucleotide positions, which decreased to 94 022 positions after the removal of all positions with at least one introduced gap (30.78 % positions removed). The 45-taxon alignment included 135 059 nucleotide positions, which decreased to 94 209 positions after the removal of all positions with at least one introduced gap (30.25 % positions removed). Removing all gapped positions reduced the 46-taxon protein-coding region alignment from 47 001 to 45 213 positions (3.80 % removed) and reduced the 45-taxon alignment from 46 708 to 45 256 positions (3.11 % removed).

**Table 3.** Lengths (bp) of newly sequenced plastomes and their sub-regions. LSC, large single-copy; IR, inverted repeat; SSC, short single-copy.

Taxon	Total	LSC	IR	SSC
*Achnatherum hymenoides*	137 742	81 709	21 615	12 803
*Ammophila breviligulata*	136 725	80 710	21 657	12 701
*Ampelodesmos mauritanicus*	136 975	79 543	22 273	12 886
*Anthoxanthum odoratum*	135 551	79 626	21 627	12 671
*Avena sativa*	135 890	80 109	21 603	12 575
*Brachyelytrum aristosum*	137 399	81 819	21 434	12 712
*Briza* maxima	136 823	79 707	22 197	12 722
*Bromus vulgaris*	136 935	80 964	21 702	12 567
*Dactylis glomerata*	134 737	79 523	21 472	12 270
*Diarrhena obovata*	137 421	81 367	21 621	12 812
*Hierochloe odorata*	136 394	80 645	21 642	12 465
*Helictochloa hookeri*	134 976	79 370	21 502	12 602
*Hordeum jubatum*	136 834	80 901	21 629	12 675
*Melica mutica*	134 710	80 478	20 831	12 570
*Melica subulata*	134 773	80 411	20 836	12 690
*Oryzopsis asperifolia*	134 287	80 475	20 503	12 806
*Phaenosperma globosum*	137 897	82 128	21 446	12 877
*Phalaris arundinacea*	135 873	79 833	21 530	12 980
*Phleum alpinum*	135 568	80 009	21 368	12 823
*Piptochaetium avenaceum*	137 701	81 613	21 625	12 838
*Poa palustris*	135 446	79 566	21 552	12 776
*Puccinellia nuttalliana*	135 352	79 594	21 516	12 726
*Schedonorus arundinaceus*	135 266	79 934	21 421	12 490
*Torreyochloa pallida*	136 102	80 137	21 641	12 683
*Trisetum cernuum*	135 539	79 828	21 636	12 439

### Testing outgroup selection effects

Sixteen iterations of randomly sampling the outgroup taxa produced 16 identical ingroup topologies. However, the maximum likelihood bootstrap support (MLBS) did vary at five nodes. The sister group to *Melica* showed a mean MLBS of 99.75 (±0.58), the sister group to *Diarrhena obovata* showed a mean MLBS of 82.19 (±5.94), the sister group to the *Phalaris–Torreyochloa* clade showed a mean bootstrap support (BS) of 93.38 (±2.16), the sister group to *Helictochloa hookeri* showed a mean BS of 74.38 (±13.09) and the sister group to *Deschampsia antarctica* showed a mean BS of 80.63 (±12.83).

### Phylogeny

**Figure 1.**
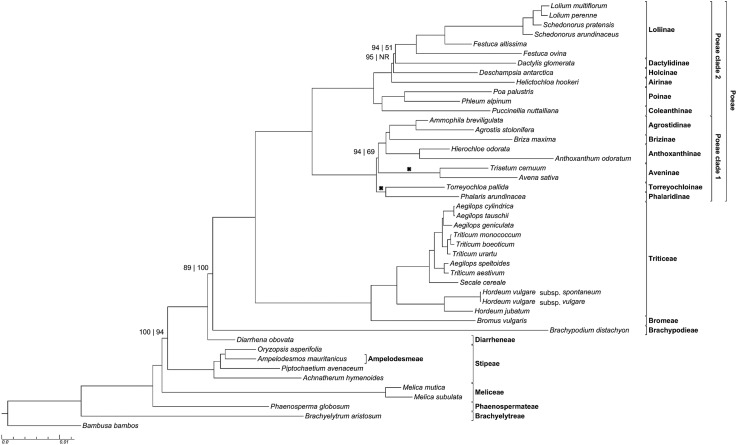
Maximum likelihood phylogram of complete plastomes of 44 pooid grasses and one outgroup taxon. Tribes of Pooideae, subtribes of Poeae and Poeae clades 1 and 2 are indicated. Bootstrap values are indicated only when at least one is less than maximally supported (ML bootstrap value precedes MP bootstrap value). ML and Bayesian (BI) topologies are identical. Posterior probabilities at all nodes = 1.00. ‘NR’ indicates a node not resolved or supported above the 50 % bootstrap level in the MP analysis. Crosses indicate clades that are in reversed positions in the MP tree. The MP bootstrap value of the node affected by the reversal of these clades reflects the value associated with the reversed topology, i.e. the topology not reflected in this figure.

**Figure 2.**
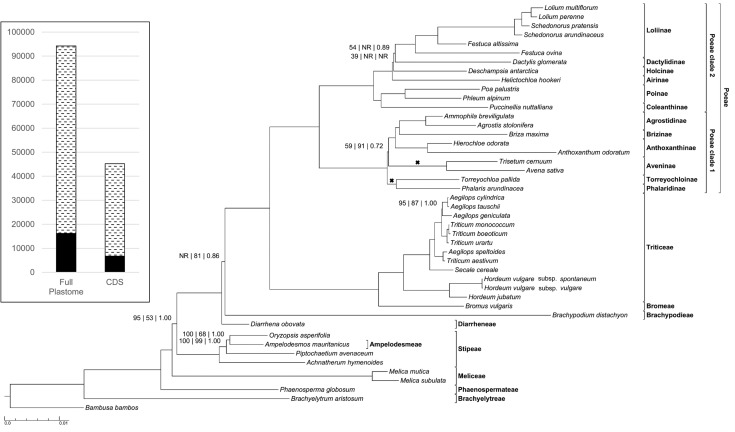
Maximum likelihood phylogram of plastome protein cds of 44 pooid grasses and 1 outgroup taxon. Tribes of Pooideae, subtribes of Poeae and Poeae clades 1 and 2 are indicated. Support values are indicated only when at least one is less than maximally supported (ML bootstrap value precedes MP bootstrap value, which precedes the Bayesian posterior probability). ‘NR’ indicates nodes not resolved or supported above the 50 % bootstrap level in one of the analyses. Crosses indicate clades that were reversed in the MP tree. The MP bootstrap value of the node affected by the reversal of clades reflects that of the reversed topology. The histogram indicates total invariant (stippled) and variable (solid) nucleotide sites in the full plastome analysis (Fig. 1) and this analysis.

The 45-taxon MP analyses of the complete and protein cds resulted in one and two equally most parsimonious trees, respectively. In the 45-taxon analysis of the complete data, all but four nodes in the ML tree and three nodes in the MP tree are maximally supported (BS = 100 %) and all nodes in the BI tree have posterior probabilities of 1 (Fig. 1). In the 45-taxon analysis of protein cds, all but four nodes in the ML tree and three nodes in the BI tree were maximally supported (Fig. 2). In the MP tree, five nodes received support between 50 and 99 %, and no topology among *Helictochloa*, *Dactylis*, *Deschampsia* and Loliinae received >50 % BS (Fig. 2). Support is lower in the protein cds analyses compared with the complete analyses at nodes that are not maximally supported in both. The topologies of the ML and BI trees are identical in the 45-taxon analyses of both data subsets. The parsimony trees differ in the relative placements of four subtribes (two clades) in one major clade of tribe Poeae, and two to three subtribes in a second major clade of the tribe. Bootstrap support in parsimony trees is generally lower than BS in ML trees at nodes that are not maximally supported in both.

In the 46-taxon analysis of the complete data, all but three nodes in the ML tree and four nodes in the MP tree are maximally supported, and all nodes in the BI tree are maximally supported **[see Supporting Information]**. In the 46-taxon analysis of protein cds, all but six nodes in the ML tree, seven nodes in the MP tree and four nodes in the BI tree are maximally supported. Support for most nodes is lower in the trees based on protein cds tree compared with the complete plastome data. The remainder of the text focusses on the 45-taxon dataset, unless indicated otherwise.

Ampelodesmeae is nested in Stipeae, and henceforth we refer to this lineage as the Ampelodesmeae–Stipeae clade. Within this clade, *Achnatherum* and *Piptochaetium* are successive sisters to an *Oryzopsis*–*Ampelodesmos* clade.

In all analyses, Brachyelytreae are inferred to be the sister group of the rest of Pooideae. Phaenospermateae, Meliceae, Ampelodesmeae–Stipeae and Diarrheneae are the next successively diverging lineages, sister to the core Pooideae. The core Pooideae including Brachypodieae, Bromeae, Poeae and Triticeae are a moderately to strongly supported clade in all analyses of the complete data [MLBS = 89 %, maximum parsimony bootstrap support (MPBS) = 100 %, PP = 1.00] (Fig. 1). Support is lower based on the protein cds (MLBS <50 %, MPBS = 81 %, PP = 0.86); in the ML tree, *Diarrhena* and *Brachypodium* are a weakly supported clade (MLBS = 56 %, data not shown) (Fig. 2).

Within the core Pooideae, Bromeae and Triticeae are sister taxa, and this clade is sister to Poeae. Within Triticeae, *Secale* is the sister group of an *Aegilops*–*Triticum* clade in which neither *Aegilops* nor *Triticum* is monophyletic, and *Hordeum* is the sister group to this larger lineage. Poeae is divided into two clades: Poeae clade 1, including Agrostidinae, Brizinae s.s., Anthoxanthinae, Aveninae, Phalaridinae and Torreyochloinae; and Poeae clade 2, including Coleanthinae, Poinae, Airinae, Holcinae, Dactylidinae and Loliinae. In Poeae clade 1, one subclade has the following topology: [Anthoxanthinae, (Brizinae, Agrostidinae)] and Phalaridinae and Torreyochloinae form a clade. In the ML and BI trees, Phalaridinae–Torreyochloinae and Aveninae are strongly (MLBS = 94 %, PP = 1.00; Fig. 1) to weakly (MLBS = 59 %, PP = 0.72; Fig. 2) supported as the successive sister groups of the rest of the clade, whereas in the MP tree the branching order of these two lineages is reversed and weakly (MPBS = 69 %; Fig. 1) to strongly (MPBS = 91 %; Fig. 2) supported. In Poeae clade 2, Coleanthinae and Poinae are sister taxa, and a second subclade comprises Airinae, Holcinae, Dactylidinae and Loliinae. In ML and BI complete data trees Airinae and Holcinae are strongly supported successive sister groups of the rest of the subclade, whereas in the MP tree Airinae and Holcinae are an unsupported clade (Fig. 1); and relationships at the base of this clade are unresolved in the MP analysis of protein cds (Fig. 2). In ML and BI analyses, Dactylidinae and Loliinae are sister taxa; this relationship is strongly supported in analyses of the complete data but poorly supported in the protein-coding analyses.

### Unique plastome features

The plastome from *Brachyelytrum aristosum* shares a 196 bp insertion with *Bambusa bambos* in the *rps16-trnQ* intergenic spacer. The two insertions exhibit 85 % identity. This insertion is not present in any other genera of Pooideae but is represented in all subfamilies of grasses except for Danthonioideae and Micrairoideae. *Phaenosperma globosum* and both species of *Melica* share a 198 bp insertion in the same region that shows no significant sequence similarity to the insertion shared by *Bambusa bambos* and *Brachyelytrum aristosum*. The insertions found in *Melica* and *P. globosum* are not found in any other species of Pooideae included here, but a BLAST query reveals similar insertions in one species of Puelioideae (*Puelia olyriformis*), an ‘early-diverging’ member of the Panicoideae (*Thysanolaena latifolia*) and many members of the temperate bamboo lineage Arundinarieae.

**Figure 3.**
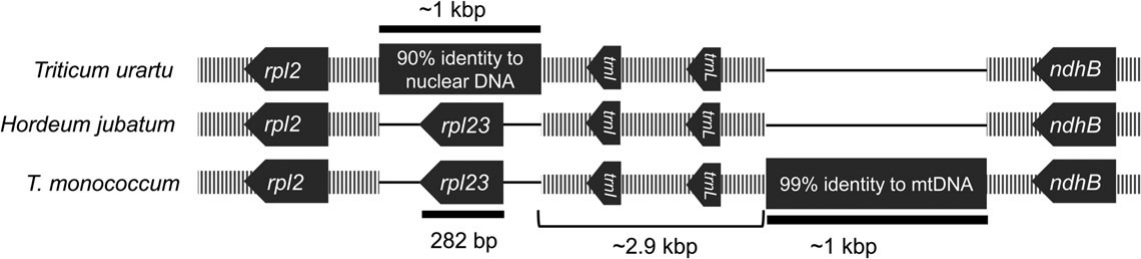
Two putative insertions of nuclear and mitochondrial homology located in IR regions of the *Triticum urartu* (NC_021762) and *T. monococcum* (NC_021762) plastomes, respectively, are shown here and compared with the typical *Hordeum jubatum* plastome (KM974741). The insertion in the *T. urartu* plastome exhibits 90 % identity to chromosome 3B of the *T. aestivum* nuclear genome (HG670306) and the insertion in the *T. monococcum* plastome exhibits 99 % identity to the *T. timopheevii* mitochondrial genome (AP013106). Gene orientation is indicated and thin lines denote regions in which gaps were introduced to preserve the alignment. Gene position is relative and lengths are not to scale.

Two undocumented putative insertions of non-plastid homology were found in two previously published plastomes (Fig. 3). The full plastome for *Triticum urartu* contains a 1060 bp insertion in the region of the inverted repeat that ordinarily contains the coding sequence for the *rpl23* gene. A BLAST query of this sequence against GenBank shows the region to have homology to various nuclear genes within multiple species of *Triticum. Triticum monococcum* contains a 1077 bp insertion in the *rpl23-ndhB* intergenic spacer of the inverted repeat region. A BLAST query of this sequence against GenBank shows the region to have homology to the *Triticum timopheevii* mitochondrial genome and many other grass mitochondrial regions. The top hit was located in the *rrn26-1* region of the *T. timopheevii* mitochondrial genome, covered nearly 100 % of plastome insertion and exhibited over 99 % identity.

### Indels

A total of 177 indels were scored that we interpreted as single evolutionary events **[see Supporting Information]**. Eighty-four of these could be attributed to slipped-strand mispairing. All of the scored indels could be straightforwardly interpreted as being putatively synapomorphic or putatively homoplastic. Given the current level of taxon sampling, 34 indels were putatively homoplastic with respect to the topology of the ML tree, while the remaining 143 represented putative synapomorphies for clades of various composition, ranging from multi-tribal clades to those of a single species (in cases where more than one individual per species was sampled) (Table 4). One indel was putatively synapomorphic for the large clade comprising Diarrheneae plus the core Pooideae; 2 indels each were putatively synapomorphic for a three-tribe and a four-tribe lineage; 30 indels were putatively synapomorphic for 6 two-tribe lineages, with 20 of these supporting the Bromeae–Triticeae clade; 37 indels were putatively synapomorphic for 8 tribes, with 13 of these supporting Meliceae and 7 supporting Triticeae and 9 indels were putatively synapomorphic for three species. Within Triticeae, 7 indels represented putative synapomorphies for clades of subsets of *Aegilops* and *Triticum* species, 4 were putatively synapomorphic for an *Aegilops*–*Triticum* clade, 10 were putatively synapomorphic for an *Aegilops*–*Triticum*–*Secale* clade and 6 were putatively synapomorphic for *Hordeum*. Within Loliinae 4 indels were putatively synapomorphies for a clade of all sampled taxa except *Festuca ovina*, and 14 were putative synapomorphies for a *Lolium*–*Schedonorus* clade. Seven and ten indels were putative synapomorphies for Poeae clades 1 and 2, respectively. A total of 111 indels are located in intergenic spacer regions, 33 in introns and 14 in protein-coding regions (genes).

**Table 4.** Unambiguous indels scored in the 46-taxon plastome alignment that are putatively synapomorphic for clades of two or more taxa, or single taxa represented by more than one individual. Putatively homoplasious indels are also scored. **[See Supporting Information]** for details of scored indels.

Clade	Number of indels
*Aegilops cylindrica*–*A. tauschii*	1
*Aegilops speltoides*–*Triticum aestivum*	2
*Aegilops*–*Triticum*	4
*Aegilops*–*Triticum* minus *A. speltoides* and *T. aestivum*	3
*Aegilops*–*Triticum*–*Secale*	10
Agrostidinae	3
Agrostidinae–Brizinae	1
Airinae–Dactylidinae–Loliinae	2
Ampelodesmeae–Stipeae	2
Anthoxanthinae	5
Aveninae	4
Bromeae–Poeae–Triticeae	2
Bromeae–Triticeae	20
Coleanthinae–Poeae	2
Diarrheneae–Brachypodieae–Bromeae–Poeae–Triticeae	1
Homoplasious indels	34
*Hordeum*	6
*Hordeum vulgare*	6
Loliinae	2
*Lolium*–*Schedonorus*	14
*Lolium*–*Schedonorus*–*Festuca altissima*	4
*Melica*	13
Phalaridinae–Torreyochloinae	1
Poeae	4
Poeae clade 1	7
Poeae clade 2	10
Poinae	2
*Schedonorus arundinaceus*	2
Stipeae	1
Triticeae	7
*Triticum* minus *T. aestivum*	1
*Triticum monococcum*	1

## Discussion

Phylogenomic analyses of 45 whole plastomes, including 25 newly sequenced here, resulted in a highly resolved and strongly supported phylogeny of Pooideae, with the caveat that whole plastomes for three tribes (Nardeae, Lygeae, Brylkineae) and *Littledalea* are not yet sampled. The few topological differences between the MP, ML and BI trees are in tribe Poeae and at the base of the core Pooideae. Each of the discordant inferred clades is subtended by a very short branch. As most branches in the Poeae clade define genera and are fairly long, the topological differences observed may be a reflection of long-branch attraction (LBA) in the MP analysis due to the proximity of very short and very long branches in the tree. This is a common problem in phylogenetic inference and has been observed in other whole plastome phylogenomic studies (Soltis and Soltis 2004; Stefanović *et al.* 2004; Givnish *et al.* 2010; Barrett *et al.* 2014). Strategies to overcome LBA include using inference methods that are less prone to long branch effects and, therefore, may be more accurate (such as ML or BI inference, which we have done here); excluding third codon positions, which may be saturated or randomized; representing clades in analyses with only short-branched taxa by excluding taxa with long branches; adding taxa to break up large branches and adding data (Bergsten 2005). An alternative strategy to adding more data could involve data filtration. For example, quality measures of ‘tree-likeness’ for data partitions could be used to identify the ‘data core.’ Aside from LBA, factors that might lead to differences in topology and/or support among data partitions include model mis-specification, intrinsic biases among the data (e.g. large differences in GC content among clades; Ruhfel *et al.* 2014) and heterotachy.

For most tribes and subtribes we have sampled only one or two exemplar taxa, so removing taxa from the analyses would be a poor approach. In contrast, with rapidly decreasing costs for sequencing whole plastomes, there is ample opportunity to increase taxon sampling within and among tribes, particularly those that are species-rich, such as Poeae—the clade in which the incongruences among analyses were found and in which there are numerous unresolved phylogenetic questions. Given our current strategy of sampling whole plastomes, adding more data from this linkage group is impossible, by definition. Adding data from the mitochondrial or nuclear genomes would be beneficial for deciphering the true evolutionary history of taxa (i.e. the species tree), as genes from these genomes may have evolutionary histories that are different than those of maternally inherited plastid genomes, such as the nuclear gene history found by Triplett *et al.* (2014), but this would not help with the long-branch problem in the plastid data. Coalescent-based analyses of multiple unlinked nuclear genes could help accurately reconstruct species relationships, especially for parts of the plastome phylogeny that may be at odds with the genealogies of some or many nuclear genes. Because probability-based methods of phylogenetic inference are less prone to long-branch effects than parsimony, our discussion below is based primarily on the topologies of our nearly identical ML and BI trees, which also are generally better supported than the MP trees.

Previous phylogenomic studies of relationships among orders and families of plants have examined only protein-coding genes (e.g. Barrett *et al.* 2013, 2014; Davis *et al.* 2013; Kim and Kim 2013; Martin *et al.* 2013; Ruhfel *et al.* 2014), while those—including the current study—that have included non-coding data have generally focussed on more recent or ‘shallow’ phylogenetic relationships (Zhang *et al.* 2011; Burke *et al.* 2012; Hand *et al.* 2013; Gornicki *et al.* 2014; Huang *et al.* 2014; Ma *et al.* 2014; Middleton *et al.* 2014). Even though spacers/introns are ‘non-coding’ some plastid intergenic regions are expected to be conserved due to containing enzyme-binding sites or important secondary structures (Peredo *et al.* 2012) and they may too be under purifying selection similar to coding loci, and thus useful in larger-scale phylogenetic reconstructions. Nevertheless, aligning non-coding regions (or a subset of them) across divergent taxa can be difficult and may introduce error into phylogenetic analyses when alignments are not accurate, and we have attempted to minimize the possibility of this type of error by excluding from analysis all positions in the alignment that include a gap in one or more taxon. The improvement in support in our analyses of complete plastomes versus protein cds indicates that the non-coding complement of the genome contributes important characters for resolving these mostly deep nodes in pooid grasses, similar to the results of other phylogenomic studies of grasses (Burke *et al.* 2012; Ma *et al.* 2014) and other plants (Eserman *et al.* 2014) in which analyses of complete plastomes and/or non-coding data and protein cds were explicitly compared. Inclusion of non-coding data in plastome phylogenomic analyses of such groups as Zingiberales (Barrett *et al.* 2014) may improve support levels at deep nodes that are weakly supported based on protein cds alone.

### ‘Early-diverging’ Pooideae

Among the tribes of Pooideae major uncertainties of relationship have revolved around the relative branching orders of Phaenospermateae, Brylkinieae–Meliceae and Ampelodesmeae–Stipeae following the respective divergences of *Brachyelytrum* and Lygeae–Nardeae. Plastomes are not yet available for *Lygeum* and *Nardus*. As expected, *Brachyelytrum* represents one of the lineages resulting from the deepest split in the subfamily, and we find strong support for Phaenospermateae, Brylkinieae–Meliceae and Ampelodesmeae–Stipeae to be the next successive sister groups to the rest of Pooideae. The branch subtending all Pooideae except *Brachyelytrum* is quite long, indicating a long period of time prior to the next divergence and/or a rapid rate of plastid evolution along this branch. Conversely, the next two deep branches at the base of the plastome trees identifying Phaenospermateae, Meliceae and Ampelodesmeae–Stipeae as successively diverging taxa are extremely short compared with most other deep branches in the tree subtending lineages recognized as tribes, possibly reflecting rapid radiation of these lineages. Given these short branches, it is not surprising that most previous studies were not able to robustly resolve the relationships among these taxa, particularly those based on one or a few gene regions (Catalán *et al.* 1997; Mathews *et al.* 2000; Döring *et al.* 2007; Schneider *et al.* 2009; Davis and Soreng 2010; Blaner *et al.* 2014). Our results are congruent with the largest study, in terms of gene regions sampled, that sampled these lineages (Romaschenko *et al.* 2012), although not all nodes in that study were strongly supported as they are in our plastome trees. In contrast, our results are discordant with two- and three-gene studies that recovered strong support in BI analyses for the opposite order of divergence for Ampelodesmeae–Stipeae and Meliceae (Duvall *et al.* 2007; Bouchenak-Khelladi *et al.* 2008). The reasons for this are not clear, but may be due to differing levels of taxon sampling in and near this part of the tree, outgroup choice (although tests of different grass outgroups here had no effect on the ingroup topology) or varying phylogenetic signal in different partitions of the data [as demonstrated for monocots in Davis *et al.* (2013), for example]. Considering the latter possibility, support was much lower for the current topology in the MP analyses of protein-coding data compared with the complete dataset, while support in the ML and BI analyses was strong in both. Diarrheneae represent the next successive sister group to the rest of the subfamily (the core Pooideae) in most of our plastome trees, congruent with the results of other studies [Clark *et al.* 1995; Catalán *et al.* 1997; Davis and Soreng 2007, 2010; Döring *et al.* 2007; Duvall *et al.* 2007 (Bayesian analysis)]. An alternative topology recovered only in the ML analysis of protein cds, in which *Brachypodium* and *Diarrhena* are a clade, was poorly supported. This topology was also found in Minaya *et al.* (2013), with strong support.

### Core Pooideae

#### Brachypodieae

Accurate placement of *Brachypodium* in the context of the phylogeny of Pooideae is important, as *B. distachyon* has become a model species for the genomics of cereal crops, grass biofuels and forage grasses (The International *Brachypodium* Initiative 2010; Mochida and Shinozaki 2013). Relationships among the four sampled core tribes of Pooideae inferred by whole plastomes are consistent with most previous plastid trees, in which Brachypodieae is the sister group of a clade comprising a Bromeae–Triticeae lineage and Poeae (Catalán *et al.* 1997; Hilu *et al.* 1999; Duvall *et al.* 2007; Schneider *et al.* 2009; Davis and Soreng 2010; Grass Phylogeny Working Group II 2012; Blaner *et al.* 2014). This topology is also present in the nuclear *topoisomerase 6* phylogeny of Blaner *et al.* (2014). Two other nuclear-based phylogenies, however, infer alternative highly supported topologies that are discordant with each other and with the plastome phylogeny. The phytochrome B tree of Mathews *et al.* (2000) identifies *Diarrhena* as the sister group to the core Pooideae (excluding *Brachypodium*), with *Brachypodium* sister to this clade, whereas the β-amylase tree of Minaya *et al.* (2013) identifies a strongly supported (Brachypodieae + Diarrheneae), [Stipeae, (Lygeae + Nardeae)] clade that is sister to the rest of the core Poeae. The combined nuclear (ITS, β-amylase) and plastid tree in Minaya *et al.* (2013), however, has the same topology as our plastome tree with respect to the relationships among the core Pooideae, with all the relevant nodes strongly supported. This level of support is surprising given the strongly discordant β-amylase tree, which was incongruent with the ITS tree in a partition homogeneity test, as were the nuclear and plastid data (Minaya *et al.* 2013). Unfortunately, the ITS and plastid trees were not reported in that study and we are therefore unable to compare them with the β-amylase tree.

Additional nuclear genes have been sequenced for *Brachypodium* and representatives of Triticeae and Meliceae in the context of understanding the evolutionary history of *Brachypodium* species, but these studies did not have sufficient sampling to address the affinities of *Brachypodium* with respect to other Pooideae (Wolny *et al.* 2011; Catalán *et al.* 2012). Similarly, gene trees of nuclear loci coding for economically important traits, such as grain endosperm texture (Charles *et al.* 2009) have been studied, but these are based on data from the few sequenced grass genomes (rice, sorghum, wheat, *Brachypodium*), a level of taxon sampling that is insufficient for reconstructing finer-scale aspects of phylogeny. Although there is consensus on the position of *Brachypodium* in plastid-based phylogenies as the sister group of the rest of the core Pooideae, additional nuclear-based phylogenies with dense sampling are needed to distinguish gene trees from the species tree to characterize the precise affinities of *Brachypodium* and other pooid grasses, given the discordances among existing nuclear gene trees.

In the current study, the single plastome representative of Brachypodieae, the annual species *Brachypodium distachyon*, lies on a very long branch and is subtended by a very short one. Although the topologies are the same in five of the six MP, ML and BI trees, the branch length may be distorting the analyses. Available plastid data indicate that *B. distachyon* has the highest substitution rate in the genus. For example, within the crown *Brachypodium* clade in an *ndhF* phylogeny *B. distachyon* lies on a branch two and a half to eight times the length of the terminal branches of other taxa in the genus, which are perennials (Catalán *et al.* 1997). Rate variation between annual and perennial plant taxa has been widely documented (e.g. Yue *et al.* 2010; Gaut *et al.* 2011) and was recently demonstrated in grasses in plastome phylogenies of woody vs. herbaceous bamboos (Burke *et al.* 2012, 2014). Until recently *B. distachyon* was considered to be the only annual species in its genus, but the taxon has now been split into three annual species on the basis of morphological, cytogenetic and molecular data (Catalán *et al.* 2012). The plastome included here was sequenced from the Bd21 (2*n* = 10) genotype of *Brachypodium* (Bortiri *et al.* 2008) and corresponds to *B. distachyon* s.s. in the revised classification. Although an earlier phylogenetic analysis identified *B. distachyon* (s.l.) as the sister group of the rest of the genus (Catalán and Olmstead 2000), a more detailed study identifies two perennial species and one of the newly described annuals (*B. stacei*) as representing the first (but poorly supported) splits in the genus, with *B. distachyon* s.s. and the other newly described annual, *B. hybridum*, sister to a clade of the remaining perennial taxa (Catalán *et al.* 2012). Sampling plastomes from one of the core perennial species and one or more of the putative ‘early-diverging’ taxa would contribute to an understanding of plastome variation in the lineage and might break up the long branch in the crown *Brachypodium* clade, which may improve support in analyses of protein cds, and may possibly affect support levels elsewhere in the tree.

#### Poeae clade 1

As in previous plastid studies, the tribe Poeae is divided into two strongly supported clades, Poeae clades 1 and 2, that are further supported by 7 and ten unambiguous indels, respectively. The plastome phylogenies of Poeae clade 1 are maximally supported at all nodes in the ML and BI trees and at all but one node in the MP trees, and the topologies of the ML/BI and MP trees differ only in the relative placements of Aveninae and Phalaridinae–Torreyochloinae as the sister group of the rest of the clade. Our sampling represents all six subtribes that are part of Poeae clade 1 in Soreng *et al.* (2014). Brizinae is divided into two informal groups in the classification: Brizinae s.s., which includes the Old World genera *Airopsis* and *Briza* s.s., and Brizinae s.l. ‘Calotheca clade’, which includes the New World genera *Chascolytrum* s.l. (see Essi *et al.* 2011 for details on the recent expansion of *Chascolytrum*) and *Relchela*. This division is based on the results of phylogenetic work that shows Brizinae to be non-monophyletic. In an early study, Soreng and Davis (2000) found Brizinae to be paraphyletic and suggested that the subtribe represents parallel evolution of a ‘brizoid’ lemma (i.e. lemmas that are as long as broad) in Eurasia and South America. This paraphyly was supported by numerous subsequent phylogenetic studies: species of *Chascolytrum* s.l. and subtribe Agrostidinae form a clade, and *Briza* s.s. and *Airopsis* represent a distinct lineage that, in most analyses, is the sister group of the former clade (Davis and Soreng 2007; Döring *et al.* 2007; Quintanar *et al.* 2007; Soreng *et al.* 2007; Saarela *et al.* 2010; Grass Phylogeny Working Group II 2012). *Chascolytrum* s.l. is anomalous morphologically in the Agrostidinae clade as it has multiple florets per spikelet, compared with a single floret per spikelet, which is the diagnostic character for the subtribe. The distinction between these Old World and New World Brizinae lineages is clearly shown in Essi *et al.* (2008), although that study unfortunately did not include any closely related non-Brizinae taxa and thus the two Brizinae lineages appear to be reciprocally monophyletic sister taxa, which may not be accurate. Although we have not sampled the Brizinae s.l. ‘Calotheca clade’ here, our plastome trees similarly identify Brizinae s.s. as the sister group of Agrostidinae, with improved support compared with most earlier studies. Characterizing relationships between the Brizinae s.l. ‘Calotheca clade’ (i.e. *Chascolytrum* s.l.) and Agrostidinae taxa will require further work. The former group has been resolved as monophyletic in some, but not all studies, and its affinities with Agrostidinae taxa have been variously resolved (Davis and Soreng 2007; Quintanar *et al.* 2007; Soreng *et al.* 2007; Saarela *et al.* 2010). The monotypic *Relchela* has only been sampled in one study, where in an ITS tree it falls in a clade of Agrostidinae taxa (Refulio-Rodriguez 2007). We have sampled only two (*Agrostis*, *Ammophila*) of the ∼16 genera recognized in Agrostidinae, and these form a strongly supported clade. Other studies have similarly identified Agrostidinae as a monophyletic group (often including *Chascolytrum* s.l., as noted above) (Soreng and Davis 2000; Davis and Soreng 2007; Döring *et al.* 2007; Quintanar *et al.* 2007; Minaya *et al.* 2013) but further research is needed to clarify generic concepts and relationships within the subtribe (Saarela *et al.* 2010).

The strongly supported sister group relationship in our plastome trees between Anthoxanthinae and Brizinae–Agrostidinae has been found previously only in two plastid trees, in which this topology was weakly supported (Bouchenak-Khelladi *et al.* 2008; Saarela *et al.* 2010). This topology is incongruent with the placement of Anthoxanthinae in a strongly supported clade with Aveninae in other plastid, nuclear and combined trees (Davis and Soreng 2007; Döring *et al.* 2007; Quintanar *et al.* 2007; Schneider *et al.* 2009; Minaya *et al.* 2013) or sister to *Phalaris* (Soreng and Davis 2000). The reasons for these varying topologies are unclear, but may be related to density of taxon sampling, variable signal in different data partitions and/or rates of evolution in Poeae clade 1.

The strongly supported sister group relationship between Phalaridinae and Torreyochloinae in our plastome trees has not been identified previously. This may be because both lineages have been sampled together in only a few analyses, at least when considering the current circumscription of Phalaridinae. This subtribe has traditionally been circumscribed as including two or three genera, the closely related *Anthoxanthum* and *Hierochloe* (often included in *Anthoxanthum*) and *Phalaris*, which have a similar and unique spikelet structure in Poeae (Clayton and Renvoize 1986; Soreng *et al.* 2007). However, this circumscription has not been supported by most molecular studies—including the current one—which have identified *Phalaris* and *Anthoxanthum* s.l. as separate lineages (Döring *et al.* 2007; Quintanar *et al.* 2007; Bouchenak-Khelladi *et al.* 2008; Gillespie *et al.* 2008; Saarela *et al.* 2010; Minaya *et al.* 2013), hence their current recognition in monotypic subtribes. Within Anthoxanthinae, a molecular study has demonstrated the need for recognition of one or three genera (Pimentel *et al.* 2013). The shared spikelet characteristics of Anthoxanthinae and Phalaridinae may be plesiomorphies or may have evolved in parallel. Some studies represented Phalaridinae solely by *Anthoxanthum* (e.g. Soreng and Davis 2000), which in retrospect is uninformative in shedding light on the origins of Phalaridinae as now understood (since *Anthoxanthum* is part of a separate lineage). Torreyochloinae includes two genera, *Amphibromus* and *Torreyochloa*, a circumscription based on plastid and nuclear ribosomal phylogenies (Soreng and Davis 2000; Davis and Soreng 2007; Soreng *et al.* 2007; Saarela *et al.* 2010). One set of studies that sampled all three genera of Phalaridinae and Torreyochloinae was based on restriction site and morphological characters and found *Phalaris*, *Amphibromus* and *Torreyochloa* to be part of a clade of Aveneae taxa, but none of these three taxa was closely related (Soreng and Davis 1998, 2000). In Saarela *et al.* (2010)—so far the only study based on DNA sequence data to sample both subtribes and all three genera—all deep branches in plastid and nuclear ribosomal trees were weakly supported and/or unresolved. Although not incongruent with our current results, the unsupported trees in Saarela *et al.* (2010) do not provide support for the lineage.

Numerous studies have sampled either Phalaridinae or Torreyochloinae. In those that sampled only *Phalaris*, its placement was either unresolved (Döring *et al.* 2007) or variously inferred to be the sister group of the rest of Poeae clade 1 (Quintanar *et al.* 2007; Bouchenak-Khelladi *et al.* 2008, 2010; Gillespie *et al.* 2008; Schaefer *et al.* 2011), Agrostidinae (Minaya *et al.* 2013) or Aveninae (Grass Phylogeny Working Group II 2012). Studies that sampled only Torreyochloinae identified it as the sister group of the rest of Poeae clade 1 (Soreng and Davis 2000; Davis and Soreng 2007; Soreng *et al.* 2007) or Agrostidinae plus Brizinae s.s. (Davis and Soreng 2010). The studies that placed Phalaridinae or Torreyochloinae as sister to the rest of Poeae clade 1 are consistent with our ML and BI trees, in which the Phalaridinae–Torreyochloinae clade is strongly supported (at least based on complete plastome data) as the sister group of the rest of the clade. However, those previous studies and our ML and BI trees contrast with the topology of our MP trees, in which the Aveninae clade is weakly (complete plastome data) to strongly (protein cds) supported as the sister group of the rest of the clade. This latter set of relationships has not been found in other studies and likely represents systematic error in the MP tree here given the extremely short internal branches and long terminal branches at the base of the clade.

The novel relationship identified between Phalaridinae and Torreyochloinae requires consideration of possible non-molecular synapomorphies for this lineage. In terms of their gross morphologies, Phalaridinae and Torreyochloinae are distinctive, differing in their inflorescence shape, the number and fertility of florets per spikelet, glume length and the presence or absence of an awn. Phalaridinae is characterized by inflorescences of false spikes, spikelets laterally compressed with a single fertile floret and two proximal sterile lemmas that are shorter than the fertile lemma, glumes exceeding the florets, lemmas awnless and calluses glabrous (Voshell *et al.* 2011). Torreyochloinae is a morphologically heterogeneous subtribe. *Torreyochloa* is characterized by terminal paniculate inflorescences, spikelets laterally compressed to terete with 2–8 florets, glumes rounded to slightly keeled, unawned and shorter than the lowest lemma, lemmas 5–7-nerved (these prominent and scaberulous) and unawned and calluses glabrous (Watson and Dallwitz 1992 onwards; Davis 2007). It is one of several genera in Poeae clade 1 (also including genera of Brizinae s.l.) with spikelet structure characteristic of the traditional Poeae (i.e. simple spikelets with short glumes, several florets, 3–5-nerved lemmas vs. long glumes, 1–several florets, (3)5–11-nerved lemmas and/or geniculate dorsal awns in traditional Aveneae; Clayton and Renvoize 1986), which are mostly part of Poeae clade 1 (Soreng *et al.* 2007). Its sister taxon, *Amphibromus*, is characterized by terminal paniculate inflorescences, spikelets laterally compressed with 2–10(–12) fertile florets, glumes rounded to slightly keeled, unawned and shorter than or subequal to the lowest lemma, lemmas 2–4-toothed with teeth extending into short bristles, lemmas dorsally awned from about the middle and calluses hairy (Watson and Dallwitz 1992 onwards; Weiller *et al.* 2009). The spikelet structure of *Torreyochloa* may be plesiomorphic in Poeae, or may be a result of convergent evolution in Poeae clades 1 and 2 (Soreng *et al.* 1990; Soreng and Davis 2000), but a possible hybrid origin for this taxon should not be ruled out without supporting evidence. We are not able to identify any putative synapomorphies for the Phalaridinae–Torreyochloinae clade.

#### Poeae clade 2

Poeae clade 2 is strongly supported in our analyses, and comprises two major subclades based on the current sampling. One strongly supported subclade includes Dactylidinae, Holcinae, Loliinae and Airinae, and a sister relationship between Dactylidinae and Loliinae is supported in most analyses. In the ML and BI analyses, Airinae and Holcinae are identified as the successive sister groups of the rest of the subclade, whereas in one MP tree (complete data) these taxa are recovered as clade but with no support (BP <50), and in the other (protein cds) their relationships are unresolved. As in Poeae clade 1, these discordant topologies are likely a function of the very short branches at the base of the subclade. Recovery of this four-tribe subclade is consistent with previous studies that identified a larger clade comprising these subtribes as well as Ammochloinae, Cynosurinae, Parapholiinae and Sesleriinae, based on one to three plastid regions (Catalán *et al.* 2004; Davis and Soreng 2007; Döring *et al.* 2007; Quintanar *et al.* 2007; Soreng *et al.* 2007; Bouchenak-Khelladi *et al.* 2008; Schneider *et al.* 2009, 2012; Schaefer *et al.* 2011; Grass Phylogeny Working Group II 2012) and combined plastid (four regions) and nuclear (ITS and *β-amylase*) data (Minaya *et al.* 2013).

To properly interpret the results of earlier studies in the context of the current subtribal classification of Poeae, the recently revised nomenclature of some previously sampled taxa in light of knowledge of their evolutionary histories must be considered. Some species treated as *Avenula* (*A. albinervis*, *A. compressa*, *A. gervaisii*, *A. hookeri*, *A. pratensis*, *A. sulcata*) and *Helictotrichon* (*H. bromoides*, *H. schellianum*) are now recognized in the genus *Helictochloa* (Romero-Zarco 2011), which is included in Airinae, and *Deschampsia flexuosa* is now recognized in *Avenella* (Chiapella 2007), also included in Airinae. Only *Avenula pubescens* remains in *Avenula* s.s. (Romero-Zarco 2011), and it combines characters of *Helictotrichon* and *Helictochloa*, but its plastid is phylogenetically isolated from any of the above, possibly aligning with Poinae s.l. Soreng and Davis (2000), as yet unaware of the nrDNA problem, commented on the intermediate nature of *Avenula pubescens* between *Avenula* subgen. *Pratavenastrum* (now *Helictochloa*) and *Helictotrichon. Helictochloa* and *Deschampsia* are apparently part of a set of taxa (also including *Avenella*, *Sesleria*, *Scolochloa* and others) possibly derived from reticulation between plastid-based Poeae clade 1 and Poeae clade 2, or involved in that event or events. They and others align with traditional Poeae in plastid analysis (i.e. Poeae clade 2) and are intermediate between those and taxa with traditional Aveneae-type plastids (i.e. Poeae clade 1) in nrDNA trees (in nrDNA trees the Aveneae taxa arise from within Poeae, and the placements of various genera are quite incongruent) (e.g. Quintanar *et al*. 2007). This is a complex area that needs further study.

In earlier plastid trees, relationships among the lineages in the subclade were unresolved and/or weakly supported, with the exception of a sister group relationship inferred between Cynosurinae and Parapholiinae (Davis and Soreng 2007; Soreng and Gillespie 2007; Schneider *et al.* 2012; Minaya *et al.* 2013), and are not in conflict with the set of relationships inferred here. The combined nuclear and plastid tree in Minaya *et al.* (2013) is much better resolved, identifying the following strongly supported topology for this subclade: {Airinae (*Helictochloa bromoides*, as *Avenula bromoides*) [Dactylidinae (Cynosurinae, Parapholiinae)]}, and identifying a second clade comprising Holcinae, Airinae (*Avenella flexuosa*, as *Deschampsia flexuosa*, and *Corynephorus*) and Sesleriinae. Sessleriinae falls within Poeae clade 2 in plastid analyses, but is nested within Poeae clade 1 taxa in nrDNA analyses. *Sesleria* has a strange morphology, including a bract below the inflorescence and oddly shaped spikelets, that are suggestive of some disruption of the developmental patterns, possibly resulting from its reticulate origin (R. J. Soreng, pers. obs). The placement of Dactylidinae in the combined nuclear and plastid tree of Minaya *et al*. (2013) contrasts with its strongly supported placements as sister to Loliinae in our ML and BI trees based on the complete plastome data. We are not able to compare our plastome tree with their plastid tree, as it was not shown.

In earlier studies, two subtribes in the subclade were not monophyletic with respect to their current circumscriptions: Airinae, with *Aira*, *Avenella*, *Corynephorus* and *Periballia* comprising a lineage separate from *Helictochloa* (Davis and Soreng 2007; Döring *et al.* 2007; Quintanar *et al.* 2007; Soreng *et al.* 2007; Saarela *et al.* 2010; Schaefer *et al.* 2011; Minaya *et al.* 2013); and Holcinae, with a *Holcus*–*Vahlodea* clade and *Deschampsia* s.s. representing separate lineages (Davis and Soreng 2007; Döring *et al.* 2007; Quintanar *et al.* 2007; Saarela *et al.* 2010). We do not yet have sufficient plastome sampling to address the monophyly of these subtribes, having sampled only a single exemplar from each. A first strategy for future work should be to obtain plastomes from the currently unsampled subtribes Ammochloinae, Cynosurinae, Parapholiinae and Sesleriinae, and each of the putative lineages representing the non-monophyletic Airinae and Holcinae (at least two of these are currently unsampled). Further sampling in *Helictochloa* (some 30 species) and *Deschampsia* s.s. should aim to maximize the phylogenetic diversity in these genera as characterized in recent studies (Chiapella 2007; Winterfeld *et al.* 2014).

The second major subclade in Poeae clade 2 comprises representatives of Poinae and Coleanthinae. Like the other subclade, this major lineage has been identified in plastid trees in numerous studies (Davis and Soreng 2007; Gillespie *et al.* 2007; Quintanar *et al.* 2007; Soreng *et al.* 2007; Bouchenak-Khelladi *et al.* 2008; Schaefer *et al.* 2011; Grass Phylogeny Working Group II 2012; Schneider *et al.* 2012). Several subtribes that were recognized recently (Soreng *et al.* 2007), including Phleinae, Cinninae, Alopecurinae and Beckmanniinae, are now included in a more broadly defined subtribe Poinae on the basis of phylogenetic data, although numerous aspects of deep relationship in this large subtribe remain unresolved for plastid data (Gillespie *et al.* 2007, 2008, 2010). The maximally supported pattern of relationships in our plastome trees, with Coleanthinae identified as the sister group of a *Phleum*–*Poa* clade (i.e. Poinae), agrees with the earlier plastid trees of Gillespie *et al.* (2007, 2008). The affinities of the monotypic subtribe Miliinae, not sampled here, remain somewhat unclear and are discordant in plastid and nuclear ribosomal trees. In plastid trees the tribe lies on a very long branch, is weakly allied with *Phleum*, and the *Phleum*–Miliinae lineage is identified as the sister group of Poinae, but with poor support (Gillespie *et al.* 2008). In contrast, in nuclear ribosomal trees a weakly supported *Phleum*–Miliinae lineage falls outside the Poinae clade, with unclear affinities to a clade of Poinae taxa excluding *Poa*, a *Poa* clade and Coleanthinae (Gillespie *et al.* 2008, 2010). In combined plastid and nuclear analyses a weakly supported *Phleum*–Miliinae lineage is the sister group of a *Poa* clade (Gillespie *et al.* 2010), but this may not be an accurate reflection of evolutionary history given the discordance between the plastid and nuclear ribosomal data partitions. In a nuclear β-amylase tree, *Milium* is sister to a clade that includes Poinae plus a paraphyletic Coleanthinae, whereas in a combined nuclear (ITS, β-amylase) and plastid tree *Milium* is sister to Poinae, and the *Milium*-Poinae clade is sister to Coleanthinae (Minaya *et al.* 2013). Although plastome data for Miliinae may help clarify its maternal affinities with respect to *Phleum* and the rest of the Poinae clade, additional nuclear data will be necessary to reconstruct its possible hybrid origins.

Within Loliinae the plastome data indicate that *Lolium* is nested within a paraphyletic *Schedonorus*, that *Festuca altissima* (also known as *Schedonorus altissimus*) is more closely related to *Lolium*–*Schedonorus* (these being part of a ‘broad-leaved’ clade of *Festuca* s.l.) than *F. ovina* (‘fine-leaved’ clade), and that recognition of *Schedonorus* and *Lolium* as genera renders *Festuca* paraphyletic. All of these findings agree with the results of previous studies (Torrecilla and Catalán 2002; Catalán *et al.* 2004; Inda *et al.* 2008, 2014; Hand *et al.* 2010, 2013). In Soreng *et al.* (2014), *Schedonorus* is treated as a synonym of *Lolium*, based on the phylogeny in Catalán *et al.* (2009) and consistent with the plastome phylogeny here. The *Lolium*–*Schedonorus* clade lies on a fairly long branch, and is further supported by 14 unambiguous indels. *Schedonorus arundinaceus* is a hexaploid species complex comprising three morphologically and physiologically distinct forms recognized as Continental, Mediterranean and rhizomatous (Hand *et al.* 2010). Hand *et al.* (2010) sampled each of these forms and related species in the clade to reconstruct the evolutionary history of this agriculturally important pasture grass, and found each of the forms to have different origins. In their plastid tree, the rhizomatous and Continental forms were part of a clade that is the sister group of the clade including *Lolium* and *S. pratensis*, with the Mediterranean form placed elsewhere. *Schedonorus arundinaceus* was similarly not monophyletic in their nuclear ribosomal or other nuclear trees, and some aspects of their interrelationships differed with respect to the plastid tree.

Two complete plastomes are now available for *Schedonorus arundinaceus*, one of which was sequenced here. The two accessions of this species analysed in our 46-taxon trees are not resolved as a clade in either of the MP analyses **[see Supporting Information]**, as they are in the ML and BI trees; this is likely a function of the extremely long branch of the accession sequenced by Cahoon *et al.* (2010), rather than the possibility that the accessions may represent different forms. The previously published plastome and our new one for the species both represent the Contintental form—the most common form in North America. Cahoon *et al.* (2010) made this identification explicit, as they sampled ‘KY31’ [Kentucky 31], a widespread cultivar of the Continental form. Our plastome is from a field-collected specimen from British Columbia, which we identify as the Continental form based on its lack of rhizomes, the main diagnostic character for differentiating these morphotypes (Hand *et al.* 2010). This identification is consistent with BLAST comparisons of *matK* variation among our sample and those of the Continental and rhizomatous forms sequenced by Hand *et al.* (2010). The *matK* sequences of these samples differ by one substitution that varies within and among the two forms, compared with the more divergent *matK* sequences in the Mediterranean form, as illustrated in the *matK* tree in Hand *et al.* (2010). Although the BLAST search does not unambiguously identify our sample as either the Contintental or rhizomatous form, it does rule out the possibility of it being the Mediterranean form, at least based on current knowledge of variation in *matK* in that form.

Hand *et al.* (2013) included the *Schedonorus arundinaceus* plastome generated by Cahoon *et al.* (2010) in an analysis with four *Festuca*, *Lolium* and *Schedonorus* plastomes they generated, and observed considerable divergence among *S. arundinaceus* and the other Loliinae taxa, as we do here. They suggested that this divergence may be due to sequencing errors in the *S. arundinaceus* genome, and that additional plastomes should be generated from the species to determine whether the observed variation is real or artefactual. The previously sequenced plastome for *S. arundinaceus* shows 2.7 % difference from the new plastome from this taxon sequenced here. The top hits from a BLAST query of the previously sequenced regions *rpoC2* and *ccsA*, which contain numerous substitutions in the alignment, show the top hits to be the respective regions from species within the PACMAD clade (data not shown), suggestive of some errors in this genome. Variation in our new plastome from *S. arundinaceus* is more in line with the other three species in the *Lolium*–*Schedonorus* clade, further indicating problems with the earlier plastome. Inclusion of the long-branch *S. arundinaceus* genome differentially affects ML BS for the core Pooideae in the complete and protein-coding analyses compared with the 45-taxon analyses, for reasons that are unclear.

#### Bromeae–Triticeae

As in previous phylogenies, Bromeae and Triticeae are a strongly supported clade in our analyses, and the clade is further supported by 20 unambiguous indels. We have not sampled *Littledalea*, which is the sister group of Bromeae–Triticeae. The pattern of relationships among the three sampled Triticeae genera agree with plastid (Petersen and Seberg 1997; Mason-Gamer *et al.* 2002), nuclear (Mason-Gamer 2005) and combined plastid/nuclear phylogenies (Escobar *et al.* 2011) of the subtribe. An earlier version of the *T. aestivum* plastome (Ogihara *et al.* 2000) was recently found to contain sequence from the rice plastid genome (Bahieldin *et al.* 2014). This contaminated genome was included in an early version of our plastome matrix, and in our preliminary MP and ML analyses the terminal branch of *T. aestivum* was considerably longer than those of the other *Aegilops* and *Triticum* taxa in the trees, indicative of this error (data not shown). In the current matrix containing the corrected *T. aestivum* plastome sequence, the terminal branch for this taxon is very similar in length to those of all other taxa in the clade. Plastomes for the *Aegilops* and other *Triticum* species (except *T. aestivum*) were sequenced by Middleton *et al.* (2014). Their phylogeny depicting relationships between wheat, rye and barley was based on a 37 kb subset (<30 %) of the whole plastome. We included the full plastomes for these species in our analyses, and find the relationships among Triticeae taxa to be maximally supported and identical to those reported by Middleton *et al.* (2014).

### Unique plastome features

Within Pooideae the presence of the *rps16-trnQ* insertion solely in *Brachyelytrum* suggests that the loss of this insertion may be synapomorphic for the remaining genera. The insertion in the *rps16-trnQ* region of *Phaenosperma* and *Melica* is of note as, after *Brachyelytrum*, these two genera are the earliest to diverge in our analyses. A greater level of sampling within Phaenospermateae and Meliceae as well as sampling from the early diverging Nardeae and Lygeeae are needed to clarify the evolutionary history of these indels.

One insertion of mitochondrial origin was identified in the plastome of *Triticum monococcum*. This is currently the fourth documented case of plastome regions exhibiting mitochondrial homology. This type of gene transfer was first documented by Goremykin *et al.* (2009) in *Daucus carota*. The second was located by Straub *et al.* (2013) in *Asclepias* and the third was located by Wysocki *et al.* (2015) in two species within the Parianinae lineage of the Bambusoideae (*Eremitis* sp., *Pariana radiciflora*). While the first three documented insertions were extensively tested for erroneous assembly, the *T. monococcum* plastome was not sequenced by our team and cannot currently be verified.

One insertion of nuclear homology was identified in the plastome of *Triticum urartu*. The presence of this insertion, as well as the mitochondrial insertion, in the inverted repeat region suggests that it may have been retained due to the conserved nature of this region. This plastome was also not sequenced by our team so the presence of this insertion cannot be verified and may be an artefact of mis-assembly.

## Conclusions

Our phylogenomic analysis of whole plastomes resolve relationships at the base of Pooideae that have varied in earlier studies, and provide new insights into several aspects of relationship among tribes of Poeae, including a strongly supported novel relationship between Torreyochloinae and Phalaridinae. Plastomes representing Lygeae, Nardeae, Brylkinieae and *Littledalea* are needed to complete tribal-level plastome sampling, and several subtribes of Poeae are as yet unsampled. Our results demonstrate that inclusion of non-coding data in whole plastome analyses provides important characters for recovering robust support at deep nodes, compared with protein-coding data alone. Given rapid advances in next-generation sequencing, achieving a densely sampled plastome-based phylogeny of Pooideae is a realistic goal that we are working towards.

## Sources of Funding

This work was supported in part by the Plant Molecular Biology Center, the Department of Biological Sciences at Northern Illinois University and the National Science Foundation under grant numbers DEB-1120750 to L.G.C., DEB-1120856 to S.A.K. and DEB-1120761 to M.R.D. and DEB-0830020 to J.I.D. Any opinions, findings and conclusions or recommendations expressed in this material are those of the authors and do not necessarily reflect the views of the National Science Foundation.

## Contributions by the Authors

M.R.D., S.A.K., L.G.C. and J.I.D. conceived and designed the study. J.C.P., P.P.E. and D.R.M. provided technical support. W.P.W., C.F.B., M.R.D., J.I.D., J.M.S. and R.J.S. acquired the data. W.P.W., M.R.D. and J.M.S. analysed and interpreted the data. J.M.S., W.P.W. and M.R.D. wrote the manuscript. J.M.S., W.P.W., M.R.D., J.I.D., R.J.S., C.F.B., S.A.K. and L.G.C. contributed to revising the manuscript.

## Conflict of Interest Statement

None declared.

## Supporting Information

The following additional information is available in the online version of this article –

**Table S1.** Summary of the Pooideae classification of Soreng *et al.* (2014).

**Table S2.** List of completed and publicly available whole plastomes in Poaceae (as of 25 September 2014).

**Figure S1.** ML phylogram of complete plastomes of 45 pooid grasses and one outgroup taxon, including the highly divergent, previously banked sequence of *Schedonorus arundinaceus* (taxon in **bold**; GenBank accession NC_011713). Bootstrap values are indicated only when at least one is <100 (ML bootstrap value precedes MP bootstrap value). ML and BI topologies were identical. Posterior probabilities at all nodes = 1.00. ‘NR’ indicates a node not resolved or supported above the 50 % bootstrap level in the MP analysis. In the MP analysis, the boldfaced *S. arundinaceus* is sister to a clade comprising the other *S. arundinaceus* sample, *Festuca pratensis*, *Lolium multiflorum* and *L. perenne*; the latter clade receives 71 % BS. Clades marked with a cross are reversed in the MP tree. The MP bootstrap value of the node affected by the reversal of these clades reflects that of the reversed topology. Taxa marked with diamonds are a maximally supported clade in the MP tree.

**Figure S2.** ML phylogram of the protein cds of 45 pooid grasses and one outgroup taxon, including the highly divergent, previously banked sequence of *Schedonorus arundinaceus* (taxon in **bold**). Support values are indicated only when at least one is less than the maximum possible value (ML bootstrap value precedes MP bootstrap value, which precedes posterior probability value). ML and MP topologies were identical. ‘NR’ indicates a node not resolved in the strict consensus of most parsimonious trees or not supported above the 50 % bootstrap level. In the MP analysis, the boldfaced *S. arundinaceus* is sister to a clade comprising the other *S. arundinaceus* sample, *Festuca pratensis*, *Lolium multiflorum* and *L. perenne*; the latter clade receives 97 % BS. A star indicates clades that were reversed in the BI tree. The posterior probability of the node affected by the reversal of these clades reflects that of the reversed topology.

**Dataset S1.** Details of scored indels in the 46-taxon matrix.

Additional Information
