# Supplementary material for: Plastid phylogenomics of the cool-season grass subfamily: clarification of relationships among early-diverging tribes
Source: AoB Plants. 2015 May 2;7:plv046. doi: 10.1093/aobpla/plv046 (PMC4480051; doi:10.1093/aobpla/plv046)
Supplement: Additional Information [file supp_7_plv046_index.html]

Plastid phylogenomics of the cool-season grass subfamily: clarification of relationships among early-diverging tribes — Additional Information 

# Plastid phylogenomics of the cool-season grass subfamily: clarification of relationships among early-diverging tribes

## Additional Information

Additional Information

**Files in this Data Supplement:**

- Supplementary Figure 1 - pptx file
- Supplementary Figure 2 - pptx file
- Supplementary Table 1 - docx file
- Supplementary Table 2 - docx file
- Supplementary Dataset 1 - docx file
