# Supplementary figures and images for: Plastid phylogenomics of the cool-season grass subfamily: clarification of relationships among early-diverging tribes
Source: AoB Plants. 2015 May 2;7:plv046. doi: 10.1093/aobpla/plv046 (PMC4480051; doi:10.1093/aobpla/plv046)

## Slide 1
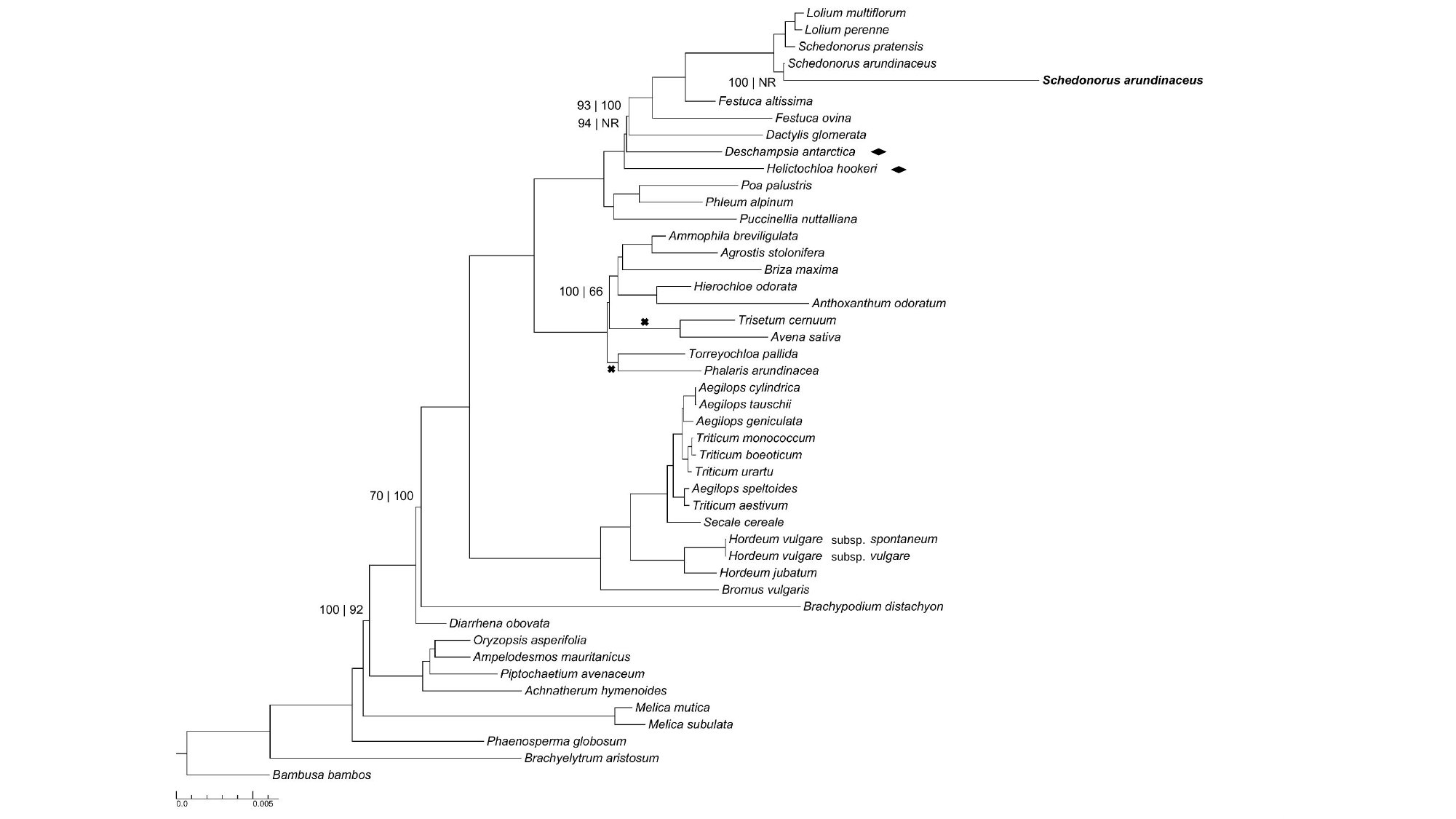

subsp.
subsp.

Supplement: Additional Information [file supp_plv046_plv046supp_fig1.pptx]

## Slide 1
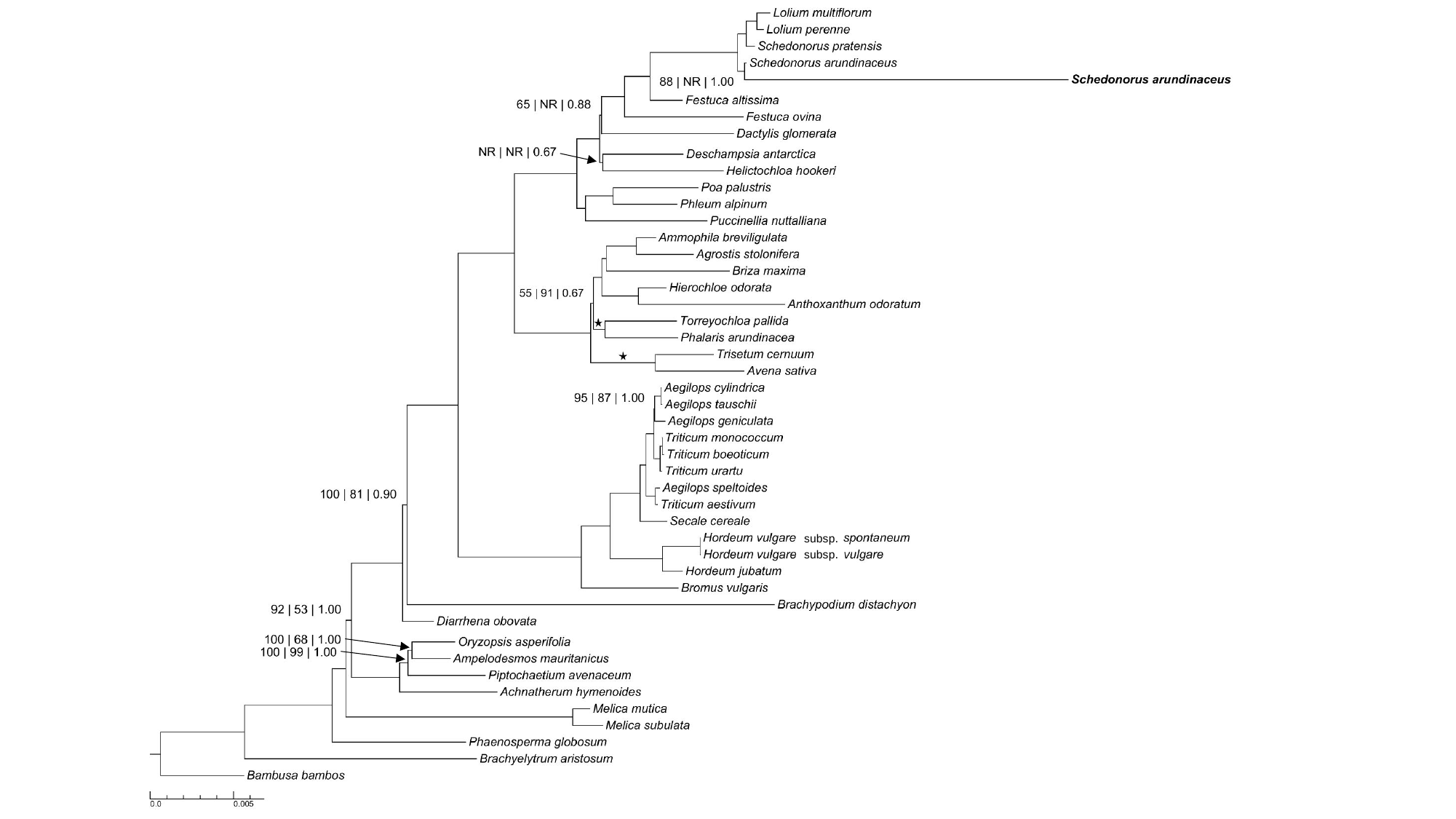

subsp.
subsp.

Supplement: Additional Information [file supp_plv046_plv046supp_fig2.pptx]
