# Supplementary material for: Plastid phylogenomics of the cool-season grass subfamily: clarification of relationships among early-diverging tribes
Source: AoB Plants. 2015 May 2;7:plv046. doi: 10.1093/aobpla/plv046 (PMC4480051; doi:10.1093/aobpla/plv046)
Supplement: Additional Information [file supp_plv046_plv046supp_table1.docx]

**Table S1.** Summary of the Pooideae classification of Soreng *et al.* (2014).

subfam. **Pooideae** Benth. [1861]

trib. **Brachyelytreae** Ohwi [1941] (syn. -- Brachyelytrinae Ohwi [1942]): *Brachyelytrum*.

trib. **Nardeae** W.D.J. Koch. [1837] (syn. -- subtrib. Nardinae Kromb. [1875]) [sister to Lygeeae]: *Nardus*

trib. **Lygeeae** J. Presl [1846] (syn. -- Lygeinae Röser [2009], Spartineae Trin. [1824, nom. inval., based on *Lygeum*]) [sister to Nardeae]: *Lygeum*.

trib. **Phaenospermateae** Renvoize & Clayton [1985] (syn. – Duthieeae Roser & Jul.Schneider [2011]):  *Phaenosperma*,*Sinochasea*,*Stephanachne*.

subtrib. **Duthieinae** Pilg. ex Potztal [1969]: *Anisopogon*, *Danthoniastrum*, Duthiea s.l. (incl. *Triavenopsis*?), *Metcalfia*, *Pseudodanthonia*.

trib. **Ampelodesmeae** Tutin [1978] (syn. – Ampelodesminae Conert [1961]): *Ampelodesmos* [apparently an ancient hybrid between parents from Stipeae and Phaenospermateae; see Romaschenko *et al.*, 2013]

trib. **Stipeae** Dumort. [1824] (syn. --  supertrib. Stipodae L. Liu [1980]):

subtrib. **Stipinae** Griseb. [1846] (syn. -- Aciachninae Caro [1982], Ortachninae Caro [1982]):*Achnatherum* [OW, incl.*Oloptum*; NA species are in limbo, none belong in *Achnatherum* s.s., most are *Eriocoma* but not yet transferred], ×*Achnella* [needs new hybrid genus *Eriocoma* × *Nassella*], *Aciachne*, *Amelichloa* [nested within *Nassella*, but an inter-generic hybrid origin has not been ruled out], *Anemanthele*, *Anatherostipa* (incl. *Nicoraella*), *Aristella*, *Austrostipa*, *Celtica*, *Eriocoma*, *Hesperostipa*, *Jarava*, *Lorenzochloa*, *Macrochloa*, *Nassella*, *Ortachne*, *Orthoraphium*, *Oryzopsis*, *Pappostipa*, *Patis*, *Piptochaetium*, *Piptatheropsis*, *Piptatherum, Psammochloa*, *Ptilagrostis*, *Stipa*, *Stipellula* (*Stipella* nom. illeg. hom.), *Timouria*, *Trikeraia*.

trib. **Brylkinieae** Tateoka [1960] [sister to Meliceae, may be better in Meliceae as subtribe] (syn. -- Brylkiniinae Ohwi [1941]): *Brylkinia*, *Koordersiochloa* (incl. *Streblochaete*) [doubt about placement within Meliceae s.s., due to presence of cylindrical to lanceoloid (non-globose) styles, puctiform hilum]

trib. **Meliceae** Link ex Endl. [1830] (syn. -- Glycerieae Link ex Endl. [1830]; subtrib. Glyceriinae Dumort. [1829], Melicinae Fr. 1835]): *Glyceria*, *Lycochloa*, *Melica*, *Pleuropogon*, *Schizachne*, *Triniochloa*.

trib. **Diarrheneae** C.S. Campb. [1985] (syn. -- subtrib. Diarrheninae Ohwi [1941]): *Diarrhena*, *Neomolinia*.

trib. **Brachypodieae** Harz [1880] (syn. -- subtrib. Brachypodiinae Hack. [1887]; Brachypodieae Hayek [1925] isonym.): *Brachypodium* (incl. *Trachynia*)

supertrib. **Poodae** L. Liu [1980] (syn. -- Poodae T.D. Macfarl. & L. Watson [1982], isonym):

trib. **Poeae** R.Br. [1814] (syn. -- Agrostideae Martinov [1820][as Koleno = tribe, indirect ref. to Kunth], Agrostidieae Dumort. [1824], Airopsideae Gren. & Godr. [1855], Alopecureae W.D.J. Koch [1837], Anthoxantheae Link ex Endl. [1830], Aveneae Dumort. [1824], Beckmannieae Nevski [1937], Calamagrostideae Trin. [1824], Cinneae Ohwi [1941], Coleantheae Husn. [1896], Cynosureae Dumort. [1824], Dupontieae A. Löve & D. Löve, [1961, nom. nud.], Festuceae Dumort. [1824], Gaudinieae Rouy [1913], Graphephoreae (Asch. & Graebn.) Hyl. [1953], Hainardieae Greuter [1967], Holceae J. Presl [1846], Lolieae Link ex Endl. [1830], Koelerieae Schur nom. nud. [1866], Milieae Link ex Endl. [1830], Phalarideae Kunth [1829], Phleeae Dumort. [1824], Scolochloeae Tzvelev [1968], Seslerieae W.D.J. Koch [1837], Triseteae Gren. & Godr. [1855], Vilfeae Trin. [1824]):

Poeae CHLOROPLAST GROUP 1 (Aveneae type):

subtrib. **Torreyochloinae** Soreng [2003]: *Amphibromus*, *Torreyochloa*.

subtrib. **Aveninae** J. Presl [1830] (syn. -- Gaudiniinae Holub ex Tzvelev [1976, nom. nud.], Graphephorinae Asch. & Graebn. [1900], Koeleriinae Asch. & Graebn. [1900]): *Arrhenatherum*,  *Avellinia*, *Avena*, *Gaudinia,* *Graphephorum*, *Helictotrichon*  s.s. (incl. *Pseudarrhenathrum*; exclud. *Avenula*, *Helictochloa*), *Koeleria* (incl. *Parafestuca*), *Lagurus*, *Leptophyllochloa*, *Peyritschia*, *Rostraria*, *Sphenopholis*, *Trisetaria*, *Tricholemma*, *Trisetum*.

subtrib. **Phalaridinae** Fr. [1835]: *Phalaris*.

subtrib. **Anthoxanthinae** A. Gray [1856] (syn. -- Foenodorinae Krause [1909, nom. inval.]): *Anthoxanthum* (incl. *Ataxia*, *Hierochloe*).

subtrib. **Brizinae** Tzvelev s.s. [1968]: *Airopsis*, *Briza* (incl. *Macrobriza*; excl. *Brizochloa*?).

subtrib. **Brizinae** s.l. “Calotheca clade”: *Chascolytrum* (incl. *Calotheca*,*Erianthecium*,*Gymnachne*,*Lombardochloa*,*Microbriza*, *Poidium*,*Rhombolytrum*), *Relchela* [this group should be a separate subtribe].

subtrib. **Agrostidinae** Fr. [1835] (syn. -- Chaeturaceae Link [1827] nom. rankless, Calamagrostidinae Lindl. [1836, nom. nud.], Vilfinae Steud. [1954]): ×*Agropogon*, *Agrostis*, *Ammophila*, *Ancistragrostis*, *Bromidium*, *Calamagrostis* p.p. (incl. *Deyeuxia*) [polyphyletic, p.p., New World], *Chaetopogon*, *Dichelachne*, *Echinopogon*, *Hypseochloa*, *Gastridium*, *Lachnagrostis*, *Limnodea* [tentatively placed in Agrostidinae rather than Cinninae], *Pentapogon*, *Podagrostis*, *Polypogon*, *Triplachne*.

Poeae CHLOROPLAST GROUP 2 (Poeae type):

*Incertae Sedis*: *Avenula* (s.s. p.p. typica -- *A*.*pubescens*; syn. -- *Homalotrichon*; exclud. *Helictochloa*) , [*Avenula* s.s. is a floater, probably allied to the set of Coleanthinae, Poinae s.l., Miliinae, Phleinae]

subtrib. **Scolochloinae** Tzvelev [1987] (syn. – subtrib. Scolochloeae Tzvelev [1968]): *Dryopoa*, *Scolochloa* [this subtribe seems to share plastids with the old Poeae and nrDNA with early Aveninae GROUP 1 above].

subtrib. **Sesleriinae** Parl. [1845] (syn. -- subtrib. Miborinae Asch. & Graebn. [1899]): *Mibora*, *Echinaria*, *Oreochloa*, *Pappagrostis*, *Sesleria*, *Sesleriella* [this subtribe also seems to share plastids with the old Poeae and nrDNA with early Aveninae GROUP 1 above].

subtrib. **Coleanthinae** Rouy [1913] (syn. -- Puccinelliinae Soreng & Davis [2003]): *Catabrosa*, *Catabrosella*, *Coleanthus*, *Colpodium* (incl. *Keniochloa*), *Hyalopoa* [apparently heterogenous], *Paracolpodium*[apparently heterogenous], *Phippsia*, ×*Pucciphippsia*, *Puccinellia* (incl. *Pseudosclerochloa*), *Sclerochloa*, *Zingeria* [probably best united with *Colpodium* s.s.].

subtrib. **Miliinae** Dumort. [1829]: *Milium* [possibly part of Poinae s.l., possibly sister to *Poa* or *Phleum*]

subtrib. **Poinae** Dumort. [1829] (syn. -- subtrib. Gramininae Krause [1909, nom. inval.], Ventenatinae Holub [1958, nom. nud.; Tzvelev, 1976, nom. inval., without Latin]): [at least 9 subgroups are evident; 6, 7, 8, and some in 3, 4, are awned]

1. *Poa* s.s. (incl. *Anthochloa*, *Aphanelytrum*, *Austrofestuca*, *Dissanthelium*, *Eremopoa*, *Libyella*, *Lindbergella*, *Neuropoa*, *Oreopoa*, *Parodiochloa*, *Tovarochloa*, *Tzvelevia*);

2. (incl. subtrib. **Phleinae** Dumort. [1868]; syn. -- Phleinae Benth. [1881]): *Phleum* s.s. (incl. *Maillea*) [*Phleum* s.s. seems to be sister to *Poa* s.s. or possibly *Milium*];

3. (incl. subtrib. **Cinninae** Caruel. [1892]): *Agrostopoa*, *Aniselytron* [apparently contains some *Poa* nrDNA], *Cinna* s.l., *Cyathopus*, *Simplicia*, *Arctopoa* [ancient × between some unknown ancestor, possibly from group 3, with chloroplast from *Poa*]

4. [generally resolved as a set branching from within or as sister to Cinninae group 3] *Arctagrostis*, *Hookerochloa* (incl. *Festucella*), *Nicoraepoa* [hybrids with *Poa* are known], *Saxipoa*, *Sylvipoa*; ×*Duarctopoa* [*Dupontia* × *Arctopoa*];

5. *Pseudophleum* [seems to be the sister to groups 6+7+8+9];

6. (incl. subtrib. **Alopecurinae** Dumort. [1829]): *Alopecurus*, *Cornucopiae*, *Limnas*, *Rhizocephalus*;

7. [a hybrid-lineage, entangled with Alopecurinae and other Poinae] ×*Arctodupontia*, *Arctophila*, *Dupontia*, *Dupontiella*;

8. [sister to Alopecurinae if 7 is not considered, *Beckmannia* still a problem] *Apera*, *Bellardiochloa*, *Gaudiniopsis*, *Nephelochloa*, *Parvotrisetum*, *Ventenata*;

9. (incl. subtrib. **Beckmanniinae** Nevski [1937]):*Beckmannia*; *Pholiurus* [possible sister to *Beckmannia*?].

subtrib. **Holcinae** Dumort. [1868] (syn. -- Deschampsinae Holub [1958, nom. nud.], Scribneriinae Soreng [2003]): *Deschampsia* s.s. [excluding *Avenella*] (incl. *Scribneria*), *Holcus*, *Vahlodea*.

subtrib. **Airinae** Fr. [1835] (syn. -- Corynephorinae subtrib V. Jirasek & Chrtek) [a heterogenous subtribe with no satisfactory resolution]: *Aira*, *Antinoria*, *Avenella*, *Corynephorus*, *Helictochloa*  [incl. *Avenula* p.p. non-typica, *A*. subg. *Pratavenastrum*), *Molineriella*, *Periballia*.

subtrib. **Loliinae** Dumort. [1829] (syn. -- Festucinae J. Presl [1830], Psilurinae Pilg. ex Potztal [1969]): *Castellia*, *Drymochloa*, *Festuca* (incl. *Ctenopsis*, *Dielsiochloa*, *Hellerochloa*, *Loliolum*, *Micropyrum*, *Narduroides*, *Psilurus*, *Vulpia*, *Wangenheimia*), *Leucopoa*(incl.*Xanthochloa*), *Lolium* (incl. *Micropyropsis*, *Schedonorus*), *Megalachne*, *Patzkea*, *Podophorus*, *Pseudobromus* [the latter seems odd here; DNA data show a long branch, but outgroup selection has not clarified its placement].

subtrib. **Dactylidinae** Stapf [1898]: *Dactylis*, *Lamarckia*

subtrib. **Cynosurinae** Fr. [1835]: *Cynosurus*

subtrib. **Ammochloinae** Tzvelev [1976]: *Ammochloa*.

subtrib. **Parapholiinae** Caro [1982] (syn. -- Monerminae Tzvelev [1987, nom. inval.]): *Agropyropsis*, *Catapodium*, *Cutandia*, *Desmazeria*, *Hainardia*, *Parapholis*, *Sphenopus*, *Vulpiella*
