# Supplementary material for: Plastid phylogenomics of the cool-season grass subfamily: clarification of relationships among early-diverging tribes
Source: AoB Plants. 2015 May 2;7:plv046. doi: 10.1093/aobpla/plv046 (PMC4480051; doi:10.1093/aobpla/plv046)
Supplement: Additional Information [file supp_plv046_plv046supp_table2.docx]

**Table S2.** List of completed and publicly available whole plastomes in Poaceae (as of 25 September 2014).

| **Subfamily** | **Taxon** | **Reference** |
| --- | --- | --- |
| Anomochlooideae | *Anomochloa marantoidea* | Morris and Duvall (2010) |
| Bambusoideae | *Acidosasa purpurea* | Zhang *et al.* (2011) |
| Bambusoideae | *Ampelocalamus calcareus* | Ma *et al.* (2014) |
| Bambusoideae | *Arundinaria appalachiana* | Burke *et al.* 2014 |
| Bambusoideae | *Arundinaria faberi* | Ma *et al.* (2014) |
| Bambusoideae | *Arundinaria fargesii* | Ma *et al.* (2014) |
| Bambusoideae | *Arundinaria gigantea* | Burke *et al.* (2012) |
| Bambusoideae | *Arundinaria tecta* | Burke *et al.* (2014) |
| Bambusoideae | *Bambusa emeiensis* | Zhang *et al.* (2011) |
| Bambusoideae | *Bambusa oldhamii* | Wu *et al.* (2009) |
| Bambusoideae | *Bergbambos tessellata* | Ma *et al.* (2014) |
| Bambusoideae | *Chimonocalamus longiusculus* | Ma *et al.* (2014) |
| Bambusoideae | *Cryptochloa strictiflora* | Burke *et al.* (2012) |
| Bambusoideae | *Dendrocalamus latiflorus* | Wu *et al.* (2009) |
| Bambusoideae | *Fargesia nitida* | Ma *et al.* (2014) |
| Bambusoideae | *Fargesia spathacea* | Ma *et al.* (2014) |
| Bambusoideae | *Fargesia yunnanensis* | Ma *et al.* (2014) |
| Bambusoideae | *Ferrocalamus rimosivaginua* | Zhang *et al.* (2011) |
| Bambusoideae | *Gaoligongshania megalothyrsa* | Ma *et al.* (2014) |
| Bambusoideae | *Gelidocalamus tessellatus* | Ma *et al.* (2014) |
| Bambusoideae | *Indocalamus longiauritus* | Zhang *et al.* (2011) |
| Bambusoideae | *Indocalamus sinicus* | Ma *et al.* (2014) |
| Bambusoideae | *Indocalamus wilsonii* | Ma *et al.* (2014) |
| Bambusoideae | *Indosasa sinica* | Ma *et al.* (2014) |
| Bambusoideae | *Oldeania alpina* | Ma *et al.* (2014) |
| Bambusoideae | *Oligostachyum shiuyingianum* | Ma *et al.* (2014) |
| Bambusoideae | *Olyra latifolia* | Burke *et al.* (2014) |
| Bambusoideae | *Phyllostachys edulis* | Zhang *et al.* (2011) |
| Bambusoideae | *Phyllostachys nigra* var. *henonis* | Zhang *et al.* (2011) |
| Bambusoideae | *Phyllostachys propinqua* | Wu and Ge (2012) |
| Bambusoideae | *Phyllostachys sulphurea* | Gao and Gao (2014) |
| Bambusoideae | *Pleioblastus maculatus* | Ma *et al.* (2014) |
| Bambusoideae | *Thamnocalamus spathiflorus* | Ma *et al.* (2014) |
| Bambusoideae | *Yushania levigata* | Ma *et al.* (2014) |
| Ehrhartoideae | *Leersia tisserantii* | Wu and Ge (2012) |
| Ehrhartoideae | *Oryza nivara* | Shahid *et al.* (2004) |
| Ehrhartoideae | *Oryza rufipogon* | Lin *et al.* (2014) |
| Ehrhartoideae | *Oryza sativa* | Tang *et al.* (2004) |
| Ehrhartoideae | *Oryza sativa* cv. Nipponbare | Hiratsuka *et al.* (1989) |
| Ehrhartoideae | *Oryza sativa* cv. PA645 | Tange *et al.* (2004) |
| Ehrhartoideae | *Rhynchoryza subulata* | Wu and Ge (2012) |
| Panicoideae | *Coix lacryima-jobi* | Leseberg and Duvall (2007) |
| Panicoideae | *Lecomtella madagascariensis* | Besnard *et al.* (2013) |
| Panicoideae | *Miscanthus* ×*giganteus* | Diekmann *et al.* (2008) |
| Panicoideae | *Saccharum officinarum* cv. NCo310 | Asano *et al.* (2004) |
| Panicoideae | *Saccharum officinarum* hybrid SP-80-3280 | Calsa *et al.* (2004) |
| Panicoideae | *Sorghum bicolor* | Saski *et al.* (2007) |
| Panicoideae | *Panicum virgatum* Kanlow Lin1 | Young *et al.* (2011) |
| Panicoideae | *Panicum virgatum* Summer Lin2 | Young *et al.* (2011) |
| Panicoideae | *Zea mays* | Maier *et al.* (1995) |
| Pharoideae | *Pharus lappulaceus* | Jones *et al.* (2014) |
| Pharoideae | *Pharus latifolius* | Jones *et al.* (2014) |
| Pooideae | *Aegilops bicornis* | Gornicki *et al.* (2014) |
| Pooideae | *Aegilops cylindrica* | Middleton *et al.* (2014) |
| Pooideae | *Aegilops geniculata* | Middleton *et al.* (2014) |
| Pooideae | *Aegilops kotschyi* TA1980 | Gornicki *et al.* (2014) |
| Pooideae | *Aegilops longissima* TA1924 | Gornicki *et al.* (2014) |
| Pooideae | *Aegilops searsii* TA1837 | Gornicki *et al.* (2014) |
| Pooideae | *Aegilops searsii* TA1841 | Gornicki *et al.* (2014) |
| Pooideae | *Aegilops searsii* TA1926 | Gornicki *et al.* (2014) |
| Pooideae | *Aegilops sharonensis* TA1995 | Gornicki *et al.* (2014) |
| Pooideae | *Aegilops sharonensis* TA1996 | Gornicki *et al.* (2014) |
| Pooideae | *Aegilops speltoides* | Middleton *et al.* 2014 |
| Pooideae | *Aegilops speltoides* subsp. *ligustica* AE918 | Gornicki *et al.* (2014) |
| Pooideae | *Aegilops speltoides* subsp. *ligustica* TA1796 | Gornicki *et al.* (2014) |
| Pooideae | *Aegilops speltoides* subsp. *speltoides* | Gornicki *et al.* (2014) |
| Pooideae | *Aegilops tauschii* | Middleton *et al.* (2014) |
| Pooideae | *Aegilops tauschii* | Gornicki *et al.* (2014) |
| Pooideae | *Agrostis stolonifera* | Saski *et al.* (2007) |
| Pooideae | *Brachypodium distachyon* | Bortiri *et al.* (2008) |
| Pooideae | *Deschampsia antarctica* | Lee *et al.* (2014) |
| Pooideae | *Festuca altissima* | Hand *et al.* (2013) |
| Pooideae | *Festuca arundinacea* | Cahoon *et al.* (2010) |
| Pooideae | *Festuca ovina* | Hand *et al.* (2013) |
| Pooideae | *Festuca pratensis* | Hand *et al.* (2013) |
| Pooideae | *Hordeum spontaneum* FT11 | Middleton *et al.* (2014) |
| Pooideae | *Hordeum spontaneum* FT462 | Middleton *et al.* (2014) |
| Pooideae | *Hordeum vulgare* cv*.* Barke | Middleton *et al.* (2014) |
| Pooideae | *Hordeum vulgare* subsp*. vulgare* | Saski *et al.* (2007) |
| Pooideae | *Lolium multiflorum* | Hand *et al.* (2013) |
| Pooideae | *Lolium perenne* | Diekmann *et al.* (2008) |
| Pooideae | *Secale cereale* | Middleton *et al.* (2014) |
| Pooideae | *Triticum aestivum* | Ogihara *et al.* (2002); Bahieldin *et al.* (2014) |
| Pooideae | *Triticum aestivum* (Chinese Spring) | Middleton *et al.* (2014) |
| Pooideae | *Triticum aestivum* subsp. *aestivum* | Gornicki *et al.* (2014) |
| Pooideae | *Triticum aestivum* subsp. *spelta* | Gornicki *et al.* (2014) |
| Pooideae | *Triticum boeoticum* | Middleton *et al.* (2014) |
| Pooideae | *Triticum monococcum* | Middleton *et al.* (2014) |
| Pooideae | *Triticum timopheevii* subsp. *armeniacum* TA0941 | Gornicki *et al.* (2014) |
| Pooideae | *Triticum timopheevii* subsp. *armeniacum* TA0944 | Gornicki *et al.* (2014) |
| Pooideae | *Triticum timopheevii* subsp. *armeniacum* TA1485 | Gornicki *et al.* (2014) |
| Pooideae | *Triticum timopheevii* subsp. *timopheevii* | Gornicki *et al.* (2014) |
| Pooideae | *Triticum turgidum* subsp. *carthlicum* 2836 | Gornicki *et al.* (2014) |
| Pooideae | *Triticum turgidum* subsp. *carthlicum* TA2801 | Gornicki *et al.* (2014) |
| Pooideae | *Triticum turgidum* subsp. *dicoccoides* TA0060 | Gornicki *et al.* (2014) |
| Pooideae | *Triticum turgidum* subsp. *dicoccoides* TA0073 | Gornicki *et al.* (2014) |
| Pooideae | *Triticum turgidum* subsp. *dicoccoides* TA1133 | Gornicki *et al.* (2014) |
| Pooideae | *Triticum turgidum* subsp. *durum* | Gornicki *et al.* (2014) |
| Pooideae | *Triticum urartu* | Middleton *et al.* (2014) |
| Pooideae | *Triticum urartu* | Gornicki *et al.* (2014) |
| Puelioideae | *Puelia olyriformis* | Jones *et al.* (2014) |
