# Supplementary material for: Plastid phylogenomics of the cool-season grass subfamily: clarification of relationships among early-diverging tribes
Source: AoB Plants. 2015 May 2;7:plv046. doi: 10.1093/aobpla/plv046 (PMC4480051; doi:10.1093/aobpla/plv046)
Supplement: Additional Information [file supp_plv046_plv046supp_table3.docx]

| 1 | Schedonorus arundinaceus Schedonorus arundinaceus[C] Schedonorus pratensis  Festuca altissima  Festuca ovina  Lolium multiflorum  Lolium perenne  Dactylis glomerata  Helictochloa hookeri  Deschampsia antarctica  Poa palustris  Phleum alpinum  Briza sp  Puccinellia nuttalliana  Ammophila breviligulata  Agrostis stolonifera  Hierochloe odorata  Anthoxanthum odoratum  Phalaris arundinacea  Torreyochloa  Avena sativa  Trisetum cernuum  Aegilops cylindrica  Aegilops geniculata  Aegilops speltoides  Aegilops tauschii  Triticum aestivum  Triticum monococcum aegilopoides Triticum monococcum (modified) Triticum urartu (modified)  Hordeum jubatum  Hordeum vulgare  Hordeum vulgare spontaneum  Secale cereale  Bromus vulgaris  Brachypodium distachyon  Diarrhena obovata  Melica mutica  Melica subulata  **Oryzopsis asperifolia Achnatherum hymenoides Ampelodesmos mauritanica Piptochaetium avenaceum**  Phaenosperma globosum Brachyelytrum aristosum  Bambusa bambosa | 1158 | IGS | AAACC----TTATCC  AAACC---TTTATCC  AAACC----TTATCC  AAACC----TTATCC  AAACC----TTATCC  AAACC----TTATCC  AAACC----TTATCC  AAACC----TTATCC  AAACC----TTATCC  AAACC----TTATCC  AAACC----TTATCC  AAACC----TTATCC  AAACC----TTATCC  AAACC----TTATCC  AAACC----TTATCC  AAACC----TTATCC  AAACC----TTATCC  AAACT----TTATCC  AAACC----TTATCC  AAACC----TTATCC  AAACC----TTATCC  AAACC----TTATCC  AAACC----TTATCC  AAACC----TTATCC  AAACC----TTATCC  AAACC----TTATCC  AAACC----TTATCC  AAACC----TTATCC  AAACC----TTATCC  AAACC----TTATCC  AAACC----TTATCC  AAACC----TTATCC  AAACC----TTATCC  AAACC----TTATCC  AAACC----TTATCC  AAACC----TTATCC  AAACC----TTATCC  AAACC----TTATCC  AAACC----TTATCC  AAGCCTTATTTATCC  AAGCCTTATTTACCC  AAGCCTTTTTTATCC  AAGCCTTATTTATCC  AAGCC----TTATCC  AAGCC----TTATCC  AAGCC----TTATCC | SSMP | Ampelodesmeae+Stipeae |
| --- | --- | --- | --- | --- | --- | --- |
| 2 | Schedonorus arundinaceus Schedonorus arundinaceus[C] Schedonorus pratensis  Festuca altissima  Festuca ovina  Lolium multiflorum  Lolium perenne  Dactylis glomerata  Helictochloa hookeri  Deschampsia antarctica  Poa palustris  Phleum alpinum  Briza sp  Puccinellia nuttalliana  Ammophila breviligulata  Agrostis stolonifera  Hierochloe odorata  Anthoxanthum odoratum  Phalaris arundinacea  Torreyochloa  Avena sativa  Trisetum cernuum  **Aegilops cylindrica**  **Aegilops geniculata**  **Aegilops speltoides**  **Aegilops tauschii**  **Triticum aestivum**  **Triticum monococcum aegilopoides Triticum monococcum (modified) Triticum urartu (modified)**  **Hordeum jubatum**  **Hordeum vulgare**  **Hordeum vulgare spontaneum Secale cereale**  Bromus vulgaris  Brachypodium distachyon  Diarrhena obovata  Melica mutica  Melica subulata  Oryzopsis asperifolia Achnatherum hymenoides Ampelodesmos mauritanica Piptochaetium avenaceum  Phaenosperma globosum **Brachyelytrum aristosum**  Bambusa bambosa | 2275  IGS |  | TATTAACTAGAAATAATATAGATAA  TATTAACTAGAAATAATATAGATAA  TATTAACTATAAATAATATAGATAA  TATTAACTAGAAATAATATAGATAA  TATTAACTAGAAATAATATAGATAA  TATTAACTATAAATAATATAGATAA  TATTAACTATAAATAATATAGATAA  TATTAACTAGAAATAATATAGATAA  TATTAACTAGAAATAATATAGATAA  TATTAACTAGAAATAATATAGATAA  TATTAACTAGAAATAATATAGATAA  TATTAACTAGAAATAATATAGATAA  TATTAACTAGAAATAATATAGATAA  TATTAACTAGAAATAATATAGATAA  TATTAACTAGAAATAATATAGATAA  TATTAACTAGAAATAATATAGATAA  TATTAACTAGAAATAATATAGATAA  TATTAACTAGAAATAATATAGATAA  TATTAACTAGAAATAATATAGATAA  TATTAACTAGAAATAATATAGATAA  TATTAACTAGAAATAATATAGATAA  TATTAACTAGAAATAATATAGATAA  TATTAACTAG----AATATAGATAA  TATTAACTAG----AATATAGATAA  TATTAACTAG----AATATAGATAA  TATTAACTAG----AATATAGATAA  TATTAACTAG----AATATAGATAA  TATTAACTAG----AATATAGATAA  TATTAACTAG----AATATAGATAA  TATTAACTAG----AATATAGATAA  TATTAACTAG----AATATAGATAA  TATTAACTAG----AATATAGATAA  TATTAACTAG----AATATAGATAA  TATTAACTAG----AATATAGATAA  TATTAACTAGAAATAATATAGATAA  TATTAACTAGAAATAATATAGATAA  TATTAACTAGAAATAATATAGATAA  TATTAACTAGAAATAATATAGATAA  TATTAACTAGAAATAATATAGATAA  TATTAACTAGAAATAATATAGATAA  TATTAACTAGAAAAAATATAGATAA  TATTAACTAGAAATAATATAGATAA  TATTAACTAGAAAGAATATAGATAA  TATTAACTAGAAAGAATATAGATAA  TATTAACTAT----AAAATAGATAA  TATTAACTAGAAAGAAAATAGATAA |  | Homoplasy |
| 3 | Schedonorus arundinaceus Schedonorus arundinaceus[C] Schedonorus pratensis  Festuca altissima  Festuca ovina  Lolium multiflorum  Lolium perenne  Dactylis glomerata  Helictochloa hookeri  Deschampsia antarctica  Poa palustris  Phleum alpinum  Briza sp  Puccinellia nuttalliana  Ammophila breviligulata  Agrostis stolonifera  Hierochloe odorata  Anthoxanthum odoratum  Phalaris arundinacea  Torreyochloa  Avena sativa  Trisetum cernuum  Aegilops cylindrica  Aegilops geniculata  Aegilops speltoides  Aegilops tauschii  Triticum aestivum  Triticum monococcum aegilopoides Triticum monococcum (modified) Triticum urartu (modified)  Hordeum jubatum  Hordeum vulgare  Hordeum vulgare spontaneum Secale cereale  Bromus vulgaris  Brachypodium distachyon  Diarrhena obovata  Melica mutica  Melica subulata  Oryzopsis asperifolia Achnatherum hymenoides Ampelodesmos mauritanica Piptochaetium avenaceum  Phaenosperma globosum Brachyelytrum aristosum  Bambusa bambosa | 2839  trnK-UUU | trnK-UUU | TTATCAACC--------CTTTTCTG  TTATCAACC--------CTTTTCTG  TTATCAACC--------CTTTTCTG  TTATCAACC--------CTTTTCTG  TTATCAACC--------CTTTTCTG  TTATCAACC--------CTTTTCTG  TTATCAACC--------CTTTTCTG  TTATCAACC--------CTTTTCTG  TTATCAACC--------CCTTTCTG  TTATCAACC--------CTTTTCTG  TTCTCAACC--------TTTTTCTG  TTTTCAACC--------TTTTTCTG  TTATCAACCCTTTCTTTCTTTTCTG  TGATCAACC--------CTTTTCGG  TTATCTACC----CTTTCTTTTCTG  TTATCTACC----CTTTCTTTTCTG  TTATCTACC----CTTTCTTTTCTG  TTATATACC----CTTTCTTTTCTG  TTATCTACC----CTTTCTTTTCTG  TTATCTACC----CTTTCTTTTCTG  TTATCTAGC----CTTTCTTTTCTG  TTATCTACC----CTTTCTTTTCTG  TTATCAACC--------CTTTCCTG  TTATCAACC--------CTTTCCTG  TTATCAACC--------CTTTCCTG  TTATCAACC--------CTTTCCTG  TTATCAACC--------CTTTCCTG  TTATCAACC--------CTTTCCTG  TTATCAACC--------CTTTCCTG  TTATCAACC--------CTTTCCTG  TTATCAACC--------CTTTCCTG  TTATCAACC--------CTTTCCTG  TTATCAACC--------CTTTCCTG  TTATCAACC--------CTTTCTTG  TTATCAACC--------CTTTCCTG  TCATCGACC--------CTTTCCTG  TTATCGACC--------CTTTCCGG  CAATCGACC-------CCTTTCCAG  CAATCGACC-------CCTTTCCAG  TTATCGACC--------CTTTCCTC  TTATCGACC--------CTTTCCTG  TTATCGACC--------CTTTCCTG  TTATCGACC--------CTTTCCTG  TTATCGACC--------CTTTCCTG  TTATTGATC--------ATTTCTAG  TTATCGATC--------ATTTCTAG | SSMP | Poeae clade 1 |
| 4 | Schedonorus arundinaceus Schedonorus arundinaceus[C] Schedonorus pratensis  Festuca altissima  Festuca ovina  Lolium multiflorum  Lolium perenne  Dactylis glomerata  Helictochloa hookeri  Deschampsia antarctica  Poa palustris  Phleum alpinum  Briza sp  Puccinellia nuttalliana  Ammophila breviligulata  Agrostis stolonifera  Hierochloe odorata  Anthoxanthum odoratum  Phalaris arundinacea  Torreyochloa  Avena sativa  Trisetum cernuum  Aegilops cylindrica  Aegilops geniculata  Aegilops speltoides  Aegilops tauschii  Triticum aestivum  Triticum monococcum aegilopoides Triticum monococcum (modified) Triticum urartu (modified)  Hordeum jubatum  Hordeum vulgare  Hordeum vulgare spontaneum Secale cereale  Bromus vulgaris  Brachypodium distachyon  Diarrhena obovata  Melica mutica  Melica subulata  Oryzopsis asperifolia Achnatherum hymenoides Ampelodesmos mauritanica Piptochaetium avenaceum  Phaenosperma globosum Brachyelytrum aristosum  Bambusa bambosa | 4287  matK | matK | TATTACAACT---ACAAATTT  TATTACAACT---ACAAATTT  TATTACAACT---AAATATTT  TATTACAACT---AACAATTT  TATTACAACT---AACAATTT  TATTACAACT---AAATATTT  TATTACAACT---AAATATTT  TATTACAACT---AACAATTT  TATTACAACA---AAAAATTT  TATTACAACT---AACAATTT  TATTACAATT---AACAATTT  TATTATAACT---AACAATTT  TATTACAACT---AACAATTT  TATTAGAACT---AACAATTT  TATTACAACT---AACAATTT  TATTACAACT---AACAATTT  TATTACAACT---AAAAATTT  TATTACAACT---AACAATTT  TATTAGAACT---AACAATTT  TATTACAACT---AACAATTT  TATTACAACT---AATAATTT  TATTACAACT---AACAATTT  TATTACAACTAACAACTATTT  TATTACAACTAACAACTATTT  TATTACAACTAACAACTATTT  TATTACAACTAACAACTATTT  TATTACAACTAACAACTATTT  TATTACAACTAACAACTATTT  TATTACAACTAACAACTATTT  TATTACAACTAACAACTATTT  TATTACAACT---AACTATTT  TATTCCAACT---AACTATTT  TATTCCAACT---AACTATTT  TATTACAACTAACAACTATTT  TATTACAACT---AACTATTT  TATTACAACT---AACAATTT  TATTACAACT---AACAATTT  TATTACAACT---AACAATTT  TATTACAACT---AACAATTT  TATTACAACT---AAAAATTT  TATTACAACT---AACAATTT  TATTACAACT---AACAATTT  TATTACAACT---AAAAATTT  TATTACAACT---AACAATTT  TATTAGAACT---AACAATTT  TATTACAACT---AACAATTT | SSMP | Aegilops+Triticum+Secale |
| 5 | Schedonorus arundinaceus Schedonorus arundinaceus[C] Schedonorus pratensis  Festuca altissima  Festuca ovina  Lolium multiflorum  Lolium perenne  Dactylis glomerata  Helictochloa hookeri  Deschampsia antarctica  Poa palustris  Phleum alpinum  Briza sp  Puccinellia nuttalliana  Ammophila breviligulata  Agrostis stolonifera  Hierochloe odorata  Anthoxanthum odoratum  Phalaris arundinacea  Torreyochloa  Avena sativa  Trisetum cernuum  Aegilops cylindrica  Aegilops geniculata  Aegilops speltoides  Aegilops tauschii  Triticum aestivum  Triticum monococcum aegilopoides Triticum monococcum (modified) Triticum urartu (modified)  Hordeum jubatum  Hordeum vulgare  Hordeum vulgare spontaneum Secale cereale  Bromus vulgaris  Brachypodium distachyon  Diarrhena obovata  Melica mutica  Melica subulata  Oryzopsis asperifolia Achnatherum hymenoides Ampelodesmos mauritanica Piptochaetium avenaceum  Phaenosperma globosum Brachyelytrum aristosum  Bambusa bambosa | 5385  IGS |  | ACCATG-AAA----AACACTC  ACCATG-AAA----AACACTC  ACCATG-AAA----AACACTC  ACCATG-AAA----AACACTC  ACCATG-AAA----AACACTC  ACCATG-AAA----AACACTC  ACCATG-AAA----AACACTC  ACCATG-AAA----AACACTC  ACCATG-AAA----AACACTC  ACCATG-AAA----AACACTA  ACCATG-AAA----AACACTC  ACCATG-AAA----AACACTC  ACCATG-AAA----AACACTC  ACCATG-AAA----AACACTA  ACCATG-AAA----AACACTC  ACCATGAAAA----AACACTC  ACCATG-AAA----AACACTC  ACCATG-AAA----AAGACTC  ACCATG-AAA----AACACTC  ACCATG-AAA----AACACTC  ACCATG-AAA----AACACTC  ACCATG-AAA----AACACTC  ACCATG-AAA----AACACTC  ACCATG-AAA----AACACTC  ACCATG-AAA----AACACTC  ACCATG-AAA----AACACTC  ACCATG-AAA----AACACTC  ACCATG-AAA----AACACTC  ACCATG-AAA----AACACTC  ACCATG-AAA----AACACTC  ACCATG-AAA----AACACTC  ACCATG-AAA----AACACTC  ACCATG-AAA----AACACTC  ACCATG-AAA----AACACTC  ACCATG-AAA----AACACTC  ACCATG-AAA----AAGACTC  ACCATG-AAA----AACACTC  ACCATTAAAAACACAACACGC  ACCATTAAAAACACAACACGC  ACCATT-AAA----AACAATC  ACCATT-AAA----AACAATC  ACCATT-AAA----AACAATC  ACCATT-AAA----AACAATC  ACCATT-AAA----AACACTC  CCCATT-AAA----AACACTT  CCCATT-AAA----AACACTC |  | Melica |
| 6 | Schedonorus arundinaceus Schedonorus arundinaceus[C] Schedonorus pratensis  Festuca altissima  Festuca ovina  Lolium multiflorum  Lolium perenne  Dactylis glomerata  Helictochloa hookeri  Deschampsia antarctica  Poa palustris  Phleum alpinum  Briza sp  Puccinellia nuttalliana  Ammophila breviligulata  Agrostis stolonifera  Hierochloe odorata  Anthoxanthum odoratum  Phalaris arundinacea  Torreyochloa  Avena sativa  Trisetum cernuum  Aegilops cylindrica  Aegilops geniculata  Aegilops speltoides  Aegilops tauschii  Triticum aestivum  Triticum monococcum aegilopoides Triticum monococcum (modified) Triticum urartu (modified)  Hordeum jubatum  Hordeum vulgare  Hordeum vulgare spontaneum Secale cereale  Bromus vulgaris  Brachypodium distachyon  Diarrhena obovata  Melica mutica  Melica subulata  Oryzopsis asperifolia Achnatherum hymenoides Ampelodesmos mauritanica Piptochaetium avenaceum  Phaenosperma globosum Brachyelytrum aristosum  Bambusa bambosa | 5470  IGS |  | CGACCCCAACCAACCA  CGACCCCAACCAACCA  CGACCCCAACCAACCA  CGACCCC----AACCA  TAACCCC----AACCA  CGACCCCAACCAACCA  CGACCCCAACCAACCA  CGACCCC----AACCA  CGACCCC----GATCA  CGACCCC----GATCA  GGACCCC----GATTA  CGACCCC----GATTA  CGACTCT----AATCA  CGACCCC----GATTA  CGACTCC----AATCA  CGACTCC----AATCA  CGACTCC----AATCA  CAACTCC----AATCA  CGACTCC----AATCA  CGACTCC----AATCA  CGACTCC----AATCA  CGACTCC----AATCA  CGACCCT----AATCA  CGACCCT----AATCA  CGACCCT----AATCA  CGACCCT----AATCA  CGACCCT----AATCA  CGACCCT----AATCA  CGACCCT----AATCA  CGACCCT----AATCA  CGACCCT----AATCA  CGACCCT----AATCA  CGACCCT----AATCA  CGACCCT----AATCA  CGACCCT----AATCA  TGACCCC----AATCG  CGACCCC----AATCA  GGACCCC----AATCA  GGACCCC----AATCA  TGACCCC----AATCA  CGACCCC----AATCA  TGACCCC----AATCA  TGACCCC----AATCA  CGACCCC----GATTA  CGGCCCC----AATCA  CGGCTCC----AATCA | Ssmp | Homoplasy |
| 7 | Schedonorus arundinaceus Schedonorus arundinaceus[C] Schedonorus pratensis  Festuca altissima  Festuca ovina  Lolium multiflorum  Lolium perenne  Dactylis glomerata  Helictochloa hookeri  Deschampsia antarctica  Poa palustris  Phleum alpinum  Briza sp  Puccinellia nuttalliana  Ammophila breviligulata  Agrostis stolonifera  Hierochloe odorata  Anthoxanthum odoratum  Phalaris arundinacea  Torreyochloa  Avena sativa  Trisetum cernuum  Aegilops cylindrica  Aegilops geniculata  Aegilops speltoides  Aegilops tauschii  Triticum aestivum  Triticum monococcum aegilopoides Triticum monococcum (modified) Triticum urartu (modified)  Hordeum jubatum  Hordeum vulgare  Hordeum vulgare spontaneum Secale cereale  Bromus vulgaris  Brachypodium distachyon  Diarrhena obovata  Melica mutica  Melica subulata  Oryzopsis asperifolia Achnatherum hymenoides Ampelodesmos mauritanica Piptochaetium avenaceum  Phaenosperma globosum Brachyelytrum aristosum  Bambusa bambosa | 5622  IGS |  | AGGAAC-----ACCCTTATCAT  AGGAAC-----ACCCTTATCAT  AGGAAC-----ACCCTTATCAT  AGGAAC-----ACCCTTATCAT  AGGAAC-----ACCCTTATCAT  AGGAAC-----ACCCTTATCAT  AGGAAC-----ACCCTTATCAT  AGGAAC-----ACCCTTATCAT  AGGAGC-----ACCCTTATCAT  AGGAAC-----ACCCTTATCAT  AGGAAC-----ACCCTTATCAT  AGGAAC-----ACCCTTATCAT  AGGGAC-----ACCCTTACCAT  AGGAAC-----ACTCTTATCAT  AGGGAC-----ACCCTTACCAT  AGAGAC-----ACCCTTACCAT  AGGGAC-----ACCCTTACCAT  AGGGGC-----A-CCTTACCAT  AGGGAC-----ACCCTTATCAT  AGGGAC-----ACCCTTATCAT  AGAGAC-----ACCCTTATCAT  AGAGAC-----ACCCTTATCAT  AGGAAC-----ACCCTTATCAT  AGGAAC-----ACCCTTATCAT  AGGAAC-----ACCCTTATCAT  AGGAAC-----ACCCTTATCAT  AGGAAC-----ACCCTTATCAT  AGGAAC-----ACCCTTATCAT  AGGAAC-----ACCCTTATCAT  AGGAAC-----ACCCTTATCAT  AGGAAC-----ACCCTTATCAT  AGGAAC-----ACCCTTATCAT  AGGAAC-----ACCCTTATCAT  AGGAAC-----ACCCTTATCAT  AGGAAC-----ACCCCTATCAT  AGAAAC-----ACCCTTATCAT  AGGAAC-----ACCCTTATCAT  AGGAACAGAACACCCTTATCAT  AGGAACAGAACACCCTTATCAT  AGGAAA-----ACCCTTATCAT  AGGAAC-----ACCCTTATCGT  AGGAAC-----ACCCTTATCAT  AGGAAC-----ACCCTTATCAT  AGGAAC-----ACCCTTATCAT  AGGGAC-----AACCCTATCAT  AGGGAC-----AACCCTATCAT |  | Melica |
| 8 | Schedonorus arundinaceus Schedonorus arundinaceus[C]  Schedonorus pratensis  Festuca altissima  Festuca ovina  Lolium multiflorum  Lolium perenne  Dactylis glomerata  Helictochloa hookeri  Deschampsia antarctica  Poa palustris  Phleum alpinum  Briza sp  Puccinellia nuttalliana  Ammophila breviligulata  Agrostis stolonifera  Hierochloe odorata  Anthoxanthum odoratum  Phalaris arundinacea  Torreyochloa  Avena sativa  Trisetum cernuum  Aegilops cylindrica  Aegilops geniculata  Aegilops speltoides  Aegilops tauschii  Triticum aestivum  Triticum monococcum aegilopoides Triticum monococcum (modified) Triticum urartu (modified)  Hordeum jubatum  Hordeum vulgare  Hordeum vulgare spontaneum  Secale cereale  Bromus vulgaris  Brachypodium distachyon  Diarrhena obovata  Melica mutica  Melica subulata  Oryzopsis asperifolia Achnatherum hymenoides Ampelodesmos mauritanica Piptochaetium avenaceum  Phaenosperma globosum Brachyelytrum aristosum  Bambusa bambosa | 5678  IGS |  | AATATGCAGTAAGGTCTATTGAACAAATAGAATTTGTAGGAGGA-TAAA  AATATGCAGTAAGGTCTATTGAACAAATAGAATTTGTAGGAGGA-TAAA  AATATGCAGTAAGGTCTATTGAACAAATAGAATTTGTAGGAGGA-TAAA  AATATGCAGT-----------------------------GAGGA-TAAA  AATATGCAGT-----------------------------GAGGA-TAAA  AATATGCAGTAAGGTCTATTGAACAAATAGAATTTGTAGGAGGA-TAAA  AATATGCAGTAAGGTCTATTGAACAAATAGAATTTGTAGGAGGA-TAAA  AATACGCAGT-----------------------------GAGGA-TAAA  AATATGCAGT-----------------------------GAGGA-TAAA  AATATGCAGT-----------------------------GAGGA-TAAA  AATATGCAGT-----------------------------GAGGA-TAAA  AATATGCAGT-----------------------------GAGGA-TAAA  AATATGGAGT-----------------------------GAGGA-TAAA  AATATGCAGT-----------------------------GAGGA-TAAA  AATATGGAGT-----------------------------GAGGA-TAAA  AATATGGAGT-----------------------------GAGGA-TAAA  AATATGGAGT-----------------------------GAGGA-TAAA  AATATGGAGT-----------------------------GAGGA-TAAA  AATATGGAGT-----------------------------GAGGA-TAAA  AATATGGAGT-----------------------------GAGGA-TAAA  AATATGGAGT-----------------------------GAGGATTAAA  AATATGGAGT-----------------------------GAGGATTAAA  AATATGGAGT-----------------------------GAGGA-TAAA  AATATGGAGT-----------------------------GAGGA-TAAA  AATATGGAGT-----------------------------GAGGA-TAAA  AATATGGAGT-----------------------------GAGGA-TAAA  AATATGGAGT-----------------------------GAGGA-TAAA  AATATGGAGT-----------------------------GAGGA-TAAA  AATATGGAGT-----------------------------GAGGA-TAAA  AATATGGAGT-----------------------------GAGGA-TAAA  AATATGCAGT-----------------------------GAGGA-TAAA  AATATGGAGT-----------------------------GAGGA-TAAA  AATATGGAGT-----------------------------GAGGA-TAAA  AATATGGAGT-----------------------------GAGGA-TAAA  AATATGGAGT-----------------------------GAGGA-TAAA  AATATGGAGT-----------------------------GAGGA-TAAA  AATATGGAGT-----------------------------GAGGA-TAAA  AATATGGAGT-----------------------------GAGGA-TAAA  AATATGGAGT-----------------------------GAGGA-TAAA  AATATGGAGT-----------------------------GAGGA-TAAA  AATATGGAGT-----------------------------GAGGA-GAAA  AATATGGAGT-----------------------------GAGGA-TAAA  AATATGGAGT-----------------------------GAGGA-TAAA  AATATGGAGT-----------------------------GAGGA-TAAA  AATAAGGAGT-----------------------------GAGGA-TAAA  AATATGGAGT-----------------------------GAGGA-TAAA |  | Lolium+Schedonorus |
| 9 | Schedonorus arundinaceus Schedonorus arundinaceus[C] Schedonorus pratensis  Festuca altissima  Festuca ovina  Lolium multiflorum  Lolium perenne  Dactylis glomerata  Helictochloa hookeri  Deschampsia antarctica  Poa palustris  Phleum alpinum  Briza sp  Puccinellia nuttalliana  Ammophila breviligulata  Agrostis stolonifera  Hierochloe odorata  Anthoxanthum odoratum  Phalaris arundinacea  Torreyochloa  Avena sativa  Trisetum cernuum  Aegilops cylindrica  Aegilops geniculata  Aegilops speltoides  Aegilops tauschii  Triticum aestivum  Triticum monococcum aegilopoides Triticum monococcum (modified) Triticum urartu (modified)  Hordeum jubatum  Hordeum vulgare  Hordeum vulgare spontaneum Secale cereale  Bromus vulgaris  Brachypodium distachyon  Diarrhena obovata  Melica mutica  Melica subulata  Oryzopsis asperifolia Achnatherum hymenoides Ampelodesmos mauritanica Piptochaetium avenaceum  Phaenosperma globosum Brachyelytrum aristosum  Bambusa bambosa | 5836  IGS |  | TGGCACGACAT-----AAATCC  TGGCACGACAT-----AAATCC  TGGCACGACAT-----AAATCC  TGGCACGACAT-----AAATCC  TGGCACGACAT-----AAATCC  TGGCACGACAT-----AAATCC  TGGCACGACAT-----AAATCC  TGGCACGACAT-----AAATCC  TGGCACGACAT-----AAATCC  TGGCACGACAG-----AAATCC  TGGCACGACAT-----AAATCC  TGGCACGACAT-----AAATCC  TGGCACGACAT-----AAATCC  TGGCGCGACAT-----AAATCC  TGGCACGACAT-----AAATCC  TGGCACGACAT-----AAATCC  TGGCACGACAT-----AAATCC  TGGCACGACAT-----AAATCC  TGGCACGACAT-----AAATCC  TGGCACGACAT-----AAATCC  TGGCACGACAT-----AAATCC  TGGCACGACAT-----AAATCC  TGGCACGATATAATATAAATCC  TGGCACGATATAATATAAATCC  TAGCACGATATAATATAAATCC  TGGCACGATATAATAGAAATCC  TAGCACGATATAATATAAATCC  TGGCACGATATAATATAAATCC  TGGCACGATATAATATAAATCC  TGGCACGATATAATATAAATCC  TGGCACGATAT-----AAATCC  TGGCACGATAT-----AAATCC  TGGCACGATAT-----AAATCC  TGGCACGATATAATATAAATCC  TGGCACGATAT-----AAATCC  TGGCACGACAT-----AAATCC  TAGCACGACAT-----AAATCC  TGGCACGACAT-----AAATCC  TGGCACGACAT-----AAATCC  TGGCACGACAT-----AAATCC  TGGCACGACAT-----AAATCC  TGGCACGACAT-----AAATCC  TGGCACGACAT-----AAATCC  TGGCACGACAT-----AAATCC  TGGCACGACAT-----AAATCC  TGGCACGACAT-----AAATCC |  | Aegilops+Triticum+Secale |
| 10 | Schedonorus arundinaceus Schedonorus arundinaceus[C] Schedonorus pratensis  Festuca altissima  Festuca ovina  Lolium multiflorum  Lolium perenne  Dactylis glomerata  Helictochloa hookeri  Deschampsia antarctica  Poa palustris  Phleum alpinum  Briza sp  Puccinellia nuttalliana  Ammophila breviligulata  Agrostis stolonifera  Hierochloe odorata  Anthoxanthum odoratum  Phalaris arundinacea  Torreyochloa  Avena sativa  Trisetum cernuum  Aegilops cylindrica  Aegilops geniculata  Aegilops speltoides  Aegilops tauschii  Triticum aestivum  Triticum monococcum aegilopoides Triticum monococcum (modified) Triticum urartu (modified)  Hordeum jubatum  Hordeum vulgare  Hordeum vulgare spontaneum Secale cereale  Bromus vulgaris  Brachypodium distachyon  Diarrhena obovata  Melica mutica  Melica subulata  Oryzopsis asperifolia Achnatherum hymenoides Ampelodesmos mauritanica Piptochaetium avenaceum  Phaenosperma globosum Brachyelytrum aristosum  Bambusa bambosa | 5852  IGS |  | AAATCC------AAATAGG  AAATCC------AAATAGG  AAATCC------AAATAGG  AAATCC------AAATAGG  AAATCC------AAATAGG  AAATCC------AAATAGG  AAATCC------AAATAGG  AAATCC------AAATAGG  AAATCC------AAATAGG  AAATCC------AAATAGT  AAATCC------AAATAGG  AAATCC------AAATAGG  AAATCC------AAATAGG  AAATCC------AAATAGG  AAATCC------AAATAGG  AAATCC------AAATAGG  AAATCC------AAATAGG  AAATCC------AAATAGG  AAATCC------AAATAGG  AAATCC------AAATAGG  AAATCC------AAATAGG  AAATCC------AAATAGG  AAATCC------AAATAGG  AAATCC------AAATAGG  AAATCC------AAATAGG  AAATCC------AAATAGG  AAATCC------AAATAGG  AAATCC------AAATAGG  AAATCC------AAATAGG  AAATCC------AAATAGG  AAATCC------AAATAGG  AAATCC------AAATAGG  AAATCC------AAATAGG  AAATCC------AAATAGG  AAATCC------AAATAGG  AAATCC------AAATAGG  AAATCC------AAATAGG  AAATCC------AAAAAGG  AAATCC------AAAAAGG  AAATCC------AAAAAGG  AAATCC------AAATAGG  AAATCC------AAAAAGG  AAATCC------AAATAGG  AAATCC------AAATAGG  AAATCCAGTCAAAAATAGG  AAATCCAGTCAAAAATAGG |  | Homoplasy |
| 11 | Schedonorus arundinaceus Schedonorus arundinaceus[C] Schedonorus pratensis  Festuca altissima  Festuca ovina  Lolium multiflorum  Lolium perenne  Dactylis glomerata  Helictochloa hookeri  Deschampsia antarctica  Poa palustris  Phleum alpinum  Briza sp  Puccinellia nuttalliana  Ammophila breviligulata  Agrostis stolonifera  Hierochloe odorata  Anthoxanthum odoratum  Phalaris arundinacea  Torreyochloa  Avena sativa  Trisetum cernuum  Aegilops cylindrica  Aegilops geniculata  Aegilops speltoides  Aegilops tauschii  Triticum aestivum  Triticum monococcum aegilopoides Triticum monococcum (modified) Triticum urartu (modified)  Hordeum jubatum  Hordeum vulgare  Hordeum vulgare spontaneum Secale cereale  Bromus vulgaris  Brachypodium distachyon  Diarrhena obovata  Melica mutica  Melica subulata  Oryzopsis asperifolia Achnatherum hymenoides Ampelodesmos mauritanica Piptochaetium avenaceum  Phaenosperma globosum Brachyelytrum aristosum  Bambusa bambosa | 5934  IGS |  | TAGTGATC-TT-----CTTCTAC  TAGTGATC-TT-----CTTCTAC  TAGTGATC-TT-----CTTCTAC  TAGTGATC-TT-----CTCCTAC  TGGTGATC-TT-----CTCCTAC  TAGTGATC-TT-----CTTCTAC  TAGTGATC-TT-----CTTCTAC  TAGTGATC-TTCTCCACTCCTAC  TAGCGACC-CT-----CTCCTAC  TAGTGATC-TT-----CTCCTAC  TAGTGATC-TT-----CTCCTAC  TAGTGATC-TT-----CTCCTAC  TAGTGATT-TT-----ATCCTAC  TAGTGATC-TT-----CTACTAC  TAGTGATC-TT-----CTCCTAC  TAGTGATC-TT-----CTCCTAC  TAGTGATC-TT-----CTCCTAC  TAGTGATT-TT-----CTCCTAC  TAGTGATC-TT-----CTCCTAC  TAGTGATC-TT-----CTCCTAC  TGGTGATC-TT-----CTCCTAC  TAGTGATC-TT-----CTCCTAC  TAATAATC-TTCTCCTATCCTAC  TAATAATC-TTCTCCTATCCTAC  TAATAATC-TTCTCCTATCCTAC  TAATAATC-TTCTCCTATCCTAC  TAATAATC-TTCTCCTATCCTAC  TAATAATC-TTCTCCTATCCTAC  TAATAATC-TTCTCCTATCCTAC  TAATAATC-TTCTCCTATCCTAT  TAATAATC-TTCTTCTATCCTAC  TAATAATC-TTCTCCTATCCTAC  TAATAATC-TTCTCCTATCCTAC  TAATAATC-TTCTCCTATCCTAC  TAATAATC-TTCTCCTATCCTAC  TAGTAATC-TT-----CTCCTAC  TAGTGATC-TT-----CTCCTAC  TAGCGATT-TT-----CTACTAC  TAGCGATT-TT-----CTACTAC  TAGTGATC-TT-----CTCCTAC  TAGTGATC-TT-----CTCCTAC  TAGTGATC-TT-----CTCCTAC  TAGTGATC-TT-----CTCCTAC  TAGTGATC-TT-----CTCCTAC  TCGCGACC-CT-----TTCCTAC  TAGTGATCCCT-----CTCCTAC |  | Homoplasy |
| 12 | Schedonorus arundinaceus Schedonorus arundinaceus[C] Schedonorus pratensis  Festuca altissima  Festuca ovina  Lolium multiflorum  Lolium perenne  Dactylis glomerata  Helictochloa hookeri  Deschampsia antarctica  Poa palustris  Phleum alpinum  Briza sp  Puccinellia nuttalliana  Ammophila breviligulata  Agrostis stolonifera  Hierochloe odorata  Anthoxanthum odoratum  Phalaris arundinacea  Torreyochloa  Avena sativa  Trisetum cernuum  Aegilops cylindrica  Aegilops geniculata  Aegilops speltoides  Aegilops tauschii  Triticum aestivum  Triticum monococcum aegilopoides Triticum monococcum (modified) Triticum urartu (modified)  Hordeum jubatum  Hordeum vulgare  Hordeum vulgare spontaneum Secale cereale  Bromus vulgaris  Brachypodium distachyon  Diarrhena obovata  Melica mutica  Melica subulata  Oryzopsis asperifolia Achnatherum hymenoides Ampelodesmos mauritanica Piptochaetium avenaceum  Phaenosperma globosum Brachyelytrum aristosum  Bambusa bambosa | 6288  Rsp16 intron 1 | Rps16 intron | AATTTCTGTCTATAGTCTATA  AATTTCTGTCTATAGTCTATA  AATTTCTGTCTATAGTCTATA  AATTTCT-------GTCTATA  AATTTCT-------GTCTATA  AATTTCTGTCTATAGTCTATA  AATTTCTGTCTATAGTCTATA  AATTTCT-------GTCTATA  AATTTCTGTCTATAGTCTATA  AATTTCT-------GTCTATA  CATTTCT-------GTCTATA  AATTTCT-------GTCTATA  AATTTCT-------GTCTATA  AATTTCT-------GTCTATA  AATTTCT-------GTCTATA  AATTTCT-------GTCTATA  AATTTCT-------GTCTATA  AATTTCT-------GTCTATA  ACTTTCC-------GTCTATA  AATTTCT-------GTCTATA  AATTTCT-------GTCTATA  AATTTCT-------GTCTATA  AATTTCT-------GTCGATA  AATTTCT-------GTCGATA  AATTTCT-------GTCGATA  AATTTCT-------GTCGATA  AATTTCT-------GTCGATA  AATTTCT-------GTCGATA  AATTTCT-------GTCGATA  AATTTCT-------GTCGATA  AATTTCT-------GTCGATA  AATTTCT-------GTCGATA  AATTTCT-------GTCGATA  AATTTCT-------GTCAATA  AATTTCT-------GTCGATA  AATTTCT-------TTCTATA  AATTTCT-------GTCTATA  AATTTCT-------GTGTATA  AATTTCT-------GTGTATA  AATTTCT-------GTCTATA  AATTTCT-------GTCTATA  AATTTCT-------GTCTATA  AATTTCT-------GTCTATA  AATTTCT-------GTCTATA  CATTTCT-------GTCTATA  AATTTCT-------GTCTATA | SSMP | Homoplasy |
| 13 | Schedonorus arundinaceus Schedonorus arundinaceus[C] Schedonorus pratensis  Festuca altissima  Festuca ovina  Lolium multiflorum  Lolium perenne  Dactylis glomerata  Helictochloa hookeri  Deschampsia antarctica  Poa palustris  Phleum alpinum  Briza sp  Puccinellia nuttalliana  Ammophila breviligulata  Agrostis stolonifera  Hierochloe odorata  Anthoxanthum odoratum  Phalaris arundinacea  Torreyochloa  Avena sativa  Trisetum cernuum  Aegilops cylindrica  Aegilops geniculata  Aegilops speltoides  Aegilops tauschii  Triticum aestivum  Triticum monococcum aegilopoides Triticum monococcum (modified) Triticum urartu (modified)  Hordeum jubatum  Hordeum vulgare  Hordeum vulgare spontaneum Secale cereale  Bromus vulgaris  Brachypodium distachyon  Diarrhena obovata  Melica mutica  Melica subulata  Oryzopsis asperifolia Achnatherum hymenoides Ampelodesmos mauritanica Piptochaetium avenaceum  Phaenosperma globosum Brachyelytrum aristosum  Bambusa bambosa | 7042  Rps16 intron 1 | Rps16 intron | AAATTT------GGTTCGGG  AAATTT------GGTTCGGG  AAATTT------GGTTCGGG  AAATTT------GGTTCGGG  AAATTT------GGCTCGGG  AAATTT------GGTTCGGG  AAATTT------GGTTCGGG  AAATTG------GGTTCGGA  AAATTG------GGTTCGGG  AAATTT------GGTTCGGG  AAATTT------GGTTCGGG  AAATTT------GGTTCGGG  AAATTTGGTTCGGGTTCGGG  AAGTTT------GGTTCGGG  AAATTTGGTCCGGGTTCGGG  AAATTTGGTTCGGGTTCGGG  AAATTT------GGTTCGGG  AAATTT------GGTTCGGG  AAATTT------GGTTCGGG  AAATTT------GGTTCGGG  AAATTA------GGTTCGGG  AAATTT------GGTTCGGG  AAATTT------GGTTCGGA  AAATTT------GGTTCGGA  AAATTT------GGTTCGGA  AAATTT------GGTTCGGA  AAATTT------GGTTCGGA  AAATTT------GGTTCGGA  AAATTT------GGTTCGGA  AAATTT------GGTTCGGA  AAATTT------GGTTCGGA  AAATTT------GGTTCGGA  AAATTT------GGTTCGGA  AAATTT------GGTTCGGA  AAATTT------GGGTCGGA  AAATTT------GGTTCGGA  AAATTC------GGTTCGGG  AAATTT------GGTTCAGG  AAATTT------TGTTCAGG  AAATTC------GGTTCGGG  AAATTC------GGTTCGGG  AAATTC------GGTTCGGG  AAATTC------GGTTCGGG  AAATTC------GGTTGGGG  AAATTA------GGTTCGGG  AAATTT------GGTTCGGG | SSMP | Agrostidinae+Brizinae |
| 14 | Schedonorus arundinaceus Schedonorus arundinaceus[C] Schedonorus pratensis  Festuca altissima  Festuca ovina  Lolium multiflorum  Lolium perenne  Dactylis glomerata  Helictochloa hookeri  Deschampsia antarctica  Poa palustris  Phleum alpinum  Briza sp  Puccinellia nuttalliana  Ammophila breviligulata  Agrostis stolonifera  Hierochloe odorata  Anthoxanthum odoratum  Phalaris arundinacea  Torreyochloa  Avena sativa  Trisetum cernuum  Aegilops cylindrica  Aegilops geniculata  Aegilops speltoides  Aegilops tauschii  Triticum aestivum  Triticum monococcum aegilopoides Triticum monococcum (modified) Triticum urartu (modified)  Hordeum jubatum  Hordeum vulgare  Hordeum vulgare spontaneum Secale cereale  Bromus vulgaris  Brachypodium distachyon  Diarrhena obovata  Melica mutica  Melica subulata  Oryzopsis asperifolia Achnatherum hymenoides Ampelodesmos mauritanica Piptochaetium avenaceum  Phaenosperma globosum Brachyelytrum aristosum  Bambusa bambosa | 7272  IGS |  | TCATTG-----GTTTAGTT  TCATTG-----GTTTAGTT  TCATTG-----GTTTAGTT  TCATTG-----GTTTAGTT  TCATTG-----GTTTAGTT  TCATTG-----GTTTAGTT  TCATTG-----GTTTAGTT  TCATTG-----GTTTAGTT  TCATTG-----GTTTAGTT  TCATTG-----GTTTAGTT  TCATTG-----GTTTTGTT  TCATTG-----GTTTAGTT  TCATTG-----GTTTAGTT  TCATTG-----GTTTAGTT  TCATTG-----GTTTAGTT  TCATTG-----GTTTAGTT  TCATTG-----GTTTCGTT  TCATTG-----GTTTAGTT  TCATTG-----GTTTAGTT  TCATTG-----GTTTAGTT  TCATTG-----GTTTAGTT  TCATTG-----GTTTAGTT  TCATTG-----GTTTAGTT  TCATTG-----GTTTAGTT  TCATTG-----GTTTAGTT  TCATTG-----GTTTAGTT  TCATTG-----GTTTAGTT  TCATTG-----GTTTAGTT  TCATTG-----GTTTAGTT  TCATTG-----GTTTAGTT  TCATTG-----GTTTAGTT  TCATTG-----GTTTTGTT  TCATTG-----GTTTTGTT  TCATTG-----GTTTAGTT  TCATTG-----GTTTAGTT  TCATTG-----GTTTAGTT  TCATTG-----GTTTTGTT  TCATTG-----GTTTAGTT  TCATTG-----GTTTAGTT  TCATTGGTTTAGTTTAGTT  TCATTG-----GTTTAG-T  TCATTGGTTTAGTTTAGTT  TCATTG-----GTTTAGTT  TCATTGGTTTAGTTTAGTT  TCATTGGTTTA---TAGTT  TCATTGGTTTAGTTTAGTT | ssmp | Homoplasy |
| 15 | Schedonorus arundinaceus Schedonorus arundinaceus[C] Schedonorus pratensis  Festuca altissima  Festuca ovina  Lolium multiflorum  Lolium perenne  Dactylis glomerata  Helictochloa hookeri  Deschampsia antarctica  Poa palustris  Phleum alpinum  Briza sp  Puccinellia nuttalliana  Ammophila breviligulata  Agrostis stolonifera  Hierochloe odorata  Anthoxanthum odoratum  Phalaris arundinacea  Torreyochloa  Avena sativa  Trisetum cernuum  Aegilops cylindrica  Aegilops geniculata  Aegilops speltoides  Aegilops tauschii  Triticum aestivum  Triticum monococcum aegilopoides Triticum monococcum (modified) Triticum urartu (modified)  Hordeum jubatum  Hordeum vulgare  Hordeum vulgare spontaneum Secale cereale  Bromus vulgaris  Brachypodium distachyon  Diarrhena obovata  Melica mutica  Melica subulata  Oryzopsis asperifolia Achnatherum hymenoides Ampelodesmos mauritanica Piptochaetium avenaceum  Phaenosperma globosum Brachyelytrum aristosum  Bambusa bambosa | 7302  IGS |  | AATTC------AAAC  AATTC------AAAC  AATTC------AAAC  AATTC------AAAC  AATTC------AAAC  AATTC------AAAC  AATTC------AAAC  AATTC------AAAC  AATTC------AAAC  AATTC------AAAC  AATTC------AAAC  AATTC------AAAC  AATTC------AAAC  AATTC------AAAC  AATTC------AAAC  AATTC------AAAC  AATTT------AAAC  AATGT------AAAC  AATTA------AAAC  AATTC------AAAC  AATCC------AAAC  AATTC------AAAC  AATTA------AAAC  AATTA------AAAC  AATTA------AAAC  AATTA------AAAC  AATTA------AAAC  AATTA------AAAC  AATTA------AAAC  AATTA------AAAC  AATTAAAATTTAAAC  AATTAAAATTAAAAC  AATTAAAATTAAAAC  AATTA------AAAC  AATTA------AAAC  AATTC------AAAC  AATTC------AAAC  AATTC------AAAC  AATTC------AAAC  AATTA------AAAC  AATTA------AAAT  AATTA------AAAC  AATTA------AAAC  AATTC------AAAC  AATTA------AAAC  AATTC------AAAC |  | Hordeum |
| 16 | Schedonorus arundinaceus Schedonorus arundinaceus[C] Schedonorus pratensis  Festuca altissima  Festuca ovina  Lolium multiflorum  Lolium perenne  Dactylis glomerata  Helictochloa hookeri  Deschampsia antarctica  Poa palustris  Phleum alpinum  Briza sp  Puccinellia nuttalliana  Ammophila breviligulata  Agrostis stolonifera  Hierochloe odorata  Anthoxanthum odoratum  Phalaris arundinacea  Torreyochloa  Avena sativa  Trisetum cernuum  Aegilops cylindrica  Aegilops geniculata  Aegilops speltoides  Aegilops tauschii  Triticum aestivum  Triticum monococcum aegilopoides Triticum monococcum (modified) Triticum urartu (modified)  Hordeum jubatum  Hordeum vulgare  Hordeum vulgare spontaneum Secale cereale  Bromus vulgaris  Brachypodium distachyon  Diarrhena obovata  Melica mutica  Melica subulata  Oryzopsis asperifolia Achnatherum hymenoides Ampelodesmos mauritanica Piptochaetium avenaceum  Phaenosperma globosum Brachyelytrum aristosum  Bambusa bambosa | 7628  IGS |  | GGATCCACTCTTCCCCA  GGATCCACTCTTCCCCA  GGATCCACTCTTCCCCA  GGATCAACTCTTCCCCA  GGATCAACTCTTCTCCA  GGATCCACTCTTCCCCA  GGATCCACTCTTCCCCA  GGATCCACTCTTCCCCA  GGATCCACTCTTTCCCA  GGATCAACTTTTCCTCA  GGATCAACTCTTCCCCA  GGATCAACTCTTCCCCA  GGATCGACTCTTCCCCA  GGATCAACTCTTCCCCA  GGATCGACTCTTCCCCA  GGATCGACTCTTCCCCA  GGATCGATTCTCCCCCA  GGATCAACTCTCCCCCA  GG---------CCCCAA  GG----------CCCCA  GGATCAACTCTTCCCCA  GGATCAACTCTTACCCA  GGATTGACTCTTCCTCA  GGATTGACTCTTCCTCA  GGATTGACTCTTCCTCA  GGATTGACTCTTCCTCA  GGATTGACTCTTCCTCA  GGATTGACTCTTCCTCA  GGATTGACTCTTCCTCA  GGATTGACTCTTCCTCA  GGATTGACTCTTCCTCA  GGATTGACTCTTCCTCA  GGATTGACTCTTCCTCA  GGATTGACTCTTCCTCA  GGATTTACTCTTCCTCA  GGAGGAACTCTTACGCA  GGATCGACTCTTCCCCA  GGATCGACTCTTCCCCA  GGATCGACTCTTCCCCA  GGATCGACTCTTCCCCA  GGATCGACTCTTTACCA  GGATCGACTCTTCCCCA  GGATCGACTCTTCCCCA  GGATCGACTCTTCCCCA  GGATCAATTCTTCCCCA  GGATCGATTCTTCTCCA |  | Phalaridinae+Torreyochloinae |
| 17 | Schedonorus arundinaceus Schedonorus arundinaceus[C] Schedonorus pratensis  Festuca altissima  Festuca ovina  Lolium multiflorum  Lolium perenne  Dactylis glomerata  Helictochloa hookeri  Deschampsia antarctica  Poa palustris  Phleum alpinum  Briza sp  Puccinellia nuttalliana  Ammophila breviligulata  Agrostis stolonifera  Hierochloe odorata  Anthoxanthum odoratum  Phalaris arundinacea  Torreyochloa  Avena sativa  Trisetum cernuum  Aegilops cylindrica  Aegilops geniculata  Aegilops speltoides  Aegilops tauschii  Triticum aestivum  Triticum monococcum aegilopoides Triticum monococcum (modified) Triticum urartu (modified)  Hordeum jubatum  Hordeum vulgare  Hordeum vulgare spontaneum Secale cereale  Bromus vulgaris  Brachypodium distachyon  Diarrhena obovata  Melica mutica  Melica subulata  Oryzopsis asperifolia Achnatherum hymenoides Ampelodesmos mauritanica Piptochaetium avenaceum  Phaenosperma globosum Brachyelytrum aristosum  Bambusa bambosa | 8851  IGS |  | GCGGAGCA----------------------CTATCAGAAAC  GCGGAGCA----------------------CTATCAGAAAC  GCGGAGCA----------------------CTATCAGAAAC  GCGGAGTA----------------------CTATCAGAAAC  GCGGGGTA----------------------CAATCAGAAAC  GCGGAGCA----------------------CTATCAGAAAC  GCGGAGCA----------------------CTATCAGAAAC  GCGAAGTA----------------------CTATCAGAAAC  GCGGAGTA----------------------CAATCAGAAAC  GCGTAGTA----------------------CTATCAGAAAC  GCGGAGTA----------------------CTATCAGAAAC  GCGGAGTA----------------------CTATCAGAAAC  GTGGAGTA----------------------CTATCCGAAAC  GCGGAGTA----------------------CTATCAGAAAC  GCGGAGTA----------------------CTATCCAAAAC  GCGGAGTA----------------------CTATCCAAAAC  GCGGAGCA----------------------CTATCCGAAAC  GCGGAGTA----------------------CTATCCGAAAC  GCGGAGTA----------------------CTATCCGAAAC  GCGGAGTA----------------------CTATCCGAAAC  GTAGAGTACTATA-----------------CTATCCGAAAC  GTAGAGTA----------------------TTATCCAAAAC  ACGGAATACTATATGATTTCACCGTTTGTTTCATCAGAAAC  ACGGAATACTATATGATTTCACCGTTTGTTTCATCAGAAAC  ACGGAATACTATATGATTTCACCGTTTGTTTCATCAGAAAC  ACGGAATACTATATGATTTCACCGTTTGTTTCATCAGAAAC  ACGGAATACTATATGATTTCACCGTTTGTTTCATCAGAAAC  ACGGAATACTATATGATTTCACCGTTTGTTTCATCAGAAAC  ACGGAATACTATATGATTTCACCGTTTGTTTCATCAGAAAC  ACGGAATACTATATGATTTCACCGTTTGTTTCATCAGAAAC  TTGGAGTACTATATGATTTCACCGTTTGTTTCATCAGAAAC  ACGGAGTACTATATGATTTCACCGTTTGTTTCATCAGAAAC  ACGGAGTACTATATGATTTCACCGTTTGTTTCATCAGAAAC  ACGGAATACTATATGATTTCACCGTTTGTTTCATCAGAAAC  ACGGAGTACTGTATGATTTCATCGTTTGTTTCATCAGAAAC  GCGGAGTACAATATGATTTCATCTTTTGTTTCATCAGAAAC  GCGGAGTACAATATGATTTCCTCTTTTGTTTCATCAGAAAC  ACGGAGTACAATATGATTTCATCTTTTGTTTCATCAGAAAT  ACGGAGTACAATATGATTTCATCTTTTGTTTCATCAGAAAT  GCGGAGTACAATATGATTTCATCTTTTGTTTCATCAGAAAC  GCGGAGTACAATATGATTTCATCTTTTGTTTCATCAGAAAC  GCGGAGTACAATATGATTTCATCTTTTGTTTCATCAGAAAC  GCGGAGTACAATATGATTTCATCTTTTGTTTCATCAGAAAC  GCGGAGTACAATATGATTTCATCTTTTGTTTCATCAGAAAC  GCGGAGTACAATACGATTTGATCTTTCATTTCATCAGAAAA  GCGGGGTACAATACGATTTCATCTTTCGTTTCATCAGAAA- |  | Poeae |
| 18 | Schedonorus arundinaceus Schedonorus arundinaceus[C] Schedonorus pratensis  Festuca altissima  Festuca ovina  Lolium multiflorum  Lolium perenne  Dactylis glomerata  Helictochloa hookeri  Deschampsia antarctica  Poa palustris  Phleum alpinum  Briza sp  Puccinellia nuttalliana  Ammophila breviligulata  Agrostis stolonifera  Hierochloe odorata  Anthoxanthum odoratum  Phalaris arundinacea  Torreyochloa  Avena sativa  Trisetum cernuum  Aegilops cylindrica  Aegilops geniculata  Aegilops speltoides  Aegilops tauschii  Triticum aestivum  Triticum monococcum aegilopoides Triticum monococcum (modified) Triticum urartu (modified)  Hordeum jubatum  Hordeum vulgare  Hordeum vulgare spontaneum Secale cereale  Bromus vulgaris  Brachypodium distachyon  Diarrhena obovata  Melica mutica  Melica subulata  Oryzopsis asperifolia Achnatherum hymenoides Ampelodesmos mauritanica Piptochaetium avenaceum  Phaenosperma globosum Brachyelytrum aristosum  Bambusa bambosa | 9263  IGS |  | TGCCTTCTCCTT-TTTCCCTT-AGA  TGCCTTCTCCTT-TTTCCCTT-AGA  TGCCTTCTCCTT-TTTCCCTT-AGA  TGCCTTCCCCTT-TTTCCCTT-AGA  TGCCTTCTCCTT-TTTCCCTT-AGA  TGCCTTCTCCTT-TTTCCCTT-AGA  TGCCTTCTCCTT-TTTCCCTT-AGA  TGCCTTTTCCTT-TTTCCCTT-AGA  TGCC-------T-TTTCCCTT-AGA  TGCCTTTTCGTT-TTTCCCTT-AGA  TGCCTTTTCCCT-TTTCCCTT-AGA  CGCCTTTTCCTT-TTTCCCTT-ATA  CTCCTTTTCCTT-TTTCCCTT-AGA  TGCCTTTTCCTT-TTTCCCTT-AGA  TCCC-------T-TTTCCCTT-AGA  TACC-------T-TTTCCCTT-AGA  TGCCTTTTCCTT-TTTCCCTT-AGA  TGCCTTTTCCTT-TTTCCCTT-AGA  CGTCTTTTCCTT-TTTCCCTT-AGA  CGCCTTTTCCTT-TTTCCCTT-AGA  TGCCTTTTCCTTATTTCCCTT-AGA  TGCCTTTTCCTTATTTCCCTT-AGA  TGCCTTTTCCTTCTTTCCCTTAAAA  TGCCTTTTCCTTCTTTCCCTTAAAA  TGCCTTTTCCTTCTTTCCCTTAAAA  TGCCTTTTCCTTCTTTCCCTTAAAA  TGCCTTTTCCTTCTTTCCCTT-AAA  CGCCTTTTCCTTCTTTCCCTT-AAA  CGCCTTTTCCTTCTTTCCCTT-AAA  TGCCTTTTCCTTCTTTCCCTT-AAA  TGCCTTTTCCTTCTTTCCCTT-AAA  TGCCTTTTCCTTCTTTCCCTT-AAA  TGCCTTTTCCTTCTTTCCCTT-AAA  TGCCTTTTCCTTCTTTCCCTT-AAA  TGCCTTTTCCTTCTTTCCCTT-AAA  TGCCTTTTCCTT-TTTTCCTT-AGA  TGCCTTTTCCTT-TTTCCCTT-AGA  TGCCTTTTCCTT-TTTCCCTT-AGA  TGCCTTTTCCTT-TTTCCCTT-AGA  TGCCTTTTCCTT-TTTCCCTT-AGA  TGCCTTTTCTTT-TTTCCCTT-AGA  TGCCTTTTCCTT-TTTCCCTT-AGA  TGCCTTTTCCTT-TTTCCCTT-AGA  TGCCTTTTCCTT-TTTCCCTT-AGA  CGCCT--------TTTCCCTT-AGA  CGCCTTTTCCTT-TTTCCCTT-AGA |  | Homoplasy |
| 19 | Schedonorus arundinaceus Schedonorus arundinaceus[C] Schedonorus pratensis  Festuca altissima  Festuca ovina  Lolium multiflorum  Lolium perenne  Dactylis glomerata  Helictochloa hookeri  Deschampsia antarctica  Poa palustris  Phleum alpinum  Briza sp  Puccinellia nuttalliana  Ammophila breviligulata  Agrostis stolonifera  Hierochloe odorata  Anthoxanthum odoratum  Phalaris arundinacea  Torreyochloa  Avena sativa  Trisetum cernuum  Aegilops cylindrica  Aegilops geniculata  Aegilops speltoides  Aegilops tauschii  Triticum aestivum  Triticum monococcum aegilopoides Triticum monococcum (modified) Triticum urartu (modified)  Hordeum jubatum  Hordeum vulgare  Hordeum vulgare spontaneum Secale cereale  Bromus vulgaris  Brachypodium distachyon  Diarrhena obovata  Melica mutica  Melica subulata  Oryzopsis asperifolia Achnatherum hymenoides Ampelodesmos mauritanica Piptochaetium avenaceum  Phaenosperma globosum Brachyelytrum aristosum  Bambusa bambosa | 9395  PsbK | psbK | TTATCCG---ACTAGTTT  TTATCCG---ACTAGTTT  TTATCCG---ACTAGATT  TTATCCG---ACTAGTTT  TTATCCG---ACTAGTTT  TTATCCG---ACTAGTTT  TTATCCG---ACTAGTTT  TTATCCG---ACTAGTTT  TTATCCG---ACTAGTTT  TTATCCG---ACTAGTTT  TTATCCG---ACTAGTTT  TTATCCG---ACTAGTTT  TTATCCGACTACTAGTTT  TTATCCG---ACTAGTTT  TTATCCGACTACTAGTTT  TTATCCGACTACTAGTTT  TTATCCGACTACTAGTTT  TTATCCGACTACTAGTTT  TTATCCGACTACTAGTTT  TTATCCGACTACTAGTTT  TTATCCGACTACTAGTTT  TTATCCGACTACTAGTTT  TTATCCT---ACTAGTTT  TTATCCT---ACTAGTTT  TTATCCT---ACTAGTTT  TTATCCT---ACTAGTTT  TTATCCT---ACTAGTTT  TTATCCT---ACTAGTTT  TTATCCT---ACTAGTTT  TTATCCT---ACTAGTTT  TTATCCT---ACTAGTTT  TTATCCT---ACTAGTTT  TTATCCT---ACTAGTTT  TTATCCT---ACTAGTTT  TTATCCT---ACTAGTTT  TTATCCG---ACTAGTTT  TTATCCG---ACTAGTTT  TTATCCA---ACTAGTTT  TTATCCA---ACTAGCTT  TTATCCG---ACTAGTTT  TTATCCG---ACTTGTTT  TTATCCG---ACTAGTTT  TTATCCG---ACTAGTTT  TTATCCG---ACTAGTTT  TTATCCG---ACTAGTTT  TTATCCG---ACTAGTTT | Ssmp | Poeae clade 1 |
| 20 | Schedonorus arundinaceus Schedonorus arundinaceus[C] Schedonorus pratensis  Festuca altissima  Festuca ovina  Lolium multiflorum  Lolium perenne  Dactylis glomerata  Helictochloa hookeri  Deschampsia antarctica  Poa palustris  Phleum alpinum  Briza sp  Puccinellia nuttalliana  Ammophila breviligulata  Agrostis stolonifera  Hierochloe odorata  Anthoxanthum odoratum  Phalaris arundinacea  Torreyochloa  Avena sativa  Trisetum cernuum  Aegilops cylindrica  Aegilops geniculata  Aegilops speltoides  Aegilops tauschii  Triticum aestivum  Triticum monococcum aegilopoides Triticum monococcum (modified) Triticum urartu (modified)  Hordeum jubatum  Hordeum vulgare  Hordeum vulgare spontaneum Secale cereale  Bromus vulgaris  Brachypodium distachyon  Diarrhena obovata  Melica mutica  Melica subulata  Oryzopsis asperifolia Achnatherum hymenoides Ampelodesmos mauritanica Piptochaetium avenaceum  Phaenosperma globosum Brachyelytrum aristosum  Bambusa bambosa | 10215  IGS |  | TTCTTACAAATTGGATTT  TTCTTACAAATTGGATTT  TTCTTACAAATTGGATTT  TTCTTACAAATTGGATTT  TTCTTACAAATTGGATTT  TTCTTACAAATTGGATTT  TTCTTACAAATTGGATTT  TTCTTACAAATTGGATTT  TTCTTACAAATTGGATTT  TTCTTACAAATTGGATTT  TTCTTACAAATTGGATTT  TTCTTACAAATTGGATTT  TTCTTACAAATTGGATTT  TTCTTACAAATTGGATTT  TTCTTACAAATTGGATTT  TTCTTACAAATTGGATTT  TTCTTACAAATTGGATTT  TTCTTACAAATTGGATTT  TTCTTACAAATTGGATTT  TTCTTACAAATTGGATTT  TTCTTACAAATTGGATTT  TTCTTACAAATTGGATTT  TTCTT----ATTGGATTT  TTCTT----ATTGGATTT  TTCTT----ATTGGATTT  TTCTT----ATTGGATTT  TTCTT----ATTGGATTT  TTCTT----ATTGGATTT  TTCTT----ATTGGATTT  TTCTT----ATTGGATTT  TTCTTACAAATTGGATTT  TTCTTACAAATTGGATTT  TTCTTACAAATTGGATTT  TTCTTACAAATTGGATTT  TTCTTACAAATTGGATTT  TTCTTACAAATTGGATTT  TTCTTACAAATTGGATTT  TTCTTACAAATTGGATTT  TTCTTACAAATTGGATTT  TTCTTACAAATTGGATTT  TTCTTACAAATTGGATTT  TTCTTACAAATTGGATTT  TTCTTACAAATTGGATTT  TTCTTACAAATTGGATTT  TTCTTACTAATTGGATTT  TTCTTACAAATTGGATTT |  | Aegilops+Triticum |
| 21 | Schedonorus arundinaceus Schedonorus arundinaceus[C] Schedonorus pratensis  Festuca altissima  Festuca ovina  Lolium multiflorum  Lolium perenne  Dactylis glomerata  Helictochloa hookeri  Deschampsia antarctica  Poa palustris  Phleum alpinum  Briza sp  Puccinellia nuttalliana  Ammophila breviligulata  Agrostis stolonifera  Hierochloe odorata  Anthoxanthum odoratum  Phalaris arundinacea  Torreyochloa  Avena sativa  Trisetum cernuum  Aegilops cylindrica  Aegilops geniculata  Aegilops speltoides  Aegilops tauschii  Triticum aestivum  Triticum monococcum aegilopoides Triticum monococcum (modified) Triticum urartu (modified)  Hordeum jubatum  Hordeum vulgare  Hordeum vulgare spontaneum Secale cereale  Bromus vulgaris  Brachypodium distachyon  Diarrhena obovata  Melica mutica  Melica subulata  Oryzopsis asperifolia Achnatherum hymenoides Ampelodesmos mauritanica Piptochaetium avenaceum  Phaenosperma globosum Brachyelytrum aristosum  Bambusa bambosa | 10441  IGS |  | GATAGAGAC-------AAGAAATAACGA  GATAGAGAC-------AAGAAATAACGA  GATAGAGAC-------AAGAAATAAGGA  GATAGAGAC-------AAGAAATAACGA  GATAGAAAC-------AAGAAACAACGA  GATAGAGAC-------AAGAAATAAGGA  GATAGAGAC-------AAGAAATAAGGA  GATAGAGAC-------AAGAAATAACGA  GATAGAGAC-------AAGAAATAACGA  GATAGAGAC-------AACAAATAACGA  GATAGAGAC-------AAGAAATAACGA  AATAGAGAC-------AAGAAATAACGA  GATAGAGAC-------AAGAAATAACGA  GATAGAGAC-------AAGAAATAACGA  GATAGAGAC-------AAGAAATAACGA  GATAGAGAC-------AAGAAATAACGA  GATAGAGAC-------AAGAAATAACGA  GATAGAGAC-------AAGAAATAAGGA  GATAGAGAC-------AAGAAATAACGA  GATAGAGAC-------AAGAAATAACGA  GATAGAGAC-------AAGAAATAATGA  GATAGAGAC-------AAGAAATAATGA  GATATAGGCAAGAAATAAGAAATAACGG  GATATAGGCAAGAAATAAGAAATAACGG  GATATAGGCAAGAAATAAGAAATAATGG  GATATAGGCAAGAAATAAGAAATAACGG  GATATAGGCAAGAAATAAGAAATAATGG  GATATAGGCAAGAAATAAGAAATAACGG  GATATAGGCAAGAAATAAGAAATAACGG  GATATAGGCAAGAAATAAGAAATAACGG  GATATAGGCAAGAAATAAGAAATAACGG  GATATAGGCAAGAAATAAGAAATAACGG  GATATAGGCAAGAAATAAGAAATAACGG  GATATAGGCAAGAAATAAGAAATAACGG  GATATAGGC-------AAGAAATAACGG  GATAGAAGC-------AAGAAATAACGA  GATAGAGGC-------AAGAAATAACGA  GACAGAGGC-------AAGAAATAACGA  GACAGAGGC-------AAGAAATAACGA  GACAGAGGC-------AAGAAATAACGA  GACAGAGGC-------AAGAAATAACGA  GACAGAGGC-------AAGAAATAACGA  GACAGAGGC-------AAGAAATAACGA  GACAGAGGC-------AAGAAATAACGA  GACAGAAGCAA-----GAGAAATAATGA  GACAGAGGC-------AAGAAATAACGA |  | Triticeae |
| 22 | Schedonorus arundinaceus Schedonorus arundinaceus[C] Schedonorus pratensis  Festuca altissima  Festuca ovina  Lolium multiflorum  Lolium perenne  Dactylis glomerata  Helictochloa hookeri  Deschampsia antarctica  Poa palustris  Phleum alpinum  Briza sp  Puccinellia nuttalliana  Ammophila breviligulata  Agrostis stolonifera  Hierochloe odorata  Anthoxanthum odoratum  Phalaris arundinacea  Torreyochloa  Avena sativa  Trisetum cernuum  Aegilops cylindrica  Aegilops geniculata  Aegilops speltoides  Aegilops tauschii  Triticum aestivum  Triticum monococcum aegilopoides Triticum monococcum (modified) Triticum urartu (modified)  Hordeum jubatum  Hordeum vulgare  Hordeum vulgare spontaneum Secale cereale  Bromus vulgaris  Brachypodium distachyon  Diarrhena obovata  Melica mutica  Melica subulata  Oryzopsis asperifolia Achnatherum hymenoides Ampelodesmos mauritanica Piptochaetium avenaceum  Phaenosperma globosum Brachyelytrum aristosum  Bambusa bambosa | 10783  IGS |  | TGTCGATTGTCAATTATTTAT  TGTCGATTGTCAATTATTTAT  TGTCGATTGTCAATTATTTAT  TGTCG-------ATTATTTAT  TGTCA-------ATTATTTAT  TGTCGATTGTCAATTATTTAT  TGTCGATTGTCAATTATTTAT  TGTCG-------ATTATTTAT  TGTCG-------ATTATTTAT  TGTCG-------ATTATTTAT  TGTCG-------ATTATTTAT  TGTCG-------ATTATTTAT  TGTTG-------ATTACTTAT  TGTTG-------ATTATTTAT  TGTCG-------ATTATTTAT  TGTCG-------ATTATTTAT  TGTCG-------ATTATTTAT  TGTCG-------ATTATTTAT  TGTCG-------ATTATTTAT  TGTCG-------AGTATTTAT  TGTTG-------ATTATTTAT  TGTCG-------ATTATTTAT  TGTCG-------ATTATTTAT  TGTCG-------ATTATTTAT  TGTCG-------ATTATTTAT  TGTCG-------ATTATTTAT  TGTCG-------ATTATTTAT  TGTCG-------ATTATTTAT  TGTCG-------ATTATTTAT  TGTCG-------ATTATTTAT  TATCG-------ATTTTTTAT  TGTCG-------ATTATTTAT  TGTCG-------ATTATTTAT  TGTCG-------ATTATTTAT  TGTAG-------ATTATTTAT  TTTCG-------ATTTTTTAT  TGTCG-------ATTATTTAT  TGTTA-------ATTATTTAT  TGTTA-------ATTATTTAT  TGTCG-------ATTATTTAT  TGTCG-------ATTATTTAT  TGTCG-------ATTATTTAT  TGTCG-------ATTATTTAT  TGTCG-------ATTACTTAT  TTTTG-------ATTATTTAT  TTTTG-------ATTATTTAT |  | Lolium+Schedonorus |
| 23 | Schedonorus arundinaceus Schedonorus arundinaceus[C] Schedonorus pratensis  Festuca altissima  Festuca ovina  Lolium multiflorum  Lolium perenne  Dactylis glomerata  Helictochloa hookeri  Deschampsia antarctica  Poa palustris  Phleum alpinum  Briza sp  Puccinellia nuttalliana  Ammophila breviligulata  Agrostis stolonifera  Hierochloe odorata  Anthoxanthum odoratum  Phalaris arundinacea  Torreyochloa  Avena sativa  Trisetum cernuum  Aegilops cylindrica  Aegilops geniculata  Aegilops speltoides  Aegilops tauschii  Triticum aestivum  Triticum monococcum aegilopoides Triticum monococcum (modified) Triticum urartu (modified)  Hordeum jubatum  Hordeum vulgare  Hordeum vulgare spontaneum Secale cereale  Bromus vulgaris  Brachypodium distachyon  Diarrhena obovata  Melica mutica  Melica subulata  Oryzopsis asperifolia Achnatherum hymenoides Ampelodesmos mauritanica Piptochaetium avenaceum  Phaenosperma globosum Brachyelytrum aristosum  Bambusa bambosa | 11330  IGS |  | GTTTA----TTTC-TAG  GTTTA----TTTC-TAG  GTTTA----TTTC-TAG  GTTTA----TTTC-TAG  GTTTA----TTTC-TAT  GTTTA----TTTC-TAG  GTTTA----TTTC-TAG  GTTTA----TTTC-TAT  GTTTA----TTTCTTAT  GTTTA----TTTC-TAT  GTTTA----TTTC-TAT  GTTTA----TTTC-TAT  GTGGA----TTTC-TAT  GTTTA----TTTC-TAT  GTTGA----TTTC-TAT  GTTGA----TTTC-TAT  GTTGA----TTTC-TAT  GTGGA----TTTC-TAT  GTTTA----TTTC-TAT  GTTGA----TTTC-TAT  GTTGA----TTTC-TAT  GTTGA----TTTC-TAT  GTTTATTTCTTTC-TAT  GTTTATTTCTTTC-TAT  GTTTATTTCTTTC-TAT  GTTTATTTCTTTC-TAT  GTTTATTTCTTTC-TAT  GTTTATTTCTTTC-TAT  GTTTATTTCTTTC-TAT  GTTTATTTCTTTC-TAT  GTTTA----TTTC-TAT  GTTTA----TTTC-TAT  GTTTA----TTTC-TAT  GTTTATTTCTTTC-TAT  ---------TTTC-TAT  GTTTA----GTTC-TTT  GTTTA----TTTC-TAT  GTTTA----TTTC-TAT  ATTTA----TTTC-TAT  GTTTA----TTTC-TAT  GTTTA----TTTC-TAT  GTTTA----TTTC-TAT  GTTTA----TTTC-TAT  GTTTA----TTTC-TAT  GTTTA----TTTC-TAT  GTTTA----TTTC-TAT | Ssmp | Aegilops+Triticum+Secale |
| 24 | Schedonorus arundinaceus Schedonorus arundinaceus[C] Schedonorus pratensis  Festuca altissima  Festuca ovina  Lolium multiflorum  Lolium perenne  Dactylis glomerata  Helictochloa hookeri  Deschampsia antarctica  Poa palustris  Phleum alpinum  Briza sp  Puccinellia nuttalliana  Ammophila breviligulata  Agrostis stolonifera  Hierochloe odorata  Anthoxanthum odoratum  Phalaris arundinacea  Torreyochloa  Avena sativa  Trisetum cernuum  Aegilops cylindrica  Aegilops geniculata  Aegilops speltoides  Aegilops tauschii  Triticum aestivum  Triticum monococcum aegilopoides Triticum monococcum (modified) Triticum urartu (modified)  Hordeum jubatum  Hordeum vulgare  Hordeum vulgare spontaneum Secale cereale  Bromus vulgaris  Brachypodium distachyon  Diarrhena obovata  Melica mutica  Melica subulata  Oryzopsis asperifolia Achnatherum hymenoides Ampelodesmos mauritanica Piptochaetium avenaceum  Phaenosperma globosum Brachyelytrum aristosum  Bambusa bambosa | 11505  IGS |  | CACGACCTCGGT------TGTGCCTCCA  CACGACCTCGGT------TGTGCCTCCA  CACGACCTCGGT------TGTGCCTCCA  CACGACCTCGGT------TGTGACTCCA  CAAGACCTCGGT------TGTGACTCCA  CACGACCCCGGT------TGTGCCTCCA  CACGACCCCGGT------TGTGCCTCCA  CACGACCTCGGT------TGTGACTCCA  TACGGCCTAGGT------TGTGACTCCA  CACGACCTCGGT------TGTGACTCCA  CACGACCTCGGT------TGTGACTCCA  CACGACCTTGGT------TGTGACTCCA  CACGACCTCGGT------TGTGACTCCA  CACGACCTCGGT------TGTGACTCCA  CACGACCTCGGT------TGTGACTCCA  CACGACCTCGGT------TGTGACTCCA  CACGACCTCGGT------TGTGACTCCA  CACGACCCCGGT------TGTGACTCCA  CACGACCTCGGT------TGTGACTCCA  CACGACCTCGGT------TGTGACTCCA  CACGACCTCGGT------TGTGACTCCC  CACGACCTCGGT------TGTGACTCCA  CACGACCTCGGTTGTGACTGTGACTCCA  CACGACCTCGGTTGTGACTGTGACTCCA  CACGACCTCGGT------TGTGACTCCA  CACGACCTCGGTTGTGACTGTGACTCCA  CACGACCTCGGT------TGTGACTCCA  CACGACCTCGGT------TGTGACTCCA  CACGACCTCGGTTGTGACTGTGACTCCA  CACGACCTCGGTTGTGACTGTGACTCCA  CACGACCTCGGT------TGTGACTCCA  AACGACCTCGGT------TGTGACTCCA  AACGACCTCGGT------TGTGACTCCA  CACGACCTCGAT------TGTGACTCCA  CACGACCGAGGT------CGTGACTCCA  CACGACCTCGGT------TGTGACTCCA  CACGACCTCGGT------TGTGACTCCA  CACGACCTCGGT------TGTGACTCCA  CACGACCTCGGT------TGTGACTCCA  CACGACCTCGGT------TGTGACTCCA  CACGACCCCGGT------TGTGACTCCA  CACGACCTCGGT------TGTGACTCCA  CACGACCTCGGT------TGTGACTCCA  CACGCCCTCGGT------TGTGACTCCA  CACAACCTCGGT------TGTGACCCCA  CACGACCTCGGT------TGTGACCCCA | ssmp | Aegilops+Triticum minues A. speltoides and T. aestivum |
| 25 | Schedonorus arundinaceus Schedonorus arundinaceus[C] Schedonorus pratensis  Festuca altissima  Festuca ovina  Lolium multiflorum  Lolium perenne  Dactylis glomerata  Helictochloa hookeri  Deschampsia antarctica  Poa palustris  Phleum alpinum  Briza sp  Puccinellia nuttalliana  Ammophila breviligulata  Agrostis stolonifera  Hierochloe odorata  Anthoxanthum odoratum  Phalaris arundinacea  Torreyochloa  Avena sativa  Trisetum cernuum  Aegilops cylindrica  Aegilops geniculata  Aegilops speltoides  Aegilops tauschii  Triticum aestivum  Triticum monococcum aegilopoides Triticum monococcum (modified) Triticum urartu (modified)  Hordeum jubatum  Hordeum vulgare  Hordeum vulgare spontaneum Secale cereale  Bromus vulgaris  Brachypodium distachyon  Diarrhena obovata  Melica mutica  Melica subulata  Oryzopsis asperifolia Achnatherum hymenoides Ampelodesmos mauritanica Piptochaetium avenaceum  Phaenosperma globosum Brachyelytrum aristosum  Bambusa bambosa | 16726  trnG-UCC intron 1 | trnG-UCC intron 1 | TTATTCCTT-TTTTAA  TTATTCCTT-TTTTAA  TTATTCCTT-TTTTAA  TTATTCCTT-TTTTAA  TTATTCCTT-TTTTAA  TTATTCCTT-TTTTAA  TTATTCCTT-TTTTAA  TTATTCCTT-TTTTAA  TTATTCCTT-TTTTAA  TTATTCCTT-TTTTAA  TTATTCCTT-TTTTAA  TTATTCCTT-TTTTAA  TTATTCCTT-TTTTAA  TTATTCCTT-TTTTAA  TTATTCCTTATTTTAA  TTATTCCTTATTTTAA  TTATTCCTTATTTTAA  TTAATTCCTTTTTTAA  TTATTCCTT-TTTTAA  TTATTCCTT-TTTTAA  TTATTCCTT-TTTTAA  TTATTCCTT-TTTTAA  TTATTCCTTATTTT--  TTATTCCTTATTTT--  TTATTCCTTATTTT--  TTATTCCTTATTTT--  TTATTCCTTATTTT--  ---TTCCTTATTTT--  ---TTCCTTATTTT--  TTATTCCTTATTTT--  TTAT-----CCTTTAA  TTAT-----CCTTTAA  TTAT-----CCTTTAA  TTATTCCTTATTTTAA  TTATTCCTTATTTTAA  TTTTCCCTTTTTGAAG  --TTTTCCTTTTTTAA  --ATTTCCTTTTTTAA  --ATTTCCTTTTTTAA  --ATTTCCTTTTTTAA  --ATTTCCTTTTTTAA  --ATTTCCTTTTTTAA  --ATTTCCTTTTTTAA  --ATTTCCTTTTTTAA  --ATTTCCTTTTTTAA  --ATTTCCTTTTTTAA |  | Hordeum |
| 26 | Schedonorus arundinaceus Schedonorus arundinaceus[C] Schedonorus pratensis  Festuca altissima  Festuca ovina  Lolium multiflorum  Lolium perenne  Dactylis glomerata  Helictochloa hookeri  Deschampsia antarctica  Poa palustris  Phleum alpinum  Briza sp  Puccinellia nuttalliana  Ammophila breviligulata  Agrostis stolonifera  Hierochloe odorata  Anthoxanthum odoratum  Phalaris arundinacea  Torreyochloa  Avena sativa  Trisetum cernuum  Aegilops cylindrica  Aegilops geniculata  Aegilops speltoides  Aegilops tauschii  Triticum aestivum  Triticum monococcum aegilopoides Triticum monococcum (modified) Triticum urartu (modified)  Hordeum jubatum  Hordeum vulgare  Hordeum vulgare spontaneum Secale cereale  Bromus vulgaris  Brachypodium distachyon  Diarrhena obovata  Melica mutica  Melica subulata  Oryzopsis asperifolia Achnatherum hymenoides Ampelodesmos mauritanica Piptochaetium avenaceum  Phaenosperma globosum Brachyelytrum aristosum  Bambusa bambosa | 16737  trnG-UCC intron 1 | trnG-UCC intron 1 | TTTAACAAAAGA  TTTAACAAAAGA  TTTAACAAAAGA  TTTAACAAAAGA  TTTAACAAAAGA  TTTAACAAAAGA  TTTAACAAAAGA  TTTAACAAAAGA  TTTAACAAAAGA  TTTAACAAAAGA  TTTAACAAAAGA  TTTAACAAAAGA  TTTAACAAAAGA  TTTAACAAAAGA  TTTAACAAAAGA  TTTAACAAAAGA  TTTAACAAAAGA  TTTAACAAAAGA  TTTAACAAAAGA  TTTAACAAAAGA  TTTAACAAAAGA  TTTAACAAAAGA  TTT-----AAGA  TTT-----AAGA  TTT-----AAGA  TTT-----AAGA  TTT-----AAGA  TTT-----AAGA  TTT-----AAGA  TTT-----AAGA  TTTAACAAAAGA  TTTAACAAAAGA  TTTAACAAAAGA  TTTAACAAAAGA  TTTAACAAAAGA  TGAAGGTAAAGG  TTTAAAAAAAGG  TTTAACAAAAGA  TTTAACAAAAGA  TTTAACAAAAGA  TTTAACAAAAGA  TTTAACAAAAGA  TTTAACAAAAGA  TTTAACAAAAGA  TTTAACAAAAGA  TTTAACAAAAGA |  | Aegilops+Triticum |
| 27 | Schedonorus arundinaceus Schedonorus arundinaceus[C] Schedonorus pratensis  Festuca altissima  Festuca ovina  Lolium multiflorum  Lolium perenne  Dactylis glomerata  Helictochloa hookeri  Deschampsia antarctica  Poa palustris  Phleum alpinum  Briza sp  Puccinellia nuttalliana  Ammophila breviligulata  Agrostis stolonifera  Hierochloe odorata  Anthoxanthum odoratum  Phalaris arundinacea  Torreyochloa  Avena sativa  Trisetum cernuum  Aegilops cylindrica  Aegilops geniculata  Aegilops speltoides  Aegilops tauschii  Triticum aestivum  Triticum monococcum aegilopoides Triticum monococcum (modified) Triticum urartu (modified)  Hordeum jubatum  Hordeum vulgare  Hordeum vulgare spontaneum Secale cereale  Bromus vulgaris  Brachypodium distachyon  Diarrhena obovata  Melica mutica  Melica subulata  Oryzopsis asperifolia Achnatherum hymenoides Ampelodesmos mauritanica Piptochaetium avenaceum  Phaenosperma globosum Brachyelytrum aristosum  Bambusa bambosa | 16865  trnG-UCC intron 1 | trnG-UCC intron 1 | TGTAC-----TCA-TAAT  TGTAC-----TCA-TAAT  TGTAC-----TCA-TAAT  TGTAC-----TCA-TAAT  TGTAC-----TCA-TAAT  TGTAC-----TCA-TAAT  TGTAC-----TCA-TAAT  TGTAC-----TCA-TAAT  TGTAC-----TCACTAAT  TGTAC-----TCA-TAAT  TGTAC-----TCA-TAAT  TGTAC-----TCA-TAAT  TGTAC-----TCA-CAAT  TGTAC-----TCA-TAAT  TGTAC-----TCA-TAAT  TGTAC-----TCA-TAAT  TGTAC-----TCA-TAAT  TGTAC-----TCA-TAAT  TGTAC-----TCA-TAAT  TGTAC-----TCA-TAAT  TGTAC-----TCC-TAAT  TGTAC-----TCC-TAAT  TGTAC-----TCA-TAAT  TGTAC-----TCA-TAAT  TGTAC-----TCA-TAAT  TGTAC-----TCA-TAAT  TGTAC-----TCA-TAAT  TGTAC-----TCA-TAAT  TGTAC-----TCA-TAAT  TGTAC-----TCA-TAAT  TGTACTCATATCA-TAAT  TGTACTCATATCA-TAAT  TGTACTCATATCA-TAAT  TGTAC-----TCA-TAAT  TGTAC-----TCA-AAAT  TGTAC-----TCA-TAAT  TGTAC-----TCA-TAAT  TGTAC-----TCC-TAAT  TGTAC-----TCC-TAAT  TGTAC-----TCA-TAAT  TGTAC-----TCA-TAAT  TGTAC-----TCA-TAAT  TGTAC-----TCA-TAAT  TGTAC-----TCA-TAAT  TGTAT-----TCA-TAAT  TGTAC-----TCA-TAAT |  | Hordeum |
| 28 | Schedonorus arundinaceus Schedonorus arundinaceus[C] Schedonorus pratensis  Festuca altissima  Festuca ovina  Lolium multiflorum  Lolium perenne  Dactylis glomerata  Helictochloa hookeri  Deschampsia antarctica  Poa palustris  Phleum alpinum  Briza sp  Puccinellia nuttalliana  Ammophila breviligulata  Agrostis stolonifera  Hierochloe odorata  Anthoxanthum odoratum  Phalaris arundinacea  Torreyochloa  Avena sativa  Trisetum cernuum  Aegilops cylindrica  Aegilops geniculata  Aegilops speltoides  Aegilops tauschii  Triticum aestivum  Triticum monococcum aegilopoides Triticum monococcum (modified) Triticum urartu (modified)  Hordeum jubatum  Hordeum vulgare  Hordeum vulgare spontaneum Secale cereale  Bromus vulgaris  Brachypodium distachyon  Diarrhena obovata  Melica mutica  Melica subulata  Oryzopsis asperifolia Achnatherum hymenoides Ampelodesmos mauritanica Piptochaetium avenaceum  Phaenosperma globosum Brachyelytrum aristosum  Bambusa bambosa | 19610  IGS |  | TTT--TTTTAA  TTT--TTTTAA  TTT--TTTTAA  TTT--TTTTAA  TTT--TTTTAA  TTT--TTTTAA  TTT--TTTTAA  TTT--TTTTAA  TTT--TTTTAA  TTT--TTTTAA  TTT--TTTTAA  TTT--TTTTAA  TTGGAATTAAA  TTT--TTTTAA  TTTGAATTAAA  TTTGAATTAAA  TTTGAATTAAA  TTTGAATTAAA  TTTGAATCAAA  TTTGAATTAAA  TTTGAATTAAA  TTTGAATTAAA  TTTGAATTAAA  TTTGAATTAAA  TTTGAATTAAA  TTTGAATTAAA  TTTGAATTAAA  TTTGAATTAAA  TTTGAATTAAA  TTTGAATTAAA  TTTGAATTAAA  TTTGAATTAAA  TTTGAATTAAA  TTTGAATTAAA  TTTGAATTAAG  TTTGAATTAAA  TTTGAATTAAA  TTTGAATTAA-  TTTAAATT-A-  TTTGAATTGAA  TTTGAATTGAA  TTTGAATTGAA  TTTGAATTGAA  TTTGAATTGAA  TTTGAATTGAA  TTGGAATTGAG |  | Poeae clade 2 |
| 29 | Schedonorus arundinaceus Schedonorus arundinaceus[C] Schedonorus pratensis  Festuca altissima  Festuca ovina  Lolium multiflorum  Lolium perenne  Dactylis glomerata  Helictochloa hookeri  Deschampsia antarctica  Poa palustris  Phleum alpinum  Briza sp  Puccinellia nuttalliana  Ammophila breviligulata  Agrostis stolonifera  Hierochloe odorata  Anthoxanthum odoratum  Phalaris arundinacea  Torreyochloa  Avena sativa  Trisetum cernuum  Aegilops cylindrica  Aegilops geniculata  Aegilops speltoides  Aegilops tauschii  Triticum aestivum  Triticum monococcum aegilopoides Triticum monococcum (modified) Triticum urartu (modified)  Hordeum jubatum  Hordeum vulgare  Hordeum vulgare spontaneum Secale cereale  Bromus vulgaris  Brachypodium distachyon  Diarrhena obovata  Melica mutica  Melica subulata  Oryzopsis asperifolia Achnatherum hymenoides Ampelodesmos mauritanica Piptochaetium avenaceum  Phaenosperma globosum Brachyelytrum aristosum  Bambusa bambosa | 20338  IGS |  | TGATTATAGTATAATCACAGTATAATCACGAGCGGTTG  TGATTATAGTATAATCACAGTATGATCACGAGCGGTTG  TGATTATAGTATAATCACAGTATAATCACGAGCGGTTG  TTATTAT-----------AGTATAATCACGAGCGGTTG  TGATTAT-----------AGTATAATCACGAGCGGTTG  TGATTATAGTATAATCACAGTATAATCACGAGCGGTTG  TGATTATAGTATAATCACAGTATAATCACGAGCGGTTG  TGATTAT-----------AGTATAATCACGAGCGGTTG  TGATTAT-----------AGTATAATCACGAGCGGTTG  TAATTAT-----------AGTATAATCACGAACGGTTG  TGATTAT-----------AGTATAATCACGAGCGGTTG  TGATTAT-----------AGTATAATCACGAGCGGTTG  TGATTAT-----------AGTATAATCACGAGCGGTTG  TGATTAT-----------AGTATAATCACGAGCGGTTG  TGATTAT-----------AGTATAATCACGAGCGGTTG  TGATTAT-----------AGTATAATCACGAGCGGTTG  TGATTAT-----------AGTATAATCACGAGCGGTTG  TGATTAT-----------AGTATAATCACGAGCGGTTG  TGATTAT-----------AGTATAATCACGAGCGGTTG  TGATTAT-----------AGTATAATCACGAGCGGTTG  TGATTAT-----------AGTATAATCACGAGCGGTTG  TGATTAT-----------AGTATAATCACGAGCGGTTG  TGATTAT-----------AGTATAATCACGAGCGGTTG  TGATTAT-----------AGTATAATCACGAGCGGTTG  TGATTAT-----------AGTATAATCACGAGCGGTTG  TGATTAT-----------AGTATAATCACGAGCGGTTG  TGATTAT-----------AGTATAATCACGAGCGGTTG  TGATTAT-----------AGTATAATCACGAGCGGTTG  TGATTAT-----------AGTATAATCACGAGCGGTTG  TGATTAT-----------AGTATAATCACGAGCGGTTG  TGATTAT-----------AGTATAATCACGAGCGGTTG  TGATTAT-----------AGTATAATCACGAGCGGTTG  TGATTAT-----------AGTATAATCACGAGCGGTTG  TGATTAT-----------AGTATAATCACGAGCGGTTG  TGATTAT-----------AGTATAATCACGAGCGGTTG  TGATTAT-----------AGTATAATCACGAGCGGTTA  TGATTAT-----------AGTATAATCACGAGCGGTTG  TAATTAT-----------AGTATAATCACGAGCGGTTG  TAATTAT-----------AGTATAATCACGAGCGGTGG  TGATTAT-----------AGTATAATCACGAGCGGTTG  TGATTAT-----------AGTATAATCACGAGCGGTTG  TGATTAT-----------AGTATAATCACGAGCGGTTG  TGATTAT-----------AGTATAATCACGAGCGGTTG  TGATTAT-----------AGTATAATCACGAGCGGTTG  TCATTAT-----------AGTATAATGACGAGCGGTTG  TCATTAT-----------AGTATAATGACGAGCGGTTG | ssmp | Lolium+Schedonorus |
| 30 | Schedonorus arundinaceus Schedonorus arundinaceus[C] Schedonorus pratensis  Festuca altissima  Festuca ovina  Lolium multiflorum  Lolium perenne  Dactylis glomerata  Helictochloa hookeri  Deschampsia antarctica  Poa palustris  Phleum alpinum  Briza sp  Puccinellia nuttalliana  Ammophila breviligulata  Agrostis stolonifera  Hierochloe odorata  Anthoxanthum odoratum  Phalaris arundinacea  Torreyochloa  Avena sativa  Trisetum cernuum  Aegilops cylindrica  Aegilops geniculata  Aegilops speltoides  Aegilops tauschii  Triticum aestivum  Triticum monococcum aegilopoides Triticum monococcum (modified) Triticum urartu (modified)  Hordeum jubatum  Hordeum vulgare  Hordeum vulgare spontaneum Secale cereale  Bromus vulgaris  Brachypodium distachyon  Diarrhena obovata  Melica mutica  Melica subulata  Oryzopsis asperifolia Achnatherum hymenoides Ampelodesmos mauritanica Piptochaetium avenaceum  Phaenosperma globosum Brachyelytrum aristosum  Bambusa bambosa | 20494  IGS |  | TAATC-----ATAGAT  TAATC-----ATAGAT  TAATC-----ATAGAT  TAATC-----ATAGAT  TAATC-----ATAGAT  TAATC-----ATAGAT  TAATC-----ATAGAT  TAATC-----ATAGAT  TAATC-----ATAGAT  TAATC-----ATAGAT  TAATC-----ATAGAT  TAATC-----ATAGAT  TAATC-----ATAGAT  TAATC-----ATAGAT  TAATC-----ATAGAT  TAATC-----ATAGAT  TAATC-----ATAGAT  TAATC-----ATAGAT  TAATC-----ATAGAT  TAATC-----ATAGAT  TAATC-----ATAGAT  TAATC-----ATAGAT  TAATC-----ATAGAT  TAATC-----ATAGAT  TAATC-----ATAGAT  TAATC-----ATAGAT  TAATC-----ATAGAT  TAATC-----ATAGAT  TAATC-----ATAGAT  TAATC-----ATAGAT  TAATC-----ATAGAT  TAATC-----ATAGAT  TAATC-----ATAGAT  TAATC-----ATAGAT  TAATC-----ATAGAT  TAATC-----ATAGAT  TAATC-----ATAGAT  TAATCATCGAATAGAT  TAATCATAGAATAGAT  TAATC-----ATAGAT  TGATC-----ATAGAT  TAATC-----ATAGAT  TAATC-----ATAGAT  TAATC-----ATAGAT  TGATC-----ATAGAT  TAATC-----ATAGAT |  | Melica |
| 31 | Schedonorus arundinaceus Schedonorus arundinaceus[C] Schedonorus pratensis  Festuca altissima  Festuca ovina  Lolium multiflorum  Lolium perenne  Dactylis glomerata  Helictochloa hookeri  Deschampsia antarctica  Poa palustris  Phleum alpinum  Briza sp  Puccinellia nuttalliana  Ammophila breviligulata  Agrostis stolonifera  Hierochloe odorata  Anthoxanthum odoratum  Phalaris arundinacea  Torreyochloa  Avena sativa  Trisetum cernuum  Aegilops cylindrica  Aegilops geniculata  Aegilops speltoides  Aegilops tauschii  Triticum aestivum  Triticum monococcum aegilopoides Triticum monococcum (modified) Triticum urartu (modified)  Hordeum jubatum  Hordeum vulgare  Hordeum vulgare spontaneum Secale cereale  Bromus vulgaris  Brachypodium distachyon  Diarrhena obovata  Melica mutica  Melica subulata  Oryzopsis asperifolia Achnatherum hymenoides Ampelodesmos mauritanica Piptochaetium avenaceum  Phaenosperma globosum Brachyelytrum aristosum  Bambusa bambosa | 20579  trnY-GUA | trnY-GUA | TCGTTGACGATATGTCTAC  TCGTTGACGATATGTCTAC  TCGTTGACGATATGTCTAC  TCGTTGACGATATGTCTAC  TCGTTGACGATATGTCTAC  TCGTTGACGATATGTCTAC  TCGTTGACGATATGTCTAC  TCGTTGACGATATGTCTAC  TCGTTGACGATATGTCTAC  TCGTTGACGATATGTCTAC  TCGTTGACGATATGTCTAC  TCGTTGACGATATGTCTAC  TCGTTGACAATATGTCTAC  TCGTTGACGATATGTCTAC  TCGTTGACAATATGTCTAC  TCGTTGACAATATGTCTAC  TCGTTGACAATATGTCTAC  TCGTTGACAATATGTCTAC  TCGTTGACAATATGTCTAC  TCGTTGACAATATGTCTAC  TCGTTGACAATATGTCTAC  TCGTTGACAATATGTCTAC  TCGTTGACA--ATGTCTAC  TCGTTGACA--ATGTCTAC  TCGTTGACA--ATGTCTAC  TCGTTGACA--ATGTCTAC  TCGTTGACA--ATGTCTAC  TCGTTGACA--ATGTCTAC  TCGTTGACA--ATGTCTAC  TCGTTGACA--ATGTCTAC  TCGTTGACA--ATGTCTAC  TCGTTGACA--ATGTCTAC  TCGTTGACA--ATGTCTAC  TCGTTGACA--ATGTCTAC  TCGTTGACA--ATGTCTAC  TCGTTGACAAAATGTCTAC  TCGTTGACAATATGTCTAC  TCGTTGACAATATGTCTAC  TCGTTGACAATATGTCTAC  TCGTTGACAATATGTCTAC  TCGTTGACAATATGTCTAC  TCGTTGACAATATGTCTAC  TCGTTGACAATATGTCTAC  TCGTTGACAATATGTCTAC  TCGTTGACAATATGTCTAC  TCGTTGACGATATGTCTAC |  | Bromeae+Triticeae |
| 32 | Schedonorus arundinaceus Schedonorus arundinaceus[C] Schedonorus pratensis  Festuca altissima  Festuca ovina  Lolium multiflorum  Lolium perenne  Dactylis glomerata  Helictochloa hookeri  Deschampsia antarctica  Poa palustris  Phleum alpinum  Briza sp  Puccinellia nuttalliana  Ammophila breviligulata  Agrostis stolonifera  Hierochloe odorata  Anthoxanthum odoratum  Phalaris arundinacea  Torreyochloa  Avena sativa  Trisetum cernuum  Aegilops cylindrica  Aegilops geniculata  Aegilops speltoides  Aegilops tauschii  Triticum aestivum  Triticum monococcum aegilopoides Triticum monococcum (modified) Triticum urartu (modified)  Hordeum jubatum  Hordeum vulgare  Hordeum vulgare spontaneum Secale cereale  Bromus vulgaris  Brachypodium distachyon  Diarrhena obovata  Melica mutica  Melica subulata  Oryzopsis asperifolia Achnatherum hymenoides Ampelodesmos mauritanica Piptochaetium avenaceum  Phaenosperma globosum Brachyelytrum aristosum  Bambusa bambosa | 20613  trnY-GUA | trnY-GUA | GCTCGGC-----CCAAAAAT  GCTCGGC-----CCAAAAAT  GCTCGGC-----CCAAAAAT  GCTCGGC-----CCAAAAAT  GCTCGGC-----CCAAAAAT  GCTCGGC-----CCAAAAAT  GCTCGGC-----CCAAAAAT  GCTCGGC-----CCAAAAAT  GCTCGGC-----CCAAAAAT  GCTCGGC-----CCAAAAAT  GCTCGGC-----CCAAAAAT  GCTCGGC-----CCAAAAAT  GCTCGGC-----CCAAAAAT  GCTCGGC-----CCAAAAAT  GCTCGGC-----CCAAAAAT  GCTCGGC-----CCAAAAAT  GCTCGGC-----CCAAAAAT  GCTCGGC-----CCAAAAAT  GCTCGGC-----CCAAAAAT  GCTCGGC-----CCAAAAAT  GCTCGGC-----CCAAAAAT  GCTCGGC-----CCAAAAAT  GCTCGGCCCAAACCAAAAAT  GCTCGGCCCAAACCAAAAAT  GCTCGGCCCAAACCAAAAAT  GCTCGGCCCAAACCAAAAAT  GCTCGGCCCAAACCAAAAAT  GCTCGGCCCAAACCAAAAAT  GCTCGGCCCAAACCAAAAAT  GCTCGGCCCAAACCAAAAAT  GCTCGGC-----CCAAAAAT  GCTCGGC-----CCAAAAAT  GCTCGGC-----CCAAAAAT  GCTCGGCCCAAACCAAAAAT  GCTCGGC-----CCAAAAAT  GCTCGGC-----CCAAAAAT  GCTCGGC-----CCAAAAAT  GCTCGGC-----CCAAAAAT  GCTCGGC-----CCAAAAAT  GCTCGGC-----CCAAAAAT  GCTCGGC-----CCAAAAAT  GCTCGGC-----CCAAAAAT  GCTCGGC-----CCAAAAAT  GCTCGGC-----CCCAAAAT  GCTCGGC-----CCAAAAAT  GCTCGGC-----CCAAAAAT | ssmp | Aegilops+Triticum+Secale |
| 33 | Schedonorus arundinaceus Schedonorus arundinaceus[C] Schedonorus pratensis  Festuca altissima  Festuca ovina  Lolium multiflorum  Lolium perenne  Dactylis glomerata  Helictochloa hookeri  Deschampsia antarctica  Poa palustris  Phleum alpinum  Briza sp  Puccinellia nuttalliana  Ammophila breviligulata  Agrostis stolonifera  Hierochloe odorata  Anthoxanthum odoratum  Phalaris arundinacea  Torreyochloa  Avena sativa  Trisetum cernuum  Aegilops cylindrica  Aegilops geniculata  Aegilops speltoides  Aegilops tauschii  Triticum aestivum  Triticum monococcum aegilopoides Triticum monococcum (modified) Triticum urartu (modified)  Hordeum jubatum  Hordeum vulgare  Hordeum vulgare spontaneum Secale cereale  Bromus vulgaris  Brachypodium distachyon  Diarrhena obovata  Melica mutica  Melica subulata  Oryzopsis asperifolia Achnatherum hymenoides Ampelodesmos mauritanica Piptochaetium avenaceum  Phaenosperma globosum Brachyelytrum aristosum  Bambusa bambosa | 22244  IGS |  | TCCTAT----TACTATT  TCCTAT----TACTATT  TCCTAT----TACTATT  TCCTATGTTATACTATT  TCCTATGTTATACTATT  TCCTAT----TACTATT  TCCTAT----TACTATT  TCCTATGTTATACTATT  TCCTATGTTATACTATT  TCCTATGTTATACTATT  TCCTATGTTATACTATT  TCCTATGTTATACTATT  TCCTATGTTATACTATT  TCCTATGTTATACTATT  TCCTATGTTATACTATT  TCCTATGTTATACTATT  TCTTATGTTATACTATT  TCCTATGTTATACTATT  TCCTATGTTATACTATT  TCCTATGTTATACTATT  TCCTATGTTATAGTATT  TCCTATGTTATAGTATT  TCCTATGTTATACTATA  TCCTATGTTATACTATA  TCCTATGTTATACTATA  TCCTATGTTATACTATA  TCCTATGTTATACTATA  TCCTATGTTATACTATA  TCCTATGTTATACTATA  TCCTATGTTATACTATA  TCCTATGTTATACTATA  TCCTATGTTATACTATA  TCCTATGTTATACTATA  TCCTATGTTATACTATA  TCCTATGTTATACTATA  TCCTATGTTATACTTTT  TCCTATGTTATACTATT  TCCTATGTTATACTATT  TCCTATGTTATACTATT  TCCTATATTATACTATT  TCCTATGTTATACTATT  TCCTATGTTATACTATT  TCCTATGTTATACTATT  TCCTATGTTATACTATT  TCCTATGTTATACTATT  TCCTATGTTATACTATT |  | Lolium+Schedonorus |
| 34 | Schedonorus arundinaceus Schedonorus arundinaceus[C] Schedonorus pratensis  Festuca altissima  Festuca ovina  Lolium multiflorum  Lolium perenne  Dactylis glomerata  Helictochloa hookeri  Deschampsia antarctica  Poa palustris  Phleum alpinum  Briza sp  Puccinellia nuttalliana  Ammophila breviligulata  Agrostis stolonifera  Hierochloe odorata  Anthoxanthum odoratum  Phalaris arundinacea  Torreyochloa  Avena sativa  Trisetum cernuum  Aegilops cylindrica  Aegilops geniculata  Aegilops speltoides  Aegilops tauschii  Triticum aestivum  Triticum monococcum aegilopoides Triticum monococcum (modified) Triticum urartu (modified)  Hordeum jubatum  Hordeum vulgare  Hordeum vulgare spontaneum Secale cereale  Bromus vulgaris  Brachypodium distachyon  Diarrhena obovata  Melica mutica  Melica subulata  Oryzopsis asperifolia Achnatherum hymenoides Ampelodesmos mauritanica Piptochaetium avenaceum  Phaenosperma globosum Brachyelytrum aristosum  Bambusa bambosa | 23386  IGS |  | GTG-----AAACT  GTG-----AAACT  GTG-----AAACT  GTG-----AAACT  GTG-----AAACT  ATG-----AAACT  ATG-----AAACT  GTG-----AAACT  GTG-----AAACT  GTG-----AAACT  GTGAAACAAAACT  GTGAAACAAAACT  GTGAAACAAAACT  GTGAAACAAAACT  GTGAAACAAAACT  GTGAAATAAAACT  GTGAAACAAAACT  GTGAAACAAAACT  GTGAAACAAAACT  GTGAAACAAAACT  GTGAAACAAAAC-  GTGAAACAAAACT  GTGAAACAAAACT  GTGAAACAAAACT  GTGAAACAAAACT  GTGAAACAAAACT  GTGAAACAAAACT  GTGAAACAAAACT  GTGAAACAAAACT  GTGAAACAAAACT  GTGAAACAAAACT  GTGAAACAAAACT  GTGAAACAAAACT  GTGAAACAAAACT  ATGAAACAAAACT  GTGAAACAAAACC  GTGAAACAAAACT  GTGAAAGAAAACT  GTGAAAGAAAACT  GTGAAACAAAACT  GTGAAACAAAACT  GTGAAACAAAACT  GTGAAACAAAACT  GTAAAACA-----  GTGAAACAAAACT  GTGAAACAAAACT |  | Airinae+Dactylidinae+Holcinae+Loliinae |
| 35 | Schedonorus arundinaceus Schedonorus arundinaceus[C] Schedonorus pratensis  Festuca altissima  Festuca ovina  Lolium multiflorum  Lolium perenne  Dactylis glomerata  Helictochloa hookeri  Deschampsia antarctica  Poa palustris  Phleum alpinum  Briza sp  Puccinellia nuttalliana  Ammophila breviligulata  Agrostis stolonifera  Hierochloe odorata  Anthoxanthum odoratum  Phalaris arundinacea  Torreyochloa  Avena sativa  Trisetum cernuum  Aegilops cylindrica  Aegilops geniculata  Aegilops speltoides  Aegilops tauschii  Triticum aestivum  Triticum monococcum aegilopoides Triticum monococcum (modified) Triticum urartu (modified)  Hordeum jubatum  Hordeum vulgare  Hordeum vulgare spontaneum Secale cereale  Bromus vulgaris  Brachypodium distachyon  Diarrhena obovata  Melica mutica  Melica subulata  Oryzopsis asperifolia Achnatherum hymenoides Ampelodesmos mauritanica Piptochaetium avenaceum  Phaenosperma globosum Brachyelytrum aristosum  Bambusa bambosa | 23445  IGS |  | AGATT---------AAAG  AGATT---------AAAG  AGATT---------AAAG  AGATT---------AAAG  AGATT---------AAAG  AGATT---------AAAG  AGATT---------AAAG  AGATT---------AAAG  AGATT---------AAAG  AGATT---------AAAG  AGATT---------AAAG  AGATT---------AAAG  AGATT---------AAAG  AGATT---------AAAG  AGATT---------AAAG  AGATT---------AAAG  AGATTAAAGAATAAAAAG  AGATTAAAGAATAAAAAG  AGATT---------AAAG  AGATT---------AAAG  AGATT---------AAAG  AGATT---------AAAG  AGATT---------AAAG  AGATT---------AAAG  AGATT---------AAAG  AGATT---------AAAG  AGATT---------AAAG  AGATT---------AAAG  AGATT---------AAAG  AGATT---------AAAG  AGATT---------AAAG  AGATT---------AAAG  AGATT---------AAAG  AGATT---------AAAG  AGATT---------AAAG  AGAAA---------AAAG  AGAGT---------AAAG  AGAGT---------AAAG  AGAGT---------AAAG  AGAGT---------AAAG  AGAAT---------AAAG  AGAGT---------AAAG  AGAGT---------AAAG  AGAGT---------AAAG  AGAGT---------AAAG  --------------AAAG |  | Anthoxanthinae |
| 36 | Schedonorus arundinaceus Schedonorus arundinaceus[C] Schedonorus pratensis  Festuca altissima  Festuca ovina  Lolium multiflorum  Lolium perenne  Dactylis glomerata  Helictochloa hookeri  Deschampsia antarctica  Poa palustris  Phleum alpinum  Briza sp  Puccinellia nuttalliana  Ammophila breviligulata  Agrostis stolonifera  Hierochloe odorata  Anthoxanthum odoratum  Phalaris arundinacea  Torreyochloa  Avena sativa  Trisetum cernuum  Aegilops cylindrica  Aegilops geniculata  Aegilops speltoides  Aegilops tauschii  Triticum aestivum  Triticum monococcum aegilopoides Triticum monococcum (modified) Triticum urartu (modified)  Hordeum jubatum  Hordeum vulgare  Hordeum vulgare spontaneum Secale cereale  Bromus vulgaris  Brachypodium distachyon  Diarrhena obovata  Melica mutica  Melica subulata  Oryzopsis asperifolia Achnatherum hymenoides Ampelodesmos mauritanica Piptochaetium avenaceum  Phaenosperma globosum Brachyelytrum aristosum  Bambusa bambosa | 23689  IGS |  | GAAT-----TA-TTCT  GAAT-----A--TTCT  GAAT-----TA-TTCT  GAAT-----TA-TTCT  GAAT-----TA-TTCT  GAAT-----TA-TTCT  GAAT-----TA-TTCT  AAATTATTCTACTTCT  GAAT-----TA-TTCT  GAAT-----TA-TTCT  GAAT-----TT-TTCT  GAAT-----TA-TTCT  GAAT-----TC-TTCT  GAAT-----TA-TTCT  GAAT-----TA-TTCT  GAAT-----TA-TTCT  GAAT-----TA-TTCT  GAAT-----TA-TTCT  GAAT-----TA-TTCT  GAAT-----TA-TTCT  GAAT-----TA-TTCT  GAAT-----TA-TTCT  GGATTATTCTA-TTCT  GGAT-----TA-TTCT  GGAT-----TA-TTCT  GGATTATTCTA-TTCT  GGAT-----TA-TTCT  GGAT-----TA-TTCT  GGAT-----TA-TTCT  GGAT-----TA-TTCT  GGAT-----TA-TTCT  GGAT-----TA-TTCT  GGAT-----TA-TTCT  GGAT-----TA-TTCT  GAAT-----TA-TTCT  GAAT-----TT-TTCT  GAAT-----TA-TTCT  GAAT-----TA-TTCT  GAAT-----TA-TTCT  GAAT-----TA-TTCT  GAAT-----TA-TTCT  GAAT-----TA-TTCT  GAAT-----TA-TTCT  GAAT-----TA-TTCT  GAAT-----TA-TTCT  AAAT-----TA-TTCT | Ssmp | Homoplasy |
| 37 | Schedonorus arundinaceus Schedonorus arundinaceus[C] Schedonorus pratensis  Festuca altissima  Festuca ovina  Lolium multiflorum  Lolium perenne  Dactylis glomerata  Helictochloa hookeri  Deschampsia antarctica  Poa palustris  Phleum alpinum  Briza sp  Puccinellia nuttalliana  Ammophila breviligulata  Agrostis stolonifera  Hierochloe odorata  Anthoxanthum odoratum  Phalaris arundinacea  Torreyochloa  Avena sativa  Trisetum cernuum  Aegilops cylindrica  Aegilops geniculata  Aegilops speltoides  Aegilops tauschii  Triticum aestivum  Triticum monococcum aegilopoides Triticum monococcum (modified) Triticum urartu (modified)  Hordeum jubatum  Hordeum vulgare  Hordeum vulgare spontaneum Secale cereale  Bromus vulgaris  Brachypodium distachyon  Diarrhena obovata  Melica mutica  Melica subulata  Oryzopsis asperifolia Achnatherum hymenoides Ampelodesmos mauritanica Piptochaetium avenaceum  Phaenosperma globosum Brachyelytrum aristosum  Bambusa bambosa | 23815  IGS |  | ACAA------ATTCTT-ATA  ACAAATTCTTAT-CTT-ATA  ACAA------ATTCTT-ATA  ACAA------ATTCTT-ATA  CCAA------ATTCTT-ATA  ACAA------ATTCTT-ATA  ACAA------ATTCTT-ATA  ACAA------ATTCTT-ATA  AAAA------ATTCTT-ATA  AGAA------ATTCTT-ATA  ACAA------ATTCTTAATA  ACAA------ATTATTAATA  ACAAATTCTTATTCTT-ATA  ACAA------ATTCTT-ATA  ACAAATTCTTATTCTT-ATA  ACAAATTCTTATTCTT-ATA  ACAAATTCTTATTCTT-ATA  ACAAATTATTATTCTT-ATA  ACAAATTCTTATTCTT-ATA  ACAAATTCTTATTCTT-ATA  ACAAATTCTTATTCTT-ATA  ACAAATTCTTATTCTT-ATA  ACAA------ATTCTT-ATA  ACAA------ATTCTT-ATA  ACAA------ATTCTT-ATA  ACAA------ATTCTT-ATA  ACAA------ATTCTT-ATA  ACAA------ATTCTT-ATA  ACAA------ATTCTT-ATA  ACAA------ATTCTT-ATA  ACAA------ATTCTT-ATA  ACAA------ATTCTT-ATA  ACAA------ATTCTT-ATA  ACAA------ATTCTT-ATA  ACAA------ATTCTT-ATA  ACAA------ATTATT-ATA  ACAA------ATTCTT-ATA  ACAA------ATTCTT-ATA  ACAA------ATTCTT-ATA  ACAA------ATTCTT-ATA  ACAA------ATTCTT-ATA  ACAA------ATTCTT-ATA  ACAA------ATTCTT-ATA  ACAA------ATTCTT-ATA  ACAA------ATTCTT-ATA  ACAA------ATTCTT-ATA | ssmp | Homoplasy |
| 38 | Schedonorus arundinaceus Schedonorus arundinaceus[C] Schedonorus pratensis  Festuca altissima  Festuca ovina  Lolium multiflorum  Lolium perenne  Dactylis glomerata  Helictochloa hookeri  Deschampsia antarctica  Poa palustris  Phleum alpinum  Briza sp  Puccinellia nuttalliana  Ammophila breviligulata  Agrostis stolonifera  Hierochloe odorata  Anthoxanthum odoratum  Phalaris arundinacea  Torreyochloa  Avena sativa  Trisetum cernuum  Aegilops cylindrica  Aegilops geniculata  Aegilops speltoides  Aegilops tauschii  Triticum aestivum  Triticum monococcum aegilopoides Triticum monococcum (modified) Triticum urartu (modified)  Hordeum jubatum  Hordeum vulgare  Hordeum vulgare spontaneum Secale cereale  Bromus vulgaris  Brachypodium distachyon  Diarrhena obovata  Melica mutica  Melica subulata  Oryzopsis asperifolia Achnatherum hymenoides Ampelodesmos mauritanica Piptochaetium avenaceum  Phaenosperma globosum Brachyelytrum aristosum  Bambusa bambosa | 23941  IGS |  | GATGTCC-------TGAATAGA  GATGTCC-------TGAATAAA  GATGTCC-------TGAATAGA  GATGTCC-------TGAATAGA  GATGTCC-------TGAATAGA  GATGTCC-------TGAATAGA  GATGTCC-------TGAATAGA  GATGTCC-------TGAATAGA  TATGTCC-------TGAATAGA  GATGTCC-------TGAATAGA  GATGTCT-------TGAATAGA  GATGTCC-------TGAATAGA  GATGTCCTAAATAATGAATAGA  GATGTCC-------TGAATAGA  GATGTCCTAAATACTGAATAGA  GATGTCCTAAATACTGAATAGA  GATGTCCTAAATACTGAATAGA  GATGCCCTAAATACTGAATAGA  GATGTCCTAAATACTGAATAGG  GATGTCCTAAATACTGAATAGA  GATGTCCTAAATACTGAATAGA  GATGTCCTAAATACTGAATAGA  GATGTCC-------TGAATAGA  GATGTCC-------TGAATAGA  GATGTCC-------TGAATAGA  GATGTCC-------TGAATAGA  GATGTCC-------TGAATAGA  GATGTCC-------TGAATAGA  GATGTCC-------TGAATAGA  GATGTCC-------TGAATAGA  GATGTCC-------TGAATAGA  GATGTCC-------TGAATAGA  GATGTCC-------TGAATAGA  GATGTCC-------TGAATAGA  GATGTCC-------TGAATAGA  GATGTCC-------TGAATATA  GATGTCC-------TGAATAGA  GGTGTTC-------TGAATAGA  GGTGTTC-------TGAATAGA  GATGTCC-------TGAATAGA  GATGTCC-------TGAATAGA  GATGTCC-------TGAATAGA  GATGTCC-------TGAATAGA  GATGTCC-------TGAATAGA  AATGTCC-------TGAATAGA  GATGTCC-------TGAATGGA |  | Poeae clade 1 |
| 39 | Schedonorus arundinaceus Schedonorus arundinaceus[C] Schedonorus pratensis  Festuca altissima  Festuca ovina  Lolium multiflorum  Lolium perenne  Dactylis glomerata  Helictochloa hookeri  Deschampsia antarctica  Poa palustris  Phleum alpinum  Briza sp  Puccinellia nuttalliana  Ammophila breviligulata  Agrostis stolonifera  Hierochloe odorata  Anthoxanthum odoratum  Phalaris arundinacea  Torreyochloa  Avena sativa  Trisetum cernuum  Aegilops cylindrica  Aegilops geniculata  Aegilops speltoides  Aegilops tauschii  Triticum aestivum  Triticum monococcum aegilopoides Triticum monococcum (modified) Triticum urartu (modified)  Hordeum jubatum  Hordeum vulgare  Hordeum vulgare spontaneum Secale cereale  Bromus vulgaris  Brachypodium distachyon  Diarrhena obovata  Melica mutica  Melica subulata  Oryzopsis asperifolia Achnatherum hymenoides Ampelodesmos mauritanica Piptochaetium avenaceum  Phaenosperma globosum Brachyelytrum aristosum  Bambusa bambosa | 23987  IGS |  | GATTCCTTTTGTTTGTTA  TCTTCCG----TTTGTTA  GATTCCTTTTGTTTGTTA  TATTCCT----TTTGTTA  TATTCCT----TTTGTTA  GATTCCTTTTGTTTGTTA  GATTCCTTTTGTTTGTTA  TATTCCT----TTTGTTA  TATTCCT----TTTTTTA  TATTCCT----TTTGTTA  TATTCCT----TTTGTTA  TATTCCT----TTTGTTA  TATTCCT----TTTGTTA  TATTCCT----TTTGTTA  TATTCCT----TTTGTTA  TATTCCT----TTTGTTA  TATTCCT----TTTGTTA  TTTTCCT----TTTGTTA  TATTCCT----TTTGTTA  TATTCCT----TTTGTTA  GATTCCT----TTTGTTA  TATTCCT----TTTGTTA  TATTCCT----TTTGTTA  TATTCCT----TTTGTTA  TATTCCT----TTTGTTA  TATTCCT----TTTGTTA  TATTCCT----TTTGTTA  TATTCCT----TTTGTTA  TATTCCT----TTTGTTA  TATTCCT----TTTGTTA  TATTCCT----TTTGTTA  TATTCCT----TTTGTTA  TATTCCT----TTTGTTA  TATTCCT----TTTGTTA  TATTCCT----TTTGTTA  TATTCCT----TTTGTTA  TATTCCT----TTTGTTA  TATTCCT----TTTGTTA  TATTCCT----TTTGTTA  TATTCCT----TTTGTTA  TATTCCT----TTTGTTA  TATTCCT----TTTGTTA  TATTCCT----TTTGTTA  TATTCCT----TTTGTTA  TCTTCCT----TTTGTTA  TCTTCCT----TTTGTTA | ssmp | Homoplasy |
| 40 | Schedonorus arundinaceus Schedonorus arundinaceus[C] Schedonorus pratensis  Festuca altissima  Festuca ovina  Lolium multiflorum  Lolium perenne  Dactylis glomerata  Helictochloa hookeri  Deschampsia antarctica  Poa palustris  Phleum alpinum  Briza sp  Puccinellia nuttalliana  Ammophila breviligulata  Agrostis stolonifera  Hierochloe odorata  Anthoxanthum odoratum  Phalaris arundinacea  Torreyochloa  Avena sativa  Trisetum cernuum  Aegilops cylindrica  Aegilops geniculata  Aegilops speltoides  Aegilops tauschii  Triticum aestivum  Triticum monococcum aegilopoides Triticum monococcum (modified) Triticum urartu (modified)  Hordeum jubatum  Hordeum vulgare  Hordeum vulgare spontaneum Secale cereale  Bromus vulgaris  Brachypodium distachyon  Diarrhena obovata  Melica mutica  Melica subulata  Oryzopsis asperifolia Achnatherum hymenoides Ampelodesmos mauritanica Piptochaetium avenaceum  Phaenosperma globosum Brachyelytrum aristosum  Bambusa bambosa | 24581  IGS |  | AAATCGAA--CCAA--  GAATCGAA--TCAA--  AAATCGAA--CCAA--  AAATCGAA--CCAA--  AAATCGAA--CCAA--  AAATCGAA--CCAA--  AAATCGAA--CCAA--  AAATCGAA--CCAA--  AAATCGAA--CCAA--  AAATCGAA--CCAA--  AAATCGAA--TCAA--  AAATCGAA--TCCA--  AAATCGAA--TCAA--  AAATCGAA--CCAA--  AAATCGAA--TCAA--  AAATCGAA--TCAA--  AAATCGAA--TCAA--  AAATCGAA--GCAA--  AAATCGAA--TCAA--  AAATCGAA--TCAA--  AAATCGAA--TCAA--  AAATCGAA--TCAA--  AAATCGAATCTCAATT  AAATCGAATCTCAATT  AAATCGAATCTCAATT  AAATCGAATCTCAATT  AAATCGAATCTCAATT  AAATCGAATCTCAATT  AAATCGAATCTCAATT  AAATCGAATCTCAATT  AAATCGAA--TCAATT  AAATCGAA--TCAATT  AAATCGAA--TCAATT  AAATCGAATCTCAATT  AAATCGAA--TCAATT  AAATAGGA--TCAATT  AAATCGAA--TCAATT  GAATCAAA--TCAATT  GAATAAAA--TCAATT  AAATCGAA--TCAATT  AAATCGAA--TCAATT  AAATCGAA--TCAATT  AAATCGAA--TCAATT  ---TCGAA--TCAATT  GAATTGAA--TCAATT  GAATCGAA--TCAATT | ssmp | Aegilops+Triticum+Secale |
| 41 | Schedonorus arundinaceus Schedonorus arundinaceus[C] Schedonorus pratensis  Festuca altissima  Festuca ovina  Lolium multiflorum  Lolium perenne  Dactylis glomerata  Helictochloa hookeri  Deschampsia antarctica  Poa palustris  Phleum alpinum  Briza sp  Puccinellia nuttalliana  Ammophila breviligulata  Agrostis stolonifera  Hierochloe odorata  Anthoxanthum odoratum  Phalaris arundinacea  Torreyochloa  Avena sativa  Trisetum cernuum  Aegilops cylindrica  Aegilops geniculata  Aegilops speltoides  Aegilops tauschii  Triticum aestivum  Triticum monococcum aegilopoides Triticum monococcum (modified) Triticum urartu (modified)  Hordeum jubatum  Hordeum vulgare  Hordeum vulgare spontaneum Secale cereale  Bromus vulgaris  Brachypodium distachyon  Diarrhena obovata  Melica mutica  Melica subulata  Oryzopsis asperifolia Achnatherum hymenoides Ampelodesmos mauritanica Piptochaetium avenaceum  Phaenosperma globosum Brachyelytrum aristosum  Bambusa bambosa | 24718  IGS |  | CAAAACA------AACCCTA  CAAAGCA------AACCCTA  CAAAACA------AACCCTA  CAAAACA------AACCCTA  CAAAACA------AACCCTA  CAAAACA------AACCCTA  CAAAACA------AACCCTA  CAAAGCA------AACCCTA  CAAAACA------AACCCTA  CAAAACA------AACCCTA  CAAAACA------AACCCTA  CAAAACA------AACCCTA  CAAAACA------AACCCCA  CAAAACA------AACCCTA  CAAAGCA------AACCCTA  CAAAGCA------AACCCTA  CAAAACA------AACCCTA  CAAAACA------AACCCTA  TAAAACA------AACCCTA  CAAAACA------AACCCTA  CAAAGCA------AACCCTA  CAAAGCA------AACCCTA  CAAAACAAACCCTAACCCTA  CAAAACAAACCCTAACCCTA  CAAAACAAATCCTAACCCTA  CAAAACAAACCCTAACCCTA  CAAAACAAATCCTAACCCTA  CAAAACAAACCCTAACCCTA  CAAAACAAACCCTAACCCTA  CAAAACAAACCCTAACCCTA  CAAAACAAACCCTAACCCTA  CAAAACAAACCCTAACCCTA  CAAAACAAACCCTAACCCTA  CAAAACAAACCCTAACCCTA  CAAAACA------AACCCTA  CAAAACA------AACCCTA  CAAAACA------AACCCTA  AAAAATA------AACCCTA  AAAAATA------AACCCTA  CAAAACA------AACCCTA  CAAAACA------AACCCTA  CAAAACA------AACCCTA  CAAAATA------AACCCTA  CAAAACA------AACCCTA  CAAAACA------AATCCTA  CAAAACA------AACCCTA | Ssmp | Triticeae |
| 42 | Schedonorus arundinaceus Schedonorus arundinaceus[C] Schedonorus pratensis  Festuca altissima  Festuca ovina  Lolium multiflorum  Lolium perenne  Dactylis glomerata  Helictochloa hookeri  Deschampsia antarctica  Poa palustris  Phleum alpinum  Briza sp  Puccinellia nuttalliana  Ammophila breviligulata  Agrostis stolonifera  Hierochloe odorata  Anthoxanthum odoratum  Phalaris arundinacea  Torreyochloa  Avena sativa  Trisetum cernuum  Aegilops cylindrica  Aegilops geniculata  Aegilops speltoides  Aegilops tauschii  Triticum aestivum  Triticum monococcum aegilopoides Triticum monococcum (modified) Triticum urartu (modified)  Hordeum jubatum  Hordeum vulgare  Hordeum vulgare spontaneum Secale cereale  Bromus vulgaris  Brachypodium distachyon  Diarrhena obovata  Melica mutica  Melica subulata  Oryzopsis asperifolia Achnatherum hymenoides Ampelodesmos mauritanica Piptochaetium avenaceum  Phaenosperma globosum Brachyelytrum aristosum  Bambusa bambosa | 25431  IGS |  | ACGACTCTTAAACTTAAATA  ACGACTCTTAAACTTAAATA  ACGACTCTTAAACTTAAATA  ACGACT------CTTAAATA  ACGACT------CTTAAATA  ACGACTCTTAAACTTAAATA  ACGACTCTTAAACTTAAATA  ACGATT------CTTAAATA  ACGACT------CTTAAATA  ACGACT------CTTAAATA  ACTACT------CTTAAATA  ACTACT------CTTAAATA  ACGATT------CTTAAATA  ACGACT------CTTAAATA  ACGACT------CTTAAATA  ACGACT------CTTAAATA  ACGACT------CTTAAATA  ACGGCT------CTTAAATA  ACGACT------CTTAAATA  ACGACT------CTTAAAGA  ACGACT------CTTAAATA  ACGACT------CTTAAATA  ACTACT------CTTAAATC  ACTACT------CTTAAATC  ACTACT------CTTAAATC  ACTACT------CTTAAATC  ACTACT------CTTAAATC  ACTACT------CTTAAATC  ACTACT------CTTAAATC  ACTACT------CTTAAATC  ACTACT------CTTAAATC  ACTACT------CTTAAATC  ACTACT------CTTAAATC  ACTACT------CTTAAATC  ACGACT------CTTAAATC  ACGACT------CTTAAATG  ACGACT------CTTAAATC  ACGACT------CTTAAATT  ACGACT------CTTAAATT  ACGACT------CTTAAATC  ACGACT------CTTAAATC  ACAACT------CTTAAATC  ACGATT------CTTAAATC  ACGACT------CTTAAATC  ACGACT------CTTAAATC  ACGACT------CTTAAATC | Ssmp | Lolium+Schedonorus |
| 43 | Schedonorus arundinaceus Schedonorus arundinaceus[C] Schedonorus pratensis  Festuca altissima  Festuca ovina  Lolium multiflorum  Lolium perenne  Dactylis glomerata  Helictochloa hookeri  Deschampsia antarctica  Poa palustris  Phleum alpinum  Briza sp  Puccinellia nuttalliana  Ammophila breviligulata  Agrostis stolonifera  Hierochloe odorata  Anthoxanthum odoratum  Phalaris arundinacea  Torreyochloa  Avena sativa  Trisetum cernuum  Aegilops cylindrica  Aegilops geniculata  Aegilops speltoides  Aegilops tauschii  Triticum aestivum  Triticum monococcum aegilopoides Triticum monococcum (modified) Triticum urartu (modified)  Hordeum jubatum  Hordeum vulgare  Hordeum vulgare spontaneum Secale cereale  Bromus vulgaris  Brachypodium distachyon  Diarrhena obovata  Melica mutica  Melica subulata  Oryzopsis asperifolia Achnatherum hymenoides Ampelodesmos mauritanica Piptochaetium avenaceum  Phaenosperma globosum Brachyelytrum aristosum  Bambusa bambosa | 25720  IGS |  | AAAGC-----ATTAAAT  AAGAC-----ATTAAAT  AAAGC-----ATTAAAT  AAAGC-----ATTAAAT  AAAGC-----ATTAAAT  AAAGC-----ATTAAAT  AAAGC-----ATTAAAT  AAAGC-----ATTAAAT  AAAGC-----ATTAAAT  AAAGC-----ATTAAAT  AAAGC-----ATTAAAT  AAAGC-----ATTAAAT  AAAGC-----ATTAAAT  AAAGC-----ATTAAAT  AAAGC-----ATTAAAT  AAAGC-----ATTAAAT  AAAGC-----ATTAAAT  ACAGC-----ATTAAAT  AAAGC-----ATTAAAT  AAAGC-----ATTAAAT  AAAGC-----ATTAAAT  AAAGC-----ATTAAAT  AAAGTATTAAATTAAAT  AAAGTATTAAATTAAAT  AAAGTATTAAATTAAAT  AAAGTATTAAATTAAAT  AAAGTATTAAATTAAAT  AAAGTATTAAATTAAAT  AAAGTATTAAATTAAAT  AAAGTATTAAATTAAAT  AAAGTCTTAAATTAAAT  AAAGTATTAAATTAAAT  AAAGTATTAAATTAAAT  AAAGTATTAAATTAAAT  AAAGCATTAAATTAAAT  AAAGC-----ATTAAAT  AAAGC-----ATTAAAT  AAAGC-----ATTAAAT  AAAGC-----ATTAAAT  AAAGC-----ATTAAAT  AAAGC-----ATTAAAT  AAAGC-----ATTAAAT  AAAGC-----ATTAAAT  AAAGC-----ATTAAAT  AAAGCATTAAATAAAAT  ATTGC-----ATTAAAT | ssmp | Bromeae+Triticeae |
| 44 | Schedonorus arundinaceus Schedonorus arundinaceus[C] Schedonorus pratensis  Festuca altissima  Festuca ovina  Lolium multiflorum  Lolium perenne  Dactylis glomerata  Helictochloa hookeri  Deschampsia antarctica  Poa palustris  Phleum alpinum  Briza sp  Puccinellia nuttalliana  Ammophila breviligulata  Agrostis stolonifera  Hierochloe odorata  Anthoxanthum odoratum  Phalaris arundinacea  Torreyochloa  Avena sativa  Trisetum cernuum  Aegilops cylindrica  Aegilops geniculata  Aegilops speltoides  Aegilops tauschii  Triticum aestivum  Triticum monococcum aegilopoides Triticum monococcum (modified) Triticum urartu (modified)  Hordeum jubatum  Hordeum vulgare  Hordeum vulgare spontaneum Secale cereale  Bromus vulgaris  Brachypodium distachyon  Diarrhena obovata  Melica mutica  Melica subulata  Oryzopsis asperifolia Achnatherum hymenoides Ampelodesmos mauritanica Piptochaetium avenaceum  Phaenosperma globosum Brachyelytrum aristosum  Bambusa bambosa | 26333  IGS |  | CCATTTGTA-----CCCCTGGC  CCATTTGTA-----CCCCTGCC  CCATTTGTA-----CCCCTGGC  CCATTTGTA-----CCCCTGGC  CTAGTTGTA-----CCCCTGGC  CCATTTGTA-----CCCCTGGC  CCATTTGTA-----CCCCTGGC  CTATTTGTA-----CCCCTGGC  CCATTTGTA-----CCCCTGGC  CCATTTGTA-----CCCCTGGC  CCATTTGTA-----CCCCTGGC  CCATTTGTA-----CCCCTGGC  CATTTTGTACCCCTCCCCTGGC  CCATTTGTA-----CCCCCGGC  CCATTTGTACCCCTCCCCTGGC  CCATTTGTGCCCCTCCCCTGGC  CCATTTGTACCCCTCCCCTAGC  CCATTTGTACCCCTCCCCTGGC  CCATTTGTACCCCTCCCCTAGC  CCATTTGTACCCCTCCCCTGGC  CCATTTGTACCCCTCCCCTGGA  CCATCTGTACCCCTCCCCTGGA  TCATTTGTA-----CCCCTGGC  TCATTTGTA-----CCCCTGGC  TCATTTGTA-----CCCCTGGC  TCATTTGTA-----CCCCTGGC  TCATTTGTA-----CCCCTGGC  TCATTTGTA-----CCCCTGGC  TCATTTGTA-----CCCCTGGC  TCATTTGTA-----CCCCTGGC  TCATTTGTA-----CCCCTGGT  TCATTTGTA-----CCCCTGGT  TCATTTGTA-----CCCCTGGT  TCATTTGTA-----CCCCTGGC  TCATTTGTA-----CCCCTGGC  CCATTTGTA-----CCCCTGGC  CCATTTGTA-----CCCCTGGC  CCATTTGTA-----CCCTCGGC  CCATTTGTA-----CCCTCGGC  CCATTTGTA-----CCCCTGGC  CCAGTTGTA-----CCCCTGGC  CCATTTGTA-----CCCCTGGC  CCATTTGTA-----CCCCTGGC  CCATTTGTA-----CCCCTGGC  CCATTTGTA-----CCCCTGGA  CGATTTGTA-----CCCCTGGC | ssmp | Poeae clade 1 |
| 45 | Schedonorus arundinaceus Schedonorus arundinaceus[C] Schedonorus pratensis  Festuca altissima  Festuca ovina  Lolium multiflorum  Lolium perenne  Dactylis glomerata  Helictochloa hookeri  Deschampsia antarctica  Poa palustris  Phleum alpinum  Briza sp  Puccinellia nuttalliana  Ammophila breviligulata  Agrostis stolonifera  Hierochloe odorata  Anthoxanthum odoratum  Phalaris arundinacea  Torreyochloa  Avena sativa  Trisetum cernuum  Aegilops cylindrica  Aegilops geniculata  Aegilops speltoides  Aegilops tauschii  Triticum aestivum  Triticum monococcum aegilopoides Triticum monococcum (modified) Triticum urartu (modified)  Hordeum jubatum  Hordeum vulgare  Hordeum vulgare spontaneum Secale cereale  Bromus vulgaris  Brachypodium distachyon  Diarrhena obovata  Melica mutica  Melica subulata  Oryzopsis asperifolia Achnatherum hymenoides Ampelodesmos mauritanica Piptochaetium avenaceum  Phaenosperma globosum Brachyelytrum aristosum  Bambusa bambosa | 29793  rpoB | rpoB | GAGGAAG-----TTTGATCG  GAGGATG-----TTGGATCG  GAGGAAG-----TTTGATCG  GAGGAAG-----TTTGATCG  GAGGAAG-----TTTGATCG  GAGGAAG-----TTTGATCG  GAGGAAG-----TTTGATCG  GAGGAAG-----TTTGATCG  GAGGAAG-----TTTGATCG  GAGGAAG-----TTTGATCG  GAGGAAG-----TTTGATCG  GAGGAAG-----TTTGATCG  GAGGAAGTTTGATTTGATCG  GAGGAAG-----TTTGATCG  GAGGAAGTTTGATTTGATCG  GAAGAAGTTTGATTTGATCG  GAGGAAGTTTGATTTGATCG  GAGGAAGTTTGATTTGATCG  GAGGAAGTTTGATTTGATCG  GAGGAAGTTTGATTTGATCG  GAGGAAGTTTGATTTGATCG  GAGGAAGTTTGATTTGATCG  GAGGAAG-----TTTGATCG  GAGGAAG-----TTTGATCG  GAGGAAG-----TTTGATCG  GAGGAAG-----TTTGATCG  GAGGAAG-----TTTGATCG  GAGGAAG-----TTTGATCG  GAGGAAG-----TTTGATCG  GAGGAAG-----TTTGATCG  GAGGAAG-----TTTGATCG  GAGGAAG-----TTTGATCG  GAGGAAG-----TTTGATCG  GAGGAAG-----TTTGATCG  GAGGAAG-----TTTGATCG  GAGGAAA-----TTTGATCG  GAGGAAG-----TTTGATCG  GAGGAAG-----TTTGATCG  GAGGAAG-----TTTGATCG  GAGGAAG-----TTTGATCG  GAGGAAG-----TTTGATCG  GAGGAAG-----TTTGATCG  GAGGAAG-----TTTGATCG  GAGGAAG-----TTTGATCG  GAGGAAG-----TTTGATCA  GAGGAAG-----TTTGATCG | ssmp | Poeae clade 1 |
| 46 | Schedonorus arundinaceus Schedonorus arundinaceus[C] Schedonorus pratensis  Festuca altissima  Festuca ovina  Lolium multiflorum  Lolium perenne  Dactylis glomerata  Helictochloa hookeri  Deschampsia antarctica  Poa palustris  Phleum alpinum  Briza sp  Puccinellia nuttalliana  Ammophila breviligulata  Agrostis stolonifera  Hierochloe odorata  Anthoxanthum odoratum  Phalaris arundinacea  Torreyochloa  Avena sativa  Trisetum cernuum  Aegilops cylindrica  Aegilops geniculata  Aegilops speltoides  Aegilops tauschii  Triticum aestivum  Triticum monococcum aegilopoides Triticum monococcum (modified) Triticum urartu (modified)  Hordeum jubatum  Hordeum vulgare  Hordeum vulgare spontaneum Secale cereale  Bromus vulgaris  Brachypodium distachyon  Diarrhena obovata  Melica mutica  Melica subulata  Oryzopsis asperifolia Achnatherum hymenoides Ampelodesmos mauritanica Piptochaetium avenaceum  Phaenosperma globosum Brachyelytrum aristosum  Bambusa bambosa | 32064  IGS |  | CAGAT-TGAGATTAAGGAA  CAGATGTGAGATTGAGAAA  CAGAT-TGAGATTAAGGAA  CAGAT-TGAGATTAAGGAA  CAAAT-TGAGATTAAAGAA  CAGAT-TGAGATTAAGGAA  CAGAT-TGAGATTAAGGAA  CAGAT-TGAGATTAAGGA-  CAGAT-TGAGATTAAGGAA  CAGAT-TGAGATTAAGGAA  CAGAT-TGAGATTAAGGAA  CAGAT-TGAGATTAAGGAA  CAGAT-TGAGATTAAGGAA  CAGAT-TGAGATTAAGGAA  CAGAT-TGAGATTAAGGAA  CAGAT-TGAGATTAAGGAA  CAGAT-TGAGATTAAGGAA  CAGAT-TGAGATTAAGGAA  CAGAT-TGAGATTAAGGAA  CAGAT-TGAGATTAAGGAA  CAGAT-TGAGATTAAGTAA  CAGAT-TGAGATTAAGGAA  CAGAT-TGAGATTGAGGC-  CAGAT-TGAGATTGAGGCA  CAGAT-TGAGATTGAGGCA  CAGAT-TGAGATTGAGGC-  CAGAT-TGAGATTGAGGCA  CAGAT-TGAGATTGAGGCA  CAGAT-TGAGATTGAGGCA  CAGAT-TGAGATTGAGGCA  CAGAT-TGAGATTGAGGCA  CAGAT-TGAGATTGAGGCA  CAGAT-TGAGATTGAGGCA  CAGAT-TGAGATTGAGGCA  CAGAT-TGAGATTGAGGAA  CAGAT-TGAGATTGAGAAA  CAGAT-TGAGATTGAGGAA  CAGAT-------TGAGGAA  CAGAT-------TGAGGAA  CAGAT-TGAGATTGAGGAA  CAGAT-TGAGATTGAGGAA  CAGAT-TGAGATTGAGGAA  CAGAT-TGAGATTGAGGAA  CAGAT-TGAGATTGAGGAA  CAGAT-------TGAGGAA  CAGAT-TGAGATTAAGGAA |  | Homoplasy |
| 47 | Schedonorus arundinaceus  Schedonorus arundinaceus[C]  Schedonorus pratensis  Festuca altissima  Festuca ovina  Lolium multiflorum  Lolium perenne  Dactylis glomerata  Helictochloa hookeri  Deschampsia antarctica  Poa palustris  Phleum alpinum  Briza sp  Puccinellia nuttalliana  Ammophila breviligulata  Agrostis stolonifera  Hierochloe odorata  Anthoxanthum odoratum  Phalaris arundinacea  Torreyochloa  Avena sativa  Trisetum cernuum  Aegilops cylindrica  Aegilops geniculata  Aegilops speltoides  Aegilops tauschii  Triticum aestivum  Triticum monococcum aegilopoides  Triticum monococcum (modified)  Triticum urartu (modified)  Hordeum jubatum  Hordeum vulgare  Hordeum vulgare spontaneum  Secale cereale  Bromus vulgaris  Brachypodium distachyon  Diarrhena obovata  Melica mutica  Melica subulata  Oryzopsis asperifolia  Achnatherum hymenoides  Ampelodesmos mauritanica  Piptochaetium avenaceum  Phaenosperma globosum  Brachyelytrum aristosum  Bambusa bambosa | 33694  rpoC2 | rpoC2 | TGAATACTTA---------------------------------------------------------------------------------TGGTAAAAAAGATAGGGAAA  TGAATACTTATTCTTTTTCTGTTCATGGAAGATATATATTTGACCTATCAATGGCTAATGATCAAGTAAGCC---------TGGAGACTTTTGGTAAAAAAGATAGGGAAA  TGAATACTTA---------------------------------------------------------------------------------TGGTAAAAAAGATAGGGAAA  TGAATACTTA---------------------------------------------------------------------------------TGGTAAAAAAGATAGGGAAA  TGAATACTTA---------------------------------------------------------------------------------TGGTAAAAAAGATAAGGAAA  TGAATACTTA---------------------------------------------------------------------------------TGGTAAAAAAGATAGGGAAA  TGAATACTTA---------------------------------------------------------------------------------TGGTAAAAAAGATAGGGAAA  TGAATACTTA---------------------------------------------------------------------------------TGGTAAAAAAAATAGGGAAA  TGAATACTTA---------------------------------------------------------------------------------TCGTAAAAAAGATAGGGAAA  TGAATACTTA---------------------------------------------------------------------------------TGGTAAAAAAGATAGGGAAA  TGAATACTTA---------------------------------------------------------------------------------TGGTAAAAAAGATAGGGAAA  TGAATACTTA---------------------------------------------------------------------------------TGGTAAAAAAGATAGGGAAA  TGAATACTTA---------------------------------------------------------------------------------TGGTAAAAAAGATAAGGAAA  TGAATACTTA---------------------------------------------------------------------------------TGGTAAAAAAGATAGGGAAA  TGAATACTTA---------------------------------------------------------------------------------TGGTAAAAAAGATAGGGAAA  TGAATACTTA---------------------------------------------------------------------------------TGGTAAAAAAGATAGGGAAA  TGAATACTTA---------------------------------------------------------------------------------TGGTAAAAAAGATAGGGAAA  TGAATACTTA---------------------------------------------------------------------------------TGGTAAAAAAGATAGGGAAA  TGAATACTTA---------------------------------------------------------------------------------TGGTAAAAAAGATAGGGAAA  TGAATACTTA---------------------------------------------------------------------------------TGGTAAAAAAGATAGGGAAA  TGAATACTTA---------------------------------------------------------------------------------TGGTAAAAAAGATAGGGAAA  TGAATACTTA---------------------------------------------------------------------------------TGGTAAAAAAGACAGGGAAA  TGAATACTTA---------------------------------------------------------------------------------TGGTAAAAAAGATAGGGAAA  TGAATACTTA---------------------------------------------------------------------------------TGGTAAAAAAGATAGGGAAA  TGAATACTTA---------------------------------------------------------------------------------TGGTAAAAAAGATAGGGAAA  TGAATACTTA---------------------------------------------------------------------------------TGGTAAAAAAGATAGGGAAA  TGAATACTTA---------------------------------------------------------------------------------TGGTAAAAAAGATAGGGAAA  TGAATACTTA---------------------------------------------------------------------------------TGGTAAAAAAGATAGGGAAA  TGAATACTTA---------------------------------------------------------------------------------TGGTAAAAAAGATAGGGAAA  TGAATACTTA---------------------------------------------------------------------------------TGGTAAAAAAGATAGGGAAA  TGAATACTTA---------------------------------------------------------------------------------TGGTAAAAAAGATAGGGAAA  TGAATACTTA---------------------------------------------------------------------------------TGGTAAAAAAGATAGGGAAA  TGAATACTTA---------------------------------------------------------------------------------TGGTAAAAAAGATAGGGAAA  TGAATACTTA---------------------------------------------------------------------------------TGGTAAAAAAGATAGGGAAA  TTAATACTTA---------------------------------------------------------------------------------TGGTAAAAAAGATAGGGAAA  TGAATACTTA---------------------------------------------------------------------------------TGGTAAAAAAGATAGGGAAA  TGAATACTTA---------------------------------------------------------------------------------TGGTAAAAAAGATAGGGAAA  TGAATACTTATTCTTTTTCTGTTGATGGAAGATATACATTTGACCTCTCAATGGCTAATGATCAAGTAAGACATAGACTGTTGGATACTTTTGATAAAAAAGATAGGGAAA  TGAATACTTATTCTTTTTCTGTTGATGGAAGATATACATTTGACCTCTCAATGGCTAATGATCAAGTAAGACATAGACTGTTGGATACTTTTGATAAAAAAGATAGGGAAA  TGAATACTTATTCTTTTTCTGTTGACGGAAGATATATCTTTGACCTCTCAATGGCTAATGATCAAGTAAGACATAGACTGTTGGATACTTTTGGTAAAAAAGATAGGGAAA  TCAATACTTATTCTTTTTCTGTTGACGGAAGATATATCTTTGACCTCTCAATGGCTAATGATCAAGTAAGACATAGACTGTTGGATACTTTTGGTAAAAAAGATAGGGAAA  TGAATACTTATTCTTTTTCTGTTGACGGAAGATATATCTTTGACCTCTCAATGGCTAATGATCAAGTAAGACATAGACTGTTGGATACTTTTGGTAAAAAAGATAGGGAAA  TGAATACTTATTCTTTTTCTGTTGACGGAAGATATATCTTTGACCTCTCAATGGCTAATGATCAAGTAAGACATAGACTGTTGGATACTTTTGGTAAAAAAGATAGGGAAA  TGAATACTTATTCCTTTTCTGTTGACGAAAGATATATCTTTGACCTCTCAATGGCTAATGATCAAGTAAGACATAGACTGTTGGATACTTTTGGTAAAAAAGATAGGAAAA  TGAATACTTATTCTTTTGCTGTTGACGAAAGATATATCTTTGACCTCTCAATGGCTAATGATCAAGTAAGCCATAAACTGTTGGATACTTTTGGTAAAAAAGATAGGGAAA  TGAATACTTATTCTTTTTCTGTTGACGGAAGATATATCTTTGACCTCTCAATGGCTAATGATCAAGTAAGACATAGACTGTTGGATACTTTTGGTAAAAAAGATAGGGAAA |  | Homoplasy |
| 48 | Schedonorus arundinaceus Schedonorus arundinaceus[C] Schedonorus pratensis  Festuca altissima  Festuca ovina  Lolium multiflorum  Lolium perenne  Dactylis glomerata  Helictochloa hookeri  Deschampsia antarctica  Poa palustris  Phleum alpinum  Briza sp  Puccinellia nuttalliana  Ammophila breviligulata  Agrostis stolonifera  Hierochloe odorata  Anthoxanthum odoratum  Phalaris arundinacea  Torreyochloa  Avena sativa  Trisetum cernuum  Aegilops cylindrica  Aegilops geniculata  Aegilops speltoides  Aegilops tauschii  Triticum aestivum  Triticum monococcum aegilopoides Triticum monococcum (modified) Triticum urartu (modified)  Hordeum jubatum  Hordeum vulgare  Hordeum vulgare spontaneum Secale cereale  Bromus vulgaris  Brachypodium distachyon  Diarrhena obovata  Melica mutica  Melica subulata  Oryzopsis asperifolia Achnatherum hymenoides Ampelodesmos mauritanica Piptochaetium avenaceum  Phaenosperma globosum Brachyelytrum aristosum  Bambusa bambosa | 34260  rpoC2 | rpoC2 | ACCCT---AGAAGA  AGCCC---AGAGAA  ACCCT---AGAAGA  ACCCT---AGAAGA  ACCCT---AGAAGA  ACCCT---AGAAGA  ACCCT---AGAAGA  ACCCT---AGAAGA  ACCCT---AGAAGA  ACCCT---AGAAGA  ATCCT---AGAAGA  ACCCT---AGAAGA  ACCCT---AGAAGA  ACCCT---AGAAGA  ACCCT---AGAAGA  ACCCT---AGAAGA  ACCCT---AGAAGA  ACCCT---AGAAGA  AACCT---ACAAGA  AACTT---ACAAGA  ACCCT---AGAAGA  ACCCT---AGAAGA  ACCCT---AGAAGA  ACCCT---AGAAGA  ACCCT---AGAAGA  ACCCT---AGAAGA  ACCCT---AGAAGA  ACCCT---AGAAGA  ACCCT---AGAAGA  ACCCT---AGAAGA  ACCCT---AGAAGA  ACCCT---AGAACA  ACCCT---AGAACA  ACCCT---AGAAGA  ACCCT---AGAAGA  ACCCT---AGAAGA  GCCCT---AGAAGA  ACCCTAGAAGAAGA  ACCCTAGAAGAAGA  ACCCT---AGAAGA  ACCCT---AGAAGA  ACCCT---AGAAGA  ACCCT---AGAAGA  ACCTT---AGAAGA  ACCCT---AGAAGA  AGCCC---AGAGAA |  | Melica |
| 49 | Schedonorus arundinaceus Schedonorus arundinaceus[C] Schedonorus pratensis  Festuca altissima  Festuca ovina  Lolium multiflorum  Lolium perenne  Dactylis glomerata  Helictochloa hookeri  Deschampsia antarctica  Poa palustris  Phleum alpinum  Briza sp  Puccinellia nuttalliana  Ammophila breviligulata  Agrostis stolonifera  Hierochloe odorata  Anthoxanthum odoratum  Phalaris arundinacea  Torreyochloa  Avena sativa  Trisetum cernuum  Aegilops cylindrica  Aegilops geniculata  Aegilops speltoides  Aegilops tauschii  Triticum aestivum  Triticum monococcum aegilopoides Triticum monococcum (modified) Triticum urartu (modified)  Hordeum jubatum  Hordeum vulgare  Hordeum vulgare spontaneum Secale cereale  Bromus vulgaris  Brachypodium distachyon  Diarrhena obovata  Melica mutica  Melica subulata  Oryzopsis asperifolia Achnatherum hymenoides Ampelodesmos mauritanica Piptochaetium avenaceum  Phaenosperma globosum Brachyelytrum aristosum  Bambusa bambosa | 38132  IGS |  | ATCAC-----TTAC  ATCAC-----TTAC  ATCAC-----TTAC  ATCAC-----TTAC  ATCAC-----TTAC  ATCAC-----TTAC  ATCAC-----TTAC  ATCAC-----TTAC  ATCAC-----TTAC  ATCAC-----TTAC  ATCAC-----TTAC  ATCAC-----TTAC  ATCAC-----TTAC  ATCTC-----TTAC  ATCAC-----TTAC  ATCAC-----TTAC  ATCAC-----TTAC  ATCAC-----TTAC  ATCAC-----TTAC  ATCAC-----TTAC  ATCAC-----TTAC  ATCAC-----TTAC  ATCACTTACTTTAC  ATCACTTACTTTAC  ATCACTTACTTTAC  ATCACTTACTTTAC  ATCACTTACTTTAC  ATCACTTACTTTAC  ATCACTTACTTTAC  ATCACTTACTTTAC  ATCAC-----TTAC  ATGAC-----TTAC  ATGAC-----TTAC  ATCACTTACTTTAC  ATCAC-----TTAC  ATCAC-----TTAC  ATCAC-----TTAC  GTCAC-----TTAC  GTCAC-----TTAC  ATCAC-----TTAC  ATCAC-----TTAC  ATCAC-----TTAC  ATCAC-----TTAC  ATCAC-----TTAC  ATCAC----TTAAC  ATCAC-----TTAC | Ssmp | Aegilops+Triticum+Secale |
| 50 | Schedonorus arundinaceus Schedonorus arundinaceus[C] Schedonorus pratensis  Festuca altissima  Festuca ovina  Lolium multiflorum  Lolium perenne  Dactylis glomerata  Helictochloa hookeri  Deschampsia antarctica  Poa palustris  Phleum alpinum  Briza sp  Puccinellia nuttalliana  Ammophila breviligulata  Agrostis stolonifera  Hierochloe odorata  Anthoxanthum odoratum  Phalaris arundinacea  Torreyochloa  Avena sativa  Trisetum cernuum  Aegilops cylindrica  Aegilops geniculata  Aegilops speltoides  Aegilops tauschii  Triticum aestivum  Triticum monococcum aegilopoides Triticum monococcum (modified) Triticum urartu (modified)  Hordeum jubatum  Hordeum vulgare  Hordeum vulgare spontaneum Secale cereale  Bromus vulgaris  Brachypodium distachyon  Diarrhena obovata  Melica mutica  Melica subulata  Oryzopsis asperifolia Achnatherum hymenoides Ampelodesmos mauritanica Piptochaetium avenaceum  Phaenosperma globosum Brachyelytrum aristosum  Bambusa bambosa | 38173  IGS |  | TTTTTG-----------TTTTGCA  TTTTTG-----------TTTTGCA  TTTTTG-----------TTTTGCA  TTTTTG-----------TTTTGCA  TTTTTG-----------TTTTGCA  TTTTTG-----------TTTTGCA  TTTTTG-----------TTTTGCA  TTTTTG-----------TTTTGCA  TTTTTA-----------TTTTGCA  TTTTTG-----------TTTTGCA  TTTTTG-----------TTTTGCA  TTTTTG-----------TTTTGCA  TTTTTG-----------TTTTGCA  TTTTTG-----------TTTTGCA  TTTTTG-----------TTTTGCA  TTTTTG-----------TTTTGCA  TTTTTG-----------TTTTGCA  TTTTTC-----------TTTTGCA  TTTTTG-----------TTTTGCA  TTTTAA-----------TTTTGCA  TTTTCG-----------TTTTGCA  TTTTTG-----------TTTTGCA  TTTTTGCATAGAATTTTTTTTGCA  TTTTTGCATAGAATTTTTTTTGCA  TTTTTGCATAGAATTTTTTTTGCA  TTTTTGCATAGAATTTTTTTTGCA  TTTTTGCATAGAATTTTTTTTGCA  TTTTTGCATAGAATTTTTTTTGCA  TTTTTGCATAGAATTTTTTTTGCA  TTTTTGCATAGAATTTTTTTTGCA  TTTTTGCATAGAATTTGTTTTGCA  TTTTTGCATAGAATTTGTTTTGCA  TTTTTGCATAGAATTTGTTTTGCA  TTTTTGCATAGAATTTTTTTTGCA  TTTTTGCATAGAATTTGTTTTGCA  TTTTTG-----------TTTTGCA  TTTTTG-----------TTTTGCA  TTT--------------TTTTGCA  TTT--------------TTTTGCA  TTTTTG-----------TTTTGCA  TTTTTG-----------TTTTGCA  TTTTTG-----------TTTTGCA  TTTTTG-----------TTTTGCA  TTTTTG-----------TTTTGCA  TTTTTG-----------TTTTGCA  TTTTTG-----------TTTTGTA |  | Bromeae+Triticeae |
| 51 | Schedonorus arundinaceus Schedonorus arundinaceus[C] Schedonorus pratensis  Festuca altissima  Festuca ovina  Lolium multiflorum  Lolium perenne  Dactylis glomerata  Helictochloa hookeri  Deschampsia antarctica  Poa palustris  Phleum alpinum  Briza sp  Puccinellia nuttalliana  Ammophila breviligulata  Agrostis stolonifera  Hierochloe odorata  Anthoxanthum odoratum  Phalaris arundinacea  Torreyochloa  Avena sativa  Trisetum cernuum  Aegilops cylindrica  Aegilops geniculata  Aegilops speltoides  Aegilops tauschii  Triticum aestivum  Triticum monococcum aegilopoides Triticum monococcum (modified) Triticum urartu (modified)  Hordeum jubatum  Hordeum vulgare  Hordeum vulgare spontaneum Secale cereale  Bromus vulgaris  Brachypodium distachyon  Diarrhena obovata  Melica mutica  Melica subulata  Oryzopsis asperifolia Achnatherum hymenoides Ampelodesmos mauritanica Piptochaetium avenaceum  Phaenosperma globosum Brachyelytrum aristosum  Bambusa bambosa | 39180  IGS |  | ATTCATGCACGGTTACGGA  ATTCATGCACGGTTACGGA  ATTCATGCACGGTTACGGA  ATTCATGCACGGTTACGGA  ATTCATGCACGGTTACGGA  ATTCATGCACGGTTACGGA  ATTCATGCACGGTTACGGA  ATTCATGCACGGTTCCAGA  ATTCATGCATGGTTCCAGA  ATTCATGCACGGTTCCAGA  ATTCATGCAGGGTTCCAGA  ATTCATGCACGGTTCCAGA  ATTCATGCATGGTTCCAGA  ATTCATGCAAGGTTCCAGA  ATTCATGCATGGTTCCAGA  ATTCATGCATGGTTACAGA  ATTCATGCATGGTTCCAGA  ATTCATGCATGGTTCCAGA  ATTCATGCAAGGTTCCAGA  ATTCATGCATGGTTCCAGA  ATTCATGCGTGGTTCCAGA  ATTCATGCATGGTTCCAGA  ATTCATGCATGGTTCCAGA  ATTCATGCATGGTTCCAGA  ATTCATGCATGGTTCCGGA  ATTCATGCATGGTTCCAGA  ATTCATGCATGGTTCCGGA  ATTCATGCATGGTTCCAGA  ATTCATGCATGGTTCCAGA  ATTCATGCATGGTTCCAGA  ATTCATGCATGGTTCCAGA  ATTCATGCATGGTTCCAGA  ATTCATGCATGGTTCCAGA  ATTCATGCATGGTTCCAGA  ATTCATGCATGGTTCCAGA  ATTCATGCATGGTTCCAGA  ATTCATGCATGGTTCCTGA  ATTC-----------CAGA  ATTC-----------CAGA  ATTCATGCATGGTTCCAGA  ATTCATGCACGGTTCCAGA  ATTCATGCATGGTTCCAGA  ATTCATGCATGGTTCCAGA  ATTCATGCATGGTTCCAGA  ATCCATGCATGGTTCCAGA  ATTCATGCATGGTTCCAGA |  | Melica |
| 52 | Schedonorus arundinaceus Schedonorus arundinaceus[C] Schedonorus pratensis  Festuca altissima  Festuca ovina  Lolium multiflorum  Lolium perenne  Dactylis glomerata  Helictochloa hookeri  Deschampsia antarctica  Poa palustris  Phleum alpinum  Briza sp  Puccinellia nuttalliana  Ammophila breviligulata  Agrostis stolonifera  Hierochloe odorata  Anthoxanthum odoratum  Phalaris arundinacea  Torreyochloa  Avena sativa  Trisetum cernuum  Aegilops cylindrica  Aegilops geniculata  Aegilops speltoides  Aegilops tauschii  Triticum aestivum  Triticum monococcum aegilopoides Triticum monococcum (modified) Triticum urartu (modified)  Hordeum jubatum  Hordeum vulgare  Hordeum vulgare spontaneum Secale cereale  Bromus vulgaris  Brachypodium distachyon  Diarrhena obovata  Melica mutica  Melica subulata  Oryzopsis asperifolia Achnatherum hymenoides Ampelodesmos mauritanica Piptochaetium avenaceum  Phaenosperma globosum Brachyelytrum aristosum  Bambusa bambosa | 39934  IGS |  | AATTCACATTATCATTTCAA  AATTCACATTATCATTTCAA  AATTCACATTATCATTTCAA  AATTCACATTATCATTTCAA  AATTCACATTATCATTTCAA  AATTCACATTATCATTTCAA  AATTCACATTATCATTTCAA  AATTCACATTATCATTTCAA  -----ACATTATCATTTCAA  AATTGACATTATCATTTCAA  AATTCACATTATCATTTCCA  AATTCACATTATAATTTCAA  AATTCACATTATCATTTCAA  AATTCACATTATCATTTTCA  AATTCACATTATCATTTCAA  AATTCACATTATCATTTCAA  AATTCACATTATCATTTCAA  AATTCACATTATCATTTCAA  AATTCACATTATCATTTCAA  AATTCACATTATCATTTCAA  AATTTACATTATAATTTCAA  AATTCAAATTATAATTTCAA  AATTCACATTATCATTTCAA  AATTCACATTATCATTTCAA  AATTCACATTATCATTTCAA  AATTCACATTATCATTTCAA  AATTCACATTATCATTTCAA  AATTCACATTATCATTTCAA  AATTCACATTATCATTTCAA  AATTCACATTATCATTTCAA  AATTCACATTATCATTTCAA  AATTCA------CATTTCAA  AATTCA------CATTTCAA  AATTCACATTATCATTTCAA  AATTCACATTATAATTTCAA  AATTCACATTATCTTTTCAA  AATTCACATTATCATTTCAA  AATTCACATTATCATTTCAA  AATTCACATTATCATTTCAA  AATTCGCATTATCATTTCAA  AATTCGCATTATCATTTCAA  AATTCGCATTATCATTTCAA  AATTCGCATTATCATTTCAA  AATTCACATTATCATTTCAA  AATTCTGATTATCATTTCAA  AATTCAGATTATCATTTCAA |  | Hordeum vulage |
| 53 | Schedonorus arundinaceus Schedonorus arundinaceus[C] Schedonorus pratensis  Festuca altissima  Festuca ovina  Lolium multiflorum  Lolium perenne  Dactylis glomerata  Helictochloa hookeri  Deschampsia antarctica  Poa palustris  Phleum alpinum  Briza sp  Puccinellia nuttalliana  Ammophila breviligulata  Agrostis stolonifera  Hierochloe odorata  Anthoxanthum odoratum  Phalaris arundinacea  Torreyochloa  Avena sativa  Trisetum cernuum  Aegilops cylindrica  Aegilops geniculata  Aegilops speltoides  Aegilops tauschii  Triticum aestivum  Triticum monococcum aegilopoides Triticum monococcum (modified) Triticum urartu (modified)  Hordeum jubatum  Hordeum vulgare  Hordeum vulgare spontaneum Secale cereale  Bromus vulgaris  Brachypodium distachyon  Diarrhena obovata  Melica mutica  Melica subulata  Oryzopsis asperifolia Achnatherum hymenoides Ampelodesmos mauritanica Piptochaetium avenaceum  Phaenosperma globosum Brachyelytrum aristosum  Bambusa bambosa | 40083  IGS |  | ACGAGGA---ACTCACCA  ACGAAGA---ACTCACCA  ACGAGGA---ACTCACCA  ACGAGGA---ACTCACCA  ACGAGGA---ACTCACCA  ACGAGGA---ACTCACCA  ACGAGGA---ACTCACCA  ACGAGGA---ACTCACCA  ACGAGGA---ACTCACCA  ACGAGGA---ACTCACCA  ACGAGGA---ACTCAACA  ACGAGGA---ACTCACCA  ATGAGGA---ACTCACCA  ACGAGGA---ACTCACCA  ACGAGGA---ACTCACCA  ACGAGGA---ACTCACCA  ACGAGGA---ACTCACCA  ACGAGGA---ACTCACCA  ACGAGGA---ACTCACCA  ACGAGGA---ACTCACCA  ACGAGGA---ACTCACCA  ACGAGGA---ACTCACCA  ACGAGGAACTACTCACCA  ACGAGGAACTACTCACCA  ACGAGGAACTACTCACCA  ACGAGGAACTACTCACCA  ACGAGGAACTACTCACCA  ACGAGGAACTACTCACCA  ACGAGGAACTACTCACCA  ACGAGGAACTACTCACCA  ACGAGGAACTACTCACCA  ACGAGGAACTACTCACCA  ACGAGGAACTACTCACCA  ACGAGGAACTACTCACCA  ACGAGGAACTACTCACCA  ACGAGGA---ACTCACCA  ACGAGGA---ACTCACCA  ACGAGGA---ACTCACCA  ACGAGGA---ACTCACCA  ACGAGGA---ACTCACCA  ACGAGGA---ACTCACCA  ACGAGGA---ACTCACCA  ACGAGGA---ACTCACCA  ACGAGGA---ACTCACCA  ACGAGGA---ACTCACCA  ACGAGGA---ACTCACCA | Ssmp | Bromeae+Triticeae |
| 54 | Schedonorus arundinaceus Schedonorus arundinaceus[C] Schedonorus pratensis  Festuca altissima  Festuca ovina  Lolium multiflorum  Lolium perenne  Dactylis glomerata  Helictochloa hookeri  Deschampsia antarctica  Poa palustris  Phleum alpinum  Briza sp  Puccinellia nuttalliana  Ammophila breviligulata  Agrostis stolonifera  Hierochloe odorata  Anthoxanthum odoratum  Phalaris arundinacea  Torreyochloa  Avena sativa  Trisetum cernuum  Aegilops cylindrica  Aegilops geniculata  Aegilops speltoides  Aegilops tauschii  Triticum aestivum  Triticum monococcum aegilopoides Triticum monococcum (modified) Triticum urartu (modified)  Hordeum jubatum  Hordeum vulgare  Hordeum vulgare spontaneum Secale cereale  Bromus vulgaris  Brachypodium distachyon  Diarrhena obovata  Melica mutica  Melica subulata  Oryzopsis asperifolia Achnatherum hymenoides Ampelodesmos mauritanica Piptochaetium avenaceum  Phaenosperma globosum Brachyelytrum aristosum  Bambusa bambosa | 40722  IGS |  | AGGTCTTTCTTTCAC  AGGTCTTTCTTTCAC  AGGTCTTTCTTTCAC  AGGTCTTTCTTTCAC  AGGTCTTTCTTTCAC  AGGTCTTTCTTTCAC  AGGTCTTTCTTTCAC  AGGTCTTTCTTTCAC  AGGTCTTTCTTTCAC  GGGTCTTTGTTTCAC  AGGTCTTTCTTTCAC  AGGTCTTTCTTTCAC  AGGTC----TTTCAC  AGGTCTTTCTTTTAC  AGGTC----TTTCAC  AGGTC----TTTCAC  AGGTC----TTTCAC  AGGTC----TTTCGC  AGGTC----TTTCAC  AGGTC----TTTCAC  AGGTC----TTTCAC  AGGTC----TTTCAC  AAGTC----TTTCAC  AAGTC----TTTCAC  AAGTC----TTTCAC  AAGTC----TTTCAC  AAGTC----TTTCAC  AAGTC----TTTCAC  AAGTC----TTTCAC  AAGTC----TTTCAC  AAGTC----TTTCAC  AAGCC----TTTCAC  AAGCC----TTTCAC  AAGTC----TTTCAC  AAGTC----TTTCAC  AAGTC----TTTCAC  AGGTC----TTTCAC  AGGTC----TTTCAC  AGGTC----TTTCAC  AGGTC----TTTCAC  AGGTC----TTTCAC  AGGTC----TTTCAC  AGGTC----TTTCAC  AGGTC----TTTCAC  AGGTC----TTTCAC  AGGTC----TTTCAC | ssmp | Poeae clade 2 |
| 55 | Schedonorus arundinaceus Schedonorus arundinaceus[C] Schedonorus pratensis  Festuca altissima  Festuca ovina  Lolium multiflorum  Lolium perenne  Dactylis glomerata  Helictochloa hookeri  Deschampsia antarctica  Poa palustris  Phleum alpinum  Briza sp  Puccinellia nuttalliana  Ammophila breviligulata  Agrostis stolonifera  Hierochloe odorata  Anthoxanthum odoratum  Phalaris arundinacea  Torreyochloa  Avena sativa  Trisetum cernuum  Aegilops cylindrica  Aegilops geniculata  Aegilops speltoides  Aegilops tauschii  Triticum aestivum  Triticum monococcum aegilopoides Triticum monococcum (modified) Triticum urartu (modified)  Hordeum jubatum  Hordeum vulgare  Hordeum vulgare spontaneum Secale cereale  Bromus vulgaris  Brachypodium distachyon  Diarrhena obovata  Melica mutica  Melica subulata  Oryzopsis asperifolia Achnatherum hymenoides Ampelodesmos mauritanica Piptochaetium avenaceum  Phaenosperma globosum Brachyelytrum aristosum  Bambusa bambosa | 40840  IGS |  | GAGC------GAAGTAAG  GAGC------GAAGTAAG  GAGC------GAAGTAAG  GAGC------GAAGTAAG  GAGC------GAAGTAAG  GAGC------GAAGTAAG  GAGC------GAAGTAAG  GAGC------GAAGTAAG  GAGC------GAAGTAAG  GAGC------GAAGTAAG  GAGC------GAAGTAAG  GAGC------GAAGTAAG  GAGC------GAAGTAAG  GAAC------GAAGTAAG  GAGC------GAAGTAAG  GAGC------GAAGTAAG  GAGC------GAAGTAAG  GAGC------AAAGTAAG  AAGC------GAAGTAAG  GAGC------GAAGTAAG  GAGC------GAAGTAAG  GAGC------GAAGTAAG  GAGC------GAAGTAAG  GAGC------GAAGTAAG  GAGC------GAAGTAAG  GAGC------GAAGTAAG  GAGC------GAAGTAAG  GAGCGAAGTAGAAGTAAG  GAGCGAAGTAGAAGTAAG  GAGC------GAAGTAAG  GAGC------GAAGTAAG  GAGC------GAAGTAAG  GAGC------GAAGTAAG  GAGC------GAAGTAAG  GAGCGAAGTAAGTGTAAG  GAGC------GAAGCAAG  GAGC------GAAGTAAG  GAGCGAAGTAGAAGTAAG  GAGCGAAGTAGAAGTAAG  GAGC------GAAGTAAG  GAGC------GAAGTAAG  GAGC------GAAGTAAG  GAGC------GAAGTAAG  GAGC------GAAGTAAG  GAGC------GAAGTAAG  GAGC------GAAGTAAG | ssmp | Homoplasy |
| 56 | Schedonorus arundinaceus Schedonorus arundinaceus[C] Schedonorus pratensis  Festuca altissima  Festuca ovina  Lolium multiflorum  Lolium perenne  Dactylis glomerata  Helictochloa hookeri  Deschampsia antarctica  Poa palustris  Phleum alpinum  Briza sp  Puccinellia nuttalliana  Ammophila breviligulata  Agrostis stolonifera  Hierochloe odorata  Anthoxanthum odoratum  Phalaris arundinacea  Torreyochloa  Avena sativa  Trisetum cernuum  Aegilops cylindrica  Aegilops geniculata  Aegilops speltoides  Aegilops tauschii  Triticum aestivum  Triticum monococcum aegilopoides Triticum monococcum (modified) Triticum urartu (modified)  Hordeum jubatum  Hordeum vulgare  Hordeum vulgare spontaneum Secale cereale  Bromus vulgaris  Brachypodium distachyon  Diarrhena obovata  Melica mutica  Melica subulata  Oryzopsis asperifolia Achnatherum hymenoides Ampelodesmos mauritanica Piptochaetium avenaceum  Phaenosperma globosum Brachyelytrum aristosum  Bambusa bambosa | 40885  igs |  | GTCCTATCTATAAGACTAT-AAGAG  GTCCTATCTATAAGACTAT-AAGAG  GTCCTATCTATAAGACTAT-AAGAG  GTCCTAT--------CTAT-AAGAG  GTCCTAT--------CTAT-AAGAG  GTCCTATCTATAAGACTAT-AAGAG  GTCCTATCTATAAGACTAT-AAGAG  GTCCTAT--------CTAT-AAGAG  GTCCTAT--------CTAT-AAGAG  GTCCTAT--------CTATAAAGAG  GTCCTAT--------CTAT-AAGAG  GTCCTAT--------CTAT-AAGAG  GTCCTAT--------CTAT-AAGAG  GTCCTAT--------CTAT-AAGAG  GTCCTAT--------CTAT-AAGAG  GTCCTAT--------CTAT-AAGAG  GTCCTAT--------CTAT-AAGAG  GTCCTAT--------CTAT-AAGAG  GTCCTAT--------CTAT-AAGAG  GTCCTAT--------CTAT-AAGAG  GTCCTAT--------CTAT-AAGAG  GTCCTAT--------CTAT-AAGAG  GTCCTAT--------CTAT-AAGAG  GTCCTAT--------CTAT-AAGAG  GTCCTAT--------CTAT-AAGAG  GTCCTAT--------CTAT-AAGAG  GTCCTAT--------CTAT-AAGAG  GTCCTAT--------CTAT-AAGAG  GTCCTAT--------CTAT-AAGAG  GTCCTAT--------CTAT-AAGAG  GTCCTAT--------CTAT-AAGAG  GTCCTAT--------CTAT-AAGAG  GTCCTAT--------CTAT-AAGAG  GTCCTAT--------CTAT-AAGAG  GTCCTAT--------CTAT-AAGAG  GTCCTAT--------CTAT-AAGAG  GTCCTAT--------CTAT-AAGAG  GTCCTAT--------CTAT-AAGAG  GTCCTAT--------CTAT-AAGAG  GTCCTAT--------CTAT-AAGAG  GTCCTAT--------CTAT-AAGAG  GTCCTAT--------CTAT-AAGAG  GTCCTAT--------CTAT-AAGAG  GTCCTAT--------CTAT-AAGAG  GTCCTAT--------TTAT-AAGAG  GTCCTAT--------CTAT-AAGAG | ssmp | Lolium+Schedonorus |
| 57 | Schedonorus arundinaceus Schedonorus arundinaceus[C] Schedonorus pratensis  Festuca altissima  Festuca ovina  Lolium multiflorum  Lolium perenne  Dactylis glomerata  Helictochloa hookeri  Deschampsia antarctica  Poa palustris  Phleum alpinum  Briza sp  Puccinellia nuttalliana  Ammophila breviligulata  Agrostis stolonifera  Hierochloe odorata  Anthoxanthum odoratum  Phalaris arundinacea  Torreyochloa  Avena sativa  Trisetum cernuum  Aegilops cylindrica  Aegilops geniculata  Aegilops speltoides  Aegilops tauschii  Triticum aestivum  Triticum monococcum aegilopoides Triticum monococcum (modified) Triticum urartu (modified)  Hordeum jubatum  Hordeum vulgare  Hordeum vulgare spontaneum Secale cereale  Bromus vulgaris  Brachypodium distachyon  Diarrhena obovata  Melica mutica  Melica subulata  Oryzopsis asperifolia Achnatherum hymenoides Ampelodesmos mauritanica Piptochaetium avenaceum  Phaenosperma globosum Brachyelytrum aristosum  Bambusa bambosa | 41187  atpF intron 1 | atpF intron 1 | TTAGAAATCACATAATATGGAA  TTAGAAATCACATAATATGGAA  TTAGAAATCACATAATATGGAA  TTAGAAATC-----ATATGGAA  TTAGAAATC-----ATATGGAA  TTAGAAATCACATAATATGGAA  TTAGAAATCACATAATATGGAA  TTAGAAATC-----ATATGGAA  TCAGAAATC-----ATATGGAA  TTAGAAATC-----ATATGGAA  TCAGAAATC-----ATATGAAA  TCAGAAATC-----ATATGGAA  TCAGAAATC-----ATATGGAA  TCAGAAATC-----ATATGGAA  TCAGAAATC-----ATATGGAA  TCAGAAATT-----ATATGGAA  TCAGAAATC-----ATATGGAA  TCAGAAATC-----ATATGGAA  TCAGAAATC-----ATATGGAA  TCAGAAATC-----ATATGGAA  TCAGAAATC-----ATATGGAA  TCAGAAATC-----ATATGGAA  TCAGAAATC-----ATATGGAA  TCAGAAATC-----ATATGGAA  TCAGAAATC-----ATATGGAA  TCAGAAATC-----ATATGGAA  TCAGAAATC-----ATATGGAA  TCAGAAATC-----ATATGGAA  TCAGAAATC-----ATATGGAA  TCAGAAATC-----ATATGGAA  TCAGAAATC-----ATATGGAA  TCAGAAATC-----ATATGGAA  TCAGAAATC-----ATATGGAA  TCAGAAATC-----ATATGGAA  TCAGAAATC-----ATATGGAA  TCAGAAATC-----ATATGGAA  TCAGAAATC-----ATATGGAA  TCAGAAATC-----ATATGGAA  TCAGAAATC-----ATATGGAA  TCAGAAATC-----ATATGGAA  TCAGAAATC-----ATATGGAA  TCAGAAATC-----ATATGGAA  TCAGAAATC-----ATATGGAA  TCAGAAATC-----ATATGGAA  TCAGAAATC-----ATATGGAA  TCAGAAATC-----ATATGGAA |  | Lolium+Schedonorus |
| 58 | Schedonorus arundinaceus Schedonorus arundinaceus[C] Schedonorus pratensis  Festuca altissima  Festuca ovina  Lolium multiflorum  Lolium perenne  Dactylis glomerata  Helictochloa hookeri  Deschampsia antarctica  Poa palustris  Phleum alpinum  Briza sp  Puccinellia nuttalliana  Ammophila breviligulata  Agrostis stolonifera  Hierochloe odorata  Anthoxanthum odoratum  Phalaris arundinacea  Torreyochloa  Avena sativa  Trisetum cernuum  Aegilops cylindrica  Aegilops geniculata  Aegilops speltoides  Aegilops tauschii  Triticum aestivum  Triticum monococcum aegilopoides Triticum monococcum (modified) Triticum urartu (modified)  Hordeum jubatum  Hordeum vulgare  Hordeum vulgare spontaneum Secale cereale  Bromus vulgaris  Brachypodium distachyon  Diarrhena obovata  Melica mutica  Melica subulata  Oryzopsis asperifolia Achnatherum hymenoides Ampelodesmos mauritanica Piptochaetium avenaceum  Phaenosperma globosum Brachyelytrum aristosum  Bambusa bambosa | 41365  atpF intron 1 | atpF intron 1 | GTACCGAAATGCTTTAAAC  GTACCGAAATGCTTTAAAC  GTACCGAAATGCTTTAAAC  GTACCGAAATGCTTCAAAC  GTACCGAAAT-CTTTAAAC  GTACCGAAATGCTTTAAAC  GTACCGAAATGCTTTAAAC  GTACCGAAATGCTTTAAAC  GTACTGAAATGTTTTAAAC  GTACCGAAATGCTTTAAAC  GTACCGAAATGCTTTAAAC  GTACCGAAATGCTTTAAAC  GTACCTAAATGCTTTAAAC  GTACCGAAATGCTTTAAAC  GTACCTAAATGCTTTAAAT  GTACCTAAATGCTTTAAAC  GTACCTAAATGCTTTAAAT  GTAGCTAAATGCTTTAAAC  GTACCTAAATGCTTTAAAC  GTACCTAAATGTTTTAAAC  GTACCTAAATGCTTTAAAT  GTACCTAAATGCTTTAAAC  GTACCGAA----TTTAAAC  GTACCGAA----TTTAAAC  GTACCGAA----TTTAAAC  GTACCGAA----TTTAAAC  GTACCGAA----TTTAAAC  GTACC-AA----TTTAAAC  GTACC-AA----TTTAAAC  GTACC-AA----TTTAAAC  GTACCGAA----TTTAAAC  GTACCGAA----TTTAAAC  GTACCGAA----TTTAAAC  GTACCGAA----TTTAAAC  ---CCGAAATACTTTAAAC  GTACCGAAATGCTTTAAAC  GTACCGAAATGCTTTAAAC  GTACCGAAATGCTTTAAAC  GTACCGAAATGCTTTAAAC  GTACCGAAATGCTTTAAAC  GTACCGAAATGCTTTAAAC  GTACCGAAATGCTTTAAAC  GTACCGAAATGCTTTAAAC  GTACCGAAATACTTTAAAC  GTACCGAAATGCTTTAAAC  GTACCGAAATGCTTTAAAC |  | Triticeae |
| 59 | Schedonorus arundinaceus Schedonorus arundinaceus[C] Schedonorus pratensis  Festuca altissima  Festuca ovina  Lolium multiflorum  Lolium perenne  Dactylis glomerata  Helictochloa hookeri  Deschampsia antarctica  Poa palustris  Phleum alpinum  Briza sp  Puccinellia nuttalliana  Ammophila breviligulata  Agrostis stolonifera  Hierochloe odorata  Anthoxanthum odoratum  Phalaris arundinacea  Torreyochloa  Avena sativa  Trisetum cernuum  Aegilops cylindrica  Aegilops geniculata  Aegilops speltoides  Aegilops tauschii  Triticum aestivum  Triticum monococcum aegilopoides Triticum monococcum (modified) Triticum urartu (modified)  Hordeum jubatum  Hordeum vulgare  Hordeum vulgare spontaneum Secale cereale  Bromus vulgaris  Brachypodium distachyon  Diarrhena obovata  Melica mutica  Melica subulata  Oryzopsis asperifolia Achnatherum hymenoides Ampelodesmos mauritanica Piptochaetium avenaceum  Phaenosperma globosum Brachyelytrum aristosum  Bambusa bambosa | 41439  atpF intron 1 | atpF intron 1 | GGTTTGAACT-----ATTTACTAG  GGTTTGAACT-----ATTTACTAG  GGTTTGAACT-----ATTTACTAG  GGTTTGAACT-----ATTTACTAG  GGTTTGAACT-----ATTTACTAG  GGTTTGAACT-----ATTTACTAG  GGTTTGAACT-----ATTTACTAG  GGTTTGAACT-----ATTTACTAG  GGTTTAAACT-----ATTTACTAG  GGTTTGAACT-----ATTTACTAG  GGTTTGAACT-----ATTTACTAG  GGTTTGAACT-----ATTTATTAG  GGTTTGAACT-----ATTTACTAG  GGTTTGAACT-----ATTTACTAG  GGTTTGAACT-----ATTTACTAG  GGTTTGAACT-----ATTTACTAG  GGTTTGAACT-----ATTTACTAG  GGTTTGAACT-----ATTTACTAG  GGTTTGCACT-----ATTTACTAG  GGTTTGAACT-----ATTTACTAG  GGTTTGAACT-----ATTTACTAG  GGTTTGAACT-----ATTTACTAG  GGTTTGAACT-----ATTTACTAG  GGTTTGAACT-----ATTTACTAG  GGTTTGAACT-----ATTTACTAG  GGTTTGAACT-----ATTTACTAG  GGTTTGAACT-----ATTTACTAG  GGTTTGAACTATTTAATTTACTAG  GGTTTGAACTATTTAATTTACTAG  GGTTTGAACT-----ATTTACTAG  GGTTTGAACT-----ATTTACTAG  GGTTTGAACT-----ATTTACTAG  GGTTTGAACT-----ATTTACTAG  GGTTTGAACT-----ATTTACTAG  GGTTTGAACT-----ATTTACTAG  GGTTTGAACT-----ATTTACTAG  GGTTTGAACT-----ATTTACTAG  GGTTTGAACT-----ATTTATTAG  GGTTTGAACT-----ATTTATTAG  GGTTTGAACT-----ATTTACTAG  GGTTTGAACT-----TTTTACTAG  GGTTTGAACT-----ATTTACTAG  GGTTTGAACT-----ATTTACTAG  GGTTTGAACT-----ATTTACTAG  GGTTTGAACT-----ATTTACTAG  GGTTTAAACT-----ATTTACTAG | Ssmp | Triticum monococcum |
| 60 | Schedonorus arundinaceus Schedonorus arundinaceus[C] Schedonorus pratensis  Festuca altissima  Festuca ovina  Lolium multiflorum  Lolium perenne  Dactylis glomerata  Helictochloa hookeri  Deschampsia antarctica  Poa palustris  Phleum alpinum  Briza sp  Puccinellia nuttalliana  Ammophila breviligulata  Agrostis stolonifera  Hierochloe odorata  Anthoxanthum odoratum  Phalaris arundinacea  Torreyochloa  Avena sativa  Trisetum cernuum  Aegilops cylindrica  Aegilops geniculata  Aegilops speltoides  Aegilops tauschii  Triticum aestivum  Triticum monococcum aegilopoides Triticum monococcum (modified) Triticum urartu (modified)  Hordeum jubatum  Hordeum vulgare  Hordeum vulgare spontaneum Secale cereale  Bromus vulgaris  Brachypodium distachyon  Diarrhena obovata  Melica mutica  Melica subulata  Oryzopsis asperifolia Achnatherum hymenoides Ampelodesmos mauritanica Piptochaetium avenaceum  Phaenosperma globosum Brachyelytrum aristosum  Bambusa bambosa | 41551  atpF intron 1 | atpF intron 1 | TTTTGG----ATTTGAAG  TTTTGG----ATTTGAAG  TTTTGG----ATTTGAAG  TTTTGG----ATTTGAAG  TTTTGG----ATTTGAAG  TTTTGG----ATTTGAAG  TTTTGG----ATTTGAAG  TTTTGG----ATTAGGAG  TTTTGG----ATTTGAAG  TTTTGG----ATTTGAAG  TTTTGG----ATTTGAAG  TTTTGG----ATTTGAAG  TTTTGG----ATTGGAAG  TTTTGG----ATTTGAAG  TTTTGG----ATTTGAAG  TTTTGG----ATTTGAAG  TTTTGGATTTATTTAAAG  TTTTGGATTTATTTAAAG  TTTTGG----ATTTGAAG  TTTTGG----ATTTGAAG  TTTTGG----ATTTGAAG  TTTTGG----ATTTGAAG  TTTTGG----ATTTGAAG  TTTTGG----ATTTGAAG  TTTTGG----ATTTGAAG  TTTTGG----ATTTGAAG  TTTTGG----ATTTGAAG  TTTTGG----ATTTGAAA  TTTTGG----ATTTGAAG  TTTTGG----ATTTGAAG  TTTTGG----ATTTGAAG  TTTTGG----ATTTGAAG  TTTTGG----ATTTGAAG  TTTTGG----ATTTGAAG  TTTTGG----ATTTGAAG  TTTTGG----ATTTGAAG  TTTTGG----ATTTGAAG  TTTTGG----ATTTGAAG  TTTTGG----ATTTGAAG  TTTTGG----ATTTGAGG  TTTTGG----ATTTGAGG  TTTTGG----ATTTTAGG  TTTTGG----ATTTGAGG  TTTTGG----ATTTGAAG  TTTTGG----ATTTGAAG  TTTTGG----ATTTGAAG | Ssmp | Anthoxanthinae |
| 61 | Schedonorus arundinaceus Schedonorus arundinaceus[C] Schedonorus pratensis  Festuca altissima  Festuca ovina  Lolium multiflorum  Lolium perenne  Dactylis glomerata  Helictochloa hookeri  Deschampsia antarctica  Poa palustris  Phleum alpinum  Briza sp  Puccinellia nuttalliana  Ammophila breviligulata  Agrostis stolonifera  Hierochloe odorata  Anthoxanthum odoratum  Phalaris arundinacea  Torreyochloa  Avena sativa  Trisetum cernuum  Aegilops cylindrica  Aegilops geniculata  Aegilops speltoides  Aegilops tauschii  Triticum aestivum  Triticum monococcum aegilopoides Triticum monococcum (modified) Triticum urartu (modified)  Hordeum jubatum  Hordeum vulgare  Hordeum vulgare spontaneum Secale cereale  Bromus vulgaris  Brachypodium distachyon  Diarrhena obovata  Melica mutica  Melica subulata  Oryzopsis asperifolia Achnatherum hymenoides Ampelodesmos mauritanica Piptochaetium avenaceum  Phaenosperma globosum Brachyelytrum aristosum  Bambusa bambosa | 41658  atpF intron 1 | atpF intron 1 | ATTAACT----AAAGGCCAA  ATTAACT----AAAGGCCAA  ATTAACT----AAAGGCCAA  ATTAACT----AAAGGCCAA  ATTAACT----AAAGGGCAA  ATTAACT----AAAGGCCAA  ATTAACT----AAAGGCCAA  ATTAACT----AAAGGGCAA  ATTAACT----AAAGAGCAA  ATTAACT----AAAGAGCAA  ATTAACT----AAAGGGCAA  ATTAACT----AAAGGGCAA  ATTAACT----AAAGGGCAA  ATTAACT----AAAGGGCAA  ATTAACT----AAAGGGCAA  ATTAACT----AAAGGGCAA  ATTAACTAAAGAAAGGGCAA  ATTAACTAAAGAAAGGGCAA  ATTAACT----AAAGGGCAA  ATTAACT----AAAGGGCAA  ATTAACT----AAAGGGCAA  ATTAACT----AAAGGGCAA  ATTAACT----AGAGGGCAA  ATTAACT----AGAGGGCAA  ATTAA------AGAGGGCAA  ATTAACT----AGAGGGCAA  ATTAA------AGAGGGCAA  ATTAACT----AGAGGGCAA  ATTAACT----AGAGGGCAA  ATTAACT----AGAGGGCAA  ATTAACT----AAAGGGCAA  ATTAACT----AAAGGGCAA  ATTAACT----AAAGGGCAA  ATTAACT----AAAGGGCAA  ATTAACT----AAAGGGCAA  CTTAAGT----AAAGTGCAA  ATTAACT----AAAGGGCAA  ATTAACT----AAAGGGCAA  ATTAACT----AAAGGGCAA  ATTAACT----AAAGGGCAA  ATTAGCT----AAAGGGTAA  ATTAACT----AAAGGGCAA  ATTAACT----AAAGGGCAA  ATTAACT----AAAGGGCAA  ATTAACC----AAAGGGCAA  ATTAACT----AAAGGGCAA | ssmp | Anthoxanthinae |
| 62 | Schedonorus arundinaceus Schedonorus arundinaceus[C] Schedonorus pratensis  Festuca altissima  Festuca ovina  Lolium multiflorum  Lolium perenne  Dactylis glomerata  Helictochloa hookeri  Deschampsia antarctica  Poa palustris  Phleum alpinum  Briza sp  Puccinellia nuttalliana  Ammophila breviligulata  Agrostis stolonifera  Hierochloe odorata  Anthoxanthum odoratum  Phalaris arundinacea  Torreyochloa  Avena sativa  Trisetum cernuum  Aegilops cylindrica  Aegilops geniculata  Aegilops speltoides  Aegilops tauschii  Triticum aestivum  Triticum monococcum aegilopoides Triticum monococcum (modified) Triticum urartu (modified)  Hordeum jubatum  Hordeum vulgare  Hordeum vulgare spontaneum Secale cereale  Bromus vulgaris  Brachypodium distachyon  Diarrhena obovata  Melica mutica  Melica subulata  Oryzopsis asperifolia Achnatherum hymenoides Ampelodesmos mauritanica Piptochaetium avenaceum  Phaenosperma globosum Brachyelytrum aristosum  Bambusa bambosa | 41669  atpF intron 1 | atpF intron 1 | TTGCTGACC-----------------ATCTA  TTGCTGACC-----------------ATCTA  TTGCTGACC-----------------ATCTA  TTGCTGACC-----------------ATCTA  TTGCTGACC-----------------ATCTA  TTGCTGACC-----------------ATCTA  TTGCTGACC-----------------ATCTA  TTGCTGACC-----------------ATCTA  TTGCTGACC-----------------ATCTA  TTGCTGACC-----------------ATCTA  TTGCTGACC-----------------ATCTA  TTGCTGACC-----------------ATCTA  TTGCTGACCATGATAGA----TTTTTATCTA  TTGCTGACC-----------------ATCTA  TTGCTGACCATGATAGA----TTTTTATCTA  TTGCTGACCATGATAGA----TTTTTATCTA  TTGCTGACCATTATAGA----TTTTTATCTA  TTGCTGACCATGATAGA----TTTTTATCTA  TTGCTGACCATGATAGA----TTTTTATCTA  TTGCTGACCATGATAGA----TTTTTATCTA  TTGCTGACCATGATAGA----TTTTTATCTA  TTGCTGACCATGATAGA----TTTTTATCTA  TTGCTGACCATGATATA----TTTTTATCTA  TTGCTGACCATGATATA----TTTTTATCTA  TTGCTGACCATGATATA----TTTTTATCTA  TTGCTGACCATGATATA----TTTTTATCTA  TTGCTGACCATGATATA----TTTTTATCTA  TTGCTGACCATGATATA----TTTTTATCTA  TTGCTGACCATGATATA----TTTTTATCTA  TTGCTGACCATGATATA----TTTTTATCTA  TTGCTGACCATGATATA----TTTTTATCTA  TTGCTGACCATGATATA----TTTTTATCTA  TTGCTGACCATGATATA----TTTTTATCTA  TTGCTGACCATGATATATTTTTTTTTATCTA  TTGCTGACCATGATATA----TTTTTATCTA  TTGCTGACCATGATAGA----TTTTTATCTA  TTGCTGACCATGATAGA----TTTTTATCTA  TTGCTGACCATGATATA----TTTTTATTTA  TTGCTGACCATGATATA----TTTTTATTTA  TTGCTGACCATGATAGA----TTTTTATCTA  TTGCTGACCATGATAGA----TTTTTATCTA  TTGCTGACCATGATAGA----TTTTTATCTA  TTGCTGACCATGATAGA----TTTTTATCTA  TTGCTGACCATGATAGA----TTTTTATCTA  TTGCTGACCATGATAGA----TTTTTATCTA  TTGCTGACCATGATAGA----TTTTTATCTA |  | Poeae clade 2 |
| 63 | Schedonorus arundinaceus Schedonorus arundinaceus[C] Schedonorus pratensis  Festuca altissima  Festuca ovina  Lolium multiflorum  Lolium perenne  Dactylis glomerata  Helictochloa hookeri  Deschampsia antarctica  Poa palustris  Phleum alpinum  Briza sp  Puccinellia nuttalliana  Ammophila breviligulata  Agrostis stolonifera  Hierochloe odorata  Anthoxanthum odoratum  Phalaris arundinacea  Torreyochloa  Avena sativa  Trisetum cernuum  Aegilops cylindrica  Aegilops geniculata  Aegilops speltoides  Aegilops tauschii  Triticum aestivum  Triticum monococcum aegilopoides Triticum monococcum (modified) Triticum urartu (modified)  Hordeum jubatum  Hordeum vulgare  Hordeum vulgare spontaneum Secale cereale  Bromus vulgaris  Brachypodium distachyon  Diarrhena obovata  Melica mutica  Melica subulata  Oryzopsis asperifolia Achnatherum hymenoides Ampelodesmos mauritanica Piptochaetium avenaceum  Phaenosperma globosum Brachyelytrum aristosum  Bambusa bambosa | 41741  atpF intron 1 | atpF intron 1 | AGAGTCCTCTTAATTCTTAATATT  AGAGTCCTCTTAATTCTTAATATT  AGAGTCCTCTTAATTCTTAATATT  AGAGTCC-------TCTTAATATT  AGAGTCC-------TCTTAATATT  AGAGTCCTCTTAATTCTTAATATT  AGAGTCCTCTTAATTCTTAATATT  AGAGTCC-------TCTTAATATT  AGAGTCC-------TCTTAATATT  AGAGTCC-------TCTTAATATT  AGAGTCC-------TCTTAATATT  AGAGTCC-------TCTTAATATT  AGAGTCC-------TCTTAATATT  AGAGTCC-------TCTTAATATT  AGAGTCC-------TCTTAATATT  AGAGTCC-------TCTTAATATT  AGAGTCC-------TCTTAATATT  AGAGTCC-------TCTTAATATT  AGAGTCC-------TCTTAATATT  AGAGTCC-------TCTTAATATT  AGAGTCC-------TCTTAATATT  AGAGTCC-------TCTTAATATT  AGAGTCC-------TCTTAATATT  AGAGTCC-------TCTTAATATT  AGAGTCC-------TCTTAATATT  AGAGTCC-------TCTTAATATT  AGAGTCC-------TCTTAATATT  AGAGTCC-------TCTTAATATT  AGAGTCC-------TCTTAATATT  AGAGTCC-------TCTTAATATT  AGAGTCC-------TCTTAATATT  AGAGTCC-------TCTTAATATT  AGAGTCC-------TCTTAATATT  AGAGTCC-------TCTTAATATT  AGAGTCC-------TCTTAATATT  AGAGTCC-------TCTTAATATT  AGAGTCC-------TCTTAATATT  AGAGTCC-------TCTTTATATT  AGAGTCC-------TCTTAATATT  AGAGTCC-------TCTTAATATT  AGAGTCC-------TCTTAATATT  AGAGTCC-------TCTTAATATT  AGAGTCC-------TCTTAATATT  AGAGTCC-------TCTTAATATT  AGAGTCC-------TCTTAATATT  AGAGTCC-------TCTTAATATT | ssmp | Lolium+Schedonorus |
| 64 | Schedonorus arundinaceus Schedonorus arundinaceus[C] Schedonorus pratensis  Festuca altissima  Festuca ovina  Lolium multiflorum  Lolium perenne  Dactylis glomerata  Helictochloa hookeri  Deschampsia antarctica  Poa palustris  Phleum alpinum  Briza sp  Puccinellia nuttalliana  Ammophila breviligulata  Agrostis stolonifera  Hierochloe odorata  Anthoxanthum odoratum  Phalaris arundinacea  Torreyochloa  Avena sativa  Trisetum cernuum  Aegilops cylindrica  Aegilops geniculata  Aegilops speltoides  Aegilops tauschii  Triticum aestivum  Triticum monococcum aegilopoides Triticum monococcum (modified) Triticum urartu (modified)  Hordeum jubatum  Hordeum vulgare  Hordeum vulgare spontaneum Secale cereale  Bromus vulgaris  Brachypodium distachyon  Diarrhena obovata  Melica mutica  Melica subulata  Oryzopsis asperifolia Achnatherum hymenoides Ampelodesmos mauritanica Piptochaetium avenaceum  Phaenosperma globosum Brachyelytrum aristosum  Bambusa bambosa | 42031  atpF intron 1 | atpF intron 1 | AACTTAATAGCAAAATA  AACTTAATAGCAAAATA  AACTTAATAGCAAAATA  AACTTAATAGCAAAATA  AACTTAATAGCAAAATA  AACTTAATAGCAAAATA  AACTTAATAGCAAAATA  AACTTAATAGCAAAATA  AACTTAATAGCAAAATA  AACTTAATAGCAAAATA  AACTTAATAGGAAAATA  AACTTAATAGCAAAATA  AACTTAATAGCAAGATA  AACTTAATAGCAAAATA  AACTTAATAGCAAGATA  AACTTAATAGCAAGATA  AACTTAATAGCAAGATA  AACTTAATAGCAAGATA  AACTTAATAGCAAGATA  AACTTAATAGCAAGATA  AACTTAATAGCAAGATA  AACTTAATAGCAAGATA  AACTTA----CAAAATA  AACTTA----CAAAATA  AACTTA----CAAAATA  AACTTA----CAAAATA  AACTTA----CAAAATA  AACTTA----CAAAATA  AACTTA----CAAAATA  AACTTA----CAAAATA  AACTTA----CAAAATA  AACTTA----CAAAATA  AACTTA----CAAAATA  AACTTA----CAAAATA  AACTTA----CAAAATA  AACTTAATAGCAAGATA  AACTTAATAGCAAGATA  AACTTAATAGCAAGATA  AACTTAATAGCAAGATA  AACTTAATAGCAAGATA  AACTTAATAGCAAGATA  AACTTAATAGCAAGATA  AACTTAATAGCAAGATA  AACTTAATAGCAAGATA  AACTTAATAGCAAGATA  AACTTAATAGCAAGATA |  | Bromeae+Triticeae |
| 65 | Schedonorus arundinaceus Schedonorus arundinaceus[C] Schedonorus pratensis  Festuca altissima  Festuca ovina  Lolium multiflorum  Lolium perenne  Dactylis glomerata  Helictochloa hookeri  Deschampsia antarctica  Poa palustris  Phleum alpinum  Briza sp  Puccinellia nuttalliana  Ammophila breviligulata  Agrostis stolonifera  Hierochloe odorata  Anthoxanthum odoratum  Phalaris arundinacea  Torreyochloa  Avena sativa  Trisetum cernuum  Aegilops cylindrica  Aegilops geniculata  Aegilops speltoides  Aegilops tauschii  Triticum aestivum  Triticum monococcum aegilopoides Triticum monococcum (modified) Triticum urartu (modified)  Hordeum jubatum  Hordeum vulgare  Hordeum vulgare spontaneum Secale cereale  Bromus vulgaris  Brachypodium distachyon  Diarrhena obovata  Melica mutica  Melica subulata  Oryzopsis asperifolia Achnatherum hymenoides Ampelodesmos mauritanica Piptochaetium avenaceum  Phaenosperma globosum Brachyelytrum aristosum  Bambusa bambosa | 44222  IGS |  | TCTTAGTT-----TTTAG-TATA  TCTTAGTT-----TTTAG-TATA  TCTTAGTT-----TTTAG-TATA  TCTTAGTT-----TTTAG-TATA  TCTTAGTT-----TTTAGTTATA  TCTTAGTT-----TTTAG-TATA  TCTTAGTT-----TTTAG-TATA  TCTTAGTT-----TTTAG-TATA  TCTTAGTT-----TTTAG-TATA  TCTTAGTT-----TTTAG-TATA  TCTTAGTT-----TTTAG-TATA  TCTTAGTT-----TTTAG-TATA  TATTAGTT-----TTTAG-TATA  TCTTAGTT-----TTTAG-TATA  TCTTAGTT-----TTTAG-TATA  TCTTAGTT-----TTTAG-TATA  TCTTAGTT-----TTTAG-TATA  TCTTAGTT-----TTTAA-GATA  TCTTAGTT-----TTTAG-TATA  TCTTAGTT-----TTTAG-TATA  TCTTAGTT-----TTTAG-TATA  TCTTAGTT-----TTTAG-TATA  TCTTTGTTTTTAGTTTAG-TATA  TCTTTGTTTTTAGTTTAG-TATA  TCTTTGTTTTTAGTTTAG-TATA  TCTTTGTTTTTAGTTTAG-TATA  TCTTTGTTTTTAGTTTAG-TATA  TCTTTGTTTTTAGTTTAG-TATA  TCTTTGTTTTTAGTTTAG-TATA  TCTTTGTTTTTAGTTTAG-TATA  TCTTTGTTTTTAGTTTAG-TATA  TCTTTGTTTTTAGTTTAG-TATA  TCTTTGTTTTTAGTTTAG-TATA  TCTTTGTTTTTAGTTTAG-TATA  TCTTTGTT-----TTTAG-TATA  TCTTAGTT-----TTTAA-TATA  TCTTAGTT-----TTTAG-TATA  TCTTAGTT-----TTTAG-TATA  TCTTAGTT-----TTTAG-TATA  TCTTAGTT-----TTTAG-TATA  TCTTAGTT-----TTTTG-TATA  TCTTAGTT-----TTTAG-TATA  TCTTAGTT-----TTTAG-TTTA  TCTTAGTT-----TTTAG-TATA  TCTTAGTT-----TTTAG-TATA  TCTTAGTT-----TTTAG-TATA | Ssm p | Triticeae |
| 66 | Schedonorus arundinaceus Schedonorus arundinaceus[C] Schedonorus pratensis  Festuca altissima  Festuca ovina  Lolium multiflorum  Lolium perenne  Dactylis glomerata  Helictochloa hookeri  Deschampsia antarctica  Poa palustris  Phleum alpinum  Briza sp  Puccinellia nuttalliana  Ammophila breviligulata  Agrostis stolonifera  Hierochloe odorata  Anthoxanthum odoratum  Phalaris arundinacea  Torreyochloa  Avena sativa  Trisetum cernuum  Aegilops cylindrica  Aegilops geniculata  Aegilops speltoides  Aegilops tauschii  Triticum aestivum  Triticum monococcum aegilopoides Triticum monococcum (modified) Triticum urartu (modified)  Hordeum jubatum  Hordeum vulgare  Hordeum vulgare spontaneum Secale cereale  Bromus vulgaris  Brachypodium distachyon  Diarrhena obovata  Melica mutica  Melica subulata  Oryzopsis asperifolia Achnatherum hymenoides Ampelodesmos mauritanica Piptochaetium avenaceum  Phaenosperma globosum Brachyelytrum aristosum  Bambusa bambosa | 50273  IGS |  | TTTTTTATTTTATTCT  TTTTTTATGCGATTCA  TTTTTTATTTTATTCT  TTTTTTATTTTATTCT  TTTTTTATTTTATTCT  TTTTTTATTTTATTCT  TTTTTTATTTTATTCT  TTTTTTATTTTATTCT  TTTTTTATTTTATTCT  TTTTTTATTTTATTCT  TTTTT----TTATTCT  TTTTT----TTATTCT  TGTTTTATTTTATTCT  -TTTT----TTATTCT  TTTTTTATTTTATTCT  TTTTTTATTTTATTCT  TTTTTTATTTTATTCT  TTTTTTATTGTATTCT  ---TTTTTTTTATTCT  TTTTTTATTTTATTCT  TTTTTTATTTTATTCT  -TTTTTATTTTATTCT  TTTTTTATTTGATTCT  TTTTTTATTTGATTCT  TTTTTTATTTGATTCT  TTTTTTATTTGATTCT  TTTTTTATTTGATTCT  -TTTTTATTTGATTCT  TTTTTTATTTGATTCT  TTTTTTATTTGATTCT  TTTTTTATTTGATTCT  TTTTTTATTTGATTCT  TTTTTTATTTGATTCT  TTTTTTATTTGATTCT  -TTTTTATTTGATTCT  TTTTTTATTTGATTCT  TTTTTTATTTGATTCT  TTTTTTATTCGATTCT  TTTTTTATTCGATTCT  TTTTTTATTCGATTCT  TTTTTTATTCGATTCT  TTTTTTATTCGATTCT  TTTTTTATTCGATTCT  TTTTTTATTCGATTCT  TTTTTTATTCGATTCC  TTTTTTATTCGATTCC |  | Coleanthinae+Poeae |
| 67 | Schedonorus arundinaceus Schedonorus arundinaceus[C] Schedonorus pratensis  Festuca altissima  Festuca ovina  Lolium multiflorum  Lolium perenne  Dactylis glomerata  Helictochloa hookeri  Deschampsia antarctica  Poa palustris  Phleum alpinum  Briza sp  Puccinellia nuttalliana  Ammophila breviligulata  Agrostis stolonifera  Hierochloe odorata  Anthoxanthum odoratum  Phalaris arundinacea  Torreyochloa  Avena sativa  Trisetum cernuum  Aegilops cylindrica  Aegilops geniculata  Aegilops speltoides  Aegilops tauschii  Triticum aestivum  Triticum monococcum aegilopoides Triticum monococcum (modified) Triticum urartu (modified)  Hordeum jubatum  Hordeum vulgare  Hordeum vulgare spontaneum Secale cereale  Bromus vulgaris  Brachypodium distachyon  Diarrhena obovata  Melica mutica  Melica subulata  Oryzopsis asperifolia Achnatherum hymenoides Ampelodesmos mauritanica Piptochaetium avenaceum  Phaenosperma globosum Brachyelytrum aristosum  Bambusa bambosa | 50539  IGS |  | ATTCGAGGATCTTAC  ATTG---GACCTTCT  ATTCGAGGATCTTAC  ATTCGAGGATCTTAC  ATTCAAGGATCTTAC  ATTCGAGGATCTTAC  ATTCGAGGATCTTAC  ATTCGAGGATCTTAC  ATTCGAGGATCTTAT  ATTCGAGGATCTTAC  ATTCGAGGATCTTAC  ATTCGAGGATCTTAC  ATTC---GATCTTAC  ATTCGAGGATCTTAC  ATTC---GATCTTAC  ATTC---GATCTTAC  ATTC---GATCTTAC  ATTC---GATCTTAC  ATTC---GATCTTAC  ATTC---GATCTTAC  ATTC---GATCTTAC  ATTC---GATCTTAC  ATTC---GATCTTAC  ATTC---GATCTTAC  ATTC---GATCTTAC  ATTC---GATCTTAC  ATTC---GATCTTAC  ATTC---GATCTTAC  ATTC---GATCTTAC  ATTC---GATCTTAC  ATTC---GATCTTAC  ATTC---GATCTTAC  ATTC---GATCTTAC  ATTC---GATCTTAC  ATTC---GATCTTAC  ATTC---GATCTTAC  ATTC---GATCTTAC  ATTC---GATCTTAC  ATTC---GATCTTAC  ATTC---GATCTTAC  ATTC---GATCTTAC  ATTC---GATCTTAC  ATTC---GATCTTAC  ATTC---GATCTTAC  ATTT---GATCTTCC  ATTC---GATCTTCC |  | Homoplasy |
| 68 | Schedonorus arundinaceus Schedonorus arundinaceus[C] Schedonorus pratensis  Festuca altissima  Festuca ovina  Lolium multiflorum  Lolium perenne  Dactylis glomerata  Helictochloa hookeri  Deschampsia antarctica  Poa palustris  Phleum alpinum  Briza sp  Puccinellia nuttalliana  Ammophila breviligulata  Agrostis stolonifera  Hierochloe odorata  Anthoxanthum odoratum  Phalaris arundinacea  Torreyochloa  Avena sativa  Trisetum cernuum  Aegilops cylindrica  Aegilops geniculata  Aegilops speltoides  Aegilops tauschii  Triticum aestivum  Triticum monococcum aegilopoides Triticum monococcum (modified) Triticum urartu (modified)  Hordeum jubatum  Hordeum vulgare  Hordeum vulgare spontaneum Secale cereale  Bromus vulgaris  Brachypodium distachyon  Diarrhena obovata  Melica mutica  Melica subulata  Oryzopsis asperifolia Achnatherum hymenoides Ampelodesmos mauritanica Piptochaetium avenaceum  Phaenosperma globosum Brachyelytrum aristosum  Bambusa bambosa | 51049  Ycf3 intron 2 | Ycf3 intron 2 | AAGCATAGATAGATCTAGAGACTTC  AAGCATAGATAGATCTAGAGACTTC  AAGCATAGATAGATCTAGAGACTTC  AAGCATAGATAGATCTAGAGACTTC  AAGCATAGATAGATCTAGAGACTTC  AAGCATAGATAGATCTAGAGACTTC  AAGCATAGATAGATCTAGAGACTTC  AAGCATAGATAGATCTAGAGACTTC  AAGCATAGATAGATTTAGAGACTTC  AAGCATAGATAGATCTAGAGACTTC  AAGCATAGATAGATCTAGAGACTTC  AAGCATAGATAGATCTAGAGACTTC  AAGCATAGATAGATCTAGAGCGTTC  AAGCATAGATAGATCTAGATACTTC  AAGCATAGATAGATCTAGAGCCTTC  AAGCAGAGATAGATCTAGAGCCTTC  AAGCATAGATAGATCTAGAGCCTTC  AAGCATAGATAGATCTAGAGTCTTC  AAGCATAGATAGATCTAGAGCCTTC  AAGCATAGATAGATCTAGAGCCTTC  AAGCATAGATAGATCTAGAGCCTTC  AAGCATAGATAGATCTAGAGCCTTC  AAGCATAGATAGATATAGAGCCTTC  AAGCATAGATAGATATAGAGCCTTC  AAGCATAGATAGATATAGAGCCTTC  AAGCATAGATAGATATAGAGCCTTC  AAGCATAGATAGATATAGAGCCTTC  AAGCATAGATAGATATAGAGCCTTC  AAGCATAGATAGATATAGAGCCTTC  AAGCATAGATAGATATAGAGCCTTC  AAGCATAGATAGATATAGAGCCTTC  AAGCATAGATAGATATAGAGCCTTC  AAGCATAGATAGATATAGAGCCTTC  AAGCATAGATAGATATAGAGCCTTC  AAGCATAGATAGATATAGAGCCTTC  AAGCATAGATAGATCGAGAGCTTTC  AAGCATAGATAGATCTAGAGCCTTC  AGGCATAGATAGATCTATAGCCTTC  AGGCATAGATAGATCTATAGCCTTC  AACCAT------------AGCCTTC  AACCAT------------CGCCTTC  AACCAT------------AGCCTTC  AACCAT------------AGTCTTC  AAGCATAGATAGATCTATAGCCTTC  AAGCATAGATAGACCTATAGCCTTC  AAGCATAGATAGACCTATAGCCTTC |  | Stipeae |
| 69 | Schedonorus arundinaceus Schedonorus arundinaceus[C] Schedonorus pratensis  Festuca altissima  Festuca ovina  Lolium multiflorum  Lolium perenne  Dactylis glomerata  Helictochloa hookeri  Deschampsia antarctica  Poa palustris  Phleum alpinum  Briza sp  Puccinellia nuttalliana  Ammophila breviligulata  Agrostis stolonifera  Hierochloe odorata  Anthoxanthum odoratum  Phalaris arundinacea  Torreyochloa  Avena sativa  Trisetum cernuum  Aegilops cylindrica  Aegilops geniculata  Aegilops speltoides  Aegilops tauschii  Triticum aestivum  Triticum monococcum aegilopoides Triticum monococcum (modified) Triticum urartu (modified)  Hordeum jubatum  Hordeum vulgare  Hordeum vulgare spontaneum Secale cereale  Bromus vulgaris  Brachypodium distachyon  Diarrhena obovata  Melica mutica  Melica subulata  Oryzopsis asperifolia Achnatherum hymenoides Ampelodesmos mauritanica Piptochaetium avenaceum  Phaenosperma globosum Brachyelytrum aristosum  Bambusa bambosa | 51294  Ycf3 in tron 2 | Ycf3 in tron 2 | ATATCATGG-----GATAAGTAAG  ATATCATGG-----GATAAGTAAG  ATATCATGG-----GATAAGTAAG  ATATCATGG-----GATAAGTAAG  ATATCATGG-----GATAAGTAAG  ATATCATGG-----GATAAGTAAG  ATATCATGG-----GATAAGTAAG  TTATCATGG-----GATAAGTAAG  ATATCATGG-----GATAAGTAAG  ATATCATGG-----GATAAGTAAG  ATATCATGG-----GATAAGTAAG  ATATCATGG-----GATAAGTAAG  ATATCATGGGATAAGATAAGTAAG  ATATCATGG-----GATAAGTAAG  ATATCATGGGATAAGATAAGTAAG  ATATCATGGGATAAGATAAGTAAG  ATATCATGGGATAAGATAAGTAAG  ATATCATGGGATAAGATAAGTAAG  ATATCATGGGATAAGATAAGTAAG  ATATCATGGGATAAGATAAGTAAG  ATATCGTGGGATAAGATAAGTAAG  ATATCATGGGATAAGATAAGTAAG  ATATCATGG-----GATAAGTAAG  ATATCATGG-----GATAAGTAAG  ATATCATGG-----GATAAGTAAG  ATATCATGG-----GATAAGTAAG  ATATCATGG-----GATAAGTAAG  ATATCATGG-----GATAAGTAAG  ATATCATGG-----GATAAGTAAG  ATATCATGG-----GATAAGTAAG  ATATCATGG-----GATAAGTAAG  ATATCATGG-----GATAAGTAAG  ATATCATGG-----GATAAGTAAG  ATATCATGG-----GATAAGTAAG  ATATCATGG-----GATAAGTAAG  ATATCATGG-----GATAAGTAAG  ATATCATGG-----GATAAGTAAG  ATATCATGG-----GATAAGTAAG  ATATCATGG-----GATAAGTAAG  ATATCATGG-----GATAAGTAAG  ATATCATGG-----GATAAGTAAG  ATATCATGG-----GATAAGTAAG  ATATCATGG-----GATAAGTAAG  ATATCATGG-----GATAAGTAAG  ATATCATGG-----GATAAGTAAG  ATATCATGG-----GATAAGTAAG |  | Poeae clade 1 |
| 70 | Schedonorus arundinaceus Schedonorus arundinaceus[C] Schedonorus pratensis  Festuca altissima  Festuca ovina  Lolium multiflorum  Lolium perenne  Dactylis glomerata  Helictochloa hookeri  Deschampsia antarctica  Poa palustris  Phleum alpinum  Briza sp  Puccinellia nuttalliana  Ammophila breviligulata  Agrostis stolonifera  Hierochloe odorata  Anthoxanthum odoratum  Phalaris arundinacea  Torreyochloa  Avena sativa  Trisetum cernuum  Aegilops cylindrica  Aegilops geniculata  Aegilops speltoides  Aegilops tauschii  Triticum aestivum  Triticum monococcum aegilopoides Triticum monococcum (modified) Triticum urartu (modified)  Hordeum jubatum  Hordeum vulgare  Hordeum vulgare spontaneum Secale cereale  Bromus vulgaris  Brachypodium distachyon  Diarrhena obovata  Melica mutica  Melica subulata  Oryzopsis asperifolia Achnatherum hymenoides Ampelodesmos mauritanica Piptochaetium avenaceum  Phaenosperma globosum Brachyelytrum aristosum  Bambusa bambosa | 51946  Ycf3 intron 1 | Ycf3 in tron 1 | CGAAA------TAATCT  CGAAA------TAATCT  CGAAA------TAATCT  CGAAA------TAATCT  CGAAA------TAATCT  CGAAA------TAATCT  CGAAA------TAATCT  CGAAA------TAATCT  CGAAA------TAATCT  CGAAA------TAATCT  CGAAA------TAATCT  CGAAA------TAATCT  CGAAA------TAATCT  CGAAA------TAATCT  CGAAA------TAATCT  CGAAA------TAATCT  CGAGA------TAATCT  CGAGA------TAGTCT  CGAAA------TAATCT  CGAAA------TAATCT  CGAAA------TAATCT  CGAAA------TAATCT  CGAAA------TAATCT  CGAAA------TAATCT  CGAAA------TAATCT  CGAAA------TAATCT  CGAAA------TAATCT  CGAAA------TAATCT  CGAAA------TAATCT  CGAAA------TAATCT  CGAAA------TAATCT  CGAAATAATCTTAATCT  CGAAATAATCTTAATCT  CGAAA------TAATCT  CGAAA------TAATCT  CGAAA------TAATCT  CGAAA------TAATCT  CGAAA------AAATCT  CGAAA------AAATCT  CGAAA------TAATCT  CGAAA------TAATCT  CGAAA------TAATCT  CGAAA------TAATCT  CGAAA------AAATCT  CGAAA------TAATCT  CGAAA------TAATCT |  | Hordeum vulgare |
| 71 | Schedonorus arundinaceus Schedonorus arundinaceus[C] Schedonorus pratensis  Festuca altissima  Festuca ovina  Lolium multiflorum  Lolium perenne  Dactylis glomerata  Helictochloa hookeri  Deschampsia antarctica  Poa palustris  Phleum alpinum  Briza sp  Puccinellia nuttalliana  Ammophila breviligulata  Agrostis stolonifera  Hierochloe odorata  Anthoxanthum odoratum  Phalaris arundinacea  Torreyochloa  Avena sativa  Trisetum cernuum  Aegilops cylindrica  Aegilops geniculata  Aegilops speltoides  Aegilops tauschii  Triticum aestivum  Triticum monococcum aegilopoides Triticum monococcum (modified) Triticum urartu (modified)  Hordeum jubatum  Hordeum vulgare  Hordeum vulgare spontaneum Secale cereale  Bromus vulgaris  Brachypodium distachyon  Diarrhena obovata  Melica mutica  Melica subulata  Oryzopsis asperifolia Achnatherum hymenoides Ampelodesmos mauritanica Piptochaetium avenaceum  Phaenosperma globosum Brachyelytrum aristosum  Bambusa bambosa | 53208  Ycf3 intron 1 | Ycf3 in tron 1 | TTGATTCCTTTCCTATT  TTGATTCCTTTCCTATT  TTGATTCCTTTCCTATT  TTGATTCCTTTCCTATT  TTGATTCCTTTCCTATT  TTGATTCCTTTCCTATT  TTGATTCCTTTCCTATT  TTGATTCCTTTCCTATT  TTGATTCCTTTTCTATT  TTGATTCC-TTCCTATT  TTGATTCCTTTCCTATT  TTGATTCCTTTTCTATT  TTGATTCCTTTCCTATT  TTGATTCCTTTTCTATT  TTGATTCCTTTCCTATT  TTGATTCCTTTCCTATT  TTGATTCCTTTCCTATT  TTGATTCCTTTCCTATT  TTGATTCCTTTACTATT  TTGATTCCTTTCCTATT  TTGATTCCTTTCCTATT  TTGATTCCTTTCCTATT  TTGATTC-----CTATT  TTGATTC-----CTATT  TTGATTC-----CTATT  TTGATTC-----CTATT  TTGATTC-----CTATT  TTGATTC-----CTATT  TTGATTC-----CTATT  TTGATTC-----CTATT  TTGATTC-----CTATT  TTGATTC-----CTATT  TTGATTC-----CTATT  TTGATTC-----CTATT  TTGATTC-----CTATT  TTGATTC-----CTATT  TTGATTC-----CTATT  TTGATTC-----CTATT  TTGATTC-----CTATT  TTGATTC-----CTATT  TTGATTC-----CTATT  TTGATTC-----CTATT  TTGATTC-----CTATT  TTGATTC-----CTATT  TTGATTG-----CTATT  TTGATTC-----CTATT |  | Poeae |
| 72 | Schedonorus arundinaceus Schedonorus arundinaceus[C] Schedonorus pratensis  Festuca altissima  Festuca ovina  Lolium multiflorum  Lolium perenne  Dactylis glomerata  Helictochloa hookeri  Deschampsia antarctica  Poa palustris  Phleum alpinum  Briza sp  Puccinellia nuttalliana  Ammophila breviligulata  Agrostis stolonifera  Hierochloe odorata  Anthoxanthum odoratum  Phalaris arundinacea  Torreyochloa  Avena sativa  Trisetum cernuum  Aegilops cylindrica  Aegilops geniculata  Aegilops speltoides  Aegilops tauschii  Triticum aestivum  Triticum monococcum aegilopoides Triticum monococcum (modified) Triticum urartu (modified)  Hordeum jubatum  Hordeum vulgare  Hordeum vulgare spontaneum Secale cereale  Bromus vulgaris  Brachypodium distachyon  Diarrhena obovata  Melica mutica  Melica subulata  Oryzopsis asperifolia Achnatherum hymenoides Ampelodesmos mauritanica Piptochaetium avenaceum  Phaenosperma globosum Brachyelytrum aristosum  Bambusa bambosa | 52535  Ycf3 intron 1 | Ycf3 in tron 1 | AGCGTGGGT----TTGCTTTAC  AGCGTGGGT----TTGCTTTAC  AGCGTGGGT----TTGCTTTAC  AGCGTGGGT----TTGCTTTAC  AGCGTGGGT----TTGTTTTAC  AGCGTGGGT----TTGCTTTAC  AGCGTGGGT----TTGCTTTAC  AGCGTGGGT----TTGCTTTAC  AGCGTGGGT----TTGCTTTAA  AGCGTGGGT----TTGTTTTAC  AGCGTGGGT----TTGCTTTAC  AGCGTGGGT----TTGCTTTAC  AGCGTGGGT----TTTCTTTAC  AGCGTGGGT----TTGCTTTAC  AGCGTGGGT----TTGCTTTAC  AGCGTGGGT----TTGCTTTAC  AGCGTGGGT----TTGCTTTAC  AGCGTGGGT----TTGCTTTAC  AGCGTGGGT----TTGCTTTAC  AGCGTGGGT----TTGCTTTAC  AGCGTGGGT----TTGCTTTAC  AGCGTGGGT----TTGCTTTAC  AGCGTGGGTTTGTTTGCTTTAC  AGCGTGGGTTTGTTTGCTTTAC  AGCGTGGGTTTGTTTGCTTTAC  AGCGTGGGTTTGTTTGCTTTAC  AGCGTGGGTTTGTTTGCTTTAC  AGCGTGGGTTTGTTTGCTTTAC  AGCGTGGGTTTGTTTGCTTTAC  AGCGTGGGTTTGTTTGCTTTAC  AGCGTGGGTTTGTTTGCTTTAC  AGCGTGGGTTTGCTTGCTTTAC  AGCGTGGGTTTGCTTGCTTTAC  AGCGTGGGTTTGTTTGCTTTAC  AGCGTGGGTTTGTTTGCTTTAC  AGCGTGGGT----TTGCTTTAC  AGCGTGGGT----TTGCTTTAC  AGCGTGGGT----TTGCTTTAC  AGCGTGGGT----TTGCTTTAC  AGCGTGGGT----TTGCTTTAC  AGCGTGGGT----TTGCTTTAC  AGCGTGGGT----TTGCTTTAC  AGCGCGGGT----TTGCTTTAC  AGCGTGGGT----TTGCTTTAC  AGCGTGGGT----TTCCTTTAA  AGCGTGGGT----TTCCTTTAC |  | Bromeae+Triticeae |
| 73 | Schedonorus arundinaceus Schedonorus arundinaceus[C] Schedonorus pratensis  Festuca altissima  Festuca ovina  Lolium multiflorum  Lolium perenne  Dactylis glomerata  Helictochloa hookeri  Deschampsia antarctica  Poa palustris  Phleum alpinum  Briza sp  Puccinellia nuttalliana  Ammophila breviligulata  Agrostis stolonifera  Hierochloe odorata  Anthoxanthum odoratum  Phalaris arundinacea  Torreyochloa  Avena sativa  Trisetum cernuum  Aegilops cylindrica  Aegilops geniculata  Aegilops speltoides  Aegilops tauschii  Triticum aestivum  Triticum monococcum aegilopoides Triticum monococcum (modified) Triticum urartu (modified)  Hordeum jubatum  Hordeum vulgare  Hordeum vulgare spontaneum Secale cereale  Bromus vulgaris  Brachypodium distachyon  Diarrhena obovata  Melica mutica  Melica subulata  Oryzopsis asperifolia Achnatherum hymenoides Ampelodesmos mauritanica Piptochaetium avenaceum  Phaenosperma globosum Brachyelytrum aristosum  Bambusa bambosa | 52866  IGS |  | TTTGATTC------TTAGAAAT  TTTGATTC------TTAGAAAT  TTTGATTC------TTAGAAAT  TTCGATTC------TTAGAAAT  TTCGATTC------TTAGAAAT  TTTGATTC------TTAGAAAT  TTTGATTC------TTAGAAAT  CTCGATTC------TTAGAAAT  TCCGATTC------TTAGAAAT  TTCGATTC------TTAGAAAT  TTCGATTT------TTATAAAT  TTCGATTC------TTATAAAT  TTCGATTC------TTATAAAT  TTTGATTC------TTATAAAT  TTCGATTC------TTATAAAT  TTCGATTC------TTATAAAT  TTCGATTC------TTATAAAT  TTTTATTC------TTATAAAT  TTCGATTT------TTATAAAT  TTCGATTC------TTATAAAT  TTCGATTC------TTATAAAT  TTCGATTC------TTATAAAT  TTCGATTCTTATAATTATAAAT  TTCGATTCTTATAATTATAAAT  TTCGATTCTTATAATTATAAAT  TTCGATTCTTATAATTATAAAT  TTCGATTCTTATAATTATAAAT  TTCGATTCTTATAATTATAAAT  TTCGATTCTTATAATTATAAAT  TTCGATTCTTATAATTATAAAT  TTCGATTCTTATAATTATAAAT  TTCGATTCTTATAATTATAAAT  TTCGATTCTTATAATTATAAAT  TTCGATTCTTATAATTATAAAT  TTCGATTCTTATAATTATAAAT  TTTGATTC------TTATAACT  TTCGATTC------TTATAAAT  TTGGATTC------TTATAAAT  TTGGATTC------TTATAAAT  TTCGATTC------TTATAAAT  TTCGATTC------TTATAAAT  TTCGATTC------TTATAAAT  TTCGATTC------TTATAAAT  TTCGATTC------TTATAAAT  TTTGATTC------TTATAAAT  TTCGATTC------TTATAAAT | ssmp | Bromeae+Triticeae |
| 74 | Schedonorus arundinaceus Schedonorus arundinaceus[C] Schedonorus pratensis  Festuca altissima  Festuca ovina  Lolium multiflorum  Lolium perenne  Dactylis glomerata  Helictochloa hookeri  Deschampsia antarctica  Poa palustris  Phleum alpinum  Briza sp  Puccinellia nuttalliana  Ammophila breviligulata  Agrostis stolonifera  Hierochloe odorata  Anthoxanthum odoratum  Phalaris arundinacea  Torreyochloa  Avena sativa  Trisetum cernuum  Aegilops cylindrica  Aegilops geniculata  Aegilops speltoides  Aegilops tauschii  Triticum aestivum  Triticum monococcum aegilopoides Triticum monococcum (modified) Triticum urartu (modified)  Hordeum jubatum  Hordeum vulgare  Hordeum vulgare spontaneum Secale cereale  Bromus vulgaris  Brachypodium distachyon  Diarrhena obovata  Melica mutica  Melica subulata  Oryzopsis asperifolia Achnatherum hymenoides Ampelodesmos mauritanica Piptochaetium avenaceum  Phaenosperma globosum Brachyelytrum aristosum  Bambusa bambosa | 52984  IGS |  | CAAATTGTAATTGTATCCTT  CAAATTGTAATTGTATCCTT  CAAATTGTAATTGTATCCTT  CAAATTGTAATTGTATCCTT  CAAATTGTAATTGTATCCTT  CAAATTGTAATTGTATCCTT  CAAATTGTAATTGTATCCTT  CAAATTGTAATTGTATCCTT  CAAATTGTAATTTTATCCTT  CAAATTGTAATTGTATCCTT  CAAATTGTAATTGTATCCTT  CAAATTGTAATTGTATCCTT  CAAATTGT------ATCCTT  CAAATTGTAATTGTATCCTT  C----TGT------ATCCTT  C----TGT------ATCCTT  CAAATTGT------ATCCTT  CAAATTGT------ATCCTT  CAAATTGT------ATCCTT  CAAATTGT------ATCCTT  CAAATTGT------ATCCTT  CAAATTGT------ATCCTT  CAAATTGT------ATCCTT  CAAATTGT------ATCCTT  CAAATTGT------ATCCTT  CAAATTGT------ATCCTT  CAAATTGT------ATCCTT  CAAATTGT------ATCCTT  CAAATTGT------ATCCTT  CAAATTGT------ATCCTT  CAAATTGT------ATCCTT  CAAATTGT------ATCCTT  CAAATTGT------ATCCTT  CAAATTGT------ATCCTT  CAAATTGT------ATCCTT  TTAATTTT------TTCCTT  CAAATTGT------ATCCTT  CAAATTGT------ATCCTT  CAAATTGT------ATCCTT  CAAATTGT------ATCCTT  CAAATTGT------ATCCTT  CAAATTGT------ATCCTT  CAAATTGT------A-----  CAAATTGT------ATCCTT  CAAATTGT------CTCCTT  CAAATTGT------GTCCTT | ssmp | Poeae clade 2 |
| 75 | Schedonorus arundinaceus Schedonorus arundinaceus[C] Schedonorus pratensis  Festuca altissima  Festuca ovina  Lolium multiflorum  Lolium perenne  Dactylis glomerata  Helictochloa hookeri  Deschampsia antarctica  Poa palustris  Phleum alpinum  Briza sp  Puccinellia nuttalliana  Ammophila breviligulata  Agrostis stolonifera  Hierochloe odorata  Anthoxanthum odoratum  Phalaris arundinacea  Torreyochloa  Avena sativa  Trisetum cernuum  Aegilops cylindrica  Aegilops geniculata  Aegilops speltoides  Aegilops tauschii  Triticum aestivum  Triticum monococcum aegilopoides Triticum monococcum (modified) Triticum urartu (modified)  Hordeum jubatum  Hordeum vulgare  Hordeum vulgare spontaneum Secale cereale  Bromus vulgaris  Brachypodium distachyon  Diarrhena obovata  Melica mutica  Melica subulata  Oryzopsis asperifolia Achnatherum hymenoides Ampelodesmos mauritanica Piptochaetium avenaceum  Phaenosperma globosum Brachyelytrum aristosum  Bambusa bambosa | 53117  IGS |  | TTTCAAAG-----TCATTAAT  TTTCAAAG-----TCATTAAT  TTTCAAAG-----TCATTAAT  TTTCAAAG-----TCATTAAT  TTTCAAAG-----TCATTA--  TTTCAAAG-----TCATTAAT  TTTCAAAG-----TCATTAAT  TTTCAAAG-----CCATTAAT  TTTCAAAG-----TTATTAAT  TTTCAAAG-----TCATTAAT  TTTCAAAG-----TCATTAAT  TTTCAAAG-----TCATTAAT  TTTCAAAG-----TCATTAAT  TTTCAAAG-----TCATTAAT  TTTCAAAG-----TCATTAAT  TTTCAAAG-----TCATTAAT  TTTCAAAG-----TCATTAAT  TTTGAAAG-----TAATTACT  TTTCAAAG-----TCATTAAT  TTTCAAAG-----TCATTAAT  TTGCAAAG-----TCATTAAT  TTGCAAAG-----TCATTAAT  TTTCAAAG-----TTATTAAT  TTTCAAAG-----TTATTAAT  TTTCAAAGTTATTTTATTAAT  TTTCAAAG-----TTATTAAT  TTTCAAAGTTATTTTATTAAT  TTTCAAAG-----TTA-AAAT  TTTCAAAG-----TTATTAAT  TTTCAAAG-----TTATTAAT  TTTCAAAG-----TTATTAAT  TTTCAAAG-----TTATTAAT  TTTCAAAG-----TTATTAAT  TTTCAAAG-----TTATTAAT  TTTCAAAG-----TTCTTAAT  TTTCAAAG-----TTATTAAT  TTTCAAAG-----TCATTAAT  TTTCAAAG-----TCATTAAT  TTTCAAAG-----TCATTAAT  TTTCAAAA-----TCATTAAT  TTTCAAAA-----TCATTAAT  TTTCAAAA-----TCATTAAT  TTTCAAAA-----TCATTAAT  TTTCAAAG-----TCTTTAAT  TTTCAAAG-----TAATTAAT  TTTCAAAG-----TCATTAAT | ssmp | Aegilops speltoides+Triticum aestivum |
| 76 | Schedonorus arundinaceus Schedonorus arundinaceus[C] Schedonorus pratensis  Festuca altissima  Festuca ovina  Lolium multiflorum  Lolium perenne  Dactylis glomerata  Helictochloa hookeri  Deschampsia antarctica  Poa palustris  Phleum alpinum  Briza sp  Puccinellia nuttalliana  Ammophila breviligulata  Agrostis stolonifera  Hierochloe odorata  Anthoxanthum odoratum  Phalaris arundinacea  Torreyochloa  Avena sativa  Trisetum cernuum  Aegilops cylindrica  Aegilops geniculata  Aegilops speltoides  Aegilops tauschii  Triticum aestivum  Triticum monococcum aegilopoides Triticum monococcum (modified) Triticum urartu (modified)  Hordeum jubatum  Hordeum vulgare  Hordeum vulgare spontaneum Secale cereale  Bromus vulgaris  Brachypodium distachyon  Diarrhena obovata  Melica mutica  Melica subulata  Oryzopsis asperifolia Achnatherum hymenoides Ampelodesmos mauritanica Piptochaetium avenaceum  Phaenosperma globosum Brachyelytrum aristosum  Bambusa bambosa | 53208  IGS |  | GGTTTC-----TTTATTT  GGTTTC-----TTTATTT  GGTTTC-----TTTATTT  GGTTTC-----TTTATTT  GGTTTC-----TTTATTT  GGTTTC-----TTTATTT  GGTTTC-----TTTATTT  GGTTTC-----TTTATTT  GGTTTC-----TTTATTT  GGTTTC-----TTTATTT  GGTTTC-----TTTATTT  GGTTTC-----TTTATTT  GGTTTC-----TTTATTT  GGTTTC-----TTTATTT  GGTTTC-----TTTATTT  GGTTTC-----TTTATTT  GGTTTC-----TTTATTT  GGTTTC-----TTTCTTT  GGTTTC-----TTTATTT  GGTTTC-----TTTATTT  GGTTTC-----TTTATTT  GGTTTC-----TTTATTT  GGTTTC-----TTTATTT  GGTTTC-----TTTATTT  GGTTTCTTTATTTTATTT  GGTTTC-----TTTATTT  GGTTTCTTTATTTTATTT  GGTTTC-----TTTATTT  GGTTTC-----TTTATTT  GGTTTC-----TTTATTT  GGTTTC-----TTTATTT  GGTTTC-----TTTATTT  GGTTTC-----TTTATTT  GGTTTC-----TTTATTT  GGTTTC-----TTTATTT  GGTTTC-----TTTATTT  GGTTTC-----TTTATTT  GGTTTC-----TTCATTT  GGTTTC-----TTCATTT  GGTTTC-----TTTATTT  GGTTTC-----TTTATTT  GGTTTC-----TTTATTT  GGTTTC-----TTTATTT  GGTTTC-----TTTTTTT  GGTTTC-----CTTATTT  GGTTCC-----TTTATTT | ssmp | Aegilops speltoides+Triticum aestivum |
| 77 | Schedonorus arundinaceus Schedonorus arundinaceus[C] Schedonorus pratensis  Festuca altissima  Festuca ovina  Lolium multiflorum  Lolium perenne  Dactylis glomerata  Helictochloa hookeri  Deschampsia antarctica  Poa palustris  Phleum alpinum  Briza sp  Puccinellia nuttalliana  Ammophila breviligulata  Agrostis stolonifera  Hierochloe odorata  Anthoxanthum odoratum  Phalaris arundinacea  Torreyochloa  Avena sativa  Trisetum cernuum  Aegilops cylindrica  Aegilops geniculata  Aegilops speltoides  Aegilops tauschii  Triticum aestivum  Triticum monococcum aegilopoides Triticum monococcum (modified) Triticum urartu (modified)  Hordeum jubatum  Hordeum vulgare  Hordeum vulgare spontaneum Secale cereale  Bromus vulgaris  Brachypodium distachyon  Diarrhena obovata  Melica mutica  Melica subulata  Oryzopsis asperifolia Achnatherum hymenoides Ampelodesmos mauritanica Piptochaetium avenaceum  Phaenosperma globosum Brachyelytrum aristosum  Bambusa bambosa | 53260  IGS |  | CA-T--------AAGAAGAA---  CA-T--------AAGAAGAA---  CA-T--------AAGAAGAATTC  CA-T--------AAGAAGAATTC  AA-T--------AAGAAGAATTC  CA-T--------AAGAAGAATTC  CA-T--------AAGAAGAATTC  AA-T--------AAGAAGAATTC  GA-T--------AAGAAGAATTC  AA-T--------AAGAAGAATTC  AATT--------AAGAAGAATTC  AA-T--------AAGAAGAATTC  AA-T--------AAGAAGAATTC  AA-T--------AAGAAGAATTT  AA-T--------AAGAAGAATTC  AA-T--------AAGAAGAATTC  AATT--------AAGAAGAATTC  AATT--------AAGAAGAATTC  AA-T--------AAGAAGAATTC  AA-T--------AAGAAGAATTC  AA-TAAGAAGAAAAGAAGAATTC  AA-TAAGAAGAAAAGAAGAATTC  AA-T--------AAGAAGAATTC  AA-T--------AAGAAGAATTC  AA-T--------AAGAAGAATTC  AA-T--------AAGAAGAATTC  AA-T--------AAGAAGAATTC  AA-T--------AAGAAGAATTC  AA-T--------AAGAAGAATTC  AA-T--------AAGAAGAATTC  AA-T--------AAGAAGAATTC  AA-T--------AAGAAGAATTC  AA-T--------AAGAAGAATTC  AA-T--------AAGAAGAATTC  AA-T--------AAGAAGAATTC  AA-T--------AAGAAGAATTC  AA-T--------AAGAAGAATTC  AA-T--------AAGAAGAATTC  AA-T--------AAGAAGAATTC  AA-T--------AAGAAGAATTC  AA-T--------AAGAAGAATTC  AA-T--------AAGAAGAATTC  AA-T--------AAGAAGAATTC  AA-T--------AAGAAGAATTA  AATT--------AAGAAGAATTC  AA-T--------AAGAAGAATTC | ssmp | Aveninae+Koelerinae |
| 78 | Schedonorus arundinaceus Schedonorus arundinaceus[C] Schedonorus pratensis  Festuca altissima  Festuca ovina  Lolium multiflorum  Lolium perenne  Dactylis glomerata  Helictochloa hookeri  Deschampsia antarctica  Poa palustris  Phleum alpinum  Briza sp  Puccinellia nuttalliana  Ammophila breviligulata  Agrostis stolonifera  Hierochloe odorata  Anthoxanthum odoratum  Phalaris arundinacea  Torreyochloa  Avena sativa  Trisetum cernuum  Aegilops cylindrica  Aegilops geniculata  Aegilops speltoides  Aegilops tauschii  Triticum aestivum  Triticum monococcum aegilopoides Triticum monococcum (modified) Triticum urartu (modified)  Hordeum jubatum  Hordeum vulgare  Hordeum vulgare spontaneum Secale cereale  Bromus vulgaris  Brachypodium distachyon  Diarrhena obovata  Melica mutica  Melica subulata  Oryzopsis asperifolia Achnatherum hymenoides Ampelodesmos mauritanica Piptochaetium avenaceum  Phaenosperma globosum Brachyelytrum aristosum  Bambusa bambosa | 53544  IGS |  | TTCC--ATAAT  TTCC--ATAAT  TTCC--ATAAT  TTCC--ATAAT  TTCC--ATAAT  TTCC--ATAAT  TTCC--ATAAT  TTCC--ATAAT  TTCC--ATAAT  TTCC--ATAAT  TTTC--ATAAT  TTCC--ATAAT  TTCC--ATAAT  TTCC--ATAAT  TTCC--ATAAT  TTCC--ATAAT  TTCC--ATAAT  TTCC--ATAAT  TTCC--ATAAT  TTCC--ATAAT  TTCC--ATAAT  TTCC--ATAAT  TTCC--ATAAT  TTCC--ATAAT  TTCC--ATAAT  TTCC--ATAAT  TTCC--ATAAT  TTCC--ATAAT  TTCC--ATAAT  TTCC--ATAAT  TTCC--ATAAT  TTCC--ATAAT  TTCC--ATAAT  TTCC--ATAAT  TTCC--ATAAT  TGCC--ATAAT  TTCC--ATAAT  TTCCATATAAT  TTCCATATAAT  TTCC--ATAAT  TTCC--ATAAT  TTCC--ATAAT  TTCC--ATAAT  TTCC--ATAAT  TTCC--ATAAT  TTCC--ATAAT | Ssmp | Melica |
| 79 | Schedonorus arundinaceus Schedonorus arundinaceus[C] Schedonorus pratensis  Festuca altissima  Festuca ovina  Lolium multiflorum  Lolium perenne  Dactylis glomerata  Helictochloa hookeri  Deschampsia antarctica  Poa palustris  Phleum alpinum  Briza sp  Puccinellia nuttalliana  Ammophila breviligulata  Agrostis stolonifera  Hierochloe odorata  Anthoxanthum odoratum  Phalaris arundinacea  Torreyochloa  Avena sativa  Trisetum cernuum  Aegilops cylindrica  Aegilops geniculata  Aegilops speltoides  Aegilops tauschii  Triticum aestivum  Triticum monococcum aegilopoides Triticum monococcum (modified) Triticum urartu (modified)  Hordeum jubatum  Hordeum vulgare  Hordeum vulgare spontaneum Secale cereale  Bromus vulgaris  Brachypodium distachyon  Diarrhena obovata  Melica mutica  Melica subulata  Oryzopsis asperifolia Achnatherum hymenoides Ampelodesmos mauritanica Piptochaetium avenaceum  Phaenosperma globosum Brachyelytrum aristosum  Bambusa bambosa | 53738  IGS |  | A-----ATAATA-TCT  A-----ATAATA-TCT  A-----ATAATA-TCT  A-----ATAATA-TCT  A-----ATAATA-TCT  A-----ATAATA-TCT  A-----ATAATA-TCT  A-----ATAATA-TCT  A-----ATAATA-TCT  A-----ATAATA-TCT  A-----ATACTA-TCT  A-----ATAATA-TCT  A-----ATAATA-TCT  A-----ATAATA-TCT  A-----ATAATA-TAT  A-----ATAATA-TAT  A-----ATAATA-TCT  A-----ATAATA-TAT  A-----ATAATA-TCT  A-----ATAATA-TCT  A-----ATAATA-TCG  A-----ATAATA-TCT  A-----ATAATA-TCT  AATAATATAATA-TCT  AATAATATAATA-TCT  A-----ATAATA-TCT  AATAATATAATA-TCT  AATAATATAATA-TCT  AATAATATAATA-TCT  AATAATATAATA-TCT  AATAATATAATA-TCT  A-----ATAATA-TCT  A-----ATAATA-TCT  AATAATATAATA-GCT  A-----ATAATA-TCT  A-----ATAATA-CCC  A-----ATAATACCCT  A-----ATAAGA-CCC  A-----ATAAGA-CCC  A-----ATAATGCCCT  A-----ATAATGCCCT  A-----ATAATGCCCT  A-----ATAAAGCCCT  A-----ATAATACCCT  A-----ATAATACCCT  A-----ATAATACCTT | Ssmp | Homoplasy |
| 80 | Schedonorus arundinaceus Schedonorus arundinaceus[C] Schedonorus pratensis  Festuca altissima  Festuca ovina  Lolium multiflorum  Lolium perenne  Dactylis glomerata  Helictochloa hookeri  Deschampsia antarctica  Poa palustris  Phleum alpinum  Briza sp  Puccinellia nuttalliana  Ammophila breviligulata  Agrostis stolonifera  Hierochloe odorata  Anthoxanthum odoratum  Phalaris arundinacea  Torreyochloa  Avena sativa  Trisetum cernuum  Aegilops cylindrica  Aegilops geniculata  Aegilops speltoides  Aegilops tauschii  Triticum aestivum  Triticum monococcum aegilopoides Triticum monococcum (modified) Triticum urartu (modified)  Hordeum jubatum  Hordeum vulgare  Hordeum vulgare spontaneum Secale cereale  Bromus vulgaris  Brachypodium distachyon  Diarrhena obovata  Melica mutica  Melica subulata  Oryzopsis asperifolia Achnatherum hymenoides Ampelodesmos mauritanica Piptochaetium avenaceum  Phaenosperma globosum Brachyelytrum aristosum  Bambusa bambosa | 53761  IGS |  | TAT----------------------TTAATAGAA  TAT----------------------TTAATAGAA  TAT----------------------TTAATAGAA  TAT----------------------TTAATAGAA  TAT----------------------TTAATAGAA  TAT----------------------TTAATAGAA  TAT----------------------TTAATAGAA  TAT----------------------TTAATAGAA  TAT----------------------TTAATAGAA  TAT----------------------TTAATAGAA  TAT----------------------TTAATAGAA  TAT----------------------TTAATAGAA  TAT----------------------TTACTAGAA  TAT----------------------TTAATAGAA  TAT----------------------TTAATAGAA  TAT----------------------TTAATAGAA  TAT----------------------TTAATAGAA  TAT----------------------TTAATAGAA  TAT----------------------TTAATAGAA  TAT----------------------TTAATAGAA  TAT----------------------TTAATAGAA  TAT----------------------TTAATAGAA  TATATTATCTTATTTTGAT------TTAATAGAA  TATATTATCTTATTTTGAT------TTAATAGAA  TATATTATCTTATTTTGAT------TTAATAGAA  TATATTATCTTATTTTGAT------TTAATAGAA  TATATTATCTTATTTTGAT------TTAATAGAA  TATATTATCTTATTTTGAT------TTAATAGAA  TATATTATCTTATTTTGAT------TTAATAGAA  TATATTATCTTATTTTGAT------TTAATAGAA  TATATTATCTTATTTTTAT------TTAATAGAA  TATATTATCTTATTTTTAT------TTAATAGAA  TATATTATCTTATTTTTAT------TTAATAGAA  TATATTATCTTATTTTGAT------TTAATAGAA  TATATTATCTTATTTTTATTTTTAGTTAATAGAA  TAT----------------------TTAATAGAA  TAT----------------------TTAATAGAA  TTT----------------------TTAATAGAA  TTT----------------------TTAATAGAA  TAT----------------------TTAATAGAA  TAT----------------------TTAATAGAA  TAT----------------------TTAATAGAA  TAT----------------------TTAATAGAA  TAT----------------------TTTATAGAA  TAT----------------------TTAATAGAA  TAT----------------------TTAATAGAA |  | Bromeae+Triticeae |
| 81 | Schedonorus arundinaceus Schedonorus arundinaceus[C] Schedonorus pratensis  Festuca altissima  Festuca ovina  Lolium multiflorum  Lolium perenne  Dactylis glomerata  Helictochloa hookeri  Deschampsia antarctica  Poa palustris  Phleum alpinum  Briza sp  Puccinellia nuttalliana  Ammophila breviligulata  Agrostis stolonifera  Hierochloe odorata  Anthoxanthum odoratum  Phalaris arundinacea  Torreyochloa  Avena sativa  Trisetum cernuum  Aegilops cylindrica  Aegilops geniculata  Aegilops speltoides  Aegilops tauschii  Triticum aestivum  Triticum monococcum aegilopoides Triticum monococcum (modified) Triticum urartu (modified)  Hordeum jubatum  Hordeum vulgare  Hordeum vulgare spontaneum Secale cereale  Bromus vulgaris  Brachypodium distachyon  Diarrhena obovata  Melica mutica  Melica subulata  Oryzopsis asperifolia Achnatherum hymenoides Ampelodesmos mauritanica Piptochaetium avenaceum  Phaenosperma globosum Brachyelytrum aristosum  Bambusa bambosa | 53791  IGS |  | AGAAAT-------------TCTCTATAGCAA  AGAAAT-------------TCTCTATAGCAA  AGAAAT-------------TCTCTATAGCAA  AGAAAT-------------TCTCTATAGAAA  AGAAAT-------------TCTCTATAGCAA  AGAAAT-------------TCTCTATAGCAA  AGAAAT-------------TCTCTATAGCAA  AGAAAT-------------TCTCTATAGCAA  AGAAAT-------------TCTCTATAGCAA  AGAAAT-------------TCTCTATATCAA  AGAAAT-------------TCTCTATAGCAA  AGAAAT-------------TCTCTATAGCAA  AGAAAT-------------TCTCTATAGCAA  AGAAAT-------------TCTCTATAGCAA  AGAAAT-------------TCTCTATAGCAA  AGAAAT-------------TCTCTATAGCAA  AGAAAT-------------TCTCTATAGAAA  AGAAAT-------------TCTCTATAGCAA  AGAAAT-------------T-TCTATAGCAA  AGAAAT-------------TATCTCTAGCAA  AGAAAT-------------TTTCTATAGCAA  AGAAAT-------------TTTCTATAGCAA  AGAAAT------TAGAAATTCTCTATAGCAA  AGAAAT------TAGAAATTCTCTATAGCAA  AGAAAT------TAGAAATTCTCTATAGCAA  AGAAAT------TAGAAATTCTCTATAGCAA  AGAAAT------TAGAAATTCTCTATAGCAA  AGAAAT------TAGAAATTCTCTATAGCAA  AGAAAT------TAGAAATTCTCTATAGCAA  AGAAAT------TAGAAATTCTCTATAGCAA  AGAAAT------TTTAAATTCTCTATAGCAA  AGAAAT------TAAAAATTCTCTATAGCAA  AGAAAT------TAAAAATTCTCTATAGCAA  AGAAAT------TAGAAATTCTCTATAGCAA  AGAAATTAGAAATAGAAATTATCTACAGCAA  AGAAAT-------------TCTCTATAGCAA  AGAAAT-------------TCTCTATAGCAA  AGAAAT-------------TCTCTATAGCAA  AGAAAT-------------TCTCTATAGCAA  AGAAAT-------------TCTCTATAGCAA  AGAAAT-------------TCTCTATAGCAA  AGAAAT-------------TCTCTATAGCAA  AGAAAT-------------TCTCTATAGCAA  AGAAAT-------------TCTCTATAGCAA  AGAAAT-------------TCTCTATAGCAA  AGAAAT-------------TCTCTATAGTAA |  | Bromeae+Triticeae |
| 82 | Schedonorus arundinaceus Schedonorus arundinaceus[C] Schedonorus pratensis  Festuca altissima  Festuca ovina  Lolium multiflorum  Lolium perenne  Dactylis glomerata  Helictochloa hookeri  Deschampsia antarctica  Poa palustris  Phleum alpinum  Briza sp  Puccinellia nuttalliana  Ammophila breviligulata  Agrostis stolonifera  Hierochloe odorata  Anthoxanthum odoratum  Phalaris arundinacea  Torreyochloa  Avena sativa  Trisetum cernuum  Aegilops cylindrica  Aegilops geniculata  Aegilops speltoides  Aegilops tauschii  Triticum aestivum  Triticum monococcum aegilopoides Triticum monococcum (modified) Triticum urartu (modified)  Hordeum jubatum  Hordeum vulgare  Hordeum vulgare spontaneum Secale cereale  Bromus vulgaris  Brachypodium distachyon  Diarrhena obovata  Melica mutica  Melica subulata  Oryzopsis asperifolia Achnatherum hymenoides Ampelodesmos mauritanica Piptochaetium avenaceum  Phaenosperma globosum Brachyelytrum aristosum  Bambusa bambosa | 53836  IGS |  | TGGGATG-----TAAAATA  TGGGATG-----TAAAATA  TGGGATG-----TAAAATA  TGGGATG-----TAAAATA  TGGGATG-----TAAAATA  TGGGATG-----TAAAATA  TGGGATG-----TAAAATA  TGGGATG-----TAAAATA  TGGGATGTAAAATAAAATA  TGGTATG-----TAAAATA  TGGGATG-----TAAAATA  TGGTATG-----TAAAATA  TGGGATG-----TCAAATA  TGGGATG-----TAAAATA  TGGGATG-----TAAAATA  TGGGATG-----TAAAATA  TGGGATGTAAAATAAAATA  TGGGATG-----TAAAATA  TGGAATG-----TAAAATA  TGGGATG-----TAAAATA  TGGGATG-----TAAAATA  TCGGATG-----TAAAATA  TGGTATG-----TAAAATA  TGGTATG-----TAAAATA  TGGTATG-----TAAAATA  TGGTATG-----TAAAATA  TGGTATG-----TAAAATA  TGGTATG-----TAAAATA  TGGTATG-----TAAAATA  TGGTATG-----TAAAATA  TGGTATG-----TAAA---  TGGTATG-----TAAA---  TGGTATG-----TAAA---  TGGTATG-----TAAAATA  TGGTATG-----TAAAATA  TGGTATGT----AAAAATA  TGGTATG-----TAAAATA  TGGTATA-----TAAAATA  TGGTATA-----TAAAATA  TGGTATG-----TAAAATA  TGGTATG-----TAAAATA  TGGTATG-----TAAAATA  TGGTATG-----TAAAATA  TGGTATG-----TAAAATA  TGGTATG-----TAAAATA  TGGTATG-----TAAAATA |  | Homoplasy |
| 83 | Schedonorus arundinaceus Schedonorus arundinaceus[C] Schedonorus pratensis  Festuca altissima  Festuca ovina  Lolium multiflorum  Lolium perenne  Dactylis glomerata  Helictochloa hookeri  Deschampsia antarctica  Poa palustris  Phleum alpinum  Briza sp  Puccinellia nuttalliana  Ammophila breviligulata  Agrostis stolonifera  Hierochloe odorata  Anthoxanthum odoratum  Phalaris arundinacea  Torreyochloa  Avena sativa  Trisetum cernuum  Aegilops cylindrica  Aegilops geniculata  Aegilops speltoides  Aegilops tauschii  Triticum aestivum  Triticum monococcum aegilopoides Triticum monococcum (modified) Triticum urartu (modified)  Hordeum jubatum  Hordeum vulgare  Hordeum vulgare spontaneum Secale cereale  Bromus vulgaris  Brachypodium distachyon  Diarrhena obovata  Melica mutica  Melica subulata  Oryzopsis asperifolia Achnatherum hymenoides Ampelodesmos mauritanica Piptochaetium avenaceum  Phaenosperma globosum Brachyelytrum aristosum  Bambusa bambosa | 53848  IGS |  | TAAAATACACATAGA  TAAAATACACATAGA  TAAAATACACATAGA  TAAAATACACATAGA  TAAAATACACATAGA  TAAAATACACATAGA  TAAAATACACATGGA  TAAAATACACATGGA  TAAAATAAACATAGA  TAAAATACACATAGA  TAAAATACACATGGA  TAAAATACACATAGA  TCAAATATACATAGA  TAAAATACACATAGA  TAAAATATACATAGA  TAAAATATACATAGA  TAAAATATACATAGA  TAAAATATACATAGA  TAAAATATACATAGA  TAAAATATACATAGA  TAAAATATACATATA  TAAAATATACATATA  TAAAATACACATAGA  TAAAATACACATAGA  TAAAATACACATAGA  TAAAATACACATAGA  TAAAATACACATAGA  TAAAATACACATAGA  TAAAATACACATAGA  TAAAATACACATAGA  TAAA-----CATAGA  TAAA-----CATAGA  TAAA-----CATAGA  TAAAATACACATAGA  TAAAATACACATAGA  AAAAATACACATAGA  TAAAATACACATAGA  TAAAATACACATAGA  TAAAATACACATAGA  TAAAATACACATAGA  TAAAATACACATAGA  TAAAATACACATAGA  TAAAATACACATAGA  TAAAATACACATAGA  TAAAATAAACATAGA  TAAAATACACATAGA |  | Hordeum |
| 84 | Schedonorus arundinaceus Schedonorus arundinaceus[C] Schedonorus pratensis  Festuca altissima  Festuca ovina  Lolium multiflorum  Lolium perenne  Dactylis glomerata  Helictochloa hookeri  Deschampsia antarctica  Poa palustris  Phleum alpinum  Briza sp  Puccinellia nuttalliana  Ammophila breviligulata  Agrostis stolonifera  Hierochloe odorata  Anthoxanthum odoratum  Phalaris arundinacea  Torreyochloa  Avena sativa  Trisetum cernuum  Aegilops cylindrica  Aegilops geniculata  Aegilops speltoides  Aegilops tauschii  Triticum aestivum  Triticum monococcum aegilopoides Triticum monococcum (modified) Triticum urartu (modified)  Hordeum jubatum  Hordeum vulgare  Hordeum vulgare spontaneum Secale cereale  Bromus vulgaris  Brachypodium distachyon  Diarrhena obovata  Melica mutica  Melica subulata  Oryzopsis asperifolia Achnatherum hymenoides Ampelodesmos mauritanica Piptochaetium avenaceum  Phaenosperma globosum Brachyelytrum aristosum  Bambusa bambosa | 53883  IGS |  | AAAGAT-----------CCTAGG  AAAGAT-----------CCTAGG  AAAGAT-----------CCTAGG  AAAGAT-----------CCTAGG  AAAGAT-----------CCTAGG  AAAGAT-----------CCTAGG  AAAGAT-----------CCTAGG  AAAGAT-----------CCTAGG  AAAGAT-----------CCTAGA  AAAGAT-----------CCTAGG  AAAGAT-----------CCTAGG  AAAGAT-----------CCTAGG  AAAGAT-----------CCTAGG  AAAGAT-----------CCTAGG  AAAGAT-----------CCTAGG  AAAGAT-----------CCTAGG  AAAGAT-----------CCTAGG  AAGGAT-----------CCTAGG  AAAGAT-----------CCTAGG  AAAGAT-----------CCTAGG  AAAGAT-----------CCTAGG  AAAGAT-----------CCTAGG  AAGGATCCTAAT-----CCTAGG  AAGGATCCTAAT-----CCTAGG  AAGGATCCTAAT-----CCTAGG  AAGGATCCTAAT-----CCTAGG  AAGGATCCTAAT-----CCTAGG  AAGGATCCTAAT-----CCTAGG  AAGGATCCTAAT-----CCTAGG  AAGGATCCTAAT-----CCTAGG  AAGGATCCTAAT-----CCTAGG  AAGGATCCTAAT-----CCTAGG  AAGGATCCTAAT-----CCTAGG  AAGGATCCTAAT-----CCTAGG  AAGGATCCTAATCCTAGCCTAGG  AAGGAT-----------CCTCGG  AAGGAT-----------CCTAGG  AAGGGT-----------CCTAGG  AAGGGT-----------CCTAGG  AAGGAT-----------CCTAGG  A--GAT-----------CCTAGG  A-GGAT-----------CCTAGG  AAGGAT-----------CCTAGG  AAGGAT-----------CCTAGG  AAGGAT-----------CCTAGG  AAGGAT-----------CCTAGG |  | Bromeae+Triticeae |
| 85 | Schedonorus arundinaceus Schedonorus arundinaceus[C] Schedonorus pratensis  Festuca altissima  Festuca ovina  Lolium multiflorum  Lolium perenne  Dactylis glomerata  Helictochloa hookeri  Deschampsia antarctica  Poa palustris  Phleum alpinum  Briza sp  Puccinellia nuttalliana  Ammophila breviligulata  Agrostis stolonifera  Hierochloe odorata  Anthoxanthum odoratum  Phalaris arundinacea  Torreyochloa  Avena sativa  Trisetum cernuum  Aegilops cylindrica  Aegilops geniculata  Aegilops speltoides  Aegilops tauschii  Triticum aestivum  Triticum monococcum aegilopoides Triticum monococcum (modified) Triticum urartu (modified)  Hordeum jubatum  Hordeum vulgare  Hordeum vulgare spontaneum Secale cereale  Bromus vulgaris  Brachypodium distachyon  Diarrhena obovata  Melica mutica  Melica subulata  Oryzopsis asperifolia Achnatherum hymenoides Ampelodesmos mauritanica Piptochaetium avenaceum  Phaenosperma globosum Brachyelytrum aristosum  Bambusa bambosa | 53902  IGS |  | TAGG-CTTAATCCATTTTT  TAGGCCTTAATCCATTTTT  TAGG-CTTAATCCATTTTT  TAGG-GTTAATCCATTTTT  TAGG-GTTAATCCATTTTT  TAGG-CTTAATCCATTTTT  TAGG-CTTAATCCATTTTT  TAGG-GTTCATCCATGTTT  TAGA-GTTAATCCATTTTT  TAGG-GTTAATCTTTTTTT  TAGG-G-----------TT  TAGG-G-----------TT  TAGG-GTTAATCCTTTTTT  TAGG-GTTAATCCATTTTT  TAGG-GTTAATCCATTTTT  TAGG-GTTAATCCATTTTT  TAGG-GTTAATCCATTTTT  TAGG-GTTAATCCATTTTT  TAGG-GTGAATCCATTTTT  TAGG-GTTAATCCATTTTT  TAGG-GTTAATCCATTTTT  TAGG-GTTAATCCATTTTT  TAGG-GTTAATCAATTTTA  TAGG-GTTAATCAATTTTA  TAGG-GTTAATCAATTTTA  TAGG-GTTAATCAATTTTA  TAGG-GTTAATCAATTTTA  TAGG-GTTAATCAATTTTA  TAGG-GTTAATCAATTTTA  TAGG-GTTAATCAATTTTA  TAGG-GTTAATCAATTTTA  TAGG-GTTAATCAATTTTA  TAGG-GTTAATCAATTTTA  TAGG-GTTAATCAATTTTA  TAGG-GTTAATCAATTTTA  TCGG-GTTAATCCATTTTT  TAGG-GTTAATCCATTTTT  TAGG-GTTAATTCATTTCT  TAGG-GTTAATTCATTTCT  TAGG-GTTAATCCATTTCT  TAGG-GTTAATCCATTTCT  TAGG-GTTAATCCATTTCT  TAGG-GTTAATCCAT----  TAGG-GTTAATCCATTTCT  TAGG-GTTAATCCATTTCT  TAGG-GTTAATCCATTTCT |  | Poinae |
| 86 | Schedonorus arundinaceus Schedonorus arundinaceus[C] Schedonorus pratensis  Festuca altissima  Festuca ovina  Lolium multiflorum  Lolium perenne  Dactylis glomerata  Helictochloa hookeri  Deschampsia antarctica  Poa palustris  Phleum alpinum  Briza sp  Puccinellia nuttalliana  Ammophila breviligulata  Agrostis stolonifera  Hierochloe odorata  Anthoxanthum odoratum  Phalaris arundinacea  Torreyochloa  Avena sativa  Trisetum cernuum  Aegilops cylindrica  Aegilops geniculata  Aegilops speltoides  Aegilops tauschii  Triticum aestivum  Triticum monococcum aegilopoides Triticum monococcum (modified) Triticum urartu (modified)  Hordeum jubatum  Hordeum vulgare  Hordeum vulgare spontaneum Secale cereale  Bromus vulgaris  Brachypodium distachyon  Diarrhena obovata  Melica mutica  Melica subulata  Oryzopsis asperifolia Achnatherum hymenoides Ampelodesmos mauritanica Piptochaetium avenaceum  Phaenosperma globosum Brachyelytrum aristosum  Bambusa bambosa | 54791  IGS |  | AGGATAAATAGAGTAACATCAACTAGAGTAACATCAACTAAA  AGGATAAATAGAGTAACATCAACTAGAGTAACATCAACTAAA  AGGATAAATAGAGTAACATCAACTAGAGTAACATCAACTAAA  AGGATAAA---------------TAGAGTAAAATCAACTAAA  AGGATAAA---------------TAGAGTAAAATCAACTAAA  AGGATAAATAGAGTAACATCAACTAGAGTAACATCAACTAAA  AGGATAAATAGAGTAACATCAACTAGAGTAACATCAACTAAA  AGGATAAA---------------TAGAATAAAATCAACTAAA  AGGATAAA---------------TAGAGTAAAATCAACTAAA  AGGATACA---------------TAGAGTAAAATCAACTAAA  AGGATAAA---------------TAGAGTGAAATCAACTAAA  AGGATAAA---------------TAGAGTGAAATCAACTAAA  AGGATAAA---------------CAGAGTAAAATCAACTAAA  AGGATAAA---------------TAGAGTGAAATCAACTAAA  AGGATAAA---------------CAGAGTAAAATCAACTAAA  AGGATAAA---------------CAGAGTAAAATCAACTAAA  AGGATAAA---------------CAGAGTAAAATCAACTAAA  AGGATAAA---------------CAGAGTAAAATCAACTAAA  AGGATAAA---------------CAGAGTAAAATCAACTAAA  AGGATAAA---------------CAGAGTAAAATCAACTAAA  AGGATAAA---------------CAGAGTAAAATCAACTAAA  AGGATAAA---------------CAGAGTAAAATCAACTAAA  AGGATAAA---------------CAGAGTAAAATCAACTAAA  AGGATAAA---------------CAGAGTAAAATCAACTAAA  AGGATAAA---------------CAGAGTAAAATCAACTAAA  AGGATAAA---------------CAGAGTAAAATCAACTAAA  AGGATAAA---------------CAGAGTAAAATCAACTAAA  AGGATAAA---------------CAGAGTAAAATCAACTAAA  AGGATAAA---------------CAGAGTAAAATCAACTAAA  AGGATAAA---------------CAGAGTAAAATCAACTAAA  AGGATAAA---------------CAGAGTAAAATCAACTAAA  AGGATAAA---------------CAGAGTAAAATCAACTAAA  AGGATAAA---------------CAGAGTAAAATCAACTAAA  AGGATAAA---------------CAGAGTAAAATCAACTAAA  AGGATAAA---------------CAGAGTAAAATCAACTAAA  AGGATAAA---------------CAGAGTAAAATCAACTAAA  AGGATAAA---------------CAGAGTAAAATCAACTAAA  AGGATAAA---------------CAGAGTAAAATCAACTAAA  AGGATAAA---------------CAGAGTAAAATCAACTAAA  AGGATAAA---------------CAGAGTAAAATCAACTAAA  AGGATAAA---------------CAGAGTAAAATCAACTAAA  AGGGTAAA---------------CAGAGTAAAATCAACTAAA  AGGATAAA---------------CAGAGTAAAATCAACTAAA  AGGATAAA---------------CAGAGTAAAATCAACTAAA  AGGATAAA---------------CAGAGTAGAATCTACTAAA  AGGATAAA---------------CAGAGTAAAATCTACTAAA |  | Lolium+Schedonorus |
| 87 | Schedonorus arundinaceus Schedonorus arundinaceus[C] Schedonorus pratensis  Festuca altissima  Festuca ovina  Lolium multiflorum  Lolium perenne  Dactylis glomerata  Helictochloa hookeri  Deschampsia antarctica  Poa palustris  Phleum alpinum  Briza sp  Puccinellia nuttalliana  Ammophila breviligulata  Agrostis stolonifera  Hierochloe odorata  Anthoxanthum odoratum  Phalaris arundinacea  Torreyochloa  Avena sativa  Trisetum cernuum  Aegilops cylindrica  Aegilops geniculata  Aegilops speltoides  Aegilops tauschii  Triticum aestivum  Triticum monococcum aegilopoides Triticum monococcum (modified) Triticum urartu (modified)  Hordeum jubatum  Hordeum vulgare  Hordeum vulgare spontaneum Secale cereale  Bromus vulgaris  Brachypodium distachyon  Diarrhena obovata  Melica mutica  Melica subulata  Oryzopsis asperifolia Achnatherum hymenoides Ampelodesmos mauritanica Piptochaetium avenaceum  Phaenosperma globosum Brachyelytrum aristosum  Bambusa bambosa | 56066  IGS |  | GAATTGCAATAAAATAAGCAA  GAATTGCAATAAAATAAGCAA  GAATTGC-----AATAAGCAA  GAATTGC-----AATAAGCAA  GAATTGC-----AATAAGCAA  GAATTGC-----AATAAGCAA  GAATTGC-----AATAAGCAA  GAATTGC-----AATAAGCAA  GAATTGC-----AATAAGCAA  GAATTGC-----AATAAGCAA  GAATTGC-----AATAAACAA  GAATTGC-----AATAAGCAA  GAATTGC-----AATAAGCAA  GAATTGC-----AATAAGCAA  GAATTGC-----AATAAGCAA  GAATTGC-----AATAAGCAA  GAATTGC-----AATAAGCAA  AAATTGC-----AATAAGCAA  TAATTGC-----AATAAGCAA  GAATTGC-----AATAAGCAA  GAATTGC-----AATAAGCAA  GAATTGC-----AATAAGCAA  GAATTGC-----AATAAGCAA  GAATTGC-----AATAAGCAA  GAATTGC-----AATAAGCAA  GAATTGC-----AATAAGCAA  GAATTGC-----AATAAGCAA  GAATTGC-----AATAAGCAA  GAATTGC-----AATAAGCAA  GAATTGC-----AATAAGCAA  GAATTGC-----AATAAGCAA  GAATTGC-----AATAAGCAA  GAATTGC-----AATAAGCAA  GAATTGC-----AATAAGCAA  GAATTGC-----AATAAGCAA  GAATTGC-----AATAAACAA  GAATTGC-----AATAAGCAA  GAATTGC-----AATAAGCAA  GAATTGC-----AATAAGCAA  GAATTGC-----AATAAGCAA  GAATTGC-----AATAAGCAA  GAATTGC-----AATAAGCAA  GAATTGC-----AATAAGCAA  GAATTGC-----AATAAGCAA  GAATTGC-----AATAAGCAA  GAATTGC-----AACAAGCA- | ssmp | Schedonorus arundinaceus |
| 88 | Schedonorus arundinaceus Schedonorus arundinaceus[C] Schedonorus pratensis  Festuca altissima  Festuca ovina  Lolium multiflorum  Lolium perenne  Dactylis glomerata  Helictochloa hookeri  Deschampsia antarctica  Poa palustris  Phleum alpinum  Briza sp  Puccinellia nuttalliana  Ammophila breviligulata  Agrostis stolonifera  Hierochloe odorata  Anthoxanthum odoratum  Phalaris arundinacea  Torreyochloa  Avena sativa  Trisetum cernuum  Aegilops cylindrica  Aegilops geniculata  Aegilops speltoides  Aegilops tauschii  Triticum aestivum  Triticum monococcum aegilopoides Triticum monococcum (modified) Triticum urartu (modified)  Hordeum jubatum  Hordeum vulgare  Hordeum vulgare spontaneum Secale cereale  Bromus vulgaris  Brachypodium distachyon  Diarrhena obovata  Melica mutica  Melica subulata  Oryzopsis asperifolia Achnatherum hymenoides Ampelodesmos mauritanica Piptochaetium avenaceum  Phaenosperma globosum Brachyelytrum aristosum  Bambusa bambosa | 56105  IGS |  | ATAGAGTC-----GAACTGCT  ATAGAGTC-----GAACTGCT  ATAGAGTC-----GAACTGCT  ATAGAGTC-----GAACTGCT  ATAGAGTC-----GAACTGCT  ATAGAGTC-----GAACTGCT  ATAGAGTC-----GAACTGCT  ATAGAGTC-----GAACTGCT  ATAGAGTC-----AAACTCCT  ATAGAGTC-----GAACTGCT  ATAGAGTC-----GAACTGCT  ATAGAGTC-----GAACTGCT  ATAGAGTC-----GAACTGCT  ATAGAGTT-----GAACTGCT  ATAGAGTC-----GAACTGCT  ATAGAGTC-----GAACTGCT  ATAGAGTC-----GAACTGCT  ATAGAGTC-----GAATTGCT  ATAGAGTC-----GAACTGCT  ATAGAGTC-----GAACTGCT  ATAGAGTC-----GAACTGCT  ATAGAGTC-----GAACTGCT  ATAGAGTCGAACTGAACTGCT  ATAGAGTCGAACTGAACTGCT  ATAGAGTCGAACTGAACTGCT  ATAGAGTCGAACTGAACTGCT  ATAGAGTCGAACTGAACTGCT  ATAGAGTCGAACTGAACTGCT  ATAGAGTCGAACTGAACTGCT  ATAGAGTCGAACTGAACTGCT  ATAGAGTCGAACTGAACTGCT  ATAGAGTCGAACTGAACTGCT  ATAGAGTCGAACTGAACTGCT  ATAGAGTCGAACTGAACTGCT  ATAGAGTAGAACTGAACTGCT  ATAGAATC-----GAACTGCT  ATAGAGTC-----GAACTGCT  ATAGAGTC-----GAACTGGT  ATAGAGTC-----GAACTGGT  ATAGAGTC-----GAACTGCT  ATAGAGTC-----GAACTGCT  ATAGAGTC-----GAACTGCT  ATAGAGTC-----GAACTGCT  ATAGAGTC-----GAACTGCT  ATAGAGTC-----GAACTGCT  ATAGAGAC-----GAACTGCT | ssmp | Bromeae+Triticeae |
| 89 | Schedonorus arundinaceus Schedonorus arundinaceus[C] Schedonorus pratensis  Festuca altissima  Festuca ovina  Lolium multiflorum  Lolium perenne  Dactylis glomerata  Helictochloa hookeri  Deschampsia antarctica  Poa palustris  Phleum alpinum  Briza sp  Puccinellia nuttalliana  Ammophila breviligulata  Agrostis stolonifera  Hierochloe odorata  Anthoxanthum odoratum  Phalaris arundinacea  Torreyochloa  Avena sativa  Trisetum cernuum  Aegilops cylindrica  Aegilops geniculata  Aegilops speltoides  Aegilops tauschii  Triticum aestivum  Triticum monococcum aegilopoides Triticum monococcum (modified) Triticum urartu (modified)  Hordeum jubatum  Hordeum vulgare  Hordeum vulgare spontaneum Secale cereale  Bromus vulgaris  Brachypodium distachyon  Diarrhena obovata  Melica mutica  Melica subulata  Oryzopsis asperifolia Achnatherum hymenoides Ampelodesmos mauritanica Piptochaetium avenaceum  Phaenosperma globosum Brachyelytrum aristosum  Bambusa bambosa | 56405  trnL-UAA intron 1 | trnL-UAA intron 1 | GGTTC-----TCGAA  GGTTC-----TCGAA  GGTTC-----TCGAA  GGTTC-----TCGAA  GGTTC-----TCGAA  GGTTC-----TCGAA  GGTTC-----TCGAA  GGTTC-----TCGAA  GGTTC-----TCGAA  GGTTC-----TCGAA  GGTTC-----TCGAA  GGTTC-----TCGAA  GGTTC-----TCAAA  GGTTC-----TCGAA  GGTTCTCGAATCGAA  GGTTCTCGAATCGAA  GGTTC-----TCGAA  GGTTC-----TCGAA  GGTTC-----TCGAA  GGTTC-----TCGAA  GGTTC-----TCGAA  GGTTC-----TCGAA  GGTTC-----TCGAA  GGTTC-----TCGAA  GGTTC-----TCGAA  GGTTC-----TCGAA  GGTTC-----TCGAA  GGTTC-----TCGAA  GGTTC-----TCGAA  GGTTC-----TCGAA  GATTC-----TCGAA  GATTC-----TCGAA  GATTC-----TCGAA  GGTTC-----TCGAA  GGTTC-----TCGAA  GGTTC-----TCGAA  GGTTC-----TCGAA  GGTTC-----TCGAA  GGTTC-----TCGAA  AGTTC-----TCGAA  AGTTC-----TCGAA  GGTTC-----TCGAA  GGTTC-----TCGAA  GGTTC-----TCGAA  GATTC-----TCGAA  GGTTC-----TCGAA | ssmp | Agrostidinae |
| 90 | Schedonorus arundinaceus Schedonorus arundinaceus[C] Schedonorus pratensis  Festuca altissima  Festuca ovina  Lolium multiflorum  Lolium perenne  Dactylis glomerata  Helictochloa hookeri  Deschampsia antarctica  Poa palustris  Phleum alpinum  Briza sp  Puccinellia nuttalliana  Ammophila breviligulata  Agrostis stolonifera  Hierochloe odorata  Anthoxanthum odoratum  Phalaris arundinacea  Torreyochloa  Avena sativa  Trisetum cernuum  Aegilops cylindrica  Aegilops geniculata  Aegilops speltoides  Aegilops tauschii  Triticum aestivum  Triticum monococcum aegilopoides Triticum monococcum (modified) Triticum urartu (modified)  Hordeum jubatum  Hordeum vulgare  Hordeum vulgare spontaneum Secale cereale  Bromus vulgaris  Brachypodium distachyon  Diarrhena obovata  Melica mutica  Melica subulata  Oryzopsis asperifolia Achnatherum hymenoides Ampelodesmos mauritanica Piptochaetium avenaceum  Phaenosperma globosum Brachyelytrum aristosum  Bambusa bambosa | 56415  trnL-UAA intron 1 | trnL-UAA intron 1 | TCGAACTAGAATACAAAGGAAAAGGAT  TCGAACTAGAATACAAAGGAAAAGGAT  TCGAACTAGAATACAAAGGAAAAGGAT  TCGAACTAGAATACAAAGGAAAAGGAT  TCGAACTAGAATACAAAGGAAAAGGAT  TCGAACTAGAATACAAAGGAAAAGGAT  TCGAACTAGAATACAAAGGAAAAGGAT  TCGAACTAGAATACAAAGGAAAAGGAT  TCGAACTAGAATACAAAGGAAAAGGAT  TCGAACTAGAATGCAAAGGAAAAGGAT  TCGAACTAGAATACAAAGGAAAAGGAT  TCGAACTAGAATACAAAGGAAAAGGAT  TCAAACTAGAATACAAAGGAAAAGGAT  TCGAACTAGAATACAAAGGAAAAGGAT  TCGAACTAGAATACAAAGGAAAAGGAT  TCGAACTATAATACAAAGGAAAAGGAT  TCGAACTAGAATACAAAGGAAAAGGAT  TCGAACTAGAATACAAAGGAAAAGGAT  TCGAACTAGAATACAAAGGAAAAGGAT  TCGAACTAGAATACAAAAGAAAAGGAT  TCGAACTAGAATACAAAGGAAAAGGAT  TCGAACTAGAATACAAAGGAAAAGGAT  TCGAACTAGAATACAAAGGAAAAGGAT  TCGAACTAGAATACAAAGGAAAAGGAT  TCGAACTAGAATACAAAGGAAAAGGAT  TCGAACTAGAATACAAAGGAAAAGGAT  TCGAACTAGAATACAAAGGAAAAGGAT  TCGAACTAGAATACAAAGGAAAAGGAT  TCGAACTAGAATACAAAGGAAAAGGAT  TCGAACTAGAATACAAAGGAAAAGGAT  TCGAACTAGAATACAAAGGAAAAGGAT  TCGAACTAGAATACAAAGGAAAAGGAT  TCGAACTAGAATACAAAGGAAAAGGAT  TCGAACTAGAATACAAAGGAAAAGGAT  TCGAACTAGAATACAAAGGAAAAGGAT  TCGAACTAGAATCCAAAGGAAAAGGAT  TCGAACTAGAATCCAAAGGAAAAGGAT  TCGAACTAGAATCCAAAGGAAAAGGAT  TCGAACTAGAATCCAAAGGAAAAGGAT  TCGAACTAG-------------AGGAT  TCGAACTAG-------------AGGAT  TCGAACTAG-------------AGGAT  TCGAACTAG-------------AGGAT  TCGAACTAGAATCCAAAGGAAAAGGAT  TCGAACTAGAATCCAAAGGAAAAGGAT  TCGAACTAGAATCCAAAGGAAAAGGAT |  | Ampelodesmeae+Stipeae |
| 91 | Schedonorus arundinaceus Schedonorus arundinaceus[C] Schedonorus pratensis  Festuca altissima  Festuca ovina  Lolium multiflorum  Lolium perenne  Dactylis glomerata  Helictochloa hookeri  Deschampsia antarctica  Poa palustris  Phleum alpinum  Briza sp  Puccinellia nuttalliana  Ammophila breviligulata  Agrostis stolonifera  Hierochloe odorata  Anthoxanthum odoratum  Phalaris arundinacea  Torreyochloa  Avena sativa  Trisetum cernuum  Aegilops cylindrica  Aegilops geniculata  Aegilops speltoides  Aegilops tauschii  Triticum aestivum  Triticum monococcum aegilopoides Triticum monococcum (modified) Triticum urartu (modified)  Hordeum jubatum  Hordeum vulgare  Hordeum vulgare spontaneum Secale cereale  Bromus vulgaris  Brachypodium distachyon  Diarrhena obovata  Melica mutica  Melica subulata  Oryzopsis asperifolia Achnatherum hymenoides Ampelodesmos mauritanica Piptochaetium avenaceum  Phaenosperma globosum Brachyelytrum aristosum  Bambusa bambosa | 56517  trnL-UAA intron 1 | trnL-UAA intron 1 | AATTC-C------TTCTAA  AATTC-C------TTCTAA  AATTC-C------TTCTAA  AATTC-C------TTCTAA  AATTC-C------TTCTAA  AATTC-C------TTCTAA  AATTC-C------TTCTAA  AAGTT-C------TTCTAA  AATTC-C------TTCTAA  AATTC-C------TTC---  AATTC-C------TTCGAA  GATTC-C------TTCTAA  AACTC-C------TTCTAA  AATTC-C------TTCTAA  AACTC-C------TTCTAA  AACTA-C------TTCTAA  AACTC-C------TTCTAA  AACTCTT------TTCTAA  AACTC-C------TTCTAA  AACTC-C------TTCTAA  AACTC-C------TTCTAA  AACTC-C------TTCTAA  AATTC-CTTCTAATTCTAA  AATTC-CTTCTAATTCTAA  AATTC-CTTCTAATTCTAA  AATTC-CTTCTAATTCTAA  AATTC-CTTCTAATTCTAA  AATTC-CTTCTAGTTCTAA  AATTC-CTTCTAGTTCTAA  AATTC-CTTCTAGTTCTAA  AATTC-CTTCTAATTCTAA  AATTC-CTTCTAATTCTAA  AATTC-CTTCTAATTCTAA  AATTC-CTTCTAATTCTAA  AATTC-CTTCTAATTCTAA  AAATC-C------TTTTAA  AATTC-C------TTCTAA  AATTC-C------TTATAA  AATTC-C------TTATAA  AATTC-C------TTCTAA  AATTC-C------TTCTAA  AATTC-C------TTCTAA  AATTC-C------TTCTAA  AATTC-C------TTTTAA  AATTC-C------TTCTAA  AACTC-C------CTCTAA | ssmp | Triticeae |
| 92 | Schedonorus arundinaceus Schedonorus arundinaceus[C] Schedonorus pratensis  Festuca altissima  Festuca ovina  Lolium multiflorum  Lolium perenne  Dactylis glomerata  Helictochloa hookeri  Deschampsia antarctica  Poa palustris  Phleum alpinum  Briza sp  Puccinellia nuttalliana  Ammophila breviligulata  Agrostis stolonifera  Hierochloe odorata  Anthoxanthum odoratum  Phalaris arundinacea  Torreyochloa  Avena sativa  Trisetum cernuum  Aegilops cylindrica  Aegilops geniculata  Aegilops speltoides  Aegilops tauschii  Triticum aestivum  Triticum monococcum aegilopoides Triticum monococcum (modified) Triticum urartu (modified)  Hordeum jubatum  Hordeum vulgare  Hordeum vulgare spontaneum Secale cereale  Bromus vulgaris  Brachypodium distachyon  Diarrhena obovata  Melica mutica  Melica subulata  Oryzopsis asperifolia Achnatherum hymenoides Ampelodesmos mauritanica Piptochaetium avenaceum  Phaenosperma globosum Brachyelytrum aristosum  Bambusa bambosa | 56566  trnL-UAA intron 1 | trnL-UAA intron 1 | GGCTT-------------TATACAT  GGCTT-------------TATACAT  GGCTT-------------TATACAT  GGCTT-------------TATACAT  G-----------------TATACAC  GGCTT-------------TATACAT  GGCTT-------------TATACAT  GGCTT-------------TATACAT  GGCTT-------------TATACAT  GGCTT-------------TATACAT  GGCTT-------------TATACAG  GGCTT-------------TATACAT  GGCTT-------------TATACAT  GGCTT-------------TATACAT  GGCTT-------------TATACAT  GGCTT-------------TATACAT  GGCTT-------------TATACAT  GGCTT-------------TATACAT  GGCTT-------------TATACAT  GGCTT-------------TATACAT  GGCTT-------------TATACAT  GGCTT-------------TATACAT  GGTTTTATACCT------TATACAT  GGTTTTATACCT------TATACAT  GGTTTTATACCT------TATACAT  GGTTTTATACCT------TATACAT  GGTTTTATACCT------TATACAT  GGTTTTATACCT------TATACAT  GGTTTTATACCT------TATACAT  GGTTTTATACCT------TATACAT  GGTTTTATACTT------TATACAT  GGTTTTATACTT------TATACAT  GGTTTTATACTT------TATACAT  GGTTTTATACCTTATACATATACAT  GGTTTCATACTT------TATACAT  GGATT-------------TTTACAT  GGCTT-------------TATACAT  GGCTT-------------TATACAT  GGCTT-------------TATACAT  GGCTT-------------TATACAT  GGCTT-------------TATACAT  GGCTT-------------TATACAT  GGCTT-------------TATACAT  GGCTT-------------TATACAT  GGCTG-------------TATACAT  GGGTT-------------TATACAT |  | Bromeae+Triticeae |
| 93 | Schedonorus arundinaceus Schedonorus arundinaceus[C] Schedonorus pratensis  Festuca altissima  Festuca ovina  Lolium multiflorum  Lolium perenne  Dactylis glomerata  Helictochloa hookeri  Deschampsia antarctica  Poa palustris  Phleum alpinum  Briza sp  Puccinellia nuttalliana  Ammophila breviligulata  Agrostis stolonifera  Hierochloe odorata  Anthoxanthum odoratum  Phalaris arundinacea  Torreyochloa  Avena sativa  Trisetum cernuum  Aegilops cylindrica  Aegilops geniculata  Aegilops speltoides  Aegilops tauschii  Triticum aestivum  Triticum monococcum aegilopoides Triticum monococcum (modified) Triticum urartu (modified)  Hordeum jubatum  Hordeum vulgare  Hordeum vulgare spontaneum Secale cereale  Bromus vulgaris  Brachypodium distachyon  Diarrhena obovata  Melica mutica  Melica subulata  Oryzopsis asperifolia Achnatherum hymenoides Ampelodesmos mauritanica Piptochaetium avenaceum  Phaenosperma globosum Brachyelytrum aristosum  Bambusa bambosa | 60216  IGS |  | AGCGCTT------AGTTTA  AGCGCTT------AGTTTA  AGCGCTT------AGTTTA  AGCGCTT------AGTTTA  AGCGCTT------AGTTTA  AGCGCTT------AGTTTA  AGCGCTT------AGTTTA  AGCGCTT------AGTTTA  AGCGCTT------AGTTTA  AGCGCTT------AGTTTA  AGCGCTT------AGTTTA  AGCGCTT------AGTTTA  AGTGCTT------AGTTTA  AGCGCTT------AGTTTA  AGTGCTT------AGTTTA  AGTTCTT------ATTTTA  AGCGCTT------AGTTTA  AGCGCTT------AGTTTA  AGCGCTT------AGTTTA  AGCGCTT------AGTTTA  AGCGCTT------TGTTTA  AGCGCTT------TGTTTA  AGCGCTT------AGTTTA  AGCGCTT------AGTTTA  AGCGCTT------AGTTTA  AGCGCTT------AGTTTA  AGCGCTT------AGTTTA  AGCGCTTAGTTTAAGTTTA  AGCGCTTAGTTTAAGTTTA  AGCGCTTAGTTTAAGTTTA  AGCGCTT------AGTTTA  AGCGCTT------AGTTTA  AGCGCTT------AGTTTA  AGCGCTT------AGTTTA  AGCGCTT------AGTTTA  ATCGCTT------AGTTTA  AGCGCTT------AGTTTA  AGCGCTT------AGTTTA  AGCGCTT------AGTTTA  AGCGCTT------AGTTTA  AGCGCTT------AGTTTA  AGCGCTT------AGTTTA  AGCGCTT------AGTTTA  AGCGCTT------AGTTTA  AGCGCTT------AGTTTA  AGCGCTT------AGTTTA | Ssmp | Triticum minus T. aestivum |
| 94 | Schedonorus arundinaceus Schedonorus arundinaceus[C] Schedonorus pratensis  Festuca altissima  Festuca ovina  Lolium multiflorum  Lolium perenne  Dactylis glomerata  Helictochloa hookeri  Deschampsia antarctica  Poa palustris  Phleum alpinum  Briza sp  Puccinellia nuttalliana  Ammophila breviligulata  Agrostis stolonifera  Hierochloe odorata  Anthoxanthum odoratum  Phalaris arundinacea  Torreyochloa  Avena sativa  Trisetum cernuum  Aegilops cylindrica  Aegilops geniculata  Aegilops speltoides  Aegilops tauschii  Triticum aestivum  Triticum monococcum aegilopoides Triticum monococcum (modified) Triticum urartu (modified)  Hordeum jubatum  Hordeum vulgare  Hordeum vulgare spontaneum Secale cereale  Bromus vulgaris  Brachypodium distachyon  Diarrhena obovata  Melica mutica  Melica subulata  Oryzopsis asperifolia Achnatherum hymenoides Ampelodesmos mauritanica Piptochaetium avenaceum  Phaenosperma globosum Brachyelytrum aristosum  Bambusa bambosa | 60268  IGS |  | AAAGA-----TGCATTGCATCC  AAAGA-----TGCATTGCATCC  AAAGA-----TGCATTGCATCC  AAAGA-----TGCATTGCATCC  AAAGATGCATTGCATTGCATCC  AAAGA-----TGCATTGCATCC  AAAGA-----TGCATTGCATCC  AAAGA-----TGCATTGCATCC  AAAGA-----TGCATTGCATCC  GAAGA-----TGCATTGCATCA  AAAGA-----TGCATTGCATCC  AAAGA-----TGCATTGCATCC  AAAGA----------TTCATCC  AAAGA-----TGTATTGCATCC  AAAGA----------TTCATCC  AAAGA----------TTCATCC  AAAGA----------TTCATCC  AACGA----------TTCATCC  AAAGA----------TTCATCC  AAAGA----------TTCATCC  AAAGA----------TTCATCC  AAAGA----------TTCATCC  AAAGA----------TTCATCC  AAAGA----------TTCATCC  AAAGA----------TTCATCC  AAAGA----------TTCATCC  AAAGA----------TTCATCC  AAAGA----------TTCATCC  AAAGA----------TTCATCC  AAAGA----------TTCATCC  AAAGA----------TTCATCC  AAAGA----------TTCATCC  AAAGA----------TTCATCC  AAAGA----------TTAATCC  AAAGA----------TTCATCC  AAAGA----------TTCATCC  AAAGA----------TTCATCC  AAAGA----------TTCATCC  AAAGA----------TTCATCC  AACGA----------TTCATCC  AACGA----------TTCATCA  AACGA----------TTCATCC  AACGA----------TTCATCC  AAAGA----------TTCATCC  AAAGA----------TTCATCC  AAAGA----------TTCATCC |  | Poeae clade 2 |
| 95 | Schedonorus arundinaceus Schedonorus arundinaceus[C] Schedonorus pratensis  Festuca altissima  Festuca ovina  Lolium multiflorum  Lolium perenne  Dactylis glomerata  Helictochloa hookeri  Deschampsia antarctica  Poa palustris  Phleum alpinum  Briza sp  Puccinellia nuttalliana  Ammophila breviligulata  Agrostis stolonifera  Hierochloe odorata  Anthoxanthum odoratum  Phalaris arundinacea  Torreyochloa  Avena sativa  Trisetum cernuum  Aegilops cylindrica  Aegilops geniculata  Aegilops speltoides  Aegilops tauschii  Triticum aestivum  Triticum monococcum aegilopoides Triticum monococcum (modified) Triticum urartu (modified)  Hordeum jubatum  Hordeum vulgare  Hordeum vulgare spontaneum Secale cereale  Bromus vulgaris  Brachypodium distachyon  Diarrhena obovata  Melica mutica  Melica subulata  Oryzopsis asperifolia Achnatherum hymenoides Ampelodesmos mauritanica Piptochaetium avenaceum  Phaenosperma globosum Brachyelytrum aristosum  Bambusa bambosa | 60343  IGS |  | TGGT----GTAGAT  TGGT----GTAGAT  TGGT----GTAGAT  TGGT----GTAGAT  TGGT----GTAGAT  TGGT----GTAGGT  TGGT----GTAGAT  TGGT----ATAGAT  TAGTGTACGTAGAT  TGGT----GTAGAT  TGGT----GTAGAT  TGGT----GTAGAT  TGGT----GTAGAT  TGGT----ATAGAT  TGGT----GTAGAT  TGGT----GTAGAT  TGGT----GTAGAT  TGGT----ATAGAT  TGGT----GTAGAT  TGGT----GTAGAT  TGGT----GTAGAT  TGGT----GTAGAT  TGGTGTATGTAGAT  TGGTGTATGTAGAT  TGGTGTATGTAGAT  TGGTGTATGTAGAT  TGGTGTATGTAGAT  TGGTGTATGTAGAT  TGGTGTATGTAGAT  TGGTGTATGTAGAT  TGGTGTATGTAGAT  TGGTGTATGTAGAT  TGGTGTATGTAGAT  TGGTGTATGTAGAT  TGGTGTATGTAGAT  TGGT----ATAGAT  TGGT----GTAGAT  TGGT----GTAGAT  TGGT----GTAGAT  TGGT----GTAGAT  TGGT----GTAGAT  TGGT----GTAGAT  TGGT----GTAGAT  TGGT----GTAGAT  TGGT----GTAGAC  TGGT----GTAGAC |  | Homoplasy |
| 96 | Schedonorus arundinaceus Schedonorus arundinaceus[C] Schedonorus pratensis  Festuca altissima  Festuca ovina  Lolium multiflorum  Lolium perenne  Dactylis glomerata  Helictochloa hookeri  Deschampsia antarctica  Poa palustris  Phleum alpinum  Briza sp  Puccinellia nuttalliana  Ammophila breviligulata  Agrostis stolonifera  Hierochloe odorata  Anthoxanthum odoratum  Phalaris arundinacea  Torreyochloa  Avena sativa  Trisetum cernuum  Aegilops cylindrica  Aegilops geniculata  Aegilops speltoides  Aegilops tauschii  Triticum aestivum  Triticum monococcum aegilopoides Triticum monococcum (modified) Triticum urartu (modified)  Hordeum jubatum  Hordeum vulgare  Hordeum vulgare spontaneum Secale cereale  Bromus vulgaris  Brachypodium distachyon  Diarrhena obovata  Melica mutica  Melica subulata  Oryzopsis asperifolia Achnatherum hymenoides Ampelodesmos mauritanica Piptochaetium avenaceum  Phaenosperma globosum Brachyelytrum aristosum  Bambusa bambosa | 60655  IGS |  | GCAGTA----TAATGAGA  GCAGTA----TAATGAGA  GCAGTA----TAATGAGA  GCAGTA----TAATGAGA  GCAGTA----TAATGAGA  GCAGTA----TAATGAGA  GCAGTA----TAATGAGA  GCAGTA----TAATGAGA  GCAGTA----TAATGAGA  GCAGTA----TAATGAGA  GCAGTA----TAATGAGA  GCAGTA----TAATGAGA  GCAGTA----TAATGAGA  GCAGTA----TAATGAGA  GCAGTA----TAATGAGA  GCAGTA----TAATGAGA  GCAGTA----TAATGAGA  GCAGTA----TAATGAGA  GCAGTA----CAATGAGA  GCAGTA----TAATGAGA  GCAGTA----TAATGAGA  GCAGTA----TAATGAGA  GCAGTATAATTAATGAGA  GCAGTATAATTAATGAGA  GCAGTATAATTAATGAGA  GCAGTATAATTAATGAGA  GCAGTAGAATTAATGAGA  GCAGTATAATTAATGAGA  GCAGTATAATTAATGAGA  GCAGTATAATTAATGAGA  GCAGTA----TAATGAGA  GCAGTA----TAATGAGA  GCAGTA----TAATGAGA  GCAGTATAATTAATGAGA  GCAGTA----TAATGAGA  GCAGTA----GACTGAGA  GCAGTA----TAATGAGA  GCAGTC----TAATGAGA  GCAGTC----TAATGAGA  GCAGTC----TAATGAGA  GCAGTC----TAAGGAGA  GCAGTC----TAATGAGA  GCAGTC----TAATGAGA  GCAGTC----TAATGAGA  GTAGCC----TAATGAGA  GCAGCC----TAATGAGG | Ssmp | Aegilops+Triticum |
| 97 | Schedonorus arundinaceus Schedonorus arundinaceus[C] Schedonorus pratensis  Festuca altissima  Festuca ovina  Lolium multiflorum  Lolium perenne  Dactylis glomerata  Helictochloa hookeri  Deschampsia antarctica  Poa palustris  Phleum alpinum  Briza sp  Puccinellia nuttalliana  Ammophila breviligulata  Agrostis stolonifera  Hierochloe odorata  Anthoxanthum odoratum  Phalaris arundinacea  Torreyochloa  Avena sativa  Trisetum cernuum  Aegilops cylindrica  Aegilops geniculata  Aegilops speltoides  Aegilops tauschii  Triticum aestivum  Triticum monococcum aegilopoides Triticum monococcum (modified) Triticum urartu (modified)  Hordeum jubatum  Hordeum vulgare  Hordeum vulgare spontaneum Secale cereale  Bromus vulgaris  Brachypodium distachyon  Diarrhena obovata  Melica mutica  Melica subulata  Oryzopsis asperifolia Achnatherum hymenoides Ampelodesmos mauritanica Piptochaetium avenaceum  Phaenosperma globosum Brachyelytrum aristosum  Bambusa bambosa | 61191  IGS |  | CTATGATTTTGATTTTCGATGA  CTATGATTTTGATTTTCGATGA  CTATGATTTTGATTTTCGATGA  CTATGATTTTGATTTTCGATGA  CTATTATTTTGATTTTCGATGA  CTATGATTTTGATTTTCGATGA  CTATGATTTTGATTTTCGATGA  CTATGATTTTGATTCTCGATGA  CTATGATTTTGATTTTCGATGA  CTATGATTTTGATTTTCGATGA  CTATGATTTTGATTTTCGATCA  CTATGATTTTTATTTTCGATGA  CTATGCTTTTGATTTTCGATGA  CTGTGATTTTGATTTTCGATGA  CTATGATTTTGATTTTCGATGA  CTATGATTTTGATTTTCGATGA  CTATGATTTTGATTTTCGATGA  CTATGATTTTGATTTTCGATGA  CTATGATTTTGATTTTCGATCA  CTATGATTTTGATTTTCAATGA  CTATGATTTTGATGTTCGATGA  CTATGATTTTGATGTTCGATGA  GTATGATTTTGATTTTCGATGA  GTATGATTTTGATTTTCGATGA  GTATGATTTTGATTTTCGATGA  GTATGATTTTGATTTTCGATGA  GTATGATTTTGATTTTCGATGA  GTATGATTTTGATTTTCGATGA  GTATGATTTTGATTTTCGATGA  GTATGATTTTGATTTTCGATGA  GTATGATTTTGATTTTCGATGA  GTATGATTTTGATTTTCGATGA  GTATGATTTTGATTTTCGATGA  GTATGATTTTGATTTTCGATGA  GTATGATTTTGATTTTCGATGA  CTATGATT------TTTGATGA  CTATGATT------TTCGATGA  CTATGATT------TTCGATAA  CTATGATT------TTCGATAA  CTATGATT------TTCGATGA  CTATGATT------TTCGATGA  CTATGATT------TTCGATGA  CTATGATT------TTCGATGA  CTATGATT------TTCGATGA  CTATGATT------TTCGATGA  CTATGTTT------TTCGAAGA |  | Bromeae+Poeae+Triticeae |
| 98 | Schedonorus arundinaceus Schedonorus arundinaceus[C] Schedonorus pratensis  Festuca altissima  Festuca ovina  Lolium multiflorum  Lolium perenne  Dactylis glomerata  Helictochloa hookeri  Deschampsia antarctica  Poa palustris  Phleum alpinum  Briza sp  Puccinellia nuttalliana  Ammophila breviligulata  Agrostis stolonifera  Hierochloe odorata  Anthoxanthum odoratum  Phalaris arundinacea  Torreyochloa  Avena sativa  Trisetum cernuum  Aegilops cylindrica  Aegilops geniculata  Aegilops speltoides  Aegilops tauschii  Triticum aestivum  Triticum monococcum aegilopoides Triticum monococcum (modified) Triticum urartu (modified)  Hordeum jubatum  Hordeum vulgare  Hordeum vulgare spontaneum Secale cereale  Bromus vulgaris  Brachypodium distachyon  Diarrhena obovata  Melica mutica  Melica subulata  Oryzopsis asperifolia Achnatherum hymenoides Ampelodesmos mauritanica Piptochaetium avenaceum  Phaenosperma globosum Brachyelytrum aristosum  Bambusa bambosa | 61565  trnV-UAC | trnV-UAC | CTTTACGGAAACATAAAGAT  CTTTACGGAAACATAAAGAT  CTTTACGGAAACATAAAGAT  CTTTACGGAAACATAAAGAT  CTTTACGGAAACATAAAGAT  CTTTACGGAAACATAAAGAT  CTTTACGGAAACATAAAGAT  CTTTACGGAAACATAAAGAT  CTTTACGGAAACATAAAGAT  CTTTACGGAAAGATAAAGAT  CTTTACGGAAAAATAAAGAT  CTTTACGGAAACATAAAGAT  CTTTACGG------AAAGAT  CTTTATGGAAACATAAAGAT  CTTTACGG------AAAGAT  CTTTACGG------AAAGAT  CTTTACGG------AAAGAT  CTTTACGG------AAAGAT  CTTTACGG------AAAGAT  CTTTACGG------AAAGAT  CTTTACGG------AAAGAT  CTTTACGG------AAAGAT  CTTTACGG------AAAGAT  CTTTACGG------AAAGAT  CTTTACGG------AAAGAT  CTTTACGG------AAAGAT  CTTTACGG------AAAGAT  CTTTACGG------AAAGAT  CTTTACGG------AAAGAT  CTTTACGG------AAAGAT  CTTTACGG------AAAGAT  CTTTACGG------AAAGAT  CTTTACGG------AAAGAT  CTTTACGG------AAAGAT  CTTTACGA------AAAGAT  CTTTACGG------AAAGAT  CTTTACGG------AAAGAT  CTTTACGG------AAAGAT  CTTTACGG------AAAGAT  CTTTACGG------AAAGAT  CTTTACGG------AAAGAT  CTTTACGG------AAAGAT  CTTTACGG------AAAGAT  CTTTACGG------AAAGAT  CTTTACGG------AAAGAT  CTTTACGG------AAAGAT |  | Poeae clade 2 |
| 99 | Schedonorus arundinaceus Schedonorus arundinaceus[C] Schedonorus pratensis  Festuca altissima  Festuca ovina  Lolium multiflorum  Lolium perenne  Dactylis glomerata  Helictochloa hookeri  Deschampsia antarctica  Poa palustris  Phleum alpinum  Briza sp  Puccinellia nuttalliana  Ammophila breviligulata  Agrostis stolonifera  Hierochloe odorata  Anthoxanthum odoratum  Phalaris arundinacea  Torreyochloa  Avena sativa  Trisetum cernuum  Aegilops cylindrica  Aegilops geniculata  Aegilops speltoides  Aegilops tauschii  Triticum aestivum  Triticum monococcum aegilopoides Triticum monococcum (modified) Triticum urartu (modified)  Hordeum jubatum  Hordeum vulgare  Hordeum vulgare spontaneum Secale cereale  Bromus vulgaris  Brachypodium distachyon  Diarrhena obovata  Melica mutica  Melica subulata  Oryzopsis asperifolia Achnatherum hymenoides Ampelodesmos mauritanica Piptochaetium avenaceum  Phaenosperma globosum Brachyelytrum aristosum  Bambusa bambosa | 69596  IGS |  | GTATGTGGCT-------------CTTTCTACAC  GTATGTGGCT-------------CTTTCTACAC  GTATGTGGCT-------------CTTTCTACAC  GTATGTGGCT-------------CTTTCTACAC  GTATGTGGCT-------------CTTTCTACAG  GTATGTGGCT-------------CTTTCTACCC  GTATGTGGCT-------------CTTTCTACCC  GTATGTGGCT-------------CTTTCTATAC  GTATGTGGCT-------------CTTTCTACAC  GTATGTGGCT-------------CTTTCTACAC  GTATGTGGCT-------------CTGTATACAA  GTCTGTGGCT-------------CTTTCTACAC  GTATGTGGTT-------------CTTTCTACAC  GTATGTGGCT-------------CTTTCTACAC  GTATGTGGCT-------------CTTTCTACAC  GTATGTGGCT-------------CTTTCTACAC  GTATGTGGCT-------------CTTTCTACAC  GTATGTGCCT-------------CTTTCTACAC  GTATGTGGCT-------------CTTTCTACAC  GTATGTGGCT-------------CTTTATACAC  ATATGTGGCT-------------CTTTCTACAC  ATATGCGGCT-------------CTTTCTACAC  GTATGTGGCTTTTTCTACACACACTTTCTACAC  GTATGTGGCTTTTTCTACACACACTTTCTACAC  GTATGTGGCTTTTTCTACACACACTTTCTACAC  GTATGTGGCTTTTTCTACACACACTTTCTACAC  GTATGTGGCTTTTTCTACACACACTTTCTACAC  GTATGTGGCTTTTTCTACACACACTTTCTACAC  GTATGTGGCTTTTTCTACACACACTTTCTACAC  GTATGTGGCTTTTTCTACACACACTTTCTACAC  GTATGTGGCTTTTTCTACACACACTTTCTACAC  GTATGTGACTTTTTCTACACACACTTTCTACAC  GTATGTGACTTTTTCTACACACACTTTCTACAC  GTATGTGGCTTTTTCTACACACACTTTCTACAC  GTATGTGGGTTTTTCTACACACACTTTCTACAC  GTATGTGGCT-------------CTTTCTACAC  GTATGTGGCT-------------CTTTCTACAC  GTGTGTGGAT-------------CTTTCTACAT  GTGTGTGGAT-------------CTTTCTACAT  GTATGTGGCT-------------CTTTTTACAC  GTATGCGGCT-------------CTTTCTACAC  GTATGTGGCT-------------CTTTCTACAC  GTATGTGGCT-------------CTTTCTACAC  GTATGCGGCT-------------CTTTCTACAC  GTATGTGGCT-------------CTTTCTACAC  GTATGTGGTT-------------CTTTCTACAC |  | Bromeae+Triticeae |
| 100 | Schedonorus arundinaceus Schedonorus arundinaceus[C] Schedonorus pratensis  Festuca altissima  Festuca ovina  Lolium multiflorum  Lolium perenne  Dactylis glomerata  Helictochloa hookeri  Deschampsia antarctica  Poa palustris  Phleum alpinum  Briza sp  Puccinellia nuttalliana  Ammophila breviligulata  Agrostis stolonifera  Hierochloe odorata  Anthoxanthum odoratum  Phalaris arundinacea  Torreyochloa  Avena sativa  Trisetum cernuum  Aegilops cylindrica  Aegilops geniculata  Aegilops speltoides  Aegilops tauschii  Triticum aestivum  Triticum monococcum aegilopoides Triticum monococcum (modified) Triticum urartu (modified)  Hordeum jubatum  Hordeum vulgare  Hordeum vulgare spontaneum Secale cereale  Bromus vulgaris  Brachypodium distachyon  Diarrhena obovata  Melica mutica  Melica subulata  Oryzopsis asperifolia Achnatherum hymenoides Ampelodesmos mauritanica Piptochaetium avenaceum  Phaenosperma globosum Brachyelytrum aristosum  Bambusa bambosa | 69731  IGS |  | CGAG-TTTT---------------CTTTTAA  CGAGTTTTT---------------CTTTTAA  CGAG-TTTT---------------CTTTTAA  CGAG-TTTT---------------CTTTTAA  CGAG-TTTT---------------CTTTTAA  CGAG-TTTT---------------CTTTTAA  CGAG-TTTT---------------CTTTTAA  CGAG-TTTT---------------CCTTTAA  CGAT-TTTT---------------CTTTTAA  CAAG-TTTT---------------CTTTTAA  CGAG-TTTT---------------CTTTTAA  CGAG-TTTT---------------ATTTTAA  CGAG-TTTT---------------ATTTTAA  CGAG-TTTT---------------GTTTTAA  CGAG-TTTT---------------ATTTTAA  CGAG-TTTT---------------ATTTTAA  CGAG-TTTT---------------ATTTTAA  CGAG-TTTT---------------ATTTTAA  CGAG-TTTT---------------ATTTTAA  CGAG-TTTT---------------ATTTTAA  CGAG-TTTT---------------TTTTTAA  CGAG-TTTT---------------TTTTTAA  CGAG-TTTTATTTTATTT------ATTTTAA  CGAG-TTTTATTTTATTT------ATTTTAA  CGAG-TTTTATTTTATTT------ATTTTAA  CGAG-TTTTATTTTATTT------ATTTTAA  CGAG-TTTTATTTTATTT------ATTTTAA  CGAG-TTTTATTTTATTT------ATTTTAA  CGAG-TTTTATTTTATTT------ATTTTAA  CGAG-TTTTATTTTATTT------ATTTTAA  CGAGTTTTTTTTTTATTT------ATTTTAA  CGAG-TTTTTTTTTATTT------ATTTTAA  CGAG-TTTTTTTTTATTT------ATTTTAA  CGAG-TTTTATTTTATTTATTTTAATTTTAA  CGAG-TTTTATTTTATTT------ATTTTAA  CTAT-TTTT---------------ATTTTAA  CGAG-TTTT---------------TTTTTAA  CGAA-TTTT---------------CTTTTAA  CGAA-TTTT---------------CTTTTAA  CGAG-TTTT---------------ATTTTAA  CGAG-TTTT----------------TTTTTA  CGAG-TTTT---------------ATTTTAA  CGAG-TTTT---------------ATTTTAA  CGAG-TTTT----------------TTTTTA  CAAG-TTTT---------------ATTTTAA  CGAG-TTTT---------------ATTTTAA |  | Bromeae+Triticeae |
| 101 | Schedonorus arundinaceus Schedonorus arundinaceus[C] Schedonorus pratensis  Festuca altissima  Festuca ovina  Lolium multiflorum  Lolium perenne  Dactylis glomerata  Helictochloa hookeri  Deschampsia antarctica  Poa palustris  Phleum alpinum  Briza sp  Puccinellia nuttalliana  Ammophila breviligulata  Agrostis stolonifera  Hierochloe odorata  Anthoxanthum odoratum  Phalaris arundinacea  Torreyochloa  Avena sativa  Trisetum cernuum  Aegilops cylindrica  Aegilops geniculata  Aegilops speltoides  Aegilops tauschii  Triticum aestivum  Triticum monococcum aegilopoides Triticum monococcum (modified) Triticum urartu (modified)  Hordeum jubatum  Hordeum vulgare  Hordeum vulgare spontaneum Secale cereale  Bromus vulgaris  Brachypodium distachyon  Diarrhena obovata  Melica mutica  Melica subulata  Oryzopsis asperifolia Achnatherum hymenoides Ampelodesmos mauritanica Piptochaetium avenaceum  Phaenosperma globosum Brachyelytrum aristosum  Bambusa bambosa | 71840  IGS |  | TCAAATT----AGCATCT  TCAAATT----AGCATCT  TCAAATT----AGCAGCT  TCAAATT----AGCATCT  TCAAATT----AGCATCT  TCAAATT----AGCAGCT  TCAAATT----AGCAGCT  TCAAATT----AGCATCT  TTAAATT----AGCATCT  TCAAATT----AGCATCT  TCAAATT----AGCATCT  TCAAATT----AGCATCT  TCAAATT----AGCATCT  TAAAATT----AGCATCT  TCAAATT----AGCATCT  TCAAATT----AGCATCT  TCAAATT----AGCATCT  TCAAATT----AGCATCT  TCAAATT----AGCATCT  ACAAATT----AGCATCT  TCAAATT----AGCATCT  TCAAATT----AGCATCT  TCAAATTAGCAAGCATCT  TCAAATTAGCAAGCATCT  TCAAATTAGCAAGCATCT  TCAAATTAGCAAGCATCT  TCAAATTAGCAAGCATCT  TCAAATTAGCAAGCATCT  TCAAATTAGCAAGCATCT  TCAAATTAGCAAGCATCT  TCAAATT----AGCATCT  TCAAATT----AGCATCT  TCAAATT----AGCATCT  TCAAATTAGCAAGCATCT  TCAAATT----AGCATCT  TCAAATT----AGCATCT  TCAAATT----AGCATCT  TCAAATT----AGCATCT  TCAAATT----AGCATCT  TCAAATA----AGCATCT  TCAAATA----AGCATCT  TCAAATA----AGCATCT  TCAAATA----AGCATCT  TCAAATT----AGCATCT  TCAAATT----AGCATCT  TCAAATT----AGCATCT | Ssmp | Aegilops+Triticum+Secale |
| 102 | Schedonorus arundinaceus Schedonorus arundinaceus[C] Schedonorus pratensis  Festuca altissima  Festuca ovina  Lolium multiflorum  Lolium perenne  Dactylis glomerata  Helictochloa hookeri  Deschampsia antarctica  Poa palustris  Phleum alpinum  Briza sp  Puccinellia nuttalliana  Ammophila breviligulata  Agrostis stolonifera  Hierochloe odorata  Anthoxanthum odoratum  Phalaris arundinacea  Torreyochloa  Avena sativa  Trisetum cernuum  Aegilops cylindrica  Aegilops geniculata  Aegilops speltoides  Aegilops tauschii  Triticum aestivum  Triticum monococcum aegilopoides Triticum monococcum (modified) Triticum urartu (modified)  Hordeum jubatum  Hordeum vulgare  Hordeum vulgare spontaneum Secale cereale  Bromus vulgaris  Brachypodium distachyon  Diarrhena obovata  Melica mutica  Melica subulata  Oryzopsis asperifolia Achnatherum hymenoides Ampelodesmos mauritanica Piptochaetium avenaceum  Phaenosperma globosum Brachyelytrum aristosum  Bambusa bambosa | 71917  IGS |  | TCT----A--------TTTCTAC  TCT----A--------TTTCTAC  TCT----A--------TTTCTAC  TCT----A--------TTTCTAC  TCT----A--------TTTCTAC  TCT----A--------TTTCTAC  TCT----A--------TTTCTAC  TCT----A--------TTTCTAC  TCT----A--------TTTCTAC  TCT----A--------TTTCTAC  TCT----A--------TTTCTAC  TCT----A--------TTTCTAC  TCT----A--------TTTCTAC  TCT----A--------TTTCTAC  TCT----A--------TTTCTAC  TCT----A--------TTTCTAC  TCC----A--------TTTCTAC  TCC----A--------TTTCTAC  TCT----A--------TTTCTAC  TCT----A--------TTTCTAC  TCTTTCTA--------TTTCTAC  TCTTTCTA--------TTTCTAC  TCT----A--------TTTATAC  TCT----A--------TTTATAC  TCT----A--------TTTATAC  TCT----A--------TTTATAC  TCT----A--------TTTATAC  TCT----A--------TTTATAC  TCT----A--------TTTATAC  TCT----A--------TTTATAC  TCT----A--------TTTATAC  TCT----A--------TTTATAC  TCT----A--------TTTATAC  TCT----A--------TTTATAC  TCT----A--------TTTATAC  TCT----A--------TTTCTAC  TCT----A--------TTTCTAC  TCT----A--------TTTCTAC  TCT----A--------TTTCTAC  TCT----ATTTCTACCTTTCTAC  TCT----A--------TTTCTAC  TCT----ATTTCTACCTTTCTAC  TCT----A--------TTTCTAC  TCT----A--------TTTCTAC  TCC----ATTTATACCTTTCTAG  TCT----A--------TTTCTAC | ssmp | Homoplasy |
| 103 | Schedonorus arundinaceus Schedonorus arundinaceus[C] Schedonorus pratensis  Festuca altissima  Festuca ovina  Lolium multiflorum  Lolium perenne  Dactylis glomerata  Helictochloa hookeri  Deschampsia antarctica  Poa palustris  Phleum alpinum  Briza sp  Puccinellia nuttalliana  Ammophila breviligulata  Agrostis stolonifera  Hierochloe odorata  Anthoxanthum odoratum  Phalaris arundinacea  Torreyochloa  Avena sativa  Trisetum cernuum  Aegilops cylindrica  Aegilops geniculata  Aegilops speltoides  Aegilops tauschii  Triticum aestivum  Triticum monococcum aegilopoides Triticum monococcum (modified) Triticum urartu (modified)  Hordeum jubatum  Hordeum vulgare  Hordeum vulgare spontaneum Secale cereale  Bromus vulgaris  Brachypodium distachyon  Diarrhena obovata  Melica mutica  Melica subulata  Oryzopsis asperifolia Achnatherum hymenoides Ampelodesmos mauritanica Piptochaetium avenaceum  Phaenosperma globosum Brachyelytrum aristosum  Bambusa bambosa | 71975  IGS |  | TGCAGTA-----GAATGA  TGCAGTA-----GAATGA  TGCAGTA-----GAATGA  TGCAGTA-----GAATGA  TGCAGTA-----GAATGA  TGCAGTA-----GAATGA  TGCAGTA-----GAATGA  TACAGTA-----GAATGA  TGCAATA-----GAATGA  TGCAGTA-----GAATGA  TGTAGTA-----GAATGA  TGTAGTA-----GAATGA  TGCAATG-----GAATGA  TGCAGTA-----GAATGA  TGCAGTAGAATGGAATGA  TGCAGTAGAATGGAATGA  TGCAGTAGAATGGAATGA  TGCAGTAGAATGGAATGA  TGCAGTA-----GAATGA  TGCAGTA-----GAATGA  TGCAGTA-----GAATCA  TGCAGTA-----GAATGA  TGCAGTA-----GAATGA  TGCAGTA-----GAATGA  TGCAGTA-----GAATGA  TGCAGTA-----GAATGA  TGCAGTA-----GAATGA  TGCAGTA-----GAATGA  TGCAGTA-----GAATGA  TGCAGTA-----GAATGA  TGCAGTA-----GAATGA  TGCAGTA-----GAATGC  TGCAGTA-----GAATGC  TGCAGTA-----GAATGA  TGCAGTA-----GAATGA  TGCAGTA-----GAATGA  TGCAGTA-----GAATGA  TGCAGTA-----GAATGA  TGCAGTA-----GAATGA  TGCAGTA-----GAATGA  TGCAGTA-----GAATGA  TGCAGTA-----GAATGA  TGCAGTA-----GAATGA  TGCAGTA-----GAATGA  TGCAGTA-----GAATGA  TGCAGTA-----GAATGA | Ssmp | Homoplasy |
| 104 | Schedonorus arundinaceus Schedonorus arundinaceus[C] Schedonorus pratensis  Festuca altissima  Festuca ovina  Lolium multiflorum  Lolium perenne  Dactylis glomerata  Helictochloa hookeri  Deschampsia antarctica  Poa palustris  Phleum alpinum  Briza sp  Puccinellia nuttalliana  Ammophila breviligulata  Agrostis stolonifera  Hierochloe odorata  Anthoxanthum odoratum  Phalaris arundinacea  Torreyochloa  Avena sativa  Trisetum cernuum  Aegilops cylindrica  Aegilops geniculata  Aegilops speltoides  Aegilops tauschii  Triticum aestivum  Triticum monococcum aegilopoides Triticum monococcum (modified) Triticum urartu (modified)  Hordeum jubatum  Hordeum vulgare  Hordeum vulgare spontaneum Secale cereale  Bromus vulgaris  Brachypodium distachyon  Diarrhena obovata  Melica mutica  Melica subulata  Oryzopsis asperifolia Achnatherum hymenoides Ampelodesmos mauritanica Piptochaetium avenaceum  Phaenosperma globosum Brachyelytrum aristosum  Bambusa bambosa | 72014  IGS |  | ACTAACAT----TAGC  ACTAACAT----TAGC  ACTAACAT----TAGC  ACTAA-AT----TAGC  ACTAAATTAACTTAGC  ACTAATAT----TAGC  ACTAATAT----TAGC  ACTAAATTAACTTAGC  ACTAAATTAACTTAGC  ACTAAATTAACTTAGC  ACTAAATTAACTTAGC  ACTAAATTAACTTAGC  ACTAAATTAACTTAGC  ACTAAATTAACTTAGC  ACTAAATTAACTTAGC  ACTAAATTAACTTAGC  ACTAAATTAACTTAGC  ACTAAATTAACTTAGC  ACTAAATAAACTTAGC  ACTAAATTAACTTAGC  ACTAAATTAATTTAGC  ACTAAATTAATTTAGC  ACTAGATTAGCTTAGC  ACTAGATTAGCTTAGC  ACTAGATTAGCTTAGC  ACTAGATTAGTTTAGC  ACTAGATTAGCTTAGC  ACTAGATTAGCTTAGC  ACTAGATTAGCTTAGC  ACTAGATTAGCTTAGC  ACTAGATTAGCTTAGC  ACTAGATTAGCTTAGC  ACTAGATTAGCTTAGC  ACTAGATTAGCTTAGC  ACTAGATTAGCTTAGC  ACTAGATTAGCTTAGC  ACTAGATTAGCTTAGC  ACTAGATTAGCTTAGC  ACTAGATTAGCTTAGC  ACTAGATTAGCTTAGC  ACTAGATTAGCTTAGC  ACTAGATTAGCTTAGC  ACTAGATTAGCTTAGC  ACTAGATTAGCTTAGC  ACTAGATTAGCTTAGC  ACTAGATTAGCTTAGC |  | Lolium+Schedonorus+Festuca altissima |
| 105 | Schedonorus arundinaceus Schedonorus arundinaceus[C] Schedonorus pratensis  Festuca altissima  Festuca ovina  Lolium multiflorum  Lolium perenne  Dactylis glomerata  Helictochloa hookeri  Deschampsia antarctica  Poa palustris  Phleum alpinum  Briza sp  Puccinellia nuttalliana  Ammophila breviligulata  Agrostis stolonifera  Hierochloe odorata  Anthoxanthum odoratum  Phalaris arundinacea  Torreyochloa  Avena sativa  Trisetum cernuum  Aegilops cylindrica  Aegilops geniculata  Aegilops speltoides  Aegilops tauschii  Triticum aestivum  Triticum monococcum aegilopoides Triticum monococcum (modified) Triticum urartu (modified)  Hordeum jubatum  Hordeum vulgare  Hordeum vulgare spontaneum Secale cereale  Bromus vulgaris  Brachypodium distachyon  Diarrhena obovata  Melica mutica  Melica subulata  Oryzopsis asperifolia Achnatherum hymenoides Ampelodesmos mauritanica Piptochaetium avenaceum  Phaenosperma globosum Brachyelytrum aristosum  Bambusa bambosa | 73123  IGS |  | CCATTTTGGATTGGAAA  CCATTTTGGATTGGAAA  CTATTTTAGATTGGAAA  CTATT-----TTGGAAA  CTATT-----TTGGAAA  CTATTTTAGATTGGAAA  CTATTTTAGATTGGAAA  CTATT-----TTGGAAA  CCATT-----TTGGAAA  CTATT-----TTGGAAA  CTATT-----TTGGAAA  CTATT-----TTGGAAA  CTATT-----TTGGAAC  CTATT-----TTGGAAA  CTATT-----TTGGAAA  CTATT-----TTGGAAA  CTATT-----TTGGAAA  CTATT-----TTGGAAA  CTATT-----TTGGAAA  CTATT-----TTGGAAA  CTATT-----TTGGAAA  CTATT-----TTGGAAA  CTATT-----TTGGAAA  CTATT-----TTGGAAA  CTATT-----TTGGAAA  CTATT-----TTGGAAA  CTATT-----TTGGAAA  CTATT-----TTGGAAA  CTATT-----TTGGAAA  CTATT-----TTGGAAA  CTTTT-----TTGGAAA  CTTTT-----TTGGAAA  CTTTT-----TTGGAAA  CTATT-----TTGGAAA  CTATT-----TTGGAAA  CTATT-----TTGGAAA  CTATT-----TTGGAAA  CTTTT-----TTGGAAA  CTTTT-----TTGGAAA  CTATT-----TTGGAAA  CTTTT-----TTGGAAA  CTATT-----TTGGAAA  CTATT-----TTGGAAA  CTTTT-----TTGGAAA  CTATT-----TTGGAAA  CTATT-----TTGGAAA |  | Lolium+Schedonorus |
| 106 | Schedonorus arundinaceus Schedonorus arundinaceus[C] Schedonorus pratensis  Festuca altissima  Festuca ovina  Lolium multiflorum  Lolium perenne  Dactylis glomerata  Helictochloa hookeri  Deschampsia antarctica  Poa palustris  Phleum alpinum  Briza sp  Puccinellia nuttalliana  Ammophila breviligulata  Agrostis stolonifera  Hierochloe odorata  Anthoxanthum odoratum  Phalaris arundinacea  Torreyochloa  Avena sativa  Trisetum cernuum  Aegilops cylindrica  Aegilops geniculata  Aegilops speltoides  Aegilops tauschii  Triticum aestivum  Triticum monococcum aegilopoides Triticum monococcum (modified) Triticum urartu (modified)  Hordeum jubatum  Hordeum vulgare  Hordeum vulgare spontaneum Secale cereale  Bromus vulgaris  Brachypodium distachyon  Diarrhena obovata  Melica mutica  Melica subulata  Oryzopsis asperifolia Achnatherum hymenoides Ampelodesmos mauritanica Piptochaetium avenaceum  Phaenosperma globosum Brachyelytrum aristosum  Bambusa bambosa | 73322  IGS |  | TTCTT----AATCCTCTA  TTCTT----AATCCTCTA  TTCTT----AATCCTCTA  TTCTT----AATCCTCTA  TTCTT----AATCCTCTA  TTCTT----AATCCTCTA  TTCTT----AATCCTCTA  TTCTT----AATCCTCTA  TTCTT----AATCCTCTA  TTCTT----AATCCTCTA  TTCTTAATCAATCCTCTA  TTCTTAATCAATCCTCTA  TTCTT----AATCCTCTA  TTCTTAATCAATCCTCTA  TTCTT----AATCCTCTA  TTCTT----AATCCTCTA  TTCTT----AATCCTCTA  TTCTT----AATCCTCTA  TTCTT----AATCCTCTA  TTCTT----AATCCTCTA  TTTTT----AATCCTCTA  TTTTT----AATCCTCTA  TTCTT----AATCGTCTA  TTCTT----AATCGTCTA  TTCTT----AATCGTCTA  TTCTT----AATCGTCTA  TTCTT----AATCGTCTA  TTCTT----AATCGTCTA  TTCTT----AATCGTCTA  TTCTT----AATCGTCTA  TTCNT----AATCGTCTA  TTCTT----AATCGTCTA  TTCTT----AATCGTCTA  TTCTT----AATCGTCTA  TTCTT----AATCGTCTA  TTGTT----AATCGTCTA  TTCTT----AATCCTCTA  TTCTT----AATCCTCTA  TTCTT----AATCCTCTA  TTCTT----AATCCTCTA  TTCTT----AATCCTTTA  TTCTT----AATCCTCTA  TTCTT----AATCCTCTA  TTCTT----AATCTTCTA  TTCTT----AATCCTCTA  TTCTT----AATCCTCTA | ssmp | Coleanthinae+Poeae |
| 107 | Schedonorus arundinaceus Schedonorus arundinaceus[C] Schedonorus pratensis  Festuca altissima  Festuca ovina  Lolium multiflorum  Lolium perenne  Dactylis glomerata  Helictochloa hookeri  Deschampsia antarctica  Poa palustris  Phleum alpinum  Briza sp  Puccinellia nuttalliana  Ammophila breviligulata  Agrostis stolonifera  Hierochloe odorata  Anthoxanthum odoratum  Phalaris arundinacea  Torreyochloa  Avena sativa  Trisetum cernuum  Aegilops cylindrica  Aegilops geniculata  Aegilops speltoides  Aegilops tauschii  Triticum aestivum  Triticum monococcum aegilopoides Triticum monococcum (modified) Triticum urartu (modified)  Hordeum jubatum  Hordeum vulgare  Hordeum vulgare spontaneum Secale cereale  Bromus vulgaris  Brachypodium distachyon  Diarrhena obovata  Melica mutica  Melica subulata  Oryzopsis asperifolia Achnatherum hymenoides Ampelodesmos mauritanica Piptochaetium avenaceum  Phaenosperma globosum Brachyelytrum aristosum  Bambusa bambosa | 73576  IGS |  | CAAATTTATTGTGATTGTGA  CAAATTTATTGTGATTGTGA  CAAATTTATTGTGATTGTGA  CAAATTTATTGTGATTGTGA  CAAATTTATTGTGATTGTGA  CAAATTTATTGTGATTGTGA  CAAATTTATTGTGATTGTGA  CAAATTTAGTGTGATTGTGA  CAAATTTATTGTGATTATGA  CAAATTTATTGGGATTGTGA  CAAATTT------ATTGTGA  CAAATTTATTGTGATTGTGA  CAAATTTTTTGTGATTGTGA  CAAATTTATTGTGATTGTGA  CAAATTTCTTGTGATTGTGA  CAAATTTCTTGTGATTGTGA  CAAATTTCTTGTGATTGTGA  CAAATTTCTTGTGATTGTGA  CAAATTTCTTGTGATTGTGA  CAAATTTTTTGTGATTGTGA  CAAATTTCTTGTGATTGTGA  CAAATTTCTTGTGATTGTGA  AAAATTTCTTGTGATTGTGA  AAAATTTCTTGTGATTGTGA  CAAATTTCTTGTGATTGTGA  AAAATTTCTTGTGATTGTGA  CAAATTTCTTGTGATTGTGA  AAAATTTCTTGTGATTGTGA  AAAATTTCTTGTGATTGTGA  AAAATTTCTTGTGATTGTGA  CAAATTTCTTGTGATTGTAA  CAAATTTCTTGTGATTGTAA  CAAATTTCTTGTGATTGTAA  CAAATTTATTGTGATTGTGA  CAAATTTCTTGTGATTGTGA  CAAATTTATTGTGATTGTGA  CAAATTTATTGTGATTGTGA  CTAATTT------CTTGTGA  CTTATTT------CTTGTGA  CAAATTT------CTTGTGA  CAAATTT------CTTGTGA  CAAATTT------CTTGTGA  CAAATTT------CTTGTGA  CAAATTT------CTTGTGA  CTCATTT------CTTGTGA  CAAATTT------CTTGTGA | ssmp | Homoplasy |
| 108 | Schedonorus arundinaceus Schedonorus arundinaceus[C] Schedonorus pratensis  Festuca altissima  Festuca ovina  Lolium multiflorum  Lolium perenne  Dactylis glomerata  Helictochloa hookeri  Deschampsia antarctica  Poa palustris  Phleum alpinum  Briza sp  Puccinellia nuttalliana  Ammophila breviligulata  Agrostis stolonifera  Hierochloe odorata  Anthoxanthum odoratum  Phalaris arundinacea  Torreyochloa  Avena sativa  Trisetum cernuum  Aegilops cylindrica  Aegilops geniculata  Aegilops speltoides  Aegilops tauschii  Triticum aestivum  Triticum monococcum aegilopoides Triticum monococcum (modified) Triticum urartu (modified)  Hordeum jubatum  Hordeum vulgare  Hordeum vulgare spontaneum Secale cereale  Bromus vulgaris  Brachypodium distachyon  Diarrhena obovata  Melica mutica  Melica subulata  Oryzopsis asperifolia Achnatherum hymenoides Ampelodesmos mauritanica Piptochaetium avenaceum  Phaenosperma globosum Brachyelytrum aristosum  Bambusa bambosa | 73592  IGS |  | GTGAGCAAATAGAATTG  GTGAGCAAATAGAATTG  GTGAGCAAATAGAATTG  GTGAGCAAATAGAATTG  GTGAGCAAATAGAATTG  GTGAGCAAATAGAATTG  GTGAGCAAATAGAATTG  GTGAGCAAATAGAATTG  ATGAGCAAATAGAATTG  GTGAGCAAATAGAATTG  GTGAGCAAATAGAATTG  GTGAGCAAATAGAATTG  GTGAGCAAATAGAATTG  GTGAGTAAATAGAATTG  GTGAGCAAATAGAATTG  GTGAGCAAATAGAATTG  GTGAGCAAATAGAATTG  GTGAGCAAATAGAATTG  GTGAGCAAATAGAATTG  GTGAGCAAATAGAATTG  GTGAGCAAATAGAATTG  GTGAGCAAATAGAATTG  GTGAGCA-----AATTG  GTGAGCA-----AATTG  GTGAACAAATAGAATTG  GTGAGCA-----AATTG  GTGAACAAATAGAATTG  GTGAGCA-----AATTG  GTGAGCA-----AATTG  GTGAGCA-----AATTG  GTAAGCAAATAGAATTG  GTAAGCAAATAGAATTG  GTAAGCAAATAGAATTG  GTGAGCAAATAGAATTG  GTGAGCAAATAGAATTG  GTGAGCAAATAGAATTG  GTGAGCAAATAGAATTG  GTGAGCAAATAGAATTG  GTGAGCAAATAGAATTG  GTGAGCAAATAGAATTG  GTGAGCAAATAGAATTG  GTGAGCAAATAGAATTG  GTGAGCAAATAGAATTG  GTGAGCAAATAGAATTG  GTGAACAAATAGAATTG  GTGAGCAAATAGAATTG |  | Aegilops+Triticum minus A. speltoides and T. aestivum |
| 109 | Schedonorus arundinaceus Schedonorus arundinaceus[C] Schedonorus pratensis  Festuca altissima  Festuca ovina  Lolium multiflorum  Lolium perenne  Dactylis glomerata  Helictochloa hookeri  Deschampsia antarctica  Poa palustris  Phleum alpinum  Briza sp  Puccinellia nuttalliana  Ammophila breviligulata  Agrostis stolonifera  Hierochloe odorata  Anthoxanthum odoratum  Phalaris arundinacea  Torreyochloa  Avena sativa  Trisetum cernuum  Aegilops cylindrica  Aegilops geniculata  Aegilops speltoides  Aegilops tauschii  Triticum aestivum  Triticum monococcum aegilopoides Triticum monococcum (modified) Triticum urartu (modified)  Hordeum jubatum  Hordeum vulgare  Hordeum vulgare spontaneum Secale cereale  Bromus vulgaris  Brachypodium distachyon  Diarrhena obovata  Melica mutica  Melica subulata  Oryzopsis asperifolia Achnatherum hymenoides Ampelodesmos mauritanica Piptochaetium avenaceum  Phaenosperma globosum Brachyelytrum aristosum  Bambusa bambosa | 73619  IGS |  | TCAATTA-----AGTTCAATTT  TCAATTA-----AGTTCAATTT  TCAATTA-----AGTTCAATTT  TCAATTA-----AGTTCAACTT  TCAATTA-----AGTTCAACTT  TCAATTA-----AGTTCAATTT  TCAATTA-----AGTTCAATTT  TCAATTA-----AGTTCAACTT  TCAATTA-----AGTTCAATTT  TTAATTA-----AGTTCAACTT  TCAATTA-----AATTCAACTT  TCAATTA-----AGTTCAACTT  TCAATTA-----AGTTCAACTT  TCAATTA-----AGTTCAACTT  TCAATTA-----AGTTCAACTT  TCAATTA-----AGTTCAACTT  TCAATTA-----AGTTCAACCT  TCAATTA-----AGTTCAACCT  TCAATTA-----AGTTCAACTT  TCAATTA-----AGTTCAACTT  TCAATTA-----AGTTCAACTT  TCAATTA-----AGTTCAACTT  TTAATTA-----AGTTCAACTT  TTAATTA-----AGTTCAACTT  TTAATTA-----AGTTCAACTT  TTAATTA-----AGTTCAACTT  TTAATTA-----AGTTCAACTT  TTAATTA-----AGTTCAACTT  TTAATTA-----AGTTCAACTT  TTAATTA-----AGTTCAACTT  TTAATTA-----AGTTTAACTT  TTAATTA-----AGTTTAACTT  TTAATTA-----AGTTTAACTT  TTAATTA-----AGTTCAACTT  TTAATTA-----AGTTCAACTT  TCGATTA-----AGTTCAATTT  TCAATTA-----AGTTCAACTT  TCAATTAAGTTCAGTTCAATTT  TCAATTAAGTTCAGTTCAATTT  TCAATTA-----AGTTCAACTT  TCAATTA-----AGTTCAACTT  TCAATTA-----AGTTCAACTT  TCAATTA-----AGTTCAACTT  TCAATTA-----AGTTCAACTT  TCAATTA-----AGTTCAACTT  TCAATTA-----AGTTCAACTT | Ssmp | Melica |
| 110 | Schedonorus arundinaceus Schedonorus arundinaceus[C] Schedonorus pratensis  Festuca altissima  Festuca ovina  Lolium multiflorum  Lolium perenne  Dactylis glomerata  Helictochloa hookeri  Deschampsia antarctica  Poa palustris  Phleum alpinum  Briza sp  Puccinellia nuttalliana  Ammophila breviligulata  Agrostis stolonifera  Hierochloe odorata  Anthoxanthum odoratum  Phalaris arundinacea  Torreyochloa  Avena sativa  Trisetum cernuum  Aegilops cylindrica  Aegilops geniculata  Aegilops speltoides  Aegilops tauschii  Triticum aestivum  Triticum monococcum aegilopoides Triticum monococcum (modified) Triticum urartu (modified)  Hordeum jubatum  Hordeum vulgare  Hordeum vulgare spontaneum Secale cereale  Bromus vulgaris  Brachypodium distachyon  Diarrhena obovata  Melica mutica  Melica subulata  Oryzopsis asperifolia Achnatherum hymenoides Ampelodesmos mauritanica Piptochaetium avenaceum  Phaenosperma globosum Brachyelytrum aristosum  Bambusa bambosa | 76646  IGS |  | CTAAAATA----ACTGA-  CTAAAATA----ACTGA-  CTAAAATA----ACTGA-  CTAAAATA----ACTGA-  CTAAAATA----ACTGA-  CTAAAATA----ACTGA-  CTAAAATA----ACTGA-  CTAAAATA----ACTGA-  CTAAAATA----ACTGA-  CTAAAATA----ACTGA-  CTAAAATA----ACTGA-  CTAAAATA----ACTGA-  CTAAAATA----ACTGAT  CTAAAATA----ACTGA-  CTAAAATA----ACTGAT  CTAAAATA----ACTGAT  CTAAAATA----ACTGAT  CTAAAATA----ATTGAT  CTAAAATA----ACTGAT  CTAAAATA----ACTGAT  CTAAAATAACTGACTGAT  CTAAAATAACTGACTGAT  CTAAAATA----ACTGAT  CTAAAATA----ACTTAT  CTAAAATA----ACTGAT  CTAAAATA----ACTGAT  CTAAAATA----ACTGAT  CTAAAATA----ACTGAT  CTAAAATA----ACTGAT  CTAAAATA----ACTGAT  CTAAAATA----ACTGAT  CTAAAATA----ACTGAT  CTAAAATA----ACTGAT  GTAAAATA----ACTGAT  CTAAAATA----ACTAAT  CTAAAATA----ACTGAT  CTAAAATA----ACTGAT  CTAAAATA----ACTGAT  CTAAAATA----ACTGAT  CTAAAATA----ACTGAT  CTAAAATA----ACTGAT  CTAAAATA----ACTGAT  CTAAAATA----ACTGAT  CTAAAATA----ACTGAT  CTAAAATA----ACTGAT  CTAAAATA----ACCGAT | ssmp | Aveninae+Koelerinae |
| 111 | Schedonorus arundinaceus Schedonorus arundinaceus[C] Schedonorus pratensis  Festuca altissima  Festuca ovina  Lolium multiflorum  Lolium perenne  Dactylis glomerata  Helictochloa hookeri  Deschampsia antarctica  Poa palustris  Phleum alpinum  Briza sp  Puccinellia nuttalliana  Ammophila breviligulata  Agrostis stolonifera  Hierochloe odorata  Anthoxanthum odoratum  Phalaris arundinacea  Torreyochloa  Avena sativa  Trisetum cernuum  Aegilops cylindrica  Aegilops geniculata  Aegilops speltoides  Aegilops tauschii  Triticum aestivum  Triticum monococcum aegilopoides Triticum monococcum (modified) Triticum urartu (modified)  Hordeum jubatum  Hordeum vulgare  Hordeum vulgare spontaneum Secale cereale  Bromus vulgaris  Brachypodium distachyon  Diarrhena obovata  Melica mutica  Melica subulata  Oryzopsis asperifolia Achnatherum hymenoides Ampelodesmos mauritanica Piptochaetium avenaceum  Phaenosperma globosum Brachyelytrum aristosum  Bambusa bambosa | 76658  IGS |  | ACTGA-------TGAATTTT  ACTGA-------TGAATTTT  ACTGA-------TGAATTTT  ACTGA-------TGAATTTT  ACTGA-------TGAATTTT  ACTGA-------TGAATTTT  ACTGA-------TGAATTTT  ACTGA-------TGAATTTT  ACTGA-------TGAATTTT  ACTGA-------TGAATTTT  ACTGA-------TGAATTTT  ACTGA-------TGAATTTT  ACTGATGAATTATGAATTTC  ACTGA-------TGAATTTT  ACTGATGAATTATGAATTTC  ACTGATGAATTATGAATTTC  ACTGATGAATTATGAATTTC  ATTGATGAAATCTGAATTTC  ACTGATGAATTATGAATTTC  ACTGATGAATTATGAATTTG  ACTGATGAATTATGAATTTC  ACTGATGAATTATGAATTTC  ACTGATGAATTCTGAATTTT  ACTTATGAATTATGAATTTT  ACTGATGAATTATGAATTTT  ACTGATGAATTCTGAATTTT  ACTGATGAATTATGAATTTT  ACTGATGAATTATGAATTTT  ACTGATGAATTATGAATTTT  ACTGATGAATTATGAATTTT  ACTGATGAATTATGAATTTT  ACTGATGAATTATGAATTTT  ACTGATGAATTATGAATTTT  ACTGATGAATTATGAATTTT  ACTAATGAATTATGAATTTT  ACTGATAAATTATGAATTTT  ACTGATGAATTATGAATTTT  ACTGATGAATTATGAATTTT  ACTGATGAATTATGAATTTT  ACTGATGAATTATGAATTTT  ACTGATGAATTATGAATTTT  ACTGATGAATTATGAATTTT  ACTGATGAGTTTTGAATTTT  ACTGATGAATTATGAATTTT  ACTGATGAATTATGAATTTT  ACCGATGAATTCTGAATTTT |  | Poeae clade 2 |
| 112 | Schedonorus arundinaceus Schedonorus arundinaceus[C] Schedonorus pratensis  Festuca altissima  Festuca ovina  Lolium multiflorum  Lolium perenne  Dactylis glomerata  Helictochloa hookeri  Deschampsia antarctica  Poa palustris  Phleum alpinum  Briza sp  Puccinellia nuttalliana  Ammophila breviligulata  Agrostis stolonifera  Hierochloe odorata  Anthoxanthum odoratum  Phalaris arundinacea  Torreyochloa  Avena sativa  Trisetum cernuum  Aegilops cylindrica  Aegilops geniculata  Aegilops speltoides  Aegilops tauschii  Triticum aestivum  Triticum monococcum aegilopoides Triticum monococcum (modified) Triticum urartu (modified)  Hordeum jubatum  Hordeum vulgare  Hordeum vulgare spontaneum Secale cereale  Bromus vulgaris  Brachypodium distachyon  Diarrhena obovata  Melica mutica  Melica subulata  Oryzopsis asperifolia Achnatherum hymenoides Ampelodesmos mauritanica Piptochaetium avenaceum  Phaenosperma globosum Brachyelytrum aristosum  Bambusa bambosa | 76839  petL | petL | TTATT-----TGAAT  TTATT-----TGAAT  TTATT-----TGAAT  TTATT-----TGAAT  TTATT-----TGAAT  TTATT-----TGAAT  TTATT-----TGAAT  TTATT-----TGAAT  TTATT-----TGAAT  TTATT-----TGAAT  TTATT-----TGAAT  TTATT-----TGAAT  TTATT-----TGAAT  TTATT-----TGAAT  TTATT-----TGAAT  TTATT-----TGAAT  TTATT-----TGAAT  TTATT-----TGAAT  TTATT-----TGAAT  TTATT-----TGAAT  TTATT-----TGAAT  TTATT-----TGAAT  TTATT-----TGAAT  TTATT-----TGAAT  TTATT-----TGAAT  TTATT-----TGAAT  TTATT-----TGAAT  TTATT-----TGAAT  TTATT-----TGAAT  TTATT-----TGAAT  TTATT-----TGAAT  TTATT-----TGAAT  TTATT-----TGAAT  TTATT-----TGAAT  TTATT-----TGAAT  TTATT-----TGAAT  TTATT-----TGAAT  TTATT-----TGAAT  TTATT-----TGAAT  TTATT-----TGAAT  TTATT-----TGAAT  TTATT-----TGAAT  TTATT-----TGAAT  TTATT-----TGAAT  TTATTTGAAATGAAT  TTATTTGAAATGAAT | ssmp | Homoplasy |
| 113 | Schedonorus arundinaceus Schedonorus arundinaceus[C] Schedonorus pratensis  Festuca altissima  Festuca ovina  Lolium multiflorum  Lolium perenne  Dactylis glomerata  Helictochloa hookeri  Deschampsia antarctica  Poa palustris  Phleum alpinum  Briza sp  Puccinellia nuttalliana  Ammophila breviligulata  Agrostis stolonifera  Hierochloe odorata  Anthoxanthum odoratum  Phalaris arundinacea  Torreyochloa  Avena sativa  Trisetum cernuum  Aegilops cylindrica  Aegilops geniculata  Aegilops speltoides  Aegilops tauschii  Triticum aestivum  Triticum monococcum aegilopoides Triticum monococcum (modified) Triticum urartu (modified)  Hordeum jubatum  Hordeum vulgare  Hordeum vulgare spontaneum Secale cereale  Bromus vulgaris  Brachypodium distachyon  Diarrhena obovata  Melica mutica  Melica subulata  Oryzopsis asperifolia Achnatherum hymenoides Ampelodesmos mauritanica Piptochaetium avenaceum  Phaenosperma globosum Brachyelytrum aristosum  Bambusa bambosa | 76854  IGS |  | TGACT------GAATAAGT  TGACT------GAATAAGT  TGACT------GAATAAGT  TGACT------GAATAAGT  TGACT------GAATAAGT  TGACT------GAATAAGT  TGACT------GAATAAGT  TGACT------GAATAAGT  TGACT------GAATAAGT  TGACT------GAATAAGT  TGACTTAATAATAATAAGT  TGACT------TAATAAGT  TAACT------GAATAAGT  TGACT------TAATAAGT  TGACT------GAATAAGT  TGACT------GAATAAGT  TGACT------TAATAAGT  TGACT------GAATAAGT  TAACT------GAATAAGT  TGAAT------GAATAAGT  TGACT------GAATAAGT  TGACT------GAATAAGT  TGACTGAATAATTATAATT  TGACTGAATAATTATAATT  TGACTGAATAATTATAATT  TGACTGAATAATTATAATT  TGACTGAATAATTATAATT  TGACTGAATAATTAGAATT  TGACTGAATAATTATAATT  TGACTGAATAATTATAATT  TGACTGAATAATAATAATT  TGACTGAATAATAATAATT  TGACTGAATAATAATAATT  TGACTGAATAATTATAATT  TGACTGAATAATTAGAATT  TGACT------GAATAAGT  TGACT------GAATAAGT  TGACT------GAATAAGT  TGACT------GAATAAGT  TGACT------GAATAAGT  TGACT------GAATAAGT  TGACT------GAATAAGT  TGACT------GAGTAAGT  TGACT------GAATAAGT  TGAAT------GAATAAGT  TGAAT------GAATAAGT | Ssmp | Homoplasy |
| 114 | Schedonorus arundinaceus Schedonorus arundinaceus[C] Schedonorus pratensis  Festuca altissima  Festuca ovina  Lolium multiflorum  Lolium perenne  Dactylis glomerata  Helictochloa hookeri  Deschampsia antarctica  Poa palustris  Phleum alpinum  Briza sp  Puccinellia nuttalliana  Ammophila breviligulata  Agrostis stolonifera  Hierochloe odorata  Anthoxanthum odoratum  Phalaris arundinacea  Torreyochloa  Avena sativa  Trisetum cernuum  Aegilops cylindrica  Aegilops geniculata  Aegilops speltoides  Aegilops tauschii  Triticum aestivum  Triticum monococcum aegilopoides Triticum monococcum (modified) Triticum urartu (modified)  Hordeum jubatum  Hordeum vulgare  Hordeum vulgare spontaneum Secale cereale  Bromus vulgaris  Brachypodium distachyon  Diarrhena obovata  Melica mutica  Melica subulata  Oryzopsis asperifolia Achnatherum hymenoides Ampelodesmos mauritanica Piptochaetium avenaceum  Phaenosperma globosum Brachyelytrum aristosum  Bambusa bambosa | 77197  IGS |  | AGGAGGTC------AAATTCGAGT  AGGAGGTC------AAATTCGAGT  AGGAGGTC------AAATTCGAGT  AGGAGGTC------AAATTCGAGT  AGGAGGTC------AAATTCGAGT  AGGAGGTC------AAATTCGAGT  AGGAGGTC------AAATTCGAGT  AGGAGGTC------AAATTCGAGT  AGGAGGTC------AAATTCCAGT  AGGAGGTC------AAATTCGAGT  AGGAGGTC------AAATTCGATT  AGGGGGTC------AAATTCGATT  AGGAGGTC------AAATTCGAGT  AGGAGGTC------AAATTCGAGT  AGGAGGTC------AAATTCGAGT  AGGAGGTC------AAATTCGAGT  AGGAGGTC------AAATTCGAGT  AGGAGGTC------AAATTCGAGT  AGGAGGTC------AAATTCGAGT  AGGAGGTC------AAATTCGAGT  AGGAGGTC------AAATTCGAGT  AGGAGGTC------AAATTCGAGT  AGGAGGTCAAATTGAAATTGGAGT  AGGAGGTCAAATTGAAATTGGAGT  AGGAGGTCAAATTGAAATTGGAGT  AGGAGGTCAAATTGAAATTGGAGT  AGGAGGTCAAATTGAAATTGGAGT  AGGAGGTCAAATTGAAATTGGAGT  AGGAGGTCAAATTGAAATTGGAGT  AGGAGGTCAAATTGAAATTGGAGT  AGGAGGTC------AAATTGGAGT  AGGAGGTC------AAATTGGAGT  AGGAGGTC------AAATTGGAGT  AGGAGGTCAAATTGAAATTGGAGT  AGGAGGTC------AAATTGGAGT  AGGAGGTC------AAATTGGAGT  AGGAGGTC------AAATTGGAGT  AGGAGGTC------AAATTGGAGT  AGGAGGTC------AAATTGGAGT  AGGAGGTC------AAATTGGAGT  AGGAGGTC------AAATTGGAGT  AGGAGGTC------AAATTGGAGT  AGGAGGTC------AAATTGGAGT  AGGAGGTC------AAATTGGAGT  GGGAGGTC------AAATTGGAGT  AGGAGGTC------AAATTGGAGT | Ssmp | Aegilops+Triticum+Secale |
| 115 | Schedonorus arundinaceus Schedonorus arundinaceus[C] Schedonorus pratensis  Festuca altissima  Festuca ovina  Lolium multiflorum  Lolium perenne  Dactylis glomerata  Helictochloa hookeri  Deschampsia antarctica  Poa palustris  Phleum alpinum  Briza sp  Puccinellia nuttalliana  Ammophila breviligulata  Agrostis stolonifera  Hierochloe odorata  Anthoxanthum odoratum  Phalaris arundinacea  Torreyochloa  Avena sativa  Trisetum cernuum  Aegilops cylindrica  Aegilops geniculata  Aegilops speltoides  Aegilops tauschii  Triticum aestivum  Triticum monococcum aegilopoides Triticum monococcum (modified) Triticum urartu (modified)  Hordeum jubatum  Hordeum vulgare  Hordeum vulgare spontaneum Secale cereale  Bromus vulgaris  Brachypodium distachyon  Diarrhena obovata  Melica mutica  Melica subulata  Oryzopsis asperifolia Achnatherum hymenoides Ampelodesmos mauritanica Piptochaetium avenaceum  Phaenosperma globosum Brachyelytrum aristosum  Bambusa bambosa | 77229  IGS |  | TCAAC-----TTTGTTTT--GGTA  TCAAC-----TTGGTTTTGGGTTA  TCAAC-----TTTGTTTT--GGTA  TCAAC-----TTTGTTTT--GTTA  TCAAC-----TTTGTTTT--GTTA  TCAAC-----TTTGTTTT--GGTA  TCAAC-----TTTGTTTT--GGTA  TCAACTTTGTTTTGTTTT--GTTA  TCAAC-----TTTGTTTT--GTTA  TCAAC-----TTTGTTTT--GTTA  TCAAC-----TTTGTTTT--CTTA  TCAAC-----TTTGTTTT--GTTA  TCAAC-----TTTGTTTT--GTTA  TCAAT-----TTTGTTTT--GTTA  TCAAC-----TTTGTTTT--GTTA  TCAAC-----TTTGTTTT--GTTA  TCAAC-----TTTGTTTT--GTTA  TCCAC-----TTTGTTTT--GTTA  TCAAC-----TTTGTTTT--GTTA  TCAAC-----TTTGTTTT--GTTA  TCAAT-----TTTGTTTT--GTTA  TCAAC-----TTTGTTTT--GTTA  TCAAT----------TTT--GTTA  TCCAT----------TTT--GTTA  TCAAT----------TTT--GTTA  TCAAT----------TTT--GTTA  TCAAT----------TTT--GTTA  TCAAT----------TTT--GTTA  TCAAT----------TTT--GTTA  TCAAT----------TTT--GTTA  TCAAT----------TTT--GTTA  TCAAT----------TTT--GTTA  TCAAT----------TTT--GTTA  TCTAT----------TTT--GTTA  TCAAT----------TTT--GTTA  TCAAC-----TTTCTTTT--GTTA  TCAAC-----TTTGTTTT--GTTA  TCAAC-----TTTGTTTT--GTTA  TCAAC-----TTTGTTTT--GTTA  TCAAC-----TTTGTTTT--ATTA  TCAAC-----TTTGTTTT--ATTA  TCAAC-----TTTGTTTT--ATTA  TCAAC-----TTTGTTTT--ATTA  TCAAC-----TTTGTTTT--GTTA  TCGAC-----TTTGTTTT--GTTA  TCAAC-----TTTGTTTT--GTTA | ssmp | Bromeae+Triticeae |
| 116 | Schedonorus arundinaceus Schedonorus arundinaceus[C] Schedonorus pratensis  Festuca altissima  Festuca ovina  Lolium multiflorum  Lolium perenne  Dactylis glomerata  Helictochloa hookeri  Deschampsia antarctica  Poa palustris  Phleum alpinum  Briza sp  Puccinellia nuttalliana  Ammophila breviligulata  Agrostis stolonifera  Hierochloe odorata  Anthoxanthum odoratum  Phalaris arundinacea  Torreyochloa  Avena sativa  Trisetum cernuum  Aegilops cylindrica  Aegilops geniculata  Aegilops speltoides  Aegilops tauschii  Triticum aestivum  Triticum monococcum aegilopoides Triticum monococcum (modified) Triticum urartu (modified)  Hordeum jubatum  Hordeum vulgare  Hordeum vulgare spontaneum Secale cereale  Bromus vulgaris  Brachypodium distachyon  Diarrhena obovata  Melica mutica  Melica subulata  Oryzopsis asperifolia Achnatherum hymenoides Ampelodesmos mauritanica Piptochaetium avenaceum  Phaenosperma globosum Brachyelytrum aristosum  Bambusa bambosa | 77359  trnW-CCA | trnW-CCA | CTAAGAGCG--------CTTTCA-TTT  CTAAGAGCG--------CTTTCATTTA  CTAAGAGCG--------CTTTCA-TTT  CTAAGAGCG--------CTTTCA-TTC  CTAAGAGCG--------CTTTCA-TTC  CTAAGAGCG--------CTTTCA-TTT  CTAAGAGCG--------CTTTCA-TTT  CTAAGAGCG--------CTTTCA-TTC  CTAAGAGCG--------CTTTCA-TTC  CTAAGAGCG--------CTTTCA-TTC  CTAAGAGCG--------CTTTCA-TTC  CTAAGAGCG--------CTTTCA-TTC  CTAAGAGCG--------CTTTCATTTC  CTAAGAGCG--------CTTTCA-TTC  CTAAGAGCG--------CTTTCA-TTC  CTAAGAGCG--------CTTTCA-TTT  CTAAGAGCGCTTTCATTCTTTCA-TTC  CTAAGAGCGCTTTCATTCTTTCA-TTC  CTAAGAGCG--------CTTTCA-TTC  CTAAGAGCG--------CTTTCA-TTC  CTAAGAGCG--------CTTTCA-TTC  CTAAGAGCG--------CTTTCA-TTC  CTAAGAGCG--------CTTTCA-TTC  CTAAGAGCG--------CTTTCA-TTC  CTAAGAGCG--------CTTTCA-TTC  CTAAGAGCG--------CTTTCA-TTC  CTAAGAGCG--------CTTTCA-TTC  CTAAGAGCG--------CTTTCA-TTC  CTAAGAGCG--------CTTTCA-TTC  CTAAGAGCG--------CTTTCA-TTC  CTAAGAGCG--------CTTTCA-TTC  CTAAGAGCG--------CTTTCA-TTC  CTAAGAGCG--------CTTTCA-TTC  CTAAGAGCG--------CTTTCA-TTC  CTAAGAGCG--------CTTTCA-TTC  CTAAGAGCG--------CTGTCA-TTC  CTAAGAGCG--------CTTTCA-TTC  CTAAGAGCG--------CTTTCA-TTC  CTAAGAGCG--------CTTTCA-TTC  CTAAGAGCG--------CTTTCA-TTC  CTAAGAGCG--------CTTTCA-TTC  CTAAGAGCG--------CTTTCA-TTC  CTAAGAGCG--------CTTTCA-TTC  CTAAGAGCG--------CTTTCA-TTC  CTAAGAGCG--------CTTTTA----  CTAAGAGCG--------CTTTCA---- | Ssmp | Anthoxanthinae |
| 117 | Schedonorus arundinaceus Schedonorus arundinaceus[C] Schedonorus pratensis  Festuca altissima  Festuca ovina  Lolium multiflorum  Lolium perenne  Dactylis glomerata  Helictochloa hookeri  Deschampsia antarctica  Poa palustris  Phleum alpinum  Briza sp  Puccinellia nuttalliana  Ammophila breviligulata  Agrostis stolonifera  Hierochloe odorata  Anthoxanthum odoratum  Phalaris arundinacea  Torreyochloa  Avena sativa  Trisetum cernuum  Aegilops cylindrica  Aegilops geniculata  Aegilops speltoides  Aegilops tauschii  Triticum aestivum  Triticum monococcum aegilopoides Triticum monococcum (modified) Triticum urartu (modified)  Hordeum jubatum  Hordeum vulgare  Hordeum vulgare spontaneum Secale cereale  Bromus vulgaris  Brachypodium distachyon  Diarrhena obovata  Melica mutica  Melica subulata  Oryzopsis asperifolia Achnatherum hymenoides Ampelodesmos mauritanica Piptochaetium avenaceum  Phaenosperma globosum Brachyelytrum aristosum  Bambusa bambosa | 77400  IGS |  | TATTTTTCTATTTCTAACA  TATTTTTCTATTTCTAACA  TATTTTTCTATTTCTAACA  TATTTTTCTATTTCTAACA  TATT------TTTCTAACA  TATTTTTCTATTTCTAACA  TATTTTTCTATTTCTAACA  TATT------TTTCTATCA  TATTTTTCTATTTCTAACA  TATTTTTCTATTTCTAACA  TATTTTTCTATTCCTAACG  TATTTTTCTATTCCTAACG  TATTTTTCTATTCCTAACG  TATTTTTCTATTCCTAACG  TATTTTTCTATTCCTAACG  TATTTTTCTATTCCTAACG  TATTTTTCTATTCCTAACG  TATTTTTCTATTCCTAACG  TATTTTTCTATTCCTAACG  TATTTTTCTATTCCTAACG  TATTTTTCTATTCCTAACG  TATTTTTCTATTCCTAACG  TCTTTTTCTATTCCTAATG  TCTTTTTCTATTCCTAATG  TCTTTTTCTATTCCTAATG  TCTTTTTCTATTCCTAATG  TCTTTTTCTATTCCTAATG  TCTTTTTCTATTCCTAATG  TCTTTTTCTATTCCTAATG  TCTTTTTCTATTCCTAATG  TCTTTTTCTATTCCTAATG  TCTTTTTCTATTCCTAATG  TCTTTTTCTATTCCTAATG  TCTTTTTCTATTCCTAATG  TCTTTTTCTATTCCTAACG  CTCTTTTCTACTCCTAACG  CCCTTTTCTACTCCTAACG  CCCTTTTCTACACGTAACG  CCCTTTTCTACTCGTAACG  CTGTTTTCTACTCCTAACG  CCGTTTTCTACTCCTAACG  CCGTTTTCTACTCCTAACG  CCGTTTTCTACTCCTAACG  GAAAACCCT-TTCCTAACG  CCCTTTTCTACTCCTAACG  CCCTTTTCTACTCCTAACG | Ssmp | Homoplasy |
| 118 | Schedonorus arundinaceus Schedonorus arundinaceus[C] Schedonorus pratensis  Festuca altissima  Festuca ovina  Lolium multiflorum  Lolium perenne  Dactylis glomerata  Helictochloa hookeri  Deschampsia antarctica  Poa palustris  Phleum alpinum  Briza sp  Puccinellia nuttalliana  Ammophila breviligulata  Agrostis stolonifera  Hierochloe odorata  Anthoxanthum odoratum  Phalaris arundinacea  Torreyochloa  Avena sativa  Trisetum cernuum  Aegilops cylindrica  Aegilops geniculata  Aegilops speltoides  Aegilops tauschii  Triticum aestivum  Triticum monococcum aegilopoides Triticum monococcum (modified) Triticum urartu (modified)  Hordeum jubatum  Hordeum vulgare  Hordeum vulgare spontaneum Secale cereale  Bromus vulgaris  Brachypodium distachyon  Diarrhena obovata  Melica mutica  Melica subulata  Oryzopsis asperifolia Achnatherum hymenoides Ampelodesmos mauritanica Piptochaetium avenaceum  Phaenosperma globosum Brachyelytrum aristosum  Bambusa bambosa | 77613  IGS |  | TTTCC-----AAATTGTTA  TTTCC-----AAATTGTTA  TTTCC-----AAATTGTTA  TTTCC-----AAATTGTTA  TTTCC-----AAATTGTTA  TTTCC-----AAATTGTTA  TTTCC-----AAATTGTTA  TTTCC-----AAATTGTTG  TTTCC-----AAATTGTTG  TTTCC-----AAATTGTTG  TTTCC-----AAATTATTG  TTTCT-----AAATTGTTG  TTTCA-----AAATTGTTG  TTTCC-----AAATTGTTG  TTTCC-----AAATTGTTG  TTTCC-----AAATTGTTG  TTTCC-----AAATTGTTG  TTTCCAAATTAAATTGTTG  TTTCC-----AAATTGTTG  TTTCC-----AAATTGTTG  TTTCA-----AAATTGTTA  TTTCC-----AAATTGTTG  TTTCCAAATTAAATTGTTG  TTTCCAAATTAAATTGTTG  TTTCCAAATTAAATTGTTG  TTTCCAAATTAAATTGTTG  TTTCCAAATTAAATTGTTG  TTTCCAAATTAAATTGTTG  TTTCCAAATTAAATTGTTG  TTTCCAAATTAAATTGTTG  TTTCCAAATTAAATTGTTG  TTTCCAAATTAAATTGTTG  TTTCCAAATTAAATTGTTG  TTTCCAAATTAAATTGTTG  TTTCCAAATTCAATTGTTG  TTTCC-----AAATTGTTG  TTTCC-----AAATTGTTG  TTTTC-----AAATTGTTG  TTTTC-----AAATTGTTG  TTTCC-----AAATTGTTG  TTTCC-----AAATTGTTG  TTTCC-----AAATTGTTG  TTTCC-----AAATTGTTG  TTTCC-----AAATTGTTG  TTTAC-----AAATAATTG  TTTCC-----AAATTGTTG | ssmp | Homoplasy |
| 119 | Schedonorus arundinaceus Schedonorus arundinaceus[C] Schedonorus pratensis  Festuca altissima  Festuca ovina  Lolium multiflorum  Lolium perenne  Dactylis glomerata  Helictochloa hookeri  Deschampsia antarctica  Poa palustris  Phleum alpinum  Briza sp  Puccinellia nuttalliana  Ammophila breviligulata  Agrostis stolonifera  Hierochloe odorata  Anthoxanthum odoratum  Phalaris arundinacea  Torreyochloa  Avena sativa  Trisetum cernuum  Aegilops cylindrica  Aegilops geniculata  Aegilops speltoides  Aegilops tauschii  Triticum aestivum  Triticum monococcum aegilopoides Triticum monococcum (modified) Triticum urartu (modified)  Hordeum jubatum  Hordeum vulgare  Hordeum vulgare spontaneum Secale cereale  Bromus vulgaris  Brachypodium distachyon  Diarrhena obovata  Melica mutica  Melica subulata  Oryzopsis asperifolia Achnatherum hymenoides Ampelodesmos mauritanica Piptochaetium avenaceum  Phaenosperma globosum Brachyelytrum aristosum  Bambusa bambosa | 78051  IGS |  | AACAATCTTGGGAAAGAGTTTCTGATC  AACAATCTTGGGAAAGAGTTTCTGATC  AACAATCTTGGGCAAGAGTTTCTGATC  AACAATCTTGGGCAAGAGTTTCTGATC  AACAATCTTGGGCAAGAGTTTCTGATC  AACAATCTCGGGCAAGAGTTTCTGATC  AACAATCTCGGGCAAGAGTTTCTGATC  AATAATCTTGGGCAAGAGTTTCTGATC  AATCATATTGGGCAAGAGTTTCTGATC  AACAATCTTGGGCAAGAGTTTCTGATC  AACAATCTTGGGCAAGAGTTTCTGATC  AACAATCTTGGGCAAGAGTTTCTGATC  AACAATCTTGGGCAAGAGTTTCTGATC  AACAATCTTGGGCAAGAGTTTCTGATC  AACAATCTTGGGCAAGAGTTTCTGATC  AACAATCTTGGGCAAGAGTTTCTGATC  AACAATCTTGGGCAAGAGTTTCTGATC  AACAATCTTGGGCAAGAGTTTCTGATC  AACAATCTTGGGCAAGAGTTTCTGATC  AACAATCTTGGGCAAGAGTTTCTGATC  AACAATCTTGGGCAAAAGTTTCTGATC  AACAATCTTGGGCAAGAGTTTCTGATC  AACAATCTTGGGCAAGAGTTTATGATC  AACAATCTTGGGCAAGAGTTTATGATC  AACAATCTTGGGCAAGAGTTTATGATC  AACAATCTTGGGCAAGAGTTTATGATC  AACAATCTTGGGCAAGAGTTTATGATC  AACAATCTTGGGCAAGAGTTTATGATC  AACAATCTTGGGCAAGAGTTTATGATC  AACAATCTTGGGCAAGAGTTTATGATC  AACAATCTTGGGCAAGAGTTTATGATC  A---------------AGTTTATGATC  A---------------AGTTTATGATC  AACAATCTTGGGCAAGAGTTTATGATC  AACAATCTTGGGCAAGAGTTTCTGATC  AACGTTCTTGGGCAAGAGTTTCTGATC  AACGATCTTGGGCAAGAGTTTCTGATC  AACGATCTTGGGCAAGAGTATCTGATC  AACGATCTTGGGCAAGAGTATCTGATC  AACGATCTTGGGCAAGAGTATCTGATC  AACGATCTTGGGCAAGAGTATCTGATC  AACGATCTTGGGCAAGAGTATCTGATC  AACGATCTTGGGCAAGAGTATCTGATC  AAGGATCTTGGGCAAGAGTATCTGATC  AACGGTATTGGGCAAGAGTATCTGATC  AAAGATCTTGGGCAAGAGTATCTGATC |  | Hordeum vulgare |
| 120 | Schedonorus arundinaceus Schedonorus arundinaceus[C] Schedonorus pratensis  Festuca altissima  Festuca ovina  Lolium multiflorum  Lolium perenne  Dactylis glomerata  Helictochloa hookeri  Deschampsia antarctica  Poa palustris  Phleum alpinum  Briza sp  Puccinellia nuttalliana  Ammophila breviligulata  Agrostis stolonifera  Hierochloe odorata  Anthoxanthum odoratum  Phalaris arundinacea  Torreyochloa  Avena sativa  Trisetum cernuum  Aegilops cylindrica  Aegilops geniculata  Aegilops speltoides  Aegilops tauschii  Triticum aestivum  Triticum monococcum aegilopoides Triticum monococcum (modified) Triticum urartu (modified)  Hordeum jubatum  Hordeum vulgare  Hordeum vulgare spontaneum Secale cereale  Bromus vulgaris  Brachypodium distachyon  Diarrhena obovata  Melica mutica  Melica subulata  Oryzopsis asperifolia Achnatherum hymenoides Ampelodesmos mauritanica Piptochaetium avenaceum  Phaenosperma globosum Brachyelytrum aristosum  Bambusa bambosa | 78469  IGS |  | TCAATTTAAATAAAGGGG  TCAATTTAAATAAAGGGG  TCAATTTAAATAAAGGGG  TCAATTTAAATAAAGTGG  TCAATTTAAATAAAGTGG  TCAATTTAAATAAAGGGG  TCAATTTAAATAAAGGGG  TCAATT----TAAAGTGG  TCAATT----TAAAGTGG  TCAATT----TAAAGTGG  TCAATT----TAAAGTGG  TAAATT----TAAAGTGG  TCAATT----TAAAGCGG  --AATT----TAAAGTGG  TCAATT----TAAAGCGG  GCAATT----TAAAGCGG  TCAATT----TAAAGCGG  TCAATT----TAAAGCGG  TCAATT----TAAAGCGG  TCAATT----TAAAGCGG  TCAATT----TAAAGTGG  TCAATT----TAAAGTGG  TCAATT----TAAAGGGG  TCAATT----TAAAGGGG  TCAATT----TAAAGGGG  TCAATT----TAAAGGGG  TCAATT----TAAAGGGG  TCAATT----TAAAGGGG  TCAATT----TAAAGGGG  TCAATT----TAAAGGGG  TCAAAT----TAAAGGGG  TCAATT----TAAAGGGG  TCAATT----TAAAGGGG  TCAATT----TAAAGGGG  TCAATT----TAAAGGGG  TCAATT----AAAAGCGA  TCAATT----AAAAGCGG  TCAATT----AAAAGAGG  TCAATT----AAAAGAGG  TCAATT----AAAAGCGG  TCGATT----AAAAGCCG  TCAATT----AAAAGCGG  TCAATT----AAAAGCGG  TCAATT----AAAAGCGG  TCAATT----AAAAACGG  TCAATT----AAAAGCGG | ssmp | Loliinae |
| 121 | Schedonorus arundinaceus Schedonorus arundinaceus[C] Schedonorus pratensis  Festuca altissima  Festuca ovina  Lolium multiflorum  Lolium perenne  Dactylis glomerata  Helictochloa hookeri  Deschampsia antarctica  Poa palustris  Phleum alpinum  Briza sp  Puccinellia nuttalliana  Ammophila breviligulata  Agrostis stolonifera  Hierochloe odorata  Anthoxanthum odoratum  Phalaris arundinacea  Torreyochloa  Avena sativa  Trisetum cernuum  Aegilops cylindrica  Aegilops geniculata  Aegilops speltoides  Aegilops tauschii  Triticum aestivum  Triticum monococcum aegilopoides Triticum monococcum (modified) Triticum urartu (modified)  Hordeum jubatum  Hordeum vulgare  Hordeum vulgare spontaneum Secale cereale  Bromus vulgaris  Brachypodium distachyon  Diarrhena obovata  Melica mutica  Melica subulata  Oryzopsis asperifolia Achnatherum hymenoides Ampelodesmos mauritanica Piptochaetium avenaceum  Phaenosperma globosum Brachyelytrum aristosum  Bambusa bambosa | 79083  IGS |  | CAACATA----ATGTGTT  CAACATA----ATGTGTT  CAACATC----ATGTGTT  GAACATC----GTGTGTT  GAACATC----GTGTGTT  CAACATC----ATGTGTT  CAACATC----ATGTGTT  GAACATC----GTGTGTT  GAACATC----GTATGTT  GAACATC----GTGTGTT  GAACATC----GTGTGTT  GAACATC----GTGTGTT  GAACATCCATCGTATGTT  GAACATC----ATGTATT  GAACATCCATCGTGTGTT  GAACATCCATCGTGTGTT  GAACATCCATTGTGTGTT  GAACATCCACCGTGTGTT  GAACATCCATCGTGTGTT  GAACATCCATCGTGTGTT  GAACACCCATCGTGTGTT  GAACATCCATCGTGTGTT  GAACATC----GTGTGTT  GAACATC----GTGTGTT  GAACATC----GTGTGTT  GAACATC----GTGTGTT  GAACATC----GTGTGTT  GAACATC----GTGTGTT  GAACATC----GTGTGTT  GAACATC----GTGTGTT  GAACATC----GTGTATT  GAACATC----GTGTATT  GAACATC----GTGTATT  GAACATC----GTGTGTT  GAACATC----ATGTGTT  GAAGATC----GTGTGTT  GAACATC----GTGTGTT  GAGCATC----GTGTGTT  GAGCATC----GTGTGTT  GAGCATC----GTGTGTT  GAGCATC----GTGTGTT  GAGCATC----GTGTGTT  GAGCATC----GTGTGTT  GAGCCTC----GTGTGTT  GAGCATC----GTGTGTT  GAGCATC----GTGTGTT | Ssmp | Poeae clade 1 |
| 122 | Schedonorus arundinaceus  Schedonorus arundinaceus[C]  Schedonorus pratensis  Festuca altissima  Festuca ovina  Lolium multiflorum  Lolium perenne  Dactylis glomerata  Helictochloa hookeri  Deschampsia antarctica  Poa palustris  Phleum alpinum  Briza sp  Puccinellia nuttalliana  Ammophila breviligulata  Agrostis stolonifera  Hierochloe odorata  Anthoxanthum odoratum  Phalaris arundinacea  Torreyochloa  Avena sativa  Trisetum cernuum  Aegilops cylindrica  Aegilops geniculata  Aegilops speltoides  Aegilops tauschii  Triticum aestivum  Triticum monococcum aegilopoides  Triticum monococcum (modified)  Triticum urartu (modified)  Hordeum jubatum  Hordeum vulgare  Hordeum vulgare spontaneum  Secale cereale  Bromus vulgaris  Brachypodium distachyon  Diarrhena obovata  Melica mutica  Melica subulata  Oryzopsis asperifolia  Achnatherum hymenoides  Ampelodesmos mauritanica  Piptochaetium avenaceum  Phaenosperma globosum  Brachyelytrum aristosum  Bambusa bambosa | 79292  IGS |  | AATATGAATTAAATAAGATTATGAATTAAA-----------------------------TAAGA  AATATGAATTAAATAAGATTATGAATTAAA-----------------------------TAAGA  AATATGAATTAAATAAGATTATGAATTAAA-----------------------------TAAGA  AA-----------------TATGAATTAAA-----------------------------TAAGA  TA-----------------TATGAATTAAC-----------------------------TAAGA  AATATGAATTAAATAAGATTATGAATTAAA-----------------------------TAAGA  AATATGAATTAAATAAGATTATGAATTAAA-----------------------------TAAGA  AA-----------------TATGAATTAAA-----------------------------TAAGA  AA-----------------TATGAATTAAA-----------------------------TAAGA  AA-----------------TATGAATTAAA-----------------------------TAAGA  AA-----------------TATGAATTAAA-----------------------------TAAGA  AA-----------------TATGAATTAAA-----------------------------TAAGA  AA-----------------GATGAATTAAA-----------------------------TAAGA  AA-----------------TATGAATTAAA-----------------------------TAAGA  AA-----------------GATGAATTAAA-----------------------------TAAGA  AA-----------------GATGAATTAAA-----------------------------TAAGA  AA-----------------GATGAATTAAA-----------------------------TAAGA  AA-----------------GATGAATTAAA-----------------------------TAAGA  AA-----------------GATGAATAAAA-----------------------------TAAGA  AA-----------------GATGAATTAAA-----------------------------TAAGA  AA-----------------GATTAATTAAA-----------------------------TAAGA  AA-----------------GATTAATTAAA-----------------------------TAAGA  AA-----------------TATGAATCAAA-----------------------------TAAGA  AA-----------------TATGAATCAAA-----------------------------TAAGA  AA-----------------TATGAATCAAA-----------------------------TAAGA  AA-----------------TATGAATCAAA-----------------------------TAAGA  AA-----------------TATGAATCAAA-----------------------------TAAGA  AA-----------------TATGAATCAAA-----------------------------TAAGA  AA-----------------TATGAATCAAA-----------------------------TAAGA  AA-----------------TATGAATCAAA-----------------------------TAAGA  TA-----------------TATGAATCAAA-----------------------------TAAGA  AA-----------------TATGAATCAAA-----------------------------TAAGA  AA-----------------TATGAATCAAA-----------------------------TAAGA  AA-----------------TATGAATCAAA-----------------------------TAAGA  AA-----------------TATGAATCAAA-----------------------------TAAGA  AA-----------------TATGAATCAAATAAGATTAAGAATATGAATCAAATAAGATTAAGA  AA-----------------TATGAATCAAA-----------------------------TAAGA  AA-----------------TATGAATCAAA-----------------------------TAAGA  AA-----------------TATGAATCAAA--------------------------ATTTAAGA  AA-----------------TATGAATCAAA-----------------------------TAAGA  AA-----------------TATGAATCAAA-----------------------------TAAGA  AA-----------------TATGAATCAAA-----------------------------TAAGA  AA-----------------TATGAATCAAA-----------------------------TAAGA  AA-----------------TATGAATCAAA-----------------------------TAAGA  TA-----------------TATGAATCAAA-----------------------------TAAGA  -A-----------------TATGAATCAAA-----------------------------TAATA | Ssmp | Lolium+Schedonorus |
| 123 | Schedonorus arundinaceus Schedonorus arundinaceus[C] Schedonorus pratensis  Festuca altissima  Festuca ovina  Lolium multiflorum  Lolium perenne  Dactylis glomerata  Helictochloa hookeri  Deschampsia antarctica  Poa palustris  Phleum alpinum  Briza sp  Puccinellia nuttalliana  Ammophila breviligulata  Agrostis stolonifera  Hierochloe odorata  Anthoxanthum odoratum  Phalaris arundinacea  Torreyochloa  Avena sativa  Trisetum cernuum  Aegilops cylindrica  Aegilops geniculata  Aegilops speltoides  Aegilops tauschii  Triticum aestivum  Triticum monococcum aegilopoides Triticum monococcum (modified) Triticum urartu (modified)  Hordeum jubatum  Hordeum vulgare  Hordeum vulgare spontaneum Secale cereale  Bromus vulgaris  Brachypodium distachyon  Diarrhena obovata  Melica mutica  Melica subulata  Oryzopsis asperifolia Achnatherum hymenoides Ampelodesmos mauritanica Piptochaetium avenaceum  Phaenosperma globosum Brachyelytrum aristosum  Bambusa bambosa | 79939  Rps18 | Rps18 | CTTAAG---------------------AAAC-----AACAA  CTTAAG---------------------AAAC-----AACAA  CTTAAG---------------------AAAC-----AACAA  CTTAAG---------------------AAAC-----AACAA  CTTAAGAAACTACAACCAAAATGTAAGAAAC-----AACAA  CTTAAG---------------------AAAC-----AACAA  CTTAAG---------------------AAAC-----AACAA  CTTAAGAAACTACAACCAAAATTTAAGAAAC-----AACAA  CTTAAGAAACTACAACCAAAATTTAAGAAACAACAAAACAA  CTTAAGAAACTACAACCAAAATTTAAGAAAC-----AACAA  CTTAAGAAACTACAACCAAAATTTAAGAAAC-----AACAA  CTTAAGAAACTACAACCAAAATTTAAGAAAC-----AACAA  CTTAAGAAACTACAAGCAAAATTTAAGAAAC-----AACAA  CTTAAGAAACTACAACCAAAATTTAAGAAAC-----AACAA  CTTAAGAAACTACAACCAAAATTTAAGAAAC-----AACAA  CTTAAGAAACTACAACCAAAATTTAAGAAAC-----ATCAA  CTTAAGAAACTACAACCAAAATTTAAGAAAC-----AACAA  CTTAAGAAACTACAACCAAAATTTAAGAAAC-----AACAA  CTTAAGAAACTACAACCAAAATTTAAGAAAC-----AACAA  CTTAAGAAACTACAACCAAAATTTAAGAAAC-----AACAA  CTTAAGAAACTACAACCAAAATTTAAGAAAC-----AACAA  CTTAAGAAACTACAACCAAAATTTAAGAAAC-----AACAA  CTTAAGAAACAACAACCAAAATTTAAGAAAC-----AACAA  CTTAAGAAACAACAACCAAAATTTAAGAAAC-----AACAA  CTTAAGAAACAACAACCAAAATTTAAGAAAC-----AACAA  CTTAAGAAACAACAACCAAAATTTAAGAAAC-----AACAA  CTTAAGAAACAACAACCAAAATTTAAGAAAC-----AACAA  CTTAAGAAACAACAACCAAAATTTAAGAAAC-----AACAA  CTTAAGAAACAACAACCAAAATTTAAGAAAC-----AACAA  CTTAAGAAACAACAACCAAAATTTAAGAAAC-----AACAA  CTTAAGAAACTACAACCAAAATTTAAGAAAC-----AACAA  CTTAAGAAACTACAACCAAAATTTAAGAAAC-----AACAA  CTTAAGAAACTACAACCAAAATTTAAGAAAC-----AACAA  CTTAAGAAACAACAACCAAAATTTAAGAAAC-----AACAA  CTTAAGAAACTACAACCAAAATTTAAGAAAC-----AACAA  CGTAAGAAACTATAACCAAAATTTAAGAAAC-----AACAA  CTTAAGAAACTACAACCAAAATTTAAGAAAC-----AACAA  CTTAAGAAACTACAACCAGAATTTAAGAAAC-----AACAA  CTTAAGAAATTACAACCAGAATTTAAGAAAC-----AACAA  CTTAAGAAACTACAACCAGAATTTAAGAAAC-----AAAAA  CTTAAGAAACTACAACCAGAATTTAAGAAAC-----AACAA  CTTAAGAAACTACAACCAGAATTTAAGAAAC-----AACAA  CTTAAGAAACTACAACCAGAATTTAAGAAAC-----AACAA  CTTAAGAAACTACAACCAGAATTTAAGAAAC-----AACAA  CTTAAGAAACTACAACCAGAATTTTAGAAAC-----AACAA  CTTAAGAAACTCCAACCAGAATTTAAGAAAC-----AACAA |  | Lolium+Schedonorus+Festuca altissima |
| 124 | Schedonorus arundinaceus Schedonorus arundinaceus[C] Schedonorus pratensis  Festuca altissima  Festuca ovina  Lolium multiflorum  Lolium perenne  Dactylis glomerata  Helictochloa hookeri  Deschampsia antarctica  Poa palustris  Phleum alpinum  Briza sp  Puccinellia nuttalliana  Ammophila breviligulata  Agrostis stolonifera  Hierochloe odorata  Anthoxanthum odoratum  Phalaris arundinacea  Torreyochloa  Avena sativa  Trisetum cernuum  Aegilops cylindrica  Aegilops geniculata  Aegilops speltoides  Aegilops tauschii  Triticum aestivum  Triticum monococcum aegilopoides Triticum monococcum (modified) Triticum urartu (modified)  Hordeum jubatum  Hordeum vulgare  Hordeum vulgare spontaneum Secale cereale  Bromus vulgaris  Brachypodium distachyon  Diarrhena obovata  Melica mutica  Melica subulata  Oryzopsis asperifolia Achnatherum hymenoides Ampelodesmos mauritanica Piptochaetium avenaceum  Phaenosperma globosum Brachyelytrum aristosum  Bambusa bambosa | 79993  Rps18 | Rps18 | TCCGATTGTTGA  TCCGATTGTTGA  TCCGATTGTTGA  TCCGATTGTTGA  TCCGATTGTTGA  TCCGATTGTTGA  TCCGATTGTTGA  TCCGATTGTTGA  TCCAGTTGTTGA  TCCGATTGTTGA  TCCGATTGTTGA  TCCGATTGTTGA  TCCGATTGTTGA  TCCGATTGTTGA  TCCGA---TTGA  TCCGA---TTGA  TCCGATTGTTGA  TCCGATTGTTGA  TCCGATTGTTGA  TCCGATTGTTGA  TCCGATTGTTGA  TCCGATTGTTGA  TCCGATTGTTGA  TCCGATTGTTGA  TCCGATTGTTGA  TCCGATTGTTGA  TCCGATTGTTGA  TCCGATTGTTGA  TCCGATTGTTGA  TCCGATTGTTGA  TCCGATTGTTGA  TCCGATTGTTGA  TCCGATTGTTGA  TCCGATTGTTGA  TCCGATTGTTGA  TCCGATTGTTGA  TCCGATTGTTGA  TCCGATTGTTGA  TCCGATTGTTGA  TCCGATTGTTGA  TCCGATTGTTGA  TCCGATTGTTGA  TCCGATTGTTGA  TCCGATTGTTGA  TCCGATTGTTGA  TCCGATTGTTGA | ssmp | Agrostidinae |
| 125 | Schedonorus arundinaceus Schedonorus arundinaceus[C] Schedonorus pratensis  Festuca altissima  Festuca ovina  Lolium multiflorum  Lolium perenne  Dactylis glomerata  Helictochloa hookeri  Deschampsia antarctica  Poa palustris  Phleum alpinum  Briza sp  Puccinellia nuttalliana  Ammophila breviligulata  Agrostis stolonifera  Hierochloe odorata  Anthoxanthum odoratum  Phalaris arundinacea  Torreyochloa  Avena sativa  Trisetum cernuum  Aegilops cylindrica  Aegilops geniculata  Aegilops speltoides  Aegilops tauschii  Triticum aestivum  Triticum monococcum aegilopoides Triticum monococcum (modified) Triticum urartu (modified)  Hordeum jubatum  Hordeum vulgare  Hordeum vulgare spontaneum Secale cereale  Bromus vulgaris  Brachypodium distachyon  Diarrhena obovata  Melica mutica  Melica subulata  Oryzopsis asperifolia Achnatherum hymenoides Ampelodesmos mauritanica Piptochaetium avenaceum  Phaenosperma globosum Brachyelytrum aristosum  Bambusa bambosa | 80010  IGS |  | TTATTCGA------AAGGG  TTATTCGA------AAGGG  TTATTCGA------AAGGG  TTATTCGA------AAGGG  TTATTCGA------AAGGG  TTATTCGA------AAGGG  TTATTCGA------AAGGG  TTATTCGA------AAAGG  TTATTCGA------AAGGG  TTATTCGA------AAGGA  TTATTCGA------AAGGG  TTATTCGA------AAAGG  TTATTCGA------AAGGG  TTATTCGA------AAGGG  TTATTCGA------AAGGG  TTATTCGA------AAGGG  TTATTCGA------AAGGG  TTATTCGA------AAGGG  TTATTCGA------AAGGG  TTATTCGA------AAGGG  TTATTCGA------AAGGC  TTATTCGA------AAGGG  TTATTCGAATTCGAAAGGG  TTATTCGAATTCGAAAGGG  TTATTCGAATTCGAAAGGG  TTATTCGAATTCGAAAGGG  TTATTCGAATTCGAAAGGG  TTATTCGAATTCGAAAGGG  TTATTCGAATTCGAAAGGG  TTATTCGAATTCGAAAGGG  TTATTCGAATTCGCAAGGG  TTATTCGAATTCGCAAGGG  TTATTCGAATTCGCAAGGG  TTATTCGAATTCGAAAGGG  TTATTCGAATTCGAAAGGG  TTATTCGA------AAGGG  TTATTTGA------AAGGG  TTATTTGA------AAGGG  TTATTTGA------AAGGG  TTATTCGA------AAGGG  TTATTCGA------AAGGG  TTATTCGA------AAGGG  TTATTCGA------AAGGG  TTATTCGA------AAGGG  TTATTCGA------AAGGA  TTATTCGA------AAGGG | Ssmp | Bromeae+Triticeae |
| 126 | Schedonorus arundinaceus Schedonorus arundinaceus[C] Schedonorus pratensis  Festuca altissima  Festuca ovina  Lolium multiflorum  Lolium perenne  Dactylis glomerata  Helictochloa hookeri  Deschampsia antarctica  Poa palustris  Phleum alpinum  Briza sp  Puccinellia nuttalliana  Ammophila breviligulata  Agrostis stolonifera  Hierochloe odorata  Anthoxanthum odoratum  Phalaris arundinacea  Torreyochloa  Avena sativa  Trisetum cernuum  Aegilops cylindrica  Aegilops geniculata  Aegilops speltoides  Aegilops tauschii  Triticum aestivum  Triticum monococcum aegilopoides Triticum monococcum (modified) Triticum urartu (modified)  Hordeum jubatum  Hordeum vulgare  Hordeum vulgare spontaneum Secale cereale  Bromus vulgaris  Brachypodium distachyon  Diarrhena obovata  Melica mutica  Melica subulata  Oryzopsis asperifolia Achnatherum hymenoides Ampelodesmos mauritanica Piptochaetium avenaceum  Phaenosperma globosum Brachyelytrum aristosum  Bambusa bambosa | 80024  IGS |  | AAGGGCCGGACCAGACC  AAGGGCCGGACCAGACC  AAGGGCCGGACCAGACC  AAGGGCCAGACCAGACC  AAGGG-----CCAGACC  AAGGGCCGGACCAGACC  AAGGGCCGGACCAGACC  AAAGG-----CCAGACC  AAGGG-----CCAGACC  AAGGA-----CCAGACT  AAGGG-----CCAGACC  AAAGG-----CCAGACC  AAGGG-----CCAGACC  AAGGG-----CCAGACC  AAGGG-----CCAGACC  AAGGG-----CCAGACC  AAGGG-----CCAGACC  AAGGG-----CCAGACC  AAGGG-----CCAGACC  AAGGG-----CCAGACC  AAGGC-----CCAGACC  AAGGG-----CCAGACC  AAGGG-----CCAGACC  AAGGG-----CCAGACC  AAGGG-----CCAGACC  AAGGG-----CCAGACC  AAGGG-----CCAGACC  AAGGG-----CCAGACC  AAGGG-----CCAGACC  AAGGG-----CCAGACC  AAGGG-----CCAGACC  AAGGG-----CCAGACC  AAGGG-----CCAGACC  AAGGG-----CCAGACC  AAGGG-----CCAGACC  AAGGG-----CCAGGCC  AAGGG-----CCAGACC  AAGGG-----CCAGACC  AAGGG-----CCAGACC  AAGGG-----CCAGACC  AAGGG-----CCAGACC  AAGGG-----CCAGACC  AAGGG-----CCAGACC  AAGGG-----CCAGACC  AAGGA-----CCAGATC  AAGGG-----CCAGACC |  | Lolium+Schedonorus+Festuca altissima |
| 127 | Schedonorus arundinaceus Schedonorus arundinaceus[C] Schedonorus pratensis  Festuca altissima  Festuca ovina  Lolium multiflorum  Lolium perenne  Dactylis glomerata  Helictochloa hookeri  Deschampsia antarctica  Poa palustris  Phleum alpinum  Briza sp  Puccinellia nuttalliana  Ammophila breviligulata  Agrostis stolonifera  Hierochloe odorata  Anthoxanthum odoratum  Phalaris arundinacea  Torreyochloa  Avena sativa  Trisetum cernuum  Aegilops cylindrica  Aegilops geniculata  Aegilops speltoides  Aegilops tauschii  Triticum aestivum  Triticum monococcum aegilopoides Triticum monococcum (modified) Triticum urartu (modified)  Hordeum jubatum  Hordeum vulgare  Hordeum vulgare spontaneum Secale cereale  Bromus vulgaris  Brachypodium distachyon  Diarrhena obovata  Melica mutica  Melica subulata  Oryzopsis asperifolia Achnatherum hymenoides Ampelodesmos mauritanica Piptochaetium avenaceum  Phaenosperma globosum Brachyelytrum aristosum  Bambusa bambosa | 80043  IGS |  | TATATA-------------------------------TATTAT  TATATA-------------------------------TATTAT  TATATA-------------------------------TATTAT  TATATA-------------------------------TATTAT  TATATA-------------------------------TATTAT  TATATA-------------------------------TATTAT  TATATA-------------------------------TATTAT  TATATA-------------------------------TATTAT  TATATA-------------------------------TATTAT  TATATA-------------------------------TATTAT  TATATA-------------------------------TATTAT  TATATA-------------------------------TATTAT  TATATA-------------------------------TATTAT  TATATA-------------------------------TATTAT  TATATA-------------------------------TATTAT  TATATA-------------------------------TATTAT  --TATA-------------------------------TATTAT  --CATA-------------------------------TATTAT  TATATA-------------------------------TATTAT  TATATA-------------------------------TATTAT  TATATA-------------------------------TATTAT  TATATA-------------------------------TATTAT  TATATA-------------------------------TATTAT  TATATA-------------------------------TATTAT  TATATA-------------------------------TATTAT  TATATA-------------------------------TATTAT  TATATA-------------------------------TATTAT  TATATA-------------------------------TATTAT  TATATA-------------------------------TATTAT  TATATA-------------------------------TATTAT  TATATA-------------------------------TATTAT  TATATA-------------------------------TATTAT  TATATA-------------------------------TATTAT  TATATA-------------------------------TATTAT  TATATA-------------------------------TATTAT  TATATA-------------------------------TATTAT  TATATA-------------------------------TATTAT  TATATA-------------------------------TATTAT  TATATA-------------------------------TATTAT  TATATA-------------------------------TATTAT  TATATA-------------------------------TATTAT  TATATA-------------------------------TATTAT  TATATA-------------------------------TATTAT  TATATA-------------------------------T-TATT  TATATAAAGAAAGTAATCCAGTTTTGATTCTTGTGTTTGTTAT  TATATAAGGAAAGTAATCCAGTTTTGATTCTTGTGTTTGTTAT |  | Homoplasy |
| 128 | Schedonorus arundinaceus Schedonorus arundinaceus[C] Schedonorus pratensis  Festuca altissima  Festuca ovina  Lolium multiflorum  Lolium perenne  Dactylis glomerata  Helictochloa hookeri  Deschampsia antarctica  Poa palustris  Phleum alpinum  Briza sp  Puccinellia nuttalliana  Ammophila breviligulata  Agrostis stolonifera  Hierochloe odorata  Anthoxanthum odoratum  Phalaris arundinacea  Torreyochloa  Avena sativa  Trisetum cernuum  Aegilops cylindrica  Aegilops geniculata  Aegilops speltoides  Aegilops tauschii  Triticum aestivum  Triticum monococcum aegilopoides Triticum monococcum (modified) Triticum urartu (modified)  Hordeum jubatum  Hordeum vulgare  Hordeum vulgare spontaneum Secale cereale  Bromus vulgaris  Brachypodium distachyon  Diarrhena obovata  Melica mutica  Melica subulata  Oryzopsis asperifolia Achnatherum hymenoides Ampelodesmos mauritanica Piptochaetium avenaceum  Phaenosperma globosum Brachyelytrum aristosum  Bambusa bambosa | 80089  IGS |  | GAAAGTA-----------------------------------ATCCAG  GAAAGTA-----------------------------------ATCCAG  GAAAGTA-----------------------------------ATCCAG  GAAAGTA-----------------------------------ATCCAG  GAAAGTA-----------------------------------ATCCAG  GAAAGTA-----------------------------------ATCCAG  GAAAGTA-----------------------------------ATCCAG  GAAAGTA-----------------------------------ATCCAG  GAAAGTA-----------------------------------ATCCAG  GAAAGTA-----------------------------------ATCCAG  GAAAGTA-----------------------------------ATCCAG  GAAAGTA-----------------------------------ATCCAG  GAAAGTA-----------------------------------ATCCAG  GAAAGTA-----------------------------------ATCCAG  GAAAGTA-----------------------------------ATCCAG  GAAAGTA-----------------------------------ATCGAG  GAAAGTA-----------------------------------ATCCAG  GAAAGTA-----------------------------------ATCCAG  GAAAGTA-----------------------------------ATCCAG  GAAAGTA-----------------------------------ATCCAG  GAAAGTA-----------------------------------ATCCTG  GAAAGTA-----------------------------------ATCCTG  GAAAGTA-----------------------------------ATCCAG  GAAAGTA-----------------------------------ATCCAG  GAAAGTA-----------------------------------ATCCAG  GAAAGTA-----------------------------------ATCCAG  GAAAGTA-----------------------------------ATCCAG  GAAAGTA-----------------------------------ATCCAG  GAAAGTA-----------------------------------ATCCAG  GAAAGTA-----------------------------------ATCCAG  GAAAGTA-----------------------------------ATCCAG  GAAAGTA-----------------------------------ATCCAG  GAAAGTA-----------------------------------ATCCAG  GAAAGTA-----------------------------------ATCCAG  GAAAGTA-----------------------------------ATCCAG  GAAAGTA-----------------------------------ATCCAG  GAAAGTA-----------------------------------ATCCAG  GAAAGTA-----------------------------------ATCCAG  GAAAGTA-----------------------------------ATCCAG  GAAAGTA-----------------------------------ATCCAG  GAAAGTA-----------------------------------ATCCAG  GAAAGTA-----------------------------------ATCCAG  GAAAGTA-----------------------------------ATCCAG  GAAAGTA-----------------------------------ATCCAG  AAAGAAAAATGGGGAAGAAGAAATCGTTTTTTTATTTATTGCAACATG  AAAGAAAAATGGGGAAGAATAAATAGTTTTTTTATTTATTGCAACATG |  | Homoplasy |
| 129 | Schedonorus arundinaceus Schedonorus arundinaceus[C] Schedonorus pratensis  Festuca altissima  Festuca ovina  Lolium multiflorum  Lolium perenne  Dactylis glomerata  Helictochloa hookeri  Deschampsia antarctica  Poa palustris  Phleum alpinum  Briza sp  Puccinellia nuttalliana  Ammophila breviligulata  Agrostis stolonifera  Hierochloe odorata  Anthoxanthum odoratum  Phalaris arundinacea  Torreyochloa  Avena sativa  Trisetum cernuum  Aegilops cylindrica  Aegilops geniculata  Aegilops speltoides  Aegilops tauschii  Triticum aestivum  Triticum monococcum aegilopoides Triticum monococcum (modified) Triticum urartu (modified)  Hordeum jubatum  Hordeum vulgare  Hordeum vulgare spontaneum Secale cereale  Bromus vulgaris  Brachypodium distachyon  Diarrhena obovata  Melica mutica  Melica subulata  Oryzopsis asperifolia Achnatherum hymenoides Ampelodesmos mauritanica Piptochaetium avenaceum  Phaenosperma globosum Brachyelytrum aristosum  Bambusa bambosa | 80212  IGS |  | TATTCCAG-----TATAATA  TATTCCAG-----TATAATA  TATTCCAG-----TATAATA  TATTCCAG-----TATAATA  TATTCCAG-----TATAATA  TATTCCAG-----TATAATA  TATTCCAG-----TATAATA  TATTCCAG-----TATATTA  TATTCCAG-----TATATTA  TATTCCAG-----TATATTA  TATTCCAC-----TATATTA  TATTCCAG-----TATATTA  TATTCCAG-----TATATTA  TATTCCAG-----TATATTA  TATTCCAG-----TATATTA  TATTCCAG-----TATATTA  TATTCCAG-----TATATTA  TATTCCAGTATATTATATTA  TATTCCAGTATATTATATTA  TATTCCAGTATATTATATTA  TATTCCAG-----TATATTA  TATTCCAG-----TATATTA  TATTCCAG-----TATATTA  TATTCCAG-----TATATTA  TATTCCAG-----TATATTA  TATTCCAG-----TATATTA  TATTCCAG-----TATATTA  TATTCCAG-----TATATTA  TATTCCAG-----TATATTA  TATTCCAG-----TATATTA  TATTCCAG-----TATATTA  TATTCCAG-----TATATTA  TATTCCAG-----TATATTA  TATTCCAG-----TATATTA  TATCCCAG-----TATATTA  TATTCCAG-----TATATTA  TATTCCAG-----TATATTA  TATTCCAG-----TATATTA  TATTCCAG-----TATATTA  TATTCCAG-----TATATTA  TATTCCAG-----TATATTA  TATTCCAG-----TATATTA  TATTCCAG-----TATATTA  TATTCCAG-----TATATTA  TATTCCTG-----TATATTA  TATTCCTG-----TATATTA |  | Homoplasy |
| 130 | Schedonorus arundinaceus Schedonorus arundinaceus[C] Schedonorus pratensis  Festuca altissima  Festuca ovina  Lolium multiflorum  Lolium perenne  Dactylis glomerata  Helictochloa hookeri  Deschampsia antarctica  Poa palustris  Phleum alpinum  Briza sp  Puccinellia nuttalliana  Ammophila breviligulata  Agrostis stolonifera  Hierochloe odorata  Anthoxanthum odoratum  Phalaris arundinacea  Torreyochloa  Avena sativa  Trisetum cernuum  Aegilops cylindrica  Aegilops geniculata  Aegilops speltoides  Aegilops tauschii  Triticum aestivum  Triticum monococcum aegilopoides Triticum monococcum (modified) Triticum urartu (modified)  Hordeum jubatum  Hordeum vulgare  Hordeum vulgare spontaneum Secale cereale  Bromus vulgaris  Brachypodium distachyon  Diarrhena obovata  Melica mutica  Melica subulata  Oryzopsis asperifolia Achnatherum hymenoides Ampelodesmos mauritanica Piptochaetium avenaceum  Phaenosperma globosum Brachyelytrum aristosum  Bambusa bambosa | 81551  IGS |  | GAAGTC------TTTCTATTTTC  GAAGTC------TTTCTATTTTC  GAAGTC------TTTCTATTTTC  GAAGTCTTTCTATTTCGATTTTC  GAAGTCTTTCTATTTCTATTTTC  GAAGTC------TTTCTATTTTC  GAAGTC------TTTCTATTTTC  GAAGTCCTTCTATTTCTATTTTC  GAAGTCTTTCGATTTCTATTTTC  GAAGTCTTTCTATTTCTATTTTC  GAAGTCTTTCTATTTCTATTTTA  GAAGTCTTTCTATTTCTATTTTA  GAAGTCTTTCTATTTCTATTTTC  GAAGTCTTTCTATTTCTATTAAA  GAAGTCTTTCTATTTCTATTTTC  GAAGTCTTTCTATTTCTATTTTC  GAAGTC------TTTCTATTTTC  GAAGTC------TTTCTATTTTC  GAAGTGTTTCTATTTCTATTTTC  GAAGTCTTTCTATTTATATTTTC  GAAGTCTTTCTATTTCGATTTTC  GAAGTCTTTCTATTTCTATTTTC  GAAGTC------TTTCTATTTTC  GAAGTC------TTTCTATTTTC  GAAGTC------TTTCTATTTTC  GAAGTC------TTTCTATTTTC  GAAGTC------TTTCTATTTTC  GAAGTT------TTTCTATTTTC  GAAGTT------TTTCTATTTTC  GAAGTC------TTTCTATTTTC  GAAGTC------TTTCTATTTTC  GAAGTC------TTTCTATTTTC  GAAGTC------TTTCTATTTTC  GAAGTC------TTTCTATTTTC  GAAGTC------TTTCTATTTTC  GAAGTC------TTTCTATTTTC  GAAGTC------TTTCTATTTTC  GAAGTC------TTTCTATTTTC  GAAGTC------TTTCTATTTTC  GAAGTC------TTTCTATTTTC  GAAGTC------TTTCTATTTTC  GAAGTC------TTTCTATTTTC  GAAGTC------TTTCTATTTTC  GAAGTC------TTTCTATTTTC  GAAGTC------TTTCTATTTTC  GAAGTA------TTTCTATTTTC | ssmp | Homoplasy |
| 131 | Schedonorus arundinaceus Schedonorus arundinaceus[C] Schedonorus pratensis  Festuca altissima  Festuca ovina  Lolium multiflorum  Lolium perenne  Dactylis glomerata  Helictochloa hookeri  Deschampsia antarctica  Poa palustris  Phleum alpinum  Briza sp  Puccinellia nuttalliana  Ammophila breviligulata  Agrostis stolonifera  Hierochloe odorata  Anthoxanthum odoratum  Phalaris arundinacea  Torreyochloa  Avena sativa  Trisetum cernuum  Aegilops cylindrica  Aegilops geniculata  Aegilops speltoides  Aegilops tauschii  Triticum aestivum  Triticum monococcum aegilopoides Triticum monococcum (modified) Triticum urartu (modified)  Hordeum jubatum  Hordeum vulgare  Hordeum vulgare spontaneum Secale cereale  Bromus vulgaris  Brachypodium distachyon  Diarrhena obovata  Melica mutica  Melica subulata  Oryzopsis asperifolia Achnatherum hymenoides Ampelodesmos mauritanica Piptochaetium avenaceum  Phaenosperma globosum Brachyelytrum aristosum  Bambusa bambosa | 84507  IGS |  | ATGGGAAACAAAACAGCTCCCATC  ATGGGAAACAAAACAGCTCCCATC  ATGGGAAACAAAACAGCTCCCATC  ACGGGAAACAAAACAGCTCCCATC  ATGGGAAACAAAACAGCTCCCATC  ATGGGAAACAAAACAGCTCCCATC  ATGGGAAACAAAACAGCTCCCATC  ATGGGAAACAAAACAGCTCCCATC  ATGGGAAACAAAACAGCTCCCATC  ATGGGAAACAAAACAGCTCCCATC  ATGGGAAA-----CAGCTCCCATC  ATGGGAAA-----CAGCTCCCATC  ATGGGAAA-----CATCTCCCATC  ATGGGAAA-----CAGCTCCCATC  ATGGGAAA-----CATCTCCCATC  ATGGGAAA-----CATCTCCCATC  ATGGGAAA-----CATCTCCCATC  ATGGGAAA-----CATCTCCCATC  ATGGGAAA-----CATCTCCCATC  ATGGGAAA-----CATCTCCCATC  TTGGGAAA-----CATCTCCCATC  ATGGGAAA-----CATCTCCCATC  ATGGGAAA-----CATCTCCCATC  ATGGGAAA-----CATCTCCCATC  ATGGGAAA-----CATCTCCCATC  ATGGGAAA-----CATCTCCCATC  ATGGGAAA-----CATCTCCCATC  ATGGGAAA-----CATCTCCCATC  ATGGGAAA-----CATCTCCCATC  ATGGGAAA-----CATCTCCCATC  -------A-----CATCTCCCATC  -------A-----CATCTCCCATC  -------A-----CATCTCCCATC  ATGGGAAA-----CATCTCCCATC  ATGGGAAA-----CATCTCCCATC  ATGGGAAA-----CATCTCCCATC  ATGGGAAA-----CATCTCCCATC  ATGGGAAA-----CATCTCCCTAC  ATGGGAAA-----CATCTCCCTAC  ATGGGAAA-----CATCTCCCATC  ATGGGAAA-----CATCTCCCATC  ATGGGAAA-----CATCTCCCATC  ATGGGAAA-----CATCTCCCATC  ATGGGAAA-----CATCTCCCATC  ATGGGAAA-----CATCTCCCATC  ATGGGAAA-----CATCTCCCATC |  | Airinae+Dactylidinae+Holcinae+Loliinae |
| 132 | Schedonorus arundinaceus Schedonorus arundinaceus[C] Schedonorus pratensis  Festuca altissima  Festuca ovina  Lolium multiflorum  Lolium perenne  Dactylis glomerata  Helictochloa hookeri  Deschampsia antarctica  Poa palustris  Phleum alpinum  Briza sp  Puccinellia nuttalliana  Ammophila breviligulata  Agrostis stolonifera  Hierochloe odorata  Anthoxanthum odoratum  Phalaris arundinacea  Torreyochloa  Avena sativa  Trisetum cernuum  Aegilops cylindrica  Aegilops geniculata  Aegilops speltoides  Aegilops tauschii  Triticum aestivum  Triticum monococcum aegilopoides Triticum monococcum (modified) Triticum urartu (modified)  Hordeum jubatum  Hordeum vulgare  Hordeum vulgare spontaneum Secale cereale  Bromus vulgaris  Brachypodium distachyon  Diarrhena obovata  Melica mutica  Melica subulata  Oryzopsis asperifolia Achnatherum hymenoides Ampelodesmos mauritanica Piptochaetium avenaceum  Phaenosperma globosum Brachyelytrum aristosum  Bambusa bambosa | 84723  psbT | psbT | AACCACCTAAGGTTCCACCAACTCCAACTAAAAGAA  AACCACCTAAGGTTCCACCAACTCCAACTAAAAGAA  AACCACCTAAGGTTCCACCAACTCCAACTAAAAGAA  AACCACCTAAGGTTCCACCAACTCCAACTAAAAGAA  AACCACCTAAGGTTCCACCAACTCCAACTAAAAGAA  AACCACCTAAGGTTCCACCAACTCCAACTAAAAGAA  AACCACCTAAGGTTCCACCAACTCCAACTAAAAGAA  AACCACCTAAGGTTCCGCCAACTCCAACTAAAAGAA  AACCACCTAAGGTTCCACCAACTCCAACTAAAAGAA  AACCGCCTAAGGTTCCACCAACTCCAACTAAAAGAA  AACCACCTAAGGTTCCACCAACTCCAACTAAAAGAA  AACCGCCTAAGGTTCCACCAACTCCAACTAAAAGAA  AACCACCTAAGGTTCCACCAACTCCAACTAAAAGAA  AACCACCTAAGGTTCCACCAACTCCAACTAAAAGAA  AACCACCTAAGGTTCCACCAACTCCAACTAAAAGAA  AACCACCTAAGGTTCCACCAACTCCAACTAAAAGAA  AACCACCTAAGGTTCCACCAACTCCAACTAAAAGAA  AACCACCTAAGGTTCCACCAACTCCAACTAAAAGAA  AACCACCTAAGGTTCCACCAACTCCAACTAAAAGAA  AACCACCTAAGGTTCCACCAACTCCAACTAAAAGAA  AACCACCTAAGGTTCCACCAACTCCAACTAAAAGAA  AACCACCTAAGGTTCCACCAACCCCAACTAAAAGAA  AACCACCTAAGGTTCCACCAACTCCAACTAAAAGAA  AACCACCTAAGGTTCCACCAACTCCAACTAAAAGAA  AACCACCTAAGGTTCCACCAACTCCAACTAAAAGAA  AACCACCTAAGGTTCCACCAACTCCAACTAAAAGAA  AACCACCTAAGGTTCCACCAACTCCAACTAAAAGAA  AACCACCTAAGGTTCCACCAACTCCAACTAAAAGAA  AACCACCTAAGGTTCCACCAACTCCAACTAAAAGAA  AACCACCTAAGGTTCCACCAACTCCAACTAAAAGAA  AACCACCTAAGGTTCCACCAACTCCAACTAAAAGAA  AACCACCTAAGGTTCCACCAACTCCAACTAAAAGAA  AACCACCTAAGGTTCCACCAACTCCAACTAAAAGAA  AACCACCTAAGGTTCCACCAACTCCAACTAAAAGAA  AACCACCTAAGGTTCCACCAACTCCAACTAAAAGAA  AACCGCCTAAGGTT---------CCAACTAAAAGAG  AACCACCTAAGGTT---------CCAACTAAAAGAA  AACCACCTAAGGTT---------CCGACTAAAAGAA  AACCACCTAAGGTT---------CCGACTAAAAGAA  AACCGCCTAAGGTT---------CCGACTAAAAGAG  AACCGCCAAAGGTT---------CCGACTAAAAGAA  AACCGCCTAAGGTT---------CCGACTAAAAGAA  AACCGCCTAAGGTT---------CCGACTAAAAGAG  AACCTCCTAAGGTT---------CCGACTAAAAGAA  AACCACCTAAGGTT---------CCGACTAAAA-AA  AACCACCTAAGGTT---------CCGACTAAAAAAA |  | Bromeae+Poeae+Triticeae |
| 133 | Schedonorus arundinaceus Schedonorus arundinaceus[C] Schedonorus pratensis  Festuca altissima  Festuca ovina  Lolium multiflorum  Lolium perenne  Dactylis glomerata  Helictochloa hookeri  Deschampsia antarctica  Poa palustris  Phleum alpinum  Briza sp  Puccinellia nuttalliana  Ammophila breviligulata  Agrostis stolonifera  Hierochloe odorata  Anthoxanthum odoratum  Phalaris arundinacea  Torreyochloa  Avena sativa  Trisetum cernuum  Aegilops cylindrica  Aegilops geniculata  Aegilops speltoides  Aegilops tauschii  Triticum aestivum  Triticum monococcum aegilopoides Triticum monococcum (modified) Triticum urartu (modified)  Hordeum jubatum  Hordeum vulgare  Hordeum vulgare spontaneum Secale cereale  Bromus vulgaris  Brachypodium distachyon  Diarrhena obovata  Melica mutica  Melica subulata  Oryzopsis asperifolia Achnatherum hymenoides Ampelodesmos mauritanica Piptochaetium avenaceum  Phaenosperma globosum Brachyelytrum aristosum  Bambusa bambosa | 85797  petB intron | petB intron | CACTGA------TTCACTA  CACTGA------TTCACTA  CACTGA------TTCACTA  CACTGA------TTCACTA  CACTGA------TTCACTA  CACTGA------TTCACTA  CACTGA------TTCACTA  CACTGA------TTCACTA  CACTGA------TTCACTA  CACTGA------TTCACTA  CACTGA------TTCACTA  CACTGA------TTCACTA  CACTGA------TTCAATA  CACTGA------TTCACTA  CACTGA------TTCAAAA  CACTGA------TTCAAAA  CACTGA------TTCAATA  CACTAA------TTCAATA  CACAGA------TTCAATA  CACTGA------TTCAATA  CACCGA------TTCCATA  CACCGA------TTCCATA  CACTGA------TTCAATA  CACTGA------TTCAATA  CACTGA------TTCAATA  CACTGA------TTCAATA  CACTGA------TTCAATA  CACTGA------TTCAATA  CACTGA------TTCAATA  CACTGA------TTCAATA  CACTGA------TTCAATA  CACTGATTCAATTTCAATA  CACTGATTCAATTTCAATA  CACTGA------TTCAATA  CACTGA------TTCAATA  CACGGA------TTCCATA  CACTGA------TTCAATA  CACTGA------TTCCATA  CACTGA------TTCCATA  CACTGA------TTCAATA  CACTGA------TTCAATA  CACTGA------TTCAATA  CACTGA------TTCAATA  CACTGA------TTCAATA  CATCGA------TTTAATA  CACCGA------TTCAATA | ssmp | Hordeum vulgare |
| 134 | Schedonorus arundinaceus Schedonorus arundinaceus[C] Schedonorus pratensis  Festuca altissima  Festuca ovina  Lolium multiflorum  Lolium perenne  Dactylis glomerata  Helictochloa hookeri  Deschampsia antarctica  Poa palustris  Phleum alpinum  Briza sp  Puccinellia nuttalliana  Ammophila breviligulata  Agrostis stolonifera  Hierochloe odorata  Anthoxanthum odoratum  Phalaris arundinacea  Torreyochloa  Avena sativa  Trisetum cernuum  Aegilops cylindrica  Aegilops geniculata  Aegilops speltoides  Aegilops tauschii  Triticum aestivum  Triticum monococcum aegilopoides Triticum monococcum (modified) Triticum urartu (modified)  Hordeum jubatum  Hordeum vulgare  Hordeum vulgare spontaneum Secale cereale  Bromus vulgaris  Brachypodium distachyon  Diarrhena obovata  Melica mutica  Melica subulata  Oryzopsis asperifolia Achnatherum hymenoides Ampelodesmos mauritanica Piptochaetium avenaceum  Phaenosperma globosum Brachyelytrum aristosum  Bambusa bambosa | 87501  petD intron | petD intron | TGTGACATGATCTAG---------TATTACACTTAC  TGTGACATGATCTAG---------TATTACACTTAC  TGTGACATGATCTAG---------TATTACAGTTAC  TGTGACATGATCCAG---------TATTACACTTAC  TGTGACATGATCCAA---------TATTACACTTAC  TGTGACATGATCTAG---------TATTACAGTTAC  TGTGACATGATCTAG---------TATTACAGTTAC  TGTGACATGATCTAA---------TATTACACTTAT  TGTGACATGATCCAA---------TATTACACTTAC  TGTGACATGATCCAA---------TATTACACTTAC  TGTGACATGATCCAA---------TATTACACTTAC  TGTGACATGATCCAA---------TATTACACTTAC  TGTGACATGATCTAA---------TATTACACTTAC  TGTGACATGATCCAA---------TATTACACTTAC  TGTGACATGATCCAA---------TATTACACTTAC  TGTGACATGATCCAA---------TATTACACTTAC  TGGGACATGATCCAA---------TATTACACTTAC  TGGGACATGATCCAA---------TATTACACTTAC  TGTGACATGATCCAA---------TATTACACTTAC  TGTGACATGATCCAA---------TATTACACTTAC  TGTGACATGATCCAA---------TATTACACTTAC  TGTGACATGATCCAA---------TATTACACTTAC  TGTGACATGATCCAGTTCTCTATTTATTACACTTAC  TGTGACATGATCCAGTTCTCTATTTATTACACTTA-  TGTGACATGATCCAGTTCTCTATTTATTACACTTAC  TGTGACATGATCCAGTTCTCTATTTATTACACTTAC  TGTGACATGATCCAGTTCTCTATTTATTACACTTAC  TGTGACATGATCCAGTTCTCTATTTATTACACTTAC  TGTGACATGATCCAGTTCTCTATTTATTACACTTAC  TGTGACATGATCCAGTTCTCTATTTATTACACTTAC  TGTGACATGATCCAATTCTCTATTTATTACACTTAC  TGTGACATGATCCAATTCTCTATTTATTACACTTAC  TGTGACATGATCCAATTCTCTATTTATTACACTTAC  TGTGACATGATCCAGTTCTCTATTTATTACACTTAC  TGTGACATGATCCAATTCTCTATTTATTACACTTAC  TGTGACATGATCCAATTCTCTATTTATTACACTTAC  TGTGACATGATCCAATTCTCTATTTATTACACTTAC  TGTGACATGATCCAATTCTCTATTTTTTAGACTTAC  TGTGACATGATCCAATTCTCTATTTTTTAGACTTAC  TGTGACATGATCCAATTCTCTATTTATTACACTTAC  TGTAACATGATCCACTTCTCTATTTATTACACTTAC  TGTGACATGATCCAATTCTCTATTTATTACACTTAC  TGTGACATGATCCAATTCTCTATTTATTACACTTAC  TGTGACATGATCCAATTCTCTATTTATTACACTTAC  TGTGACATGATTCAATTCTCTATTTATTACACTTAC  TATCACATGATCTAATTCTCTATTTATTACACTTAC |  | Poeae |
| 135 | Schedonorus arundinaceus Schedonorus arundinaceus[C] Schedonorus pratensis  Festuca altissima  Festuca ovina  Lolium multiflorum  Lolium perenne  Dactylis glomerata  Helictochloa hookeri  Deschampsia antarctica  Poa palustris  Phleum alpinum  Briza sp  Puccinellia nuttalliana  Ammophila breviligulata  Agrostis stolonifera  Hierochloe odorata  Anthoxanthum odoratum  Phalaris arundinacea  Torreyochloa  Avena sativa  Trisetum cernuum  Aegilops cylindrica  Aegilops geniculata  Aegilops speltoides  Aegilops tauschii  Triticum aestivum  Triticum monococcum aegilopoides Triticum monococcum (modified) Triticum urartu (modified)  Hordeum jubatum  Hordeum vulgare  Hordeum vulgare spontaneum Secale cereale  Bromus vulgaris  Brachypodium distachyon  Diarrhena obovata  Melica mutica  Melica subulata  Oryzopsis asperifolia Achnatherum hymenoides Ampelodesmos mauritanica Piptochaetium avenaceum  Phaenosperma globosum Brachyelytrum aristosum  Bambusa bambosa | 87808  petD intron 1 | petD intron 1 | GCATTTACC----CATAAGAA-  GCATTTACC----CATAAGAA-  GCATTTACC----CATAAGAA-  GCATTTACC----CATAAGAA-  GCATTTACC----CATAAGAA-  GCATTTACC----CATAAGAA-  GCATTTACC----CATAAGAA-  GCATTTACC----CATAAGAA-  GCATTTACC----CATAAGAA-  GCATTTACC----CATAAGAA-  GCATTTACC----CATAAGAA-  GCATTTATC----CATAAGAA-  GCATTTGCC----CATAAGAA-  GCATTTACC----TATAAGAA-  GCATTTACC----TATAAGAA-  GCATTTACC----TATAAGAA-  GCATTTACCCATACATAAGAA-  GCATTTACCCATACATAAGAA-  GCATTTACC----CATAAGAA-  GCATTTACC----CATAAGAA-  GCATTTACC----CATAAGAA-  GCATTTACC----CATAAGAA-  GCATTTACC----CATAAGAA-  GCATTTACC----CATAAGAA-  GCATTTACC----CATAAGAA-  GCATTTACC----CATAAGAA-  GCATTTACC----CATAAGAA-  GCATTTACC----CATAAGAA-  GCATTTACC----CATAAGAA-  GCATTTACC----CATAAGAA-  GCATTTACC----CATAAGAA-  GCATTTACC----CATAAGAA-  GCATTTACC----CATAAGAA-  GCATTTACC----CATAAGAA-  GCATTTACC----CATAAGAA-  GCATTTACC----CATAAGAA-  GCATTTACC----CATAAGAA-  GCATTTACC----CATAAGAAT  GCATTTACC----CATAAGAA-  GCATTTACC----CATAAGAA-  GCATTTACC----CATAAGAA-  GCATTTACC----CATAAGAA-  GCATTTACC----AATAAGAA-  GCATTTACC----CATAAGAA-  GCATTTACC----CATAAGAA-  GCATTTACC----CATAAGAA- | ssmp | Anthoxanthinae |
| 136 | Schedonorus arundinaceus Schedonorus arundinaceus[C] Schedonorus pratensis  Festuca altissima  Festuca ovina  Lolium multiflorum  Lolium perenne  Dactylis glomerata  Helictochloa hookeri  Deschampsia antarctica  Poa palustris  Phleum alpinum  Briza sp  Puccinellia nuttalliana  Ammophila breviligulata  Agrostis stolonifera  Hierochloe odorata  Anthoxanthum odoratum  Phalaris arundinacea  Torreyochloa  Avena sativa  Trisetum cernuum  Aegilops cylindrica  Aegilops geniculata  Aegilops speltoides  Aegilops tauschii  Triticum aestivum  Triticum monococcum aegilopoides Triticum monococcum (modified) Triticum urartu (modified)  Hordeum jubatum  Hordeum vulgare  Hordeum vulgare spontaneum Secale cereale  Bromus vulgaris  Brachypodium distachyon  Diarrhena obovata  Melica mutica  Melica subulata  Oryzopsis asperifolia Achnatherum hymenoides Ampelodesmos mauritanica Piptochaetium avenaceum  Phaenosperma globosum Brachyelytrum aristosum  Bambusa bambosa | 88576  IGS |  | AAGTACCGTGCATAG  AAGTACCGTGCATAG  AAGTACCGTGCATAG  AAGTACCGTGCATAG  AAGTACCGCGCATAG  AAGTACCGTGCATAG  AAGTACCGTGCATAG  AAGTACTGTGCATAG  AAGTACCGTGCATAG  AAGTACCGTGCATAG  AAGTAC----CATAG  AAGTACCGTGCATAG  AAGTACCGTGCATAG  AAGTAC----CATAG  AAGTACCGTGCATAG  AAGTACCGTGCATAG  AAGTACCGTGCATAG  AAGTAC----CATAG  AAGTACCGTGCATAG  AAGTACCGTGCATAG  AAGTACCGTGCATAG  AATTACCGTGCATAG  AAGTAC----CATAG  AAGTAC----CATAG  AAGTAC----CATAG  AAGTAC----CATAG  AAGTAC----CATAG  AAGTAC----CATAG  AAGTAC----CATAG  AAGTAC----CATAG  AAGTAC----CATAG  AAGTAC----CATAG  AAGTAC----CATAG  AAGTAC----CATAG  AAGTAC----CGTAG  AAGTACCGTGCATAG  AAGTACCGTGCATAG  AAGTACTGTGTATAG  AAGTACTGTGTATAG  AAGTACCGTGCATAG  AAGTACCGTGCATAG  AAGTACCATGCATAG  AAGTACTGTGCATAG  AAGTACCGTGCATAG  AACTACCGCGCATAG  AAGTACCGTGCATAG |  | Homoplasy |
| 137 | Schedonorus arundinaceus Schedonorus arundinaceus[C] Schedonorus pratensis  Festuca altissima  Festuca ovina  Lolium multiflorum  Lolium perenne  Dactylis glomerata  Helictochloa hookeri  Deschampsia antarctica  Poa palustris  Phleum alpinum  Briza sp  Puccinellia nuttalliana  Ammophila breviligulata  Agrostis stolonifera  Hierochloe odorata  Anthoxanthum odoratum  Phalaris arundinacea  Torreyochloa  Avena sativa  Trisetum cernuum  Aegilops cylindrica  Aegilops geniculata  Aegilops speltoides  Aegilops tauschii  Triticum aestivum  Triticum monococcum aegilopoides Triticum monococcum (modified) Triticum urartu (modified)  Hordeum jubatum  Hordeum vulgare  Hordeum vulgare spontaneum Secale cereale  Bromus vulgaris  Brachypodium distachyon  Diarrhena obovata  Melica mutica  Melica subulata  Oryzopsis asperifolia Achnatherum hymenoides Ampelodesmos mauritanica Piptochaetium avenaceum  Phaenosperma globosum Brachyelytrum aristosum  Bambusa bambosa | 88638  IGS |  | TAAAATCC--------ATTTTATTATG  TAAAATCC--------ATTTTATTATG  TAAAATCC--------ATTTTATTATG  TAAAATCC--------ATTTTATTATG  TAAAATCC--------ATTTTATTATG  TAAAATCC--------ATTTTATTATG  TAAAATCC--------ATTTTATTATG  TAAAATCC--------ATTTTATTATG  TAAAATCC--------ATTTTATTATG  TAAAATCC--------ATTTTATTATG  TAAAATCC--------ATTTTATTATG  TAAAATCC--------ATTTTATTATG  TAAAATCC--------ATTTTATTATG  TAAAATCC--------ATTTTATTATG  TAAAATCC--------ATTTTATTATG  TAAAATCC--------ATTTTATTATG  TAAAATCC--------ATTTTATTATG  TAAAATCC--------ATTTTATTATG  TAAAACCC--------ATTTTATTATG  TAAAATTC--------TTTTTATTATG  TAAAATCC--------ATTTTATTATG  TAAAATCC--------ATTTTATTATG  TAAAATCC--------ATTTTATTATG  TAAAATCC--------ATTTTATTATG  TAAAATCC--------ATTTTATTATG  TAAAATCC--------ATTTTATTATG  TAAAATCC--------ATTTTATTATG  TAAAATCC--------ATTTTATTATG  TAAAATCC--------ATTTTATTATG  TAAAATCC--------ATTTTATTATG  TAAAATCCATTTTTTTATTTTATTATG  TAAAATCCATTTTATTATTTTATTATG  TAAAATCCATTTTATTATTTTATTATG  TAAAATCC--------ATTTTATTATG  TAAAATCC--------ATTTTATTATG  TAAAATCC--------ATTTTATTATG  TAAAATCC--------ATTTTATTATG  TAAAATCC--------TTTTTATTATG  TAAAATCC--------TTTTTATTATG  TAAAATAC--------ATTTTATTATG  TAAAATCC--------ATTTTATTATG  TAAAATCC--------ATTTTATTATG  TAAAATCC--------ATTTTATTATG  TAAAATCC--------TTTTTATTATG  TAAAATCT--------ATTTTATTATG  TAAAATCC--------ATTTTATTATG | ssmp | Hordeum |
| 138 | Schedonorus arundinaceus Schedonorus arundinaceus[C] Schedonorus pratensis  Festuca altissima  Festuca ovina  Lolium multiflorum  Lolium perenne  Dactylis glomerata  Helictochloa hookeri  Deschampsia antarctica  Poa palustris  Phleum alpinum  Briza sp  Puccinellia nuttalliana  Ammophila breviligulata  Agrostis stolonifera  Hierochloe odorata  Anthoxanthum odoratum  Phalaris arundinacea  Torreyochloa  Avena sativa  Trisetum cernuum  Aegilops cylindrica  Aegilops geniculata  Aegilops speltoides  Aegilops tauschii  Triticum aestivum  Triticum monococcum aegilopoides Triticum monococcum (modified) Triticum urartu (modified)  Hordeum jubatum  Hordeum vulgare  Hordeum vulgare spontaneum Secale cereale  Bromus vulgaris  Brachypodium distachyon  Diarrhena obovata  Melica mutica  Melica subulata  Oryzopsis asperifolia Achnatherum hymenoides Ampelodesmos mauritanica Piptochaetium avenaceum  Phaenosperma globosum Brachyelytrum aristosum  Bambusa bambosa | 92283  IGS |  | TGAATCATA-------TTATTTTGA  TGAATCATA-------TTATTTTGA  TGAATCATA-------TTATTTTGA  TGAATCATA-------TTATTTTGA  TGAATCATA-------TTATTTTGA  TGAATCATA-------TTATTTTGA  TGAATCATA-------TTATTTTGA  TGAATCATA-------TTATTTTGA  TGAATCATA-------TTATTTTGA  TGAATCATA-------TTATTTTGA  TGAATCATA-------TTATTTTGA  TGAATCATA-------TTATTTTGA  TGAATCATA-------TTATTTTGA  TGAATCATA-------TTATTTTGA  TGAATCATA-------TTATTTTGA  TGAATCATA-------TTATTTTGA  TGAATCATA-------TTATTTTGA  TGAATCATA-------TTATTTTGA  TGAATCATA-------TTATTTTGA  TGAATCATA-------TTATTTTGA  TGAATCATA-------TTATTTTGA  TGAATCATA-------TTATTTTGA  TGAATCATATTATTTTTTATTTTGA  TGAATCATA-------TTATTTTGA  TGAATCATA-------TTATTTTGA  TGAATCATATTATTTTTTATTTTGA  TGAATCATA-------TTATTTTGA  TGAATCATA-------TTATTTTGA  TGAATCATA-------TTATTTTGA  TGAATCATA-------TTATTTTGA  TGAATCATA-------TTATTTTGA  TGAATCATA-------TTATTTTGA  TGAATCATA-------TTATTTTGA  TGAATCATA-------TTATTTTGA  TGAATCATA-------TTATTTTGA  TGAATCATA-------TTATTTGGA  TGAATCATA-------TTATTTTGA  TGAATCATA-------TTATTTTGG  TGAATCATA-------TTATTTTGA  TGAATCATA-------TTATTTTGA  TGAATCATA-------TTATTTTGA  TGAATCATA-------TTATTTTGA  TGAATCATA-------TTATTTTGA  TGAATCATA-------TTATTTTGA  TGAATCATA-------TTATTTTGA  TGAATCATA-------TTATTTTGA | ssmp | Aegilops cylindra+A. tauschii |
| 139 | Schedonorus arundinaceus Schedonorus arundinaceus[C] Schedonorus pratensis  Festuca altissima  Festuca ovina  Lolium multiflorum  Lolium perenne  Dactylis glomerata  Helictochloa hookeri  Deschampsia antarctica  Poa palustris  Phleum alpinum  Briza sp  Puccinellia nuttalliana  Ammophila breviligulata  Agrostis stolonifera  Hierochloe odorata  Anthoxanthum odoratum  Phalaris arundinacea  Torreyochloa  Avena sativa  Trisetum cernuum  Aegilops cylindrica  Aegilops geniculata  Aegilops speltoides  Aegilops tauschii  Triticum aestivum  Triticum monococcum aegilopoides Triticum monococcum (modified) Triticum urartu (modified)  Hordeum jubatum  Hordeum vulgare  Hordeum vulgare spontaneum Secale cereale  Bromus vulgaris  Brachypodium distachyon  Diarrhena obovata  Melica mutica  Melica subulata  Oryzopsis asperifolia Achnatherum hymenoides Ampelodesmos mauritanica Piptochaetium avenaceum  Phaenosperma globosum Brachyelytrum aristosum  Bambusa bambosa | 92308  IGS |  | TTTCAATTTGTTTTGTTAT  TTTCAATTTGTTTTGTTAT  TTTCAATTTGTTTTGTTAT  TTTCAATTTGTTTTGTTAT  TTTCAA-----TTTGTTAT  TTTCAATTTGTTTTGTTAT  TTTCAATTTGTTTTGTTAT  TTTCAA-----TTTGTTAT  TTTCAA-----TTTGTTAT  TTTCAA-----TTTGTTAT  TTTCAA-----TTTGTTAT  TTTCAA-----TTTGTTAT  TTTCAA-----TTTGTTAT  TTTCAA-----TTTGTTAT  TTTCAA-----TTTGTTAT  TTTCAA-----TTTGTTAT  TTTCCA-----TTTGTTAT  TTTCCA-----TTTGTTAT  TTTCAA-----TTTGTTAT  TTTCAA-----TTTGTTAT  TTTCCA-----TTTGTTAT  TTTCCA-----TTTGTTAT  TTTCAA-----TTTGTTAT  TTTCAA-----TTTGTTAT  TTTCAA-----TTTGTTAT  TTTCAA-----TTTGTTAT  TTTCAA-----TTTGTTAT  TTTCAA-----TTTGTTAT  TTTCAA-----TTTGTTAT  TTTCAA-----TTTGTTAT  TTTCAA-----TTTGTTAT  TTTCAA-----TTTGTTAT  TTTCAA-----TTTGTTAT  TTTCAA-----TTTGTTAT  TTTCAA-----TTTGTTAT  TTTCAA-----TTTTTTAT  TTTCAA-----TTTGTTAT  TTTCAA-----TTTGTTAT  TTTCAA-----TTTGTTAT  TTTCAA-----TTTGTTAT  TTTCAA-----TTTGTTAT  TTTCAA-----TTTGTTAT  TTTCAA-----TTTGTTAT  TTTCAA-----TTTGTTAT  TTTCAA-----TTTGTTAT  TTTCAA-----TTTGTTAT | Ssmp | Lolium+Schedonorus+Festuca altissima |
| 140 | Schedonorus arundinaceus Schedonorus arundinaceus[C] Schedonorus pratensis  Festuca altissima  Festuca ovina  Lolium multiflorum  Lolium perenne  Dactylis glomerata  Helictochloa hookeri  Deschampsia antarctica  Poa palustris  Phleum alpinum  Briza sp  Puccinellia nuttalliana  Ammophila breviligulata  Agrostis stolonifera  Hierochloe odorata  Anthoxanthum odoratum  Phalaris arundinacea  Torreyochloa  Avena sativa  Trisetum cernuum  Aegilops cylindrica  Aegilops geniculata  Aegilops speltoides  Aegilops tauschii  Triticum aestivum  Triticum monococcum aegilopoides Triticum monococcum (modified) Triticum urartu (modified)  Hordeum jubatum  Hordeum vulgare  Hordeum vulgare spontaneum Secale cereale  Bromus vulgaris  Brachypodium distachyon  Diarrhena obovata  Melica mutica  Melica subulata  Oryzopsis asperifolia Achnatherum hymenoides Ampelodesmos mauritanica Piptochaetium avenaceum  Phaenosperma globosum Brachyelytrum aristosum  Bambusa bambosa | 92859  Rpl16 intron 1 | Rpl16 intron 1 | GATGGT--TCTTTCT  GATGGT--TCTTTCT  GATGGT--TCTTTCT  GATGGT--TCTTTCT  GATGGT--TCTTTCT  GATGGT--TCTTTCT  GATGGT--TCTTTCT  GATGGT--TCTTTCT  GATGGT--TCTTTCT  GATGGT--TCTTTCT  GATGGT--TCTTTCT  GATGGT--TCTTTCT  GATGGT--TCTTTCT  GATGGT--TCTTTCT  GATGGT--TCTTTCT  GATGGT--TCTTTCT  GATGGT--TCTTTCT  GATGGT--TCTTTCT  GATGGT--TCTTTCT  GATGGT--TCTTTCT  GATGGT--TCTTTCT  GATGGT--TCTTTCT  GATGGTTCTCTTTCT  GATGGTTCTCTTTCT  GATGGTTCTCTTTCT  GATGGTTCTCTTTCT  GATGGTTCTCTTTCT  GATGGTTCTCTTTCT  GATGGTTCTCTTTCT  GATGGTTCTCTTTCT  GATGGTTCTCTTTCT  GATGGTTCTCTTTCT  GATGGTTCTCTTTCT  GATGGTTCTCTTTCT  GATGGT--TCTTTCT  GATGGT--TCTTTCG  GATGGT--TCTTTCT  GATGGT--TCTTTCG  GATGGT--TCTTTCG  GATGGT--TCTTTCT  GATGGT--TTTTTCT  GATGGT--TCTTTCT  GATGGT--TCTTTCT  GATGGT--TCTTTCT  GATAGT--TCTTTCT  GATGGT--TCTTTCT | Ssmp | Triticeae |
| 141 | Schedonorus arundinaceus Schedonorus arundinaceus[C] Schedonorus pratensis  Festuca altissima  Festuca ovina  Lolium multiflorum  Lolium perenne  Dactylis glomerata  Helictochloa hookeri  Deschampsia antarctica  Poa palustris  Phleum alpinum  Briza sp  Puccinellia nuttalliana  Ammophila breviligulata  Agrostis stolonifera  Hierochloe odorata  Anthoxanthum odoratum  Phalaris arundinacea  Torreyochloa  Avena sativa  Trisetum cernuum  Aegilops cylindrica  Aegilops geniculata  Aegilops speltoides  Aegilops tauschii  Triticum aestivum  Triticum monococcum aegilopoides Triticum monococcum (modified) Triticum urartu (modified)  Hordeum jubatum  Hordeum vulgare  Hordeum vulgare spontaneum Secale cereale  Bromus vulgaris  Brachypodium distachyon  Diarrhena obovata  Melica mutica  Melica subulata  Oryzopsis asperifolia Achnatherum hymenoides Ampelodesmos mauritanica Piptochaetium avenaceum  Phaenosperma globosum Brachyelytrum aristosum  Bambusa bambosa | 93636  Rpl16 intron 1 | Rpl16 intron 1 | CTGGTCGGCTCTTTATTCTT------TATTA  CTGGTCGGCTCTTTATTCTT------TATTA  CTGGTCGGCTCTTTATTCTT------TATTA  CCGGTCGGCTCTTTATTCTT------TATTA  CCGGTCGGCTCTTTATTCTT------TATTA  CTGGTCGGCTCTTTATTCTT------TATTA  CTGGTCGGCTCTTTATTCTT------TATTA  CCGGTCGGC-------TCTT------TATTA  CCGGGGGGCTCTTTATTCTT------TATTA  CCGGTCGGCTCTTTATTCTT------TATTA  CCGGTCGGCTCTTTATTCTT------TATTA  TCGGTCGGCTCTTTATTCTT------TATTA  CAGGGCGGCTCTTTATTAT-------TATTA  CCGGGCGGCTCTTTATTCTT------TATTA  CAGGGCGGC-------TCTT------TATTA  CAGGGGGGC-------TCTT------TATTA  CAGAGCGGC-------TCTT------TATTA  CAGAGGAGC-------TCTT------TTTTA  CAGGGCGGC-------TCTT------TATTA  TAGGGTGGC-------TCTT------TATTA  CAGGATGGC-------TCTT------TATTA  CAGGACGGC-------TCTT------TATTA  CCGGGCGGC-------TCTT------TATTA  CCGGGCGGC-------TCTT------TATTA  CCGGGCGGC-------TCTT------TATTA  CCGGGCGGC-------TCTT------TATTA  CCGGGCGGC-------TCTT------TATTA  CCGGGCGGC-------TCTT------TATTA  CCGGGCGGC-------TCTT------TATTA  CCGGGCGGC-------TCTT------TATTA  CCGGGCGGC-------TCTT------TATTA  CCGGGCGGC-------TCTT------TATTA  CCGGGCGGC-------TCTT------TATTA  CCGGGCGGC-------TCTT------TATTA  CCGGGCGGC-------TCTT------TATTA  CCGGGTGGC-------TCTT-----TAATTA  CCGGGCGGC-------TCTT------TATTA  CGCGGCGGC-------TCTT------TATTA  CGCGGCGGC-------TCTT------TATTA  CCGAGCGGC-------TCTT------TATTA  CCGAGCGGC-------TCTT------TATTA  CCGAGCGGC-------TCTT------TATTA  CCGAGCGGC-------TCTT------TATTA  CCGGGCAGC-------TCTT------TATTA  CCGGACCGC-------TCTT------TATTA  CCGGGCCGC-------TCTT------TATTA |  | Homoplasy |
| 142 | Schedonorus arundinaceus Schedonorus arundinaceus[C] Schedonorus pratensis  Festuca altissima  Festuca ovina  Lolium multiflorum  Lolium perenne  Dactylis glomerata  Helictochloa hookeri  Deschampsia antarctica  Poa palustris  Phleum alpinum  Briza sp  Puccinellia nuttalliana  Ammophila breviligulata  Agrostis stolonifera  Hierochloe odorata  Anthoxanthum odoratum  Phalaris arundinacea  Torreyochloa  Avena sativa  Trisetum cernuum  Aegilops cylindrica  Aegilops geniculata  Aegilops speltoides  Aegilops tauschii  Triticum aestivum  Triticum monococcum aegilopoides Triticum monococcum (modified) Triticum urartu (modified)  Hordeum jubatum  Hordeum vulgare  Hordeum vulgare spontaneum Secale cereale  Bromus vulgaris  Brachypodium distachyon  Diarrhena obovata  Melica mutica  Melica subulata  Oryzopsis asperifolia Achnatherum hymenoides Ampelodesmos mauritanica Piptochaetium avenaceum  Phaenosperma globosum Brachyelytrum aristosum  Bambusa bambosa | 93929  Rpl16 intron 1 | Rpl16 intron 1 | AAAA---GCAG  AAAA---GCAG  AAAA---GCAG  AAAA---GCAG  AAAA---GCAG  AAAA---GCAG  AAAA---GCAG  AAAA---GCAG  AAAA---GCAG  AAAA---GCAG  AAAA---GCAG  AAAA---GCAG  AAAA---GCAG  AAAA---GCAG  AAAA---GCAG  AAAA---GCAG  AAAA---GCAG  AAAA---GCAG  AAAA---GCAG  AAAA---GCAG  AAAA---GCAG  AAAA---GCAG  AAAAATTGCAG  AAAAATTGCAG  AAAAATTGCAG  AAAAATTGCAG  AAAAATTGCAG  AAAAATTGCAG  AAAAATTGCAG  AAAAATTGCAG  AAAAATTGCAG  AAAAAGTGCAG  AAAAAGTGCAG  AAAAATTGCAG  AAAAATTGCAG  AAAAATTGCAG  AAAAATTGCAG  AAAAATTGCAG  AAAATTTGCAG  AAAAATTGCAG  AAAAATTGCAG  AAAAATTGCAG  AAAAATTGCAG  AAAAATTGCAG  AAAAATTGCAG  AAAAATTGCAG |  | Poeae |
| 143 | Schedonorus arundinaceus  Schedonorus arundinaceus[C]  Schedonorus pratensis  Festuca altissima  Festuca ovina  Lolium multiflorum  Lolium perenne  Dactylis glomerata  Helictochloa hookeri  Deschampsia antarctica  Poa palustris  Phleum alpinum  Briza sp  Puccinellia nuttalliana  Ammophila breviligulata  Agrostis stolonifera  Hierochloe odorata  Anthoxanthum odoratum  Phalaris arundinacea  Torreyochloa  Avena sativa  Trisetum cernuum  Aegilops cylindrica  Aegilops geniculata  Aegilops speltoides  Aegilops tauschii  Triticum aestivum  Triticum monococcum aegilopoides  Triticum monococcum (modified)  Triticum urartu (modified)  Hordeum jubatum  Hordeum vulgare  Hordeum vulgare spontaneum  Secale cereale  Bromus vulgaris  Brachypodium distachyon  Diarrhena obovata  Melica mutica  Melica subulata  Oryzopsis asperifolia  Achnatherum hymenoides  Ampelodesmos mauritanica  Piptochaetium avenaceum  Phaenosperma globosum  rachyelytrum aristosum  Bambusa bambosa | 93971  Rpl16 intron 1 | Rpl16 intron 1 | TTCATTTATAATAGAGGTATTTATTCATATAT-----TTATAATAGAGGTATTTATTCATATAG  TTCATTTATAATAGAGGTATTTATTCATATAT-----TTATAATAGAGGTATTTATTCATATAG  TTC---------------------------AT-----TTATAATAGAGGTATTTATTCATATAG  TTC---------------------------AT-----TTATAATAGAGGTATTTATTCATATAG  TTC---------------------------AT-----TTATAATGGAGGTATTTATTCATATAG  TTC---------------------------AT-----TTATAATAGAGGTATTTATTCATATAG  TTC---------------------------AT-----TTATAATAGAGGTATTTATTCATATAG  TTC---------------------------AT-----TTATAATAGAGATATTTATTCATATAG  TTC---------------------------AT-----TTATAATAGAGGTATTTATTCATATAG  TTC---------------------------AT-----TTATAATAGAGGTATTTATTCATATAG  TTC---------------------------AT-----TTATAATAGAGGTATTTATTCATATAG  TTC---------------------------AT-----TTATAATAGAGGTATTTATTCATATAG  CTC---------------------------AT-----TTATGATAGAGATATTTATTCATATAG  TTC---------------------------AT-----TTATAATAGAGGTATTTATTCATATAG  CTC---------------------------AT-----TTATGATAGAGATATTTATTCATATAG  CTC---------------------------AT-----TTATGATAGAGATATTTATTCATATAG  CTC---------------------------AT-----TTATGATAGATATATTTATTCATATAG  CTC---------------------------AT-----TTATGATAGAGATATTTATTCATATAG  CTC---------------------------AT-----TTATGATAGAGATATTTATTCATATAG  CTC---------------------------AT-----TTATGATAGAGATATTTATTCATATAG  CTC---------------------------AT-----TTATGATAGAGATATTTATTCATATAG  CTC---------------------------AT-----TTATGATAGAGATATTTATTCATATAG  CTC---------------------------AT-----TTATGATAGAGGTATTTATTCATATAG  CTC---------------------------AT-----TTATGATAGAGGTATTTATTCATATAG  CTC---------------------------AT-----TTATGATAGAGGTATTTATTCATATAG  CTC---------------------------AT-----TTATGATAGAGGTATTTATTCATATAG  CTC---------------------------AT-----TTATGATAGAGGTATTTATTCATATAG  CTC---------------------------AT-----TTATGATAGAGGTATTTATTCATATAG  CTC---------------------------AT-----TTATGATAGAGGTATTTATTCATATAG  CTC---------------------------ATTTATGTTATGATAGAGGTATTTATTCATATAG  CTC---------------------------AT-----TTATGATAGAGGTATTTATTCATATAG  CTC---------------------------AT-----TTATGATAGAGGTATTTATTCATATAG  CTC---------------------------AT-----TTATGATAGAGGTATTTATTCATATAG  CTC---------------------------AT-----TTATGATAGAGGTATTTATTCATATAG  CTC---------------------------AT-----TTATGATAGAGGTATTTATTCATATAG  CTC---------------------------AT-----TTATGATAGAGATATTTATTCAGATAG  CTC---------------------------AT-----TTATGATAGAGGTATTTATTCATATAG  CTC---------------------------AT-----TTATGATAGAGGTATTTATTCATATAG  CTC---------------------------AT-----TTATGATAGAGGTATTTATTCATATAG  CTC---------------------------AT-----TTATGATAGAAGTATTTATTCATATAG  CTC---------------------------AT-----TTATGATAGAAGTATTTATTCATATAG  CTC---------------------------AT-----TTATGATAGAAGTATTTATTCATATAG  CTC---------------------------TT-----TTATGATAGAAGTA-----TCATATAG  CTC---------------------------AT-----TTATGATAGATGTATTTATTCATATAG  CTC---------------------------AT-----TTATAATAGATGTATTTATTCATATAG  CTC---------------------------AT-----TTATGATAGATGTA-----TCATATAG | Ssmp | Schedonorus arundinaceus |
| 144 | Schedonorus arundinaceus Schedonorus arundinaceus[C] Schedonorus pratensis  Festuca altissima  Festuca ovina  Lolium multiflorum  Lolium perenne  Dactylis glomerata  Helictochloa hookeri  Deschampsia antarctica  Poa palustris  Phleum alpinum  Briza sp  Puccinellia nuttalliana  Ammophila breviligulata  Agrostis stolonifera  Hierochloe odorata  Anthoxanthum odoratum  Phalaris arundinacea  Torreyochloa  Avena sativa  Trisetum cernuum  Aegilops cylindrica  Aegilops geniculata  Aegilops speltoides  Aegilops tauschii  Triticum aestivum  Triticum monococcum aegilopoides Triticum monococcum (modified) Triticum urartu (modified)  Hordeum jubatum  Hordeum vulgare  Hordeum vulgare spontaneum Secale cereale  Bromus vulgaris  Brachypodium distachyon  Diarrhena obovata  Melica mutica  Melica subulata  Oryzopsis asperifolia Achnatherum hymenoides Ampelodesmos mauritanica Piptochaetium avenaceum  Phaenosperma globosum Brachyelytrum aristosum  Bambusa bambosa | 94017  Rpl16 intron 1 | Rpl16 intron 1 | AGGTATTTATTCATATAG  AGGTATTTATTCATATAG  AGGTATTTATTCATATAG  AGGTATTTATTCATATAG  AGGTATTTATTCATATAG  AGGTATTTATTCATATAG  AGGTATTTATTCATATAG  AGATATTTATTCATATAG  AGGTATTTATTCATATAG  AGGTATTTATTCATATAG  AGGTATTTATTCATATAG  AGGTATTTATTCATATAG  AGATATTTATTCATATAG  AGGTATTTATTCATATAG  AGATATTTATTCATATAG  AGATATTTATTCATATAG  ATATATTTATTCATATAG  AGATATTTATTCATATAG  AGATATTTATTCATATAG  AGATATTTATTCATATAG  AGATATTTATTCATATAG  AGATATTTATTCATATAG  AGGTATTTATTCATATAG  AGGTATTTATTCATATAG  AGGTATTTATTCATATAG  AGGTATTTATTCATATAG  AGGTATTTATTCATATAG  AGGTATTTATTCATATAG  AGGTATTTATTCATATAG  AGGTATTTATTCATATAG  AGGTATTTATTCATATAG  AGGTATTTATTCATATAG  AGGTATTTATTCATATAG  AGGTATTTATTCATATAG  AGGTATTTATTCATATAG  AGATATTTATTCAGATAG  AGGTATTTATTCATATAG  AGGTATTTATTCATATAG  AGGTATTTATTCATATAG  AAGTATTTATTCATATAG  AAGTATTTATTCATATAG  AAGTATTTATTCATATAG  AAGTA-----TCATATAG  ATGTATTTATTCATATAG  ATGTATTTATTCATATAG  ATGTA-----TCATATAG |  | Homoplasy |
| 145 | Schedonorus arundinaceus Schedonorus arundinaceus[C] Schedonorus pratensis  Festuca altissima  Festuca ovina  Lolium multiflorum  Lolium perenne  Dactylis glomerata  Helictochloa hookeri  Deschampsia antarctica  Poa palustris  Phleum alpinum  Briza sp  Puccinellia nuttalliana  Ammophila breviligulata  Agrostis stolonifera  Hierochloe odorata  Anthoxanthum odoratum  Phalaris arundinacea  Torreyochloa  Avena sativa  Trisetum cernuum  Aegilops cylindrica  Aegilops geniculata  Aegilops speltoides  Aegilops tauschii  Triticum aestivum  Triticum monococcum aegilopoides Triticum monococcum (modified) Triticum urartu (modified)  Hordeum jubatum  Hordeum vulgare  Hordeum vulgare spontaneum Secale cereale  Bromus vulgaris  Brachypodium distachyon  Diarrhena obovata  Melica mutica  Melica subulata  Oryzopsis asperifolia Achnatherum hymenoides Ampelodesmos mauritanica Piptochaetium avenaceum  Phaenosperma globosum Brachyelytrum aristosum  Bambusa bambosa | 94071  Rpl16 intron 1 | Rpl16 intron 1 | CAATAGGTTGGT------TAGTAGTTA  CAATAGGTTGGT------TAGTAGTTA  CAATAGGTTGGT------TAGTAGTTA  CAATAGGTTGGT------TAGTAGTTA  CAATAGGTTGGT------TAGTAGTTA  CAATAGGTTGGT------TAGTAGTTA  CAATAGGTTGGT------TAGTAGTTA  CAATAGGTTGGT------TAGTAGTTA  CAATAGGTTGGT------TAGTAGTTA  CAATAGGTTGGT------TAGTAGTTA  CAATAAGTTGGT------TAGTAGTTA  CAATAGGTTGGT------TAGTAGTTA  CAATAGGTTGGT------TATTAGTTA  CAATAAGTTGGT------TAGTAGTTA  CAATAGGTTGGT------TATTAGTTA  CAATAGGTTGGT------TATTAGTTA  CAATAGGTTGGT------TATTAGTTA  CAATAGGTTGGT------TATTAGTTA  CAATAGGTTGGT------TATTAGTTA  CAATAGGTTGGT------TATTAGTTA  CAATAGGTTGGT------TATTAGTTA  CAATAGGTTGGT------TATTAGTTA  CAATAGGTTGGT------TATTAGTTA  CAATAGGTTGGT------TATTAGTTA  CAATAGGTTGGT------TATTAGTTA  CAATAGGTTGGT------TATTAGTTA  CAATAGGTTGGT------TATTAGTTA  CAATAGGTTGGT------TATTAGTTA  CAATAGGTTGGT------TATTAGTTA  CAATAGGTTGGT------TATTAGTTA  CAATAGGTTGGTTATTAGTATTAGTTA  CAATAGGTTGGTTATTAGTATTAGTTA  CAATAGGTTGGTTATTAGTATTAGTTA  CAATAGGTTGGT------TATTAGTTA  CAATAGGTTGGT------TATTAGTTA  CAATAGGTTGGT------TATTAGTTA  CAATAGGTTGGT------TATTAGTTA  CAATAGGTTAGT------TATTAGTTA  CAATAGGTTGGT------TATTAGTTA  CAATAGGTTGGT------TATTAGTTA  CAATAGGTTGGT------TATTAGTTA  CAATATGTTGGT------TATTAGTTA  CAATAGGTTGCT------TATTAGTTA  CAATAGGTTCGT------TATTAGTTA  CAATAGGTTGGT------TATTAGTTA  CAATAGGTTGGT------TATTAGTTA |  | Hordeum |
| 146 | Schedonorus arundinaceus Schedonorus arundinaceus[C] Schedonorus pratensis  Festuca altissima  Festuca ovina  Lolium multiflorum  Lolium perenne  Dactylis glomerata  Helictochloa hookeri  Deschampsia antarctica  Poa palustris  Phleum alpinum  Briza sp  Puccinellia nuttalliana  Ammophila breviligulata  Agrostis stolonifera  Hierochloe odorata  Anthoxanthum odoratum  Phalaris arundinacea  Torreyochloa  Avena sativa  Trisetum cernuum  Aegilops cylindrica  Aegilops geniculata  Aegilops speltoides  Aegilops tauschii  Triticum aestivum  Triticum monococcum aegilopoides Triticum monococcum (modified) Triticum urartu (modified)  Hordeum jubatum  Hordeum vulgare  Hordeum vulgare spontaneum Secale cereale  Bromus vulgaris  Brachypodium distachyon  Diarrhena obovata  Melica mutica  Melica subulata  Oryzopsis asperifolia Achnatherum hymenoides Ampelodesmos mauritanica Piptochaetium avenaceum  Phaenosperma globosum Brachyelytrum aristosum  Bambusa bambosa | 94261  IGS |  | AATCTTAT----AGAAAGAG  AATCTTAT----AGAAAGAG  AATCTTAT----AGAAAGAG  AATCTTAT----AGAAAGAG  AATCTTAT----AGAAAGAG  AATCTTAT----AGAAAGAG  AATCTTAT----AGAAAGAG  AATCTTAT----AGAAAGAG  AATCTTAT----AGAAAGAG  AATCTTAT----AGAAAGAG  AATCTTAT----AGAAAGAG  AATCTTAT----AGAAAGAG  AATCTTAT----AGAAAGAG  AATCTTAT----AGAAAGAG  AATCTTAT----AGAAAGAG  AATCTTAT----AGAAAGAG  AATCTTAT----AGAAAGAG  AATCTTAT----AGAAAGAG  AATCTTAT----AGAAAGAG  AATCTTAT----AGAAAGAG  AATCTTATAGAAAGAAAGAG  AATCTTATAGAAAGAAAGAG  AATCTTAT----AGAAAGAG  AATCTTAT----AGAAAGAG  AATCTTAT----AGAAAGAG  AATCTTAT----AGAAAGAG  AATCTTAT----AGAAAGAG  AATCTTAT----AGAAAGAG  AATCTTAT----AGAAAGAG  AATCTTAT----AGAAAGAG  AATCTTAT----AGAAAGAG  AATCTTAT----AGAAAGAG  AATCTTAT----AGAAAGAG  AATCTTAT----AGAAAGAG  AATCTTAT----AGAAAGAG  AATCTTAT----AGAAAGAG  AATCTTAT----AGAAAGAG  AATCTTAT----AGAAAGAG  AATCTTAT----AGAAAGAG  AATCTTAT----AGAAAGAG  AATCTTAT----AGAAAGAG  AATCTTAT----AGAAAGAG  AATCTTAT----AGAAAGAG  AATCTTAT----AGAAAGAG  AACTTTAT----AGAAAGAT  AATCTTAT----AGAAAGAG | ssmp | Aveninae+Koelerinae |
| 147 | Schedonorus arundinaceus Schedonorus arundinaceus[C] Schedonorus pratensis  Festuca altissima  Festuca ovina  Lolium multiflorum  Lolium perenne  Dactylis glomerata  Helictochloa hookeri  Deschampsia antarctica  Poa palustris  Phleum alpinum  Briza sp  Puccinellia nuttalliana  Ammophila breviligulata  Agrostis stolonifera  Hierochloe odorata  Anthoxanthum odoratum  Phalaris arundinacea  Torreyochloa  Avena sativa  Trisetum cernuum  Aegilops cylindrica  Aegilops geniculata  Aegilops speltoides  Aegilops tauschii  Triticum aestivum  Triticum monococcum aegilopoides Triticum monococcum (modified) Triticum urartu (modified)  Hordeum jubatum  Hordeum vulgare  Hordeum vulgare spontaneum Secale cereale  Bromus vulgaris  Brachypodium distachyon  Diarrhena obovata  Melica mutica  Melica subulata  Oryzopsis asperifolia Achnatherum hymenoides Ampelodesmos mauritanica Piptochaetium avenaceum  Phaenosperma globosum Brachyelytrum aristosum  Bambusa bambosa | 101119  IRb | IRb | TCTTGTGGT------TTCCTCGGT  TCTTGTGGT------TTCCTCGGT  TCTTGTGGT------TTCCTCGGT  TCTTGTGGT------TTCCTCGGT  TCTTGTGGT------TTCCTCGGT  TCTTGTGGT------TTCCTCGGT  TCTTGTGGT------TTCCTCGGT  TCTTGTGGT------TTCCTCGGT  TCTTGTGGT------TTCCTCGGT  TCTTGTGGT------TTCCTCGGT  TCTTGTGGT------TTCCTCGGT  TCTTGTGGT------TTCCTCGGT  TCTTGTGGT------TTCTTCGGT  TCTTGTGGT------TTCCTCGGT  TCTTGTGGT------TTCCTCGGT  TCTTGTGGT------TTCCTCGGT  TCTTGTGGT------TTCCTCGGT  TCTTGTGGT------TTCCTCGGT  TCTTGTGGT------TTCCTCGGT  TCTTGTGGT------TTCCTCGGT  TCTTGTGGT------TTCCTCGGT  TCTTGTGGT------TTCCTCGGT  TCTTGTGGTTTCCTCTTCCTCGGT  TCTTGTGGTTTCCTCTTCCTCGGT  TCTTGTGGTTTCCTCTTCCTCGGT  TCTTGTGGTTTCCTCTTCCTCGGT  TCTTGTGGTTTCCTCTTCCTCGGT  TCTTGTGGTTTCCTCTTCCTCGGT  TCTTGTGGTTTCCTCTTCCTCGGT  TCTTGTGGTTTCCTCTTCCTCGGT  TCTTGTGGTTTCCTCTTCCTCGGT  TCTTGTGGTTTCCTCTTCCTCGGT  TCTTGTGGTTTCCTCTTCCTCGGT  TCTTGTGGTTTCCTCTTCCTCGGT  TCTTGTGGTTTCCTCTTCCTCGGT  TCTTGTGGT------TTCCTCGGT  TCTTGTGGT------TTCCTCGGT  TCTTGTGGT------TTCCTCGGT  TCTTGTGGT------TTCCTCGGT  TCTTGTGGT------TTCCTCGGT  TCTTGTGGT------TTCCTCGGT  TCTTGTGGT------TTCCTCGGT  TCTTGTGGT------TTCCTCGGT  TCTTGTGGT------TTCCTCGGT  TCTTGTGGT------TTCCTCGGT  TCTTGTGGT------TTCCTCGGT | Ssmp | Bromeae+Triticeae |
| 148 | Schedonorus arundinaceus  Schedonorus arundinaceus[C]  Schedonorus pratensis  Festuca altissima  Festuca ovina  Lolium multiflorum  Lolium perenne  Dactylis glomerata  Helictochloa hookeri  Deschampsia antarctica  Poa palustris  Phleum alpinum  Briza sp  Puccinellia nuttalliana  Ammophila breviligulata  Agrostis stolonifera  Hierochloe odorata  Anthoxanthum odoratum  Phalaris arundinacea  Torreyochloa  Avena sativa  Trisetum cernuum  Aegilops cylindrica  Aegilops geniculata  Aegilops speltoides  Aegilops tauschii  Triticum aestivum  Triticum monococcum aegilopoides  Triticum monococcum (modified)  Triticum urartu (modified)  Hordeum jubatum  Hordeum vulgare  Hordeum vulgare spontaneum  Secale cereale  Bromus vulgaris  Brachypodium distachyon  Diarrhena obovata  Melica mutica  Melica subulata  Oryzopsis asperifolia  Achnatherum hymenoides  Ampelodesmos mauritanica  Piptochaetium avenaceum  Phaenosperma globosum  Brachyelytrum aristosum  Bambusa bambosa | 101249  irb | irB | TGGGCTGGCATAGTAGTTATTACCTTTGCAATTGCGGTTCGAATCTATCCGATCTTTATCTTTTTGCTCAAAGAACGAAGAAAACCCCTTGTCGAAGCCCTTTATGATAAGCTTCCCTGGATCTGGGAAGTTTCTCTTTCACG  TGGGCTGGCATAGTAGTTATTACCTTTGCAATTGCGGTTCGAATCTATCCGATCTTTATCTTTTTGCTCAAAGAACGAAGAAAACCCCTTGTCGAAGCCCTTTATGATAAGCTTCCCTGGATCTGGGAAGTTTCTCTTTCACG  TGGGCTGGCATAGTAGTTATTACCTTTGCAATTGCGGTTCGAATCTATCCGATCTTTATCTTTTTGCTCAAAGAACGAAGAAAACCCCTTGTCGAAGCCCTTTATGATAAGCTTCCCTGGATCTGGGAAGTTTCTCTTTCACG  TGGGCTGGCATAGTAGTTATTACCTTTGCAATTGCGGTTCGAATCTATCCGATCTTTATCTTTTTGCTCAAAGAACGAAGAAAACCCCTTGTCGAAGCCCTTTATGATAAGCTTCCCTGGATCTGGGAAGTTTCTCTTTCACG  TGGGCTGGCATAGTAGTTATTACCTTTGCAATTGGGGTTCGAATCTATCCGATCTTTATCTTTTTGCTCAAAGAACGAAGAAAACCCCTTGTCGAAGCCCTTTATGATAAGCTTCCCTGGATCTGGGAAGTTTCTCTTTCACG  TGGGCTGGCATAGTAGTTATTACCTTTGCAATTGCGGTTCGAATCTATCCGATCTTTATCTTTTTGCTCAAAGAACGAAGAAAACCCCTTGTCGAAGCCCTTTATGATAAGCTTCCCTGGATCTGGGAAGTTTCTCTTTCACG  TGGGCTGGCATAGTAGTTATTACCTTTGCAATTGCGGTTCGAATCTATCCGATCTTTATCTTTTTGCTCAAAGAACGAAGAAAACCCCTTGTCGAAGCCCTTTATGATAAGCTTCCCTGGATCTGGGAAGTTTCTCTTTCACG  TGGGCTGGCATAGTAGTTATTACCTTTGCAATTGCGGTTCGAGTCTATCCGATCTTTATCTTTTTGCTCAAAGAACGAAGAAAACCCCTTGTCGAAGCCCTTTATGATAAGCTTCCCTGGATCTGGGAAGTTTCTCTTTCACG  TGGGCTGGCATAGTAG--------TTTGCAATTGCGGTTCGAATCTATCCGATCTTTATCTTTTTGCTCAAAGAACGAAGAAAACCCCTTGTCGAAGCCCTTTATGATAAGCTTCCCTGGATCTGGGAAGTTTCTCTTTCACG  TGGGCTGGCATAGTAGTTATTACCTTTGCAATTGCGGTTCGAATCTATCCGATCTTTATCTTTTTGCTCAAAGAACGAAGAAAACCCCTTGTCGAAGCCCTTTATGATAAGCTTCCCTGGATCTGGGAAGTTTCTCTTTCACG  TGGGCTGGCATAGTAGTTATTACCTTTGCAATTGCGGTTCGAATCTATCCGATCTTTATCTTTTTGCTCAAAGAACGAAGAAAACCCCTTGTCGAAGCCCTTTATGATAAGCTTCCCTGGATCTGGGAAGTTTATCTTTCACG  TGGGCTGGCATAGTAGTTATTACCTTTGCAATTGCGGTTCGAATCTATCCGATCTTTATCTTTTTGCTCAAAGAACGAAGAAAACCCCTTGTCGAAGCCCTTTATGATAAGCTTCCCTGGATCTGGGAAGTTTCTCTTTCACG  TGGGCTGGCATAGTAGTTATTACCTTTGCAATTGCGGTTCGAATCTATCCGATCTTTATCTTTTTGCTCAAAGAACGAATAAAACCCCTTGTCGAAGCCCTTTATGATAAGCTTCCCTGGATCTGGGAAGTTTCTCTTTCACG  TGGGCTGGCAGAGTAGTTATTACCTTTGCAATTGCGGTTCGAATCTATCCGATCTTTATCTTTTTGCTCAAAGAACGAAGAAAACCCCTTGTCGAAGCCCTTTATGATAAGCTTCCCTGGATCTGGGAAGTTTCTCTTTCACG  TGGGCTGGCATAGTAGTTATTACCTTTGCAATTGCGGTTCGAATCTATCCGATCTTTATCTTTTTGCTCAAAGAACGAATAAAACCCCTTGTCGAAGCCCTTTATGATAAGCTTCCCTGGATCTGGGAAGTTTCTCTTTCACG  TGGGCTGGCATAGTAGTTATTACCTTTGCAATTGCGGTTCGAATCTATCCGATCTTTATCTTTTTGCTCAAAGAACGAATAAAACCCCTTGTCGAAGCCCTTTATGATAAGCTTCCCTGGATCTGGGAAGTTTCTCTTTCACG  TGGGCTGGCATAGTAGTTATTACCTTTGCAATTGCGGTTCGAATCTATCCGATCTTTATCTTTTTGCTCAAAGAACGAATAAAACCCCTTGTCGAAGCCCTTTATGATAAGCTTCCCTGGATCTGGGAAGTTTCTCTTTCACG  TGGGCTGGCATAGTAGTTATTACCTTTGCAATTGCGGTTCGAATCTATCCGATCTTTATCTTTTTGCTCAAAGAACGAATAAAATCCCTTGTCGAAGCCCTTTATGATAAGCTTCCCTGGATCTGGGAAGTTTCTCTTTCACG  TGGGCTGGCATAGTAGTTATTACCTTTGCAATTGCGGTTCGAATCTATCCGCTCTTTATCTTTTTGCTCAAAGAACGAATAAAACCCCTTGTCGAAGCCCTTTATGATAAGCTTCCCTGGATCTGGGAAGTTTCTCTTTCACG  TGGGCTGGCATAGTAGTTATTACCTTTGCAATTGCGGTTCGAATCTATCCGATCTTTATCTTTTTGCTCAAAGAACGAATAAAACCCCTTGTCGAAGCCCTTTATGATAAGCTTCCCTGGATCTGGGAAGTTTCTCTTTCACG  TGAGCTGGCATAGTAGTTATTACCTTTGCAATTGCGGTTCGAATCTATCCGATCTTTATCTTTTTGCTCAAAGAACGAATAAAACCCCTTGTCGAAGCCCTTTATGATAAGCTTCCCTGGATCTGGGAAGTTTCTCTTTCACG  TGGGCTGGCATAGTAGTTATTACCTTTGCAATTGCGGTTCGAATCTATCCGATCTTTATCTTTTTGCTCAAAGAACGAATAAAACCCCTTGTCGAAGCCCTTTATGATAAGCTTCCCTGGATCTGGGAAGTTTCTCTTTCACG  TGGGCTGG-----------------------------------------------------------------------------------------------------------------------------------CACG  TGGGCTGG-----------------------------------------------------------------------------------------------------------------------------------CACG  TGGGCCGG-----------------------------------------------------------------------------------------------------------------------------------CACG  TGGGCTGG-----------------------------------------------------------------------------------------------------------------------------------CACG  TGGGCCGG-----------------------------------------------------------------------------------------------------------------------------------CACG  TGGGCTGG-----------------------------------------------------------------------------------------------------------------------------------CACG  TGGGCTGG-----------------------------------------------------------------------------------------------------------------------------------CACG  TGGGCTGG-----------------------------------------------------------------------------------------------------------------------------------CACG  TGGGCTGG-----------------------------------------------------------------------------------------------------------------------------------CACG  TGGGCTGG-----------------------------------------------------------------------------------------------------------------------------------CACG  TGGGCTGG-----------------------------------------------------------------------------------------------------------------------------------CACG  TGGGCTGG-----------------------------------------------------------------------------------------------------------------------------------CACG  TGGGCTGG-----------------------------------------------------------------------------------------------------------------------------------CACG  TGGGCTGGCATAGTAGTTATTACCTTTGCAATTGCGGTTCGAATCTATCCGATCTTTATCTTTTTGCTCAAAGAACGAATAAAACCCCTTGTCGAAGCCCTTTATGATAAGCTTCCCTGGATCTGGGAAGTTTCTCTTTCACG  TGGGCTGGCATAGTAGTTATTACCTTTGCAATTGCGGTTCGAATCTATCCGATCTTTATCTTTTTGCTCAAAGAACGAATAAAACCCCTTGTCGAAGCCCTTTATGATAAGCTTCCCTGGATCTGGGAAGTTTCTCTTTCACG  TGGGCTGGCATAGTAGTTATTACCTTTGCAATTGCGGTTCGAATCTATCCGATCTTTATCTTTTTGCTCAAAGAACGAATAAAACCCCTTGTCGAAGCCCTTTATGATAAGCTTCCCTCGATCTTGGAAGTTTCTCTTTCATG  TGGGCTGGCATAGTAGTTATTACCTTTGCAATTGCGGTTCGAATCTATCCGATCTTTATCTTTTTGCTCAAAGAACGAATAAAACCCCTTGTCGAAGCCCTTTATGATAAGCTTCCCTCGATCTTGGAAGTTTCTCTTTCATG  TGGGCTGGCATAGTAGTTATTACCTTTGCAATTGCGGTTCGAATCTATCCGATCTTTATCTTTTTGCTCAAAGAACGAATAAAACCCCTTGTCGAAGCCCTTTATGATAAGCTTCCCTGGATCTGGGAAGTTTCTCTTTCACG  TGGGCTGGCATAGTAGTTATTACCTTTGCAATTGCGGTTCTAGTCTATCCGATCTTTATCTTTTTGCTCAAAGAACGAATAAAACCCCTTGTCGAAGCCCTTTATGATAAGCTTCCCTGGATCTGGGAAGTTTCTCTTTCACG  TGGGCTGGCATAGTAGTTATTACCTTTGCAATTGCGGTTCGAATCTATCCGATCTTTATCTTTTTGCTCAAAGAACGAATAAAACCCCTTGTCGAAGCCCTTTATGATAAGCTTCCCTGGATCTGGGAAGTTTCTCTTTCACG  TGGGCTGGCATAGTAGTTATTACCTTTGCAATTGCGGTTCGAATCTATCGGATCTTTATCTTTTTGCTCAAAGAACGAATAAAACCCCTTGTCGAAGCCCTTTATGATAAGCTTCCCTGGATCTGGGAAGTTTCTCTTTCACG  TGGGCTGGCATAGTAGTTATTACCTTTGCAATTGCGGTTCGAATCTATCCGATCTTTATCTTTTTGCTCAAAGAACGAATAAAACCCCTTGTCGAAGCCCTTTATGATAAGCTTCCCTGGATCTGGGAAGTTTCTCTTTCACG  TGGGCTGGCATAGTAGTTATTACCTTTGCAATTGCGGTTCGAATCTATCCGATCTTTATCTTTTTGCTCAAAGAACAAATAAAACCCCTTGTCGAAGCCCTTTATGATAAGCTTCCCTGGATCTGGGAAGTTTCTCTTTCACG  TGGGCTGGCATAGTAGTTATTACCTTTGCAATTGCGGTTCGAATCTATCCGATCTTTATCTTTTTGCTCAAAGAACGAATAAAACCCCTTGTCGAAGCCCTTTATGATAAGCTTCCCTGGATCTGGGAAGTTTCTCTTTCACG |  | Bromeae+Triticeae |
| 149 | Schedonorus arundinaceus Schedonorus arundinaceus[C] Schedonorus pratensis  Festuca altissima  Festuca ovina  Lolium multiflorum  Lolium perenne  Dactylis glomerata  Helictochloa hookeri  Deschampsia antarctica  Poa palustris  Phleum alpinum  Briza sp  Puccinellia nuttalliana  Ammophila breviligulata  Agrostis stolonifera  Hierochloe odorata  Anthoxanthum odoratum  Phalaris arundinacea  Torreyochloa  Avena sativa  Trisetum cernuum  Aegilops cylindrica  Aegilops geniculata  Aegilops speltoides  Aegilops tauschii  Triticum aestivum  Triticum monococcum aegilopoides Triticum monococcum (modified) Triticum urartu (modified)  Hordeum jubatum  Hordeum vulgare  Hordeum vulgare spontaneum Secale cereale  Bromus vulgaris  Brachypodium distachyon  Diarrhena obovata  Melica mutica  Melica subulata  Oryzopsis asperifolia Achnatherum hymenoides Ampelodesmos mauritanica Piptochaetium avenaceum  Phaenosperma globosum Brachyelytrum aristosum  Bambusa bambosa | 101388  IRb | IRb | CACGGTATTGGGATCG  CACGGTATTGGGATCG  CACGGTATTGGGATCG  CACGGTATTGGGATCG  CACGGTATTGGGATCG  CACGGTATTGGGATCG  CACGGTATTGGGATCG  CACGGTATTGGGATCG  CACGGTATTGGGATCG  CACGGTATTGGGATCG  CACGGTATTAGGATCG  CACGGTATTAGGATCG  CACGGTATTGGGATCG  CACGGTATTGGGATCG  CACGGTATTGGGATCG  CACGGTATTGGGATCG  CACGGTATTGGGATCG  CACGGTATTGGGATCG  CACGGTATTGGGATCG  CACGGTATTGGGATCG  CACGGTATTGAGATCG  CACGGTATTGGGATCG  CACG-----GGGATCG  CACG-----GGGATCG  CACGGTATTGGGATCG  CACG-----GGGATCG  CACGGTATTGGGATCG  CACG-----GGGATCG  CACG-----GGGATCG  CACG-----GGGATCG  CACGGTATTGGGATCG  CACGGTATTGGGATCG  CACGGTATTGGGATCG  CACGGTATTGGGATCG  CACGGTATTGGGATCG  CACGGTATTGGGATCG  CACGGTATTGGGATCG  CATGGTATTGGGATCG  CATGGTATTGGGATCG  CACGGTATTGGGATCG  CACGGTATTGGGATCG  CACGGTATTGGGATCG  CACGGTATTGGGATCG  CACGGTATTGGGATCG  CACGGTATTGGGATCG  CACGGTATTGGGATCG |  | Aegilops+Triticum minus A. speltoides and T. aestivum |
| 150 | Schedonorus arundinaceus Schedonorus arundinaceus[C] Schedonorus pratensis  Festuca altissima  Festuca ovina  Lolium multiflorum  Lolium perenne  Dactylis glomerata  Helictochloa hookeri  Deschampsia antarctica  Poa palustris  Phleum alpinum  Briza sp  Puccinellia nuttalliana  Ammophila breviligulata  Agrostis stolonifera  Hierochloe odorata  Anthoxanthum odoratum  Phalaris arundinacea  Torreyochloa  Avena sativa  Trisetum cernuum  Aegilops cylindrica  Aegilops geniculata  Aegilops speltoides  Aegilops tauschii  Triticum aestivum  Triticum monococcum aegilopoides Triticum monococcum (modified) Triticum urartu (modified)  Hordeum jubatum  Hordeum vulgare  Hordeum vulgare spontaneum Secale cereale  Bromus vulgaris  Brachypodium distachyon  Diarrhena obovata  Melica mutica  Melica subulata  Oryzopsis asperifolia Achnatherum hymenoides Ampelodesmos mauritanica Piptochaetium avenaceum  Phaenosperma globosum Brachyelytrum aristosum  Bambusa bambosa | 101412  IRb | IRb | ATTTCCTTGATTTGATCG  ATTTCCTTGATTTGATCG  ATTTCCTTGATTTGCTCG  ATTTCCTTGATTTGATCG  ATTTCCTTGATTTGATCG  ATTTCCTTGATTTGATCG  ATTTCCTTGATTTGATCG  ATTTCCTTGATTTGATCG  ATTTCCTTGATTTGATCG  ATTTCCTTGATTTGATCG  ATTTCCTTGATTTGATCG  ATTTCCTTGATTTGATCG  ATTTCCTTGATTTGATCG  ATTTCCTTGATTTGATCG  ATTTCCTTGATTTGATCG  ATTTCCTTGATTTGATCG  ATTTCCTTGATTTGATCG  ATTTCCTTGATTTGATCG  ATTTCCTTGATTTGATCG  ATTTCCTTGATTTGATCG  ATTTCCTTGATTTGATCG  ATTTCCTTGATTTGATCG  ATTTCCTTGATTTGATCG  ATTTCCTTGATTTGATCG  ATTTCCTTGATTTGATCG  ATTTCCTTGATTTGATCG  ATTTCCTTGATTTGATCG  ATTTCCTTGATTTGATCG  ATTTCCTTGATTTGATCG  ATTTCCTTGATTTGATCG  ATTTCCTTAATTTGATCG  ATTTCCTTAATTTGATCG  ATTTCCTTAATTTGATCG  ATTTCCTTGATTTGATCG  ATTTCCTTGATTTGATCG  ATTTCCTTGATTTGATCG  ATTTCCTTGATTTGATCG  ATTTCCTTGATTTGATCG  ATTTCCTTGATTTGATCG  ATTTCCTTGATTTGATCG  ATTTCCTTGATTTGATCG  ATTTCCTTGATTTGATCG  ATTTCCTTGATTTGATCG  ATTTCCTTGATTTGATCG  ATTTCC-----TTGATCG  ATTTCC-----TTGATCG | ssmp | Homoplasy |
| 151 | Schedonorus arundinaceus Schedonorus arundinaceus[C] Schedonorus pratensis  Festuca altissima  Festuca ovina  Lolium multiflorum  Lolium perenne  Dactylis glomerata  Helictochloa hookeri  Deschampsia antarctica  Poa palustris  Phleum alpinum  Briza sp  Puccinellia nuttalliana  Ammophila breviligulata  Agrostis stolonifera  Hierochloe odorata  Anthoxanthum odoratum  Phalaris arundinacea  Torreyochloa  Avena sativa  Trisetum cernuum  Aegilops cylindrica  Aegilops geniculata  Aegilops speltoides  Aegilops tauschii  Triticum aestivum  Triticum monococcum aegilopoides Triticum monococcum (modified) Triticum urartu (modified)  Hordeum jubatum  Hordeum vulgare  Hordeum vulgare spontaneum Secale cereale  Bromus vulgaris  Brachypodium distachyon  Diarrhena obovata  Melica mutica  Melica subulata  Oryzopsis asperifolia Achnatherum hymenoides Ampelodesmos mauritanica Piptochaetium avenaceum  Phaenosperma globosum Brachyelytrum aristosum  Bambusa bambosa | 101683  IRb | IRb | TCGTTCAGGC----TATATTTAGTC  TCGTTCAGGC----TATATTTAGTC  TCGTTCAGGC----TATATTTAGTC  TCGTTCAGGC----TATATTTAGTC  TCGTTCAGGC----TATATTTAGTC  TCGTTCAGGC----TATATTTAGTC  TCGTTCAGGC----TATATTTAGTC  TCGTTCAGGC----TATATTTAGTC  TCGTTCAGGC----TATATTTAGTC  TCGTTCAGGC----TATATTTAGTC  TCGTTCAGGC----TATATTTAGTC  TCGTTCAGGC----TATATTTAGTC  TCGTTCAGGC----TATATTTAGTC  TCGTTCAGGC----TATATTTAGTC  TCGTTCAGGC----TATATTTAGTC  TCGTTCAGGC----TATATTTAGTC  TCGTTCAGGC----TATATTTAGTC  TCGTTCAGGC----TATATTTAGTC  TCGTTCAGGC----TATATTTAGTC  TCGTTCAGGC----TATATTTAGTC  TCGTTCAGGC----TATATTTAGTC  TCGTTCAGGC----TATATTTAGTC  TCGTTCAGGCTATATATATTTAGTC  TCGTTCAGGCTATATATATTTAGTC  TCGTTCAGGCTATATATATTTAGTC  TCGTTCAGGCTATATATATTTAGTC  TCGTTCAGGCTATATATATTTAGTC  TCGTTCAGGCTATATATATTTAGTC  TCGTTCAGGCTATATATATTTAGTC  TCGTTCAGGCTATATATATTTAGTC  TCGTTCAGGC----TATATTTAGTC  TCGTTCAGGC----TATATTTAGTC  TCGTTCAGGC----TATATTTAGTC  TCGTTCAGGCTATATATATTTAGTC  TCGTTCAGGC----TATATTTAGTC  TCGTTCAGGC----TATATTTAGTC  TCGTTCAGGC----TATATTTAGTC  TCGTTCAGGC----TATATTTAGTC  TCGTTCAGGC----TATATTTAGTC  TCGTTCAGGC----TATATTTAGTC  TCGTTCAGGC----TATATTTAGTC  TCGTTCAGGC----TATATTTAGTC  TCGTTCAGGC----TATATTTAGTC  TCGTTCAGGC----TATATTTAGTC  TCGTTCAGGC----TATATTTAGTC  TCGTTCAGGC----TATATTTAGTC | ssmp | Aegilops+Triticum+Secale |
| 152 | Schedonorus arundinaceus  Schedonorus arundinaceus[C]  Schedonorus pratensis  Festuca altissima  Festuca ovina  Lolium multiflorum  Lolium perenne  Dactylis glomerata  Helictochloa hookeri  Deschampsia antarctica  Poa palustris  Phleum alpinum  Briza sp  Puccinellia nuttalliana  Ammophila breviligulata  Agrostis stolonifera  Hierochloe odorata  Anthoxanthum odoratum  Phalaris arundinacea  Torreyochloa  Avena sativa  Trisetum cernuum  Aegilops cylindrica  Aegilops geniculata  Aegilops speltoides  Aegilops tauschii  Triticum aestivum  Triticum monococcum aegilopoides Triticum monococcum (modified)  Triticum urartu (modified)  Hordeum jubatum  Hordeum vulgare  Hordeum vulgare spontaneum  Secale cereale  Bromus vulgaris  Brachypodium distachyon  Diarrhena obovata  Melica mutica  Melica subulata  Oryzopsis asperifolia  Achnatherum hymenoides Ampelodesmos mauritanica  Piptochaetium avenaceum  Phaenosperma globosum  Brachyelytrum aristosum  Bambusa bambosa | 103367  IRb | IRb | TTACACCTATTCCTAATCCTAATCTGATTACACCTATTCCTAATCCTAAATAGAATG  TTACACGTATTCCTAATCCTAATCTGATTACACCTATTCCTAATCCTAAATAGAATG  TTACACCTATTCCTAATCCTAATCTGATTACACCTATTCCTAATCCTAAATAGAATG  TTACACCTATTCCTAATCCTAATCTGATTACACCTATTCCTAATCCTAAATAGAATG  TTACACC---------------------------TATTCCTAATCCTAAATAGAATG  TTACACCTATTCCTAATCCTAATCTGATTACACCTATTCCTAATCCTAAATAGAATG  TTACACCTATTCCTAATCCTAATCTGATTACACCTATTCCTAATCCTAAATAGAATG  TTACACC---------------------------TATTCCTAATCCTAAATAGAATG  TTACACC---------------------------TATTCCTAATCCTAAATAGAATG  TTACACC---------------------------TATTCCTAATCCTAAATAGAATG  TTACACC---------------------------TATTCCTAATCCTAAATAGAATG  TTACACC---------------------------TATTCCTAATCCTAAATAGAATG  TTACACC---------------------------TATTCCTAATCCTAAATAGAATG  TTACACC---------------------------TATTCCTAATCCTAAATAGAATG  TTACACC---------------------------TATTCCTAATCCTAAATAGAATG  TTACACC---------------------------TATTCCTAATCCTAAATAGAATG  TTACACC---------------------------TATTCCTAATCCTAAATAGAATG  TTACACC---------------------------TATTCCTAATCCTAAATAGAATG  TTACACC---------------------------TATTCCTAATCCTAAATAGAATG  TTACACC---------------------------TATTCCTAATCCTAAATAGAATG  TTACACC---------------------------TATTCCTAATCCTAAATAGAATG  TTACACC---------------------------TATTCCTAATCCTAAATAGAATG  TTACACC---------------------------TATTCCTAATCCTAAATAGAATG  TTACACC---------------------------TATTCCTAATCCTAAATAGAATG  TTACACC---------------------------TATTCCTAATCCTAAATAGAATG  TTACACC---------------------------TATTCCTAATCCTAAATAGAATG  TTACACC---------------------------TATTCCTAATCCTAAATAGAATG  TTACACC---------------------------TATTCCTAATCCTAAATAGAATG  TTACACC---------------------------TATTCCTAATCCTAAATAGAATG  TTACACC---------------------------TATTCCTAATCCTAAATAGAATG  TTACACC---------------------------TATTCCTAATCCTAAATAGAATG  TTACACC---------------------------TATTCCTAATCCTAAATAGAATG  TTACACC---------------------------TATTCCTAATCCTAAATAGAATG  TTACACC---------------------------TATTCCTAATCCTAAATAGAATG  TTACACC---------------------------TATTCCTAATTCTAAATAGAATG  TTACACC---------------------------TATTCCTAATCCTAAATAGAATG  TTACACC---------------------------TATTCCTAATCCTAAATAGAATG  TTACACC---------------------------TATTCCTAATCCTAAATAGAATG  TTACACC---------------------------TATTCCTAATCCTAAATAGAATG  TTACACC---------------------------TATTCCTAATCCTAAATAGAATG  TTACACC---------------------------TATTCCTAATCCTAAATAGAATG  TTACACC---------------------------TATTCCTAATCCTAAATAGAATG  TTACACC---------------------------TATTCCTAATCCTAAATAGAATG  TTACACC---------------------------TATTCCTAATCCTAAATAGAATG  TTACACC---------------------------TATTCCTAATCCTAAATAGAATG  TTACACC---------------------------TATTCCTAATCCTAAATAGAATG |  | Lolium+Schedonorus |
| 153 | Schedonorus arundinaceus  Schedonorus arundinaceus[C]  Schedonorus pratensis  Festuca altissima  Festuca ovina  Lolium multiflorum  Lolium perenne  Dactylis glomerata  Helictochloa hookeri  Deschampsia antarctica  Poa palustris  Phleum alpinum  Briza sp  Puccinellia nuttalliana  Ammophila breviligulata  Agrostis stolonifera  Hierochloe odorata  Anthoxanthum odoratum  Phalaris arundinacea  Torreyochloa  Avena sativa  Trisetum cernuum  Aegilops cylindrica  Aegilops geniculata  Aegilops speltoides  Aegilops tauschii  Triticum aestivum  Triticum monococcum aegilopoides  Triticum monococcum (modified)  Triticum urartu (modified)  Hordeum jubatum  Hordeum vulgare  Hordeum vulgare spontaneum  Secale cereale  Bromus vulgaris  Brachypodium distachyon  Diarrhena obovata  Melica mutica  Melica subulata  Oryzopsis asperifolia  Achnatherum hymenoides  Ampelodesmos mauritanica  Piptochaetium avenaceum  Phaenosperma globosum  Brachyelytrum aristosum  Bambusa bambosa | 103437  IRb | IRb | ATTTCTATGTAA------------------------------------------------------------------------ATGGAA  ATTTCTATGTAA------------------------------------------------------------------------ATGGAA  ATTTCTATGTAA------------------------------------------------------------------------ATGGAA  ATTTCTATGTAA------------------------------------------------------------------------ATGGAA  ATTTCTATGTAA------------------------------------------------------------------------ATGGAA  ATTTCTATGTAA------------------------------------------------------------------------ATGGAA  ATTTCTATGTAA------------------------------------------------------------------------ATGGAA  ATTTCTATGTAAACAGAGTATCCTATTTCCATAGGCTCGAATGA-CCCCTTCTCATAATAAGAATGTGCACGGTCTGGTCCGGTATGGAA  ATTTCTATGTAAACAGAGTATCCTATTTCCATAGGCTCGAATGA-CTCCTTCTCATAATAAGAATGTGCACGGTCTGGTCCGGTATGGAA  ATTTCTATGTAAACAGAATATCCTATTTCCATAGGCTCGAATGA-CCCCTTCTCATAATAAGAATGTGCACGGTCTGGTCCGGTATGGAA  ATTTCTATGTAAACAGAGTAGCCTATTTCCATAGGCTCGAATGA-CCCCTTCTCATAATAAGAATGTGCACGGTCTGGTCCGGTATGGAA  ATTTCTATGTAAACAGAGTATCCTATTTCCATAGGCTCGAATGA-CCCCTTCTCATAATAAGAATGTGCACGGTCTGGTCCGGTATGGAA  ATTTCTATGTAAACAGAGTATCCTATTTCCATAGGATCGAATGA-CCCCTTCTCATAATAAGAATGTGCACGGTCTGGTCCGGTATGGAA  ATTTCTATGTAAACAGAGTATCCTATTTCCATAGGCTCGAATGA-CCCCTTCTCATAATAAGAATGTGCACGGTCTGGTCCGGTATGGAA  ATTTCTATGTAAACAGAGTATCCTATTTCCATAGGCTCGAATGA-CCCCTTCTCATAATAAGAATGTGCACGGTCTGGTCCGGTATGGAA  ATTTCTATGTAAACAGAGTATCCTATTTCCATAGGCTCGAATGA-CCCCTTCTCATAATAAGAATGTGCACGGTCTGGTCCGGTATGGAA  ATTTCTATGTAAACAGAGTATCCTATTTCCATAGGCTCGAATGA-CCCCTTCTCATAATAAGAATGTGCACGGTCTGGTCCGGTATGGAA  ATTTCTATGTAAACAGAGTATCCTATTTCCATAGGCTCAAATGA-CCCCTTCTCATAATAAGAATGTGCACGGTCTGGTCCGGTATGGAA  ATTTCTATGTAAACAGAGTATCCTATTTCCATAGGCTCGAATGA-CCCCTTCTCATAATAAGAATGTGCACGGTCTGGTCCGGTATGGAA  ATTTCTATGTAAACAGAGTATCCTATTTCCATAGGCTCGAATGA-CCCCTTCTCATAATAAGAATGTGCACGGTCTGGTCCGGTATGGAA  ATTTCTATGTAAACAGAGTATCCTATTTCCATAGGCTCGAATGACCCCCTTCTCATAATAAGAATGTGCACGGTCTGGTCCGGTATGGAA  ATTTCTATGTAAACAGAGTATCCTATTTCCATAGGCTCGAATGACCCCCTTCTCATAATAAGAATGTGCACGGTCTGGTCCGGTATGGAA  ATTTCTATGTAAACAGAGTATCCTATTTCCATAGGCTCGAATGA-CCCCTTCTCATAATAAGAATGTGCACGGTCTGGTTCGGTATGGAA  ATTTCTATGTAAACAGAGTATCCTATTTCCATAGGCTCGAATGA-CCCCTTCTCATAATAAGAATGTGCACGGTCTGGTTCGGTATGGAA  ATTTCTATGTAAACAGAGTATCCTATTTCCATAGGCTCGAATGA-CCCCTTCTCATAATAAGAATGTGCACGGTCTGGTTCGGTATGGAA  ATTTCTATGTAAACAGAGTATCCTATTTCCATAGGCTCGAATGA-CCCCTTCTCATAATAAGAATGTGCACGGTCTGGTTCGGTATGGAA  ATTTCTATGTAAACAGAGTATCCTATTTCCATAGGCTCGAATGA-CCCCTTCTCATAATAAGAATGTGCACGGTCTGGTTCGGTATGGAA  ATTTCTATGTAAACAGAGTATCCTATTTCCATAGGCTCGAATGA-CCCCTTCTCATAATAAGAATGTGCGCGGTCTGGTTCGGTATGGAA  ATTTCTATGTAAACAGAGTATCCTATTTCCATAGGCTCGAATGA-CCCCTTCTCATAATAAGAATGTGCACGGTCTGGTTCGGTATGGAA  ATTTCTATGTAAACAGAGTATCCTATTTCCATAGGCTCGAATGA-CCCCTTCTCATAATAAGAATGTGCACGGTCTGGTTCGGTATGGAA  ATTTCTATGTAAACAGAGTATCCTATTTCCATAGGCTCGAATGA-CCCCTTCTCATAATAAGAATGTGCACGGTCTGGTCCGGTATGGAA  ATTTCTATGTAAACAGAGTATCCTATTTCCATAGGCTCGAATGA-CCCCTTCTCATAATAAGAATGTGCACGGTCTGGTCCGGTATGGAA  ATTTCTATGTAAACAGAGTATCCTATTTCCATAGGCTCGAATGA-CCCCTTCTCATAATAAGAATGTGCACGGTCTGGTCCGGTATGGAA  ATTTCTATGTAAACAGAGTATCCTATTTCCATAGGCTCGAATGA-CCCCTTCTCATAATAAGAATGTGCACGGTCTGGTTCGGTATGGAA  ATTTCTATGTAAACAGAGTATCCTATTTCCATAGGCTCGAATGA-CCCCTTCTCATAATAAGAATGTGCACGGTCTGGTCCGGTATGGAA  ATTTCTATGTAAACAGAGTATCCTATTTCCATAGGCTCGAATGA-CCCCTTCTCATAATAAGAATGTGCACGGTCTAGTCCGGTATGGAA  ATTTCTATGTAAACAGAGTATCCTATTTCCATAGGCTCGAATGA-CCCCTTCTCATAATAAGAATGTGCACGGTCTGGTCCGGTATGGAA  ATTTCTATGTAAACAGAGTATCCAATTTCCATAGGCTCGAATGA-CCCCTTCTCATAATAAGAATGTGCACGGTCTGGTCCGGTATGGAA  ATTTCTATGTAAACAGAGTATCCTATTTCCATAGGCTCGAATGA-CCCCTTCTCATAATAAGAATGTGCACGGTCTGGTCCGGTATGGAA  ATTTCTATGTAAACAGAGTATCCTATTTCCATAGGCTCGAATGA-CCCCTTCTCATAATAAGAATGTGCACGGTCTGGTCCGGTATGGAA  ATTTCTATGTAAACAGAGTATCCTATTTCCATAGGCTCGAATGA-CCCCTTCTCATAATAAGAATGTGCACGGTCTGGTCCGGTATGGAA  ATTTCTATGTAAACAGAGTATCCTATTTCCATAGGCTCGAATGA-CCCCTTCTCATAATAAGAATGTGCACGGTCTGGTCCGGTATGGAA  ATTTCTATGTAAACAGAGTATCCTATTTCCATAGGCTCGAATGA-CCCCTTCTCATAATAAGAATGTGCACGGTCTGGTCCGGTATGGAA  ATTTCTATGTAAACAGAGTAGCCTATTTCCATAGGCTCGAATGA-CCCCTTCTCATAATAAGAATGTGCACGGTCTGGTCCGGTATGGAA  ATTTCTATGTAAACAGAGTATCCTATTTCCATAGGCTCGAATGA-CCCCTTCTCATAATAAGAATGTGCACGGTCTGGTCCGGTATGGAA  ATTTCTATGTAAACAGAGTATCCTATTTCCATAGGCTCGAATGA-CCCCTTCTCATAATAAGAATGTGCACGGCCTGGTCCGGTATGGAA |  | Loliinae |
| 154 | Schedonorus arundinaceus Schedonorus arundinaceus[C] Schedonorus pratensis  Festuca altissima  Festuca ovina  Lolium multiflorum  Lolium perenne  Dactylis glomerata  Helictochloa hookeri  Deschampsia antarctica  Poa palustris  Phleum alpinum  Briza sp  Puccinellia nuttalliana  Ammophila breviligulata  Agrostis stolonifera  Hierochloe odorata  Anthoxanthum odoratum  Phalaris arundinacea  Torreyochloa  Avena sativa  Trisetum cernuum  Aegilops cylindrica  Aegilops geniculata  Aegilops speltoides  Aegilops tauschii  Triticum aestivum  Triticum monococcum aegilopoides Triticum monococcum (modified) Triticum urartu (modified)  Hordeum jubatum  Hordeum vulgare  Hordeum vulgare spontaneum Secale cereale  Bromus vulgaris  Brachypodium distachyon  Diarrhena obovata  Melica mutica  Melica subulata  Oryzopsis asperifolia Achnatherum hymenoides Ampelodesmos mauritanica Piptochaetium avenaceum  Phaenosperma globosum Brachyelytrum aristosum  Bambusa bambosa | 103684  irB | IRb | ATCTTGAACTAAGAAAGAAATAGA  ATCTTGAACTAAGAAAGAAATAGA  ATCTTGAACTAAGAAAGAAATAGA  ATCTTGAACTAAGAAAGAAATAGA  ATCTTGAACTAAGAAAGAAATAGA  ATCTTGAACTAAGAAAGAAATAGA  ATCTTGAACTAAGAAAGAAATAGA  ATCTTGAACTAAGAAAGAAATAGA  ATCTTGAACTAAGAAAGAAATAGA  ATCTTGAACTAAGAAAGAAATAGA  ATCTTGAACTAAGAAAGAAATAGA  ATCTTGAACTAAGAAAGAAATAGA  ATCTTGAACT----AAGAAATAGA  ATCTTGAACTAAGAAAGAAATAGA  ATCTTGAACT----AAGAAATAGA  ATCTTGAACT----AAGAAATAGA  ATCTTGAACT----AAGAAATAGA  ATCTTGAACT----AAGAAATAGA  ATCTTGAACT----AAGAAATAGA  ATCTTGAACT----AAGAAATAGA  ATCTTGAACT----AAGAAATAGA  ATCTTGAACT----AAGAAATAGA  ATCTTGAACT----AAGAAATAGA  ATCTTGAACT----AAGAAATAGA  ATCTTGAACT----AAGAAATAGA  ATCTTGAACT----AAGAAATAGA  ATCTTGAACT----AAGAAATAGA  ATCTTGAACT----AAGAAATAGA  ATCTTGAACT----AAGAAATAGA  ATCTTGAACT----AAGAAATAGA  ATCTTGAACT----AAGAAATAGA  ATCTTGAACT----AAGAAATAGA  ATCTTGAACT----AAGAAATAGA  ATCTTGAACT----AAGAAATAGA  ATCTTGAACT----AAGAAATAGA  ATCTTGAACT----AAGAAATAGA  ATCTTGAACT----AAGAAATAGA  ATCTTGAACT----AAGAAATAGA  ATCTTGAACT----AAGAAATAGA  ATCTTGAACT----AAGAAATAGA  ATCTTGAACT----AAGAAATAGA  ATCTTGAACT----AAGAAATAGA  ATCTTGAACT----AAGAAATAGA  ATCTTGAACT----AAGAAATAGA  ATCTTGAACT----AAGAAATAGA  ATCTTGAACT----AAGAAATAGA | ssmp | Poeae clade 2 |
| 155 | Schedonorus arundinaceus Schedonorus arundinaceus[C] Schedonorus pratensis  Festuca altissima  Festuca ovina  Lolium multiflorum  Lolium perenne  Dactylis glomerata  Helictochloa hookeri  Deschampsia antarctica  Poa palustris  Phleum alpinum  Briza sp  Puccinellia nuttalliana  Ammophila breviligulata  Agrostis stolonifera  Hierochloe odorata  Anthoxanthum odoratum  Phalaris arundinacea  Torreyochloa  Avena sativa  Trisetum cernuum  Aegilops cylindrica  Aegilops geniculata  Aegilops speltoides  Aegilops tauschii  Triticum aestivum  Triticum monococcum aegilopoides Triticum monococcum (modified) Triticum urartu (modified)  Hordeum jubatum  Hordeum vulgare  Hordeum vulgare spontaneum Secale cereale  Bromus vulgaris  Brachypodium distachyon  Diarrhena obovata  Melica mutica  Melica subulata  Oryzopsis asperifolia Achnatherum hymenoides Ampelodesmos mauritanica Piptochaetium avenaceum  Phaenosperma globosum Brachyelytrum aristosum  Bambusa bambosa | 106263  IRb | IRb | CATGC----CTAGATTC  CATGC----CTAGATTC  CATGC----CTAGATTC  CATGC----CTAGATTC  CATAC----CTAGATTC  CATGC----CTAGATTC  CATGC----CTAGATTC  CATAC----CTAGATTC  CATAC----CTAGATTC  CATAC----CTAGATTC  CATACCTAGCTAGATTC  CATACCTAGCTAGATTC  CATAC----CTAGATTC  CATAC----CTAGATTC  CATAC----CTAGATTC  CATAC----CTAGATTC  CATAC----CTAGATTC  CATAC----CTAGATTC  CATAC----CTAGATTC  CATAC----CTAGATTC  CATAC----CTAGATTC  CATAC----CTAGATTC  CATAC----ATAGATTC  CATAC----ATAGATTC  CATAC----ATAGATTC  CATAC----ATAGATTC  CATAC----ATAGATTC  CATAC----ATAGATTC  CATAC----ATAGATTC  CATAC----ATAGATTC  CATAC----ATAGATTC  CATAC----ATAGATTC  CATAC----ATAGATTC  CATAC----ATAGATTC  CATAC----ATAGATTC  CATAC----CTAGATTC  CATAC----CTAGATTC  CATAC----CTAGATTC  CATAC----CTAGATTC  CATAC----CTAGATTC  CATAC----CTAGATTC  CATAC----CTAGATTC  CATAC----CTAGATTC  CATAC----CTAGATTC  CATAC----CTGGATTC  CATAC----CTAGATTC | ssmp | Poinae |
| 156 | Schedonorus arundinaceus Schedonorus arundinaceus[C] Schedonorus pratensis  Festuca altissima  Festuca ovina  Lolium multiflorum  Lolium perenne  Dactylis glomerata  Helictochloa hookeri  Deschampsia antarctica  Poa palustris  Phleum alpinum  Briza sp  Puccinellia nuttalliana  Ammophila breviligulata  Agrostis stolonifera  Hierochloe odorata  Anthoxanthum odoratum  Phalaris arundinacea  Torreyochloa  Avena sativa  Trisetum cernuum  Aegilops cylindrica  Aegilops geniculata  Aegilops speltoides  Aegilops tauschii  Triticum aestivum  Triticum monococcum aegilopoides Triticum monococcum (modified) Triticum urartu (modified)  Hordeum jubatum  Hordeum vulgare  Hordeum vulgare spontaneum Secale cereale  Bromus vulgaris  Brachypodium distachyon  Diarrhena obovata  Melica mutica  Melica subulata  Oryzopsis asperifolia Achnatherum hymenoides Ampelodesmos mauritanica Piptochaetium avenaceum  Phaenosperma globosum Brachyelytrum aristosum  Bambusa bambosa | 107084  Rps12 intron 2 | Rps12 intron 2 | TGTAATGG-AAAAAG  TGTAATGG-AAAAAG  TGTAATGG-AAAAAG  TGTAATGG-AAAAAG  TGTAATGG-AAAAAG  TGTAATGG-AAAAAG  TGTAATGG-AAAAAG  TGTAATGG-AAAAAG  TGTAATGG-AAAAAG  TGTAATGG-AAAAAG  TGTAATGG-AAAAAG  TGTAATGG-AAAAAG  TGTAATGG-AAAAAG  TGTAATGG-AAAAAG  TGTAATGG-AAAAAG  TGTAATGG-AAAAAG  TGTAATGG-AAAAAG  TGTAATGG-AAAAAG  TGTAATGG-AAAAAG  TGTAATGG-AAAAAG  TGTAATGG-AAAAAG  TGTAATGG-AAAAAG  TGTAATGG-AAAAAG  TGTAATGG-AAAAAG  TGTAATGG-AAAAAG  TGTAATGG-AAAAAG  TGTAATGG-AAAAAG  TGTAATGG-AAAAAG  TGTAATGG-AAAAAG  TGTAATGG-AAAAAG  TGTAATGG-AAAAAG  TGTAATGG-AAAAAG  TGTAATGG-AAAAAG  TGTAATGG-AAAAAG  TGTAATGG-AAAAAG  TGTAATGG-AAAAAG  TGTAATGG-AAAAAG  TG-------TAAAAG  TG-------TAAAAG  TGTAATGG-AAAAAG  TGTAATGG-AAAAAG  TGTAATGG-AAAAAG  TGTAATGG-AAAAAG  TGTAATGG-AAAAAG  TGTAATGGAAAAAAG  TGTAATGG-AAAAAG |  | Melica |
| 157 | Schedonorus arundinaceus Schedonorus arundinaceus[C] Schedonorus pratensis  Festuca altissima  Festuca ovina  Lolium multiflorum  Lolium perenne  Dactylis glomerata  Helictochloa hookeri  Deschampsia antarctica  Poa palustris  Phleum alpinum  Briza sp  Puccinellia nuttalliana  Ammophila breviligulata  Agrostis stolonifera  Hierochloe odorata  Anthoxanthum odoratum  Phalaris arundinacea  Torreyochloa  Avena sativa  Trisetum cernuum  Aegilops cylindrica  Aegilops geniculata  Aegilops speltoides  Aegilops tauschii  Triticum aestivum  Triticum monococcum aegilopoides Triticum monococcum (modified) Triticum urartu (modified)  Hordeum jubatum  Hordeum vulgare  Hordeum vulgare spontaneum Secale cereale  Bromus vulgaris  Brachypodium distachyon  Diarrhena obovata  Melica mutica  Melica subulata  Oryzopsis asperifolia Achnatherum hymenoides Ampelodesmos mauritanica Piptochaetium avenaceum  Phaenosperma globosum Brachyelytrum aristosum  Bambusa bambosa | 108566  IRb | IRb | AGTAGTACTATTCTATTAGT  AGTAGTACTATTCTATTAGT  AGTAGTACTATTCTATTAGT  AGTAGTACTATTCTATTAGT  AGTAGTACTATTCTATTAGT  AGTAGTACTATTCTATTAGT  AGTAGTACTATTCTATTAGT  AGTAGTACTATTCTATTAGT  AGTAGTACTATTCTATTAGT  AGTAGTACTATTCTATTAGT  AGTAGTACTATTCTATTAGT  AGTAGTACTATTCTATTAGT  AGTAGTACTATTCTATTAGT  AGTAGTACTATTCTATTAGT  AGTAGTACTATTCTATTAGT  AGTAGTACTATTCTATTAGT  AGTAGTACTATTCTATTAGT  AGTAGTACTATTCTATTAGT  AGTAGTACTATTCTATTAGT  AGTAGTACTATTCTATTAGT  AGTAGTACTATTCTATTAGT  AGTAGTACTATTCTATTAGT  AGTAGTA-----CTATTAGT  AGTAGTA-----CTATTAGT  AGTAGTA-----CTATTAGT  AGTAGTA-----CTATTAGT  AGTAGTA-----CTATTAGT  AGTAGTA-----CTATTAGT  AGTAGTA-----CTATTAGT  AGTAGTA-----CTATTAGT  AGTAGTACTATTCTATTAGT  AGTAGTACTATTCTATTAGT  AGTAGTACTATTCTATTAGT  AGTAGTA-----CTATTAGT  AGTAGTACTATTCTATTAGT  AGTAGTACTATTCTATTAGT  AGTAGTACTATTCTATTAGT  AGTAGTACTATTCTATTAGT  AGTAGTACTATTCTATTAGT  AGTAGTACTATTCTATTAGT  AGTAGTACTATTCTATTAGT  AGTAGTACTATTCTATTAGT  AGTAGTACTATTCTATTAGT  AGTAGTACTATTCTATTAGT  AGTAGTACTATTCTATTAGT  AGTAGTACTATTCTATTAGT | ssmp | Aegilops+Triticum+Secale |
| 158 | Schedonorus arundinaceus  Schedonorus arundinaceus[C]  Schedonorus pratensis  Festuca altissima  Festuca ovina  Lolium multiflorum  Lolium perenne  Dactylis glomerata  Helictochloa hookeri  Deschampsia antarctica  Poa palustris  Phleum alpinum  Briza sp  Puccinellia nuttalliana  Ammophila breviligulata  Agrostis stolonifera  Hierochloe odorata  Anthoxanthum odoratum  Phalaris arundinacea  Torreyochloa  Avena sativa  Trisetum cernuum  Aegilops cylindrica  Aegilops geniculata  Aegilops speltoides  Aegilops tauschii  Triticum aestivum  Triticum monococcum aegilopoides  Triticum monococcum (modified)  Triticum urartu (modified)  Hordeum jubatum  Hordeum vulgare  Hordeum vulgare spontaneum  Secale cereale  Bromus vulgaris  Brachypodium distachyon  Diarrhena obovata  Melica mutica  Melica subulata  Oryzopsis asperifolia  Achnatherum hymenoides  Ampelodesmos mauritanica  Piptochaetium avenaceum  Phaenosperma globosum  Brachyelytrum aristosum  Bambusa bambosa | 108581  irB | irB | TTAGTT----------------------------------------------------------------------------ACCGAGG  TTAGTT----------------------------------------------------------------------------ACCGAGG  TTAGTT----------------------------------------------------------------------------ACCGAGG  TTAGTT----------------------------------------------------------------------------ACCGAGG  TTAGTT----------------------------------------------------------------------------ACCGAGG  TTAGTT----------------------------------------------------------------------------ACCGAGG  TTAGTT----------------------------------------------------------------------------ACCGAGG  TTAGTT----------------------------------------------------------------------------ACCGAGG  TTAGTT----------------------------------------------------------------------------ACCGAGG  TTAGTT----------------------------------------------------------------------------ACCGAGG  TTAGTT----------------------------------------------------------------------------ACCGAGG  TTAGTT----------------------------------------------------------------------------ACCGAGG  TTAGTTACCGATCCCGGCTCTGTGAGTTCTTTCTTCCGTGATGAACTGTCGGCACCAGTCCTACATTTTTGTT-CTCTGTGGACCGAGG  TTAGTT----------------------------------------------------------------------------ACCGAGG  TTAGTTACCGATCCCGGCTCTGTGAGTTCTTTCTTCCGTGATGAACTGTCGGCACCAGTCCTACATTTTTGTT-CTCTGTGGACCGAGG  TTAGTTACCGATCCCGGCTCTGTGAGTTCTTTCTTCCGTGATGAACTGTCGGCACCAGTCCTACATTTTTGTT-CTCTGTGGACCGAGG  TTAGTTACCGATCCCGGCTCTGTGAGTTCTTTCTTCCGTGATGAACTGTCGGCACCAGTCCTACATTTTTGTT-CTCTGTGGACCGAGG  TTAGTTACCGATCCCGGCTCTGTGAGTTCTTTCTTCCGTGATGAACTGTCGGCACCAGTCCTACATTTTTGTT-CTCTGTGGACCGAGG  TTAGTTACCGATCCCGGCTCTGTGAGTTCTTTCTTCCGTGATGAACTGTCGGCACCAGTCCTACATTTTTGTT-CTCTGTGGACCGAGG  TTAGTTACCGATCCCGGCTCTGTGAGTTCTTTCTTCCGTGATGAACTGTCGGCACCAGTCCTACATTTTTGTT-CTCTGTGGACCGAGG  TTAGTTACCGATCCCGGCTCTGTGAGTTCTTTCTTCCGTGATGAACTGTCGGCACCAGTCCTACATTTTTGTT-CTCTGTGGACCGAGG  TTAGTTACCGATCCCGGCTCTGTGAGTTCTTTCTTCCGTGATGAACTGTCGGCACCAGTCCTACATTTTTGTT-CTCTGTGGACCGAGG  TTAGTTACCGATCCCGGCTCTGTGAGTTCTTTCTTCCGTGATGAACTGTCGGCACCAGTCCTACATTTTTTT--CTCTGTGGACCGAGG  TTAGTTACCGATCCCGGCTCTGTGAGTTCTTTCTTCCGTGATGAACTGTCGGCACCAGTCCTACATTTTTTT--CTCTGTGGACCGAGG  TTAGTTACCGATCCCGGCTCTGTGAGTTCTTTCTTCCGTGATGAACTGTCGGCACCAGTCCTACATTTTTTT--CTCTGTGGACCGAGG  TTAGTTACCGATCCCGGCTCTGTGAGTTCTTTCTTCCGTGATGAACTGTCGGCACCAGTCCTACATTTTTTT--CTCTGTGGACCGAGG  TTAGTTACCGATCCCGGCTCTGTGAGTTCTTTCTTCCGTGATGAACTGTCGGCACCAGTCCTACATTTTTTT--CTCTGTGGACCGAGG  TTAGTTACCGATCCCGGCTCTGTGAGTTCTTTCTTCCGTGATGAACTGTCGGCACCAGTCCTACATTTTTTT--CTCTGTGGACCGAGG  TTAGTTACCGATCCCGGCTCTGTGAGTTCTTTCTTCCGTGATGAACTGTCGGCACCAGTCCTACATTTTTTT--CTCTGTGGACCGAGG  TTAGTTACCGATCCCGGCTCTGTGAGTTCTTTCTTCCGTGATGAACTGTCGGCACCAGTCCTACATTTTTTT--CTCTGTGGACCGAGG  TTAGTTACCGATCCCGGCTCTGTGAGTTCTTTCTTCCGTGATGAACTGTCGGCACCAGTCCTACATTTTTTT--CTCTGTGGACCGAGG  TTAGTTACCGATCCCGGCTCTGTGAGTTCTTTCTTCCGTGATGAACTGTCGGCACCAGTCCTACATTTTTTT--CTCTGTGGACCGAGG  TTAGTTACCGATCCCGGCTCTGTGAGTTCTTTCTTCCGTGATGAACTGTCGGCACCAGTCCTACATTTTTTT--CTCTGTGGACCGAGG  TTAGTTACCGATCCCGGCTCTGTGAGTTCTTTCTTCCGTGATGAACTGTCGGCACCAGTCCTACATTTTTTT--CTCTGTGGACCGAGG  TTAGTTACCGATCCCGGCTCTGTGAGTTCTTTCTTCCGTGATGAACTGTCGGCACCAGTCCTACATTTTTTT--CTCTGTGGACCGAGG  TTAGTTACCGATCCCGGCTCTGTGAGTTCTTTCTTCCGTGATGAACTGTCGGCACCAGTCCTACATTTTTTT--CTCTGTGGACCGAGG  TTAGTTACCGATCCCGGCTCTGTGAGTTCTTTCTTCCGTGATGAACTGTCGGCACCAGTCCTACATTTTTTTT-CTCTGTGGACCGAGG  TTAGTTACCGATCCCGGCTCTGTGAGTTCTTTCTTCCGTGATGAACTGTCGGCACCAGTCCTACATTTTTTTT-CTCTGTGGACCGAGG  TTAGTTACCGATCCCGGCTCTGTGAGTTCTTTCTTCCGTGATGAACTGTCGGCACCAGTCCTACATTTTTTTT-CTCTGTGGACCGAGG  TTAGTTACCGATCCCGGCTCTGTGAGTTCTTTCTTCCGTGATGAACTGTTGGCACCAGTCCTACATTTTTTTT-CTCTGTGGACCGAGG  TTAGTTACCGATCCCGGCTCTGTGAGTTCTTTCTTCCGTGATGAACTGTTGGCACCAGTCCTACATTTTTTTT-CTCTGTGGACCGAGG  TTAGTTACCGATCCCGGCTCTGTGAGTTCTTTCTTCCGTGATGAACTGTTGGCACCAGTCCTACATTTTTTTT-CTCTGTGGACCGAGG  TTAGTTACCGATCCCGGCTCTGTGAGTTCTTTCTTCCGTGATGAACTGTTGGCACCAGTCCTACATTTTTTTT-CTCTGTGGACCGAGG  TTAGTTAGCGATCCCGGCTCTGTGAGTTCTTTCTTCCGTGATGAACTGTCGGCACCAGTCCTACATTTTTTTTCCTCTATGGACCGAGG  TTAGTTAGCGATCCCGGCTCTGTGAGTTCTTTCTTCCGTGATGAACTGTTGGCACCAGTCCTACATTTTTTTT-CTCTGTGGACCGAGG  TTAGTTAGCGATCCCGGCTCTGTGAGTTCTTTCTTCCGTGATGAACTGTCGGCACCAGTCCTACATTTTTT---CTCTGTGGACCGAGG |  | Poeae clade 2 |
| 159 | Schedonorus arundinaceus Schedonorus arundinaceus[C] Schedonorus pratensis  Festuca altissima  Festuca ovina  Lolium multiflorum  Lolium perenne  Dactylis glomerata  Helictochloa hookeri  Deschampsia antarctica  Poa palustris  Phleum alpinum  Briza sp  Puccinellia nuttalliana  Ammophila breviligulata  Agrostis stolonifera  Hierochloe odorata  Anthoxanthum odoratum  Phalaris arundinacea  Torreyochloa  Avena sativa  Trisetum cernuum  Aegilops cylindrica  Aegilops geniculata  Aegilops speltoides  Aegilops tauschii  Triticum aestivum  Triticum monococcum aegilopoides Triticum monococcum (modified) Triticum urartu (modified)  Hordeum jubatum  Hordeum vulgare  Hordeum vulgare spontaneum Secale cereale  Bromus vulgaris  Brachypodium distachyon  Diarrhena obovata  Melica mutica  Melica subulata  Oryzopsis asperifolia Achnatherum hymenoides Ampelodesmos mauritanica Piptochaetium avenaceum  Phaenosperma globosum Brachyelytrum aristosum  Bambusa bambosa | 108778  IRb | irB | ATATCGATCTGATCCG  ATATCGATCTGATCCG  ATATCGATCTGATCCG  ATATCGATCTGATCCG  ATATCGATCCGATCCG  ATATCGATCTGATCCG  ATATCGATCTGATCCG  ATATCGATCCGATCCG  ATATCGATCCGATCCG  ATATCGATCCGATCCG  ATAT-----CGATCCG  ATATCGATCCGATCCG  ATATCGATCCGATCCG  ATAT-----CGATCCG  ATATCGATCCGATCCG  CTATCGATCCGATCCG  ATATCAATCCGATCCG  ATATCAATCCGATCCG  ATATCGATCCGATCCG  ATATCGATCCGATCCG  ATATCGATCCGATCCG  ATATCGATCCGATCCG  ATAT-----CGATCCG  ATAT-----CGATCCG  ATAT-----CGATCCG  ATAT-----CGATCCG  ATAT-----CGATCCG  ATAT-----CGATCCG  ATAT-----CGATCCG  ATAT-----CGATCCG  ATAT-----CGATCCG  ATAT-----CGATCCG  ATAT-----CGATCCG  ATAT-----CGATCCG  ATAT-----CGATCCG  ATATCGATCCGATCCG  ATATCGATCCGATCCG  ATATCGATCCGATCCG  ATATCGATCCGATCCG  ATATCGATCCGATCCG  ATATCGATCCGATCCG  ATATCGATCCGATCCG  ATATCGATCCGATCCG  ATAT-----CGATCCG  ATAT-----CGATCCG  ATAT-----CGATCCG | Ssmp | Homoplasy |
| 160 | Schedonorus arundinaceus Schedonorus arundinaceus[C] Schedonorus pratensis  Festuca altissima  Festuca ovina  Lolium multiflorum  Lolium perenne  Dactylis glomerata  Helictochloa hookeri  Deschampsia antarctica  Poa palustris  Phleum alpinum  Briza sp  Puccinellia nuttalliana  Ammophila breviligulata  Agrostis stolonifera  Hierochloe odorata  Anthoxanthum odoratum  Phalaris arundinacea  Torreyochloa  Avena sativa  Trisetum cernuum  Aegilops cylindrica  Aegilops geniculata  Aegilops speltoides  Aegilops tauschii  Triticum aestivum  Triticum monococcum aegilopoides Triticum monococcum (modified) Triticum urartu (modified)  Hordeum jubatum  Hordeum vulgare  Hordeum vulgare spontaneum Secale cereale  Bromus vulgaris  Brachypodium distachyon  Diarrhena obovata  Melica mutica  Melica subulata  Oryzopsis asperifolia Achnatherum hymenoides Ampelodesmos mauritanica Piptochaetium avenaceum  Phaenosperma globosum Brachyelytrum aristosum  Bambusa bambosa | 108978  IRb | irB | ATTTGTCC---TGATGTTT  ATTTGTCC---TGATGTTT  ATTTGTCC---TGATGTTT  ATTTGTCC---TGATGTTT  ATTTGTCC---TGATGTTT  ATTTGTCC---TGATGTTT  ATTTGTCC---TGATGTTT  ATTTGTCC---TGATGTTT  ATTTGTCC---TGATGTTT  ATTTGTCC---TGATGTTT  ATTTGTCC---TGATGTTT  ATTTGTCC---TGATGTTT  ATTTGTCC---TGATGTTT  ATTTGTCC---TGATGTTT  ATTTGTCC---TGATGTTT  ATTTGTCC---TGATGTTT  ATTTGTCC---TGATGTTT  ATTTGTCC---TGATGTTT  ATTTGTCC---TGATGTTT  ATTTGTCC---TGATGTTT  ATTTGTCC---TGATGTTT  ATTTGTCC---TGATGTTT  ATTTGTCC---TCATGTTT  ATTTGTCC---TCATGTTT  ATTTGTCC---TCATGTTT  ATTTGTCC---TCATGTTT  ATTTGTCC---TCATGTTT  ATTTGTCC---TCATGTTT  ATTTGTCC---TCATGTTT  ATTTGTCC---TCATGTTT  ATTTGTCC---TCATGTTT  ATTTGTCC---TCATGTTT  ATTTGTCC---TCATGTTT  ATTTGTCC---TCATGTTT  ATTTGTCC---TCATGTTT  ATTTGTCC---TCATGTTT  ATTTGTCC---TCATGTTT  ATTTGTCCATTTCATGTTT  ATTTGTCCATTTCATGTTT  ATTTGTCCATTTCATGTTT  ATTTGTCCATTTCATGTTT  ATTTGTCCATTTCATGTTT  ATTTGTCCATTTCATGTTT  ATTTGTCCATTTCATGTTT  ATTTGTCCATTTCATGTTT  ATTTGTCCATTTCATGTTT |  | Diarrheneae+Brachypodieae+Bromeae+Poeae+Triticeae |
| 161 | Schedonorus arundinaceus Schedonorus arundinaceus[C] Schedonorus pratensis  Festuca altissima  Festuca ovina  Lolium multiflorum  Lolium perenne  Dactylis glomerata  Helictochloa hookeri  Deschampsia antarctica  Poa palustris  Phleum alpinum  Briza sp  Puccinellia nuttalliana  Ammophila breviligulata  Agrostis stolonifera  Hierochloe odorata  Anthoxanthum odoratum  Phalaris arundinacea  Torreyochloa  Avena sativa  Trisetum cernuum  Aegilops cylindrica  Aegilops geniculata  Aegilops speltoides  Aegilops tauschii  Triticum aestivum  Triticum monococcum aegilopoides Triticum monococcum (modified) Triticum urartu (modified)  Hordeum jubatum  Hordeum vulgare  Hordeum vulgare spontaneum Secale cereale  Bromus vulgaris  Brachypodium distachyon  Diarrhena obovata  Melica mutica  Melica subulata  Oryzopsis asperifolia Achnatherum hymenoides Ampelodesmos mauritanica Piptochaetium avenaceum  Phaenosperma globosum Brachyelytrum aristosum  Bambusa bambosa | 108999  irB | irb | AGGTCT-----CAAAAAAA  AGGTCT-----CAAAAAAA  AGGTCT-----CAAAAAAA  AGGTCT-----CAAAAAAA  AGGTCT-----CAAAAAAA  AGGTCT-----CAAAAAAA  AGGTCT-----CAAAAAAA  AGGTCT-----CAAAAAAA  AGGTCT-----CAAAAAAA  AGGTCT-----CAAAAAAA  AGGTCT-----CAAAAAAA  AGGTCT-----CAAAAAAA  AGGTCT-----CAAAAAAA  AGGTCT-----CAAAAAAA  AGGTCT-----CAAAAAAA  AGGTCT-----CAAAAAAA  AGGTCT-----AAAAAAAA  AGGTCT----AAAAAAAAA  AGGTCT-----CAAAAAAA  AGGTCT-----CAAAAAAA  AGGTCT-----CAAAAAAA  AGGTCT-----CAAAAAAA  AGGTCT-----CAAGAAAA  AGGTCT-----CAAGAAAA  AGGTCT-----CAAGAAAA  AGGTCT-----CAAGAAAA  AGGTCT-----CAAGAAAA  AGGTCT-----CAAGAAAA  AGGTCT-----CAAGAAAA  AGGTCT-----CAAGAAAA  AGGTCT-----CAAGAAAA  AGGTCT-----CAAGAAAA  AGGTCT-----CAAGAAAA  AGGTCT-----CAAGAAAA  AGGTCT-----CAAAAAAA  AGGTCT-----CAAAAAAA  AGGTCT-----CAAGAAAA  GGGTCTCAAAACAAAAAAA  GGGTCTCAAAACAAAAAAA  AGGTCT-----CAAAAAAA  AGGTCT-----CAAAAAAA  AGGTCT-----CAAAAAAA  AGGTCT-----CAAAAAAA  AGGTCT-----CAAAAAAA  AGGTCT-----CAAAAAAA  AGGTCT-----CAAAAAAA | Ssmp | Melica |
| 162 | Schedonorus arundinaceus Schedonorus arundinaceus[C] Schedonorus pratensis  Festuca altissima  Festuca ovina  Lolium multiflorum  Lolium perenne  Dactylis glomerata  Helictochloa hookeri  Deschampsia antarctica  Poa palustris  Phleum alpinum  Briza sp  Puccinellia nuttalliana  Ammophila breviligulata  Agrostis stolonifera  Hierochloe odorata  Anthoxanthum odoratum  Phalaris arundinacea  Torreyochloa  Avena sativa  Trisetum cernuum  Aegilops cylindrica  Aegilops geniculata  Aegilops speltoides  Aegilops tauschii  Triticum aestivum  Triticum monococcum aegilopoides Triticum monococcum (modified) Triticum urartu (modified)  Hordeum jubatum  Hordeum vulgare  Hordeum vulgare spontaneum Secale cereale  Bromus vulgaris  Brachypodium distachyon  Diarrhena obovata  Melica mutica  Melica subulata  Oryzopsis asperifolia Achnatherum hymenoides Ampelodesmos mauritanica Piptochaetium avenaceum  Phaenosperma globosum Brachyelytrum aristosum  Bambusa bambosa | 111662  IRb IGS |  | GCCTCCCTTTCTTTGGGAGG  GCCTCCCTTTCTTTGGGAGG  GCCTCCCTTTCTTTGGGAGG  GCCTCCCTTTCTTTGGGAGG  GCCTCCCTTTCTTTGGGAGG  GCCTCCCTTTCTTTGGGAGG  GCCTCCCTTTCTTTGGGAGG  GCCTCCCTTTCTTTGGGAGG  GCCTCCCTTTCTTTGGGAGG  GCCTCCCTTTCTTTGGGAGG  GCCTCCCTTTCTTTGGGAGG  GCCTCCCTTTCTTTGGGAGG  GCCTCC----CTTTGGGAGG  GCCTCCCTTTCTTTGGGAGG  GCCTCC----CTTTGGGAGG  GCCTCC----CTTTGGGAGG  GCCTCC----CTTTGGGAGG  GCCTCC----CAAAGGGAGG  GCCTCC----CAAAGGGAGG  GCCTCC----CTTTGGGAGG  GCCTCC----CTTTGGGAGG  GCCTCC----CTTTGGGAGG  GCCTCC----CTTTGGGAGG  GCCTCC----CTTTGGGAGG  GCCTCC----CTTTGGGAGG  GCCTCC----CTTTGGGAGG  GCCTCC----CTTTGGGAGG  GCCTCC----CTTTGGGAGG  GCCTCC----CTTTGGGAGG  GCCTCC----CTTTGGGAGG  GCCTCC----CAAAGGGAGG  GCCTCC----CTTTGGGAGG  GCCTCC----CTTTGGGAGG  GCCTCC----CAAAGGGAGG  GCCTCC----CTTTGGGAGG  GCCTCC----CTTTGGGAGG  GCCTCC----CTTTGGGAGG  GCCTCC----CAAAGGGAGG  GCCTCC----CTTTGGGAGG  GCCTCC----CTTTGGGAGG  GCCTCC----CTTTGGGAGG  GCCTCC----CAAAGGGAGG  GCCTCC----CTTTGGGAGG  GCCTCC----CAAAGGGAGG  GCCTCC----CTTTGGGAGG  GCCTCC----CTTTGGGAGG | ssmp | Poeae clade 2 |
| 163 | Schedonorus arundinaceus  Schedonorus arundinaceus[C  ] Schedonorus pratensis  Festuca altissima  Festuca ovina  Lolium multiflorum  Lolium perenne  Dactylis glomerata  Helictochloa hookeri  Deschampsia antarctica  Poa palustris  Phleum alpinum  Briza sp  Puccinellia nuttalliana  Ammophila breviligulata  Agrostis stolonifera  Hierochloe odorata  Anthoxanthum odoratum  Phalaris arundinacea  Torreyochloa  Avena sativa  Trisetum cernuum  Aegilops cylindrica  Aegilops geniculata  Aegilops speltoides  Aegilops tauschii  Triticum aestivum  Triticum monococcum aegilopoides  Triticum monococcum (modified)  Triticum urartu (modified)  Hordeum jubatum  Hordeum vulgare  Hordeum vulgare spontaneum  Secale cereale  Bromus vulgaris  Brachypodium distachyon  Diarrhena obovata  Melica mutica  Melica subulata  Oryzopsis asperifolia  Achnatherum hymenoides  Ampelodesmos mauritanica  Piptochaetium avenaceum  Phaenosperma globosum  Brachyelytrum aristosum  Bambusa bambosa | 112258 | trnL-GAU intron 1 | TCTTTTTCCTT-------------------------------------------------------------------------------------------------------------------------------------------------AAATGGGAGCAGAGCAGGTTTGAAAAAGGATCTT  TCTTTTTCCTT-------------------------------------------------------------------------------------------------------------------------------------------------AAATGGGAGCAGAGCAGGTTTGAAAAAGGATCTT  TCTTTTTCCTT-------------------------------------------------------------------------------------------------------------------------------------------------AAATGGGAGCAGAGCAGGTTTGAAAAAGGATCTT  TCTTTTTCCTT-------------------------------------------------------------------------------------------------------------------------------------------------AAATGGGAGCAGAGCAGGTTTGAAAAAGGATCTT  TCTTTTTCCTT-------------------------------------------------------------------------------------------------------------------------------------------------AAATGGGAGCAGAGCAGGTTTGAAAAAGGATCTT  TCTTTTTCCTT-------------------------------------------------------------------------------------------------------------------------------------------------AAATGGGAGCAGAGCAGGTTTGAAAAAGGATCTT  TCTTTTTCCTT-------------------------------------------------------------------------------------------------------------------------------------------------AAATGGGAGCAGAGCAGGTTTGAAAAAGGATCTT  TCTTTTTCCTT-------------------------------------------------------------------------------------------------------------------------------------------------AAATGGGAGCAGAGCAGGTTTGAAAAAGGATCTT  TCTTTTTCCTT-------------------------------------------------------------------------------------------------------------------------------------------------AAATGGGAGCAGAGCAGGTTTGAAAAAGGATCTT  TCTTTTTCCTT-------------------------------------------------------------------------------------------------------------------------------------------------AAATGGGAGCAGAGCAGGTTTGAAAAAGGATCTT  TCTTTTTCCTT-------------------------------------------------------------------------------------------------------------------------------------------------AAATGGGAGCGGAGCAGGTTTGAAAAAGGATCTT  TCTTTTTCCTT-------------------------------------------------------------------------------------------------------------------------------------------------AAATGGGAGCGGAGCAGGTTTGAAAAAGGATCTT  TCTTTTTCCTT-------------------------------------------------------------------------------------------------------------------------------------------------AAATGGGAGCAGAGCAGGTTTGAAAAAGGATCTT  TCTTTTTCCTT-------------------------------------------------------------------------------------------------------------------------------------------------AAATGGGAGCGGAGCAGGTTTGAAAAAGGATCTT  TCTTTTTCCTT-------------------------------------------------------------------------------------------------------------------------------------------------AAATGGGAGCAGAGCAGGTTTGAAAAAGGATCTT  TCTTTTTCCTT-------------------------------------------------------------------------------------------------------------------------------------------------AAATGGGAGCAGAGCAGGTTTGAAAAAGGATCTT  TCTTTTTCCTT-------------------------------------------------------------------------------------------------------------------------------------------------AAATGGGAGCAGAGCAGGTTTGAAAAAGGATCTT  TCTTTTTCCTT-------------------------------------------------------------------------------------------------------------------------------------------------AAATGGGAGCAGAGCAGGTTTGAAAAAGGATCTT  TCTTTTTCCTT-------------------------------------------------------------------------------------------------------------------------------------------------AAATGGGAGCAGAGCAGGTTTGAAAAAGGATCTT  TCTTTTTCCTT-------------------------------------------------------------------------------------------------------------------------------------------------AAATGGGAGCAGAGCAGGTTTGAAAAAGGATCTT  TCTTTTTCCTT-------------------------------------------------------------------------------------------------------------------------------------------------AAATGGGAGCAGAGCAGGTTTGAAAAAGGATCTT  TCTTTTTCCTT-------------------------------------------------------------------------------------------------------------------------------------------------AAATGGGAGCAGAGCAGGTTTGAAAAAGGATCTT  TCTTTTTCCTT-------------------------------------------------------------------------------------------------------------------------------------------------AAATGGGAGCAGAGCAGGTTTGAAAAAGGATCTT  TCTTTTTCCTT-------------------------------------------------------------------------------------------------------------------------------------------------AAATGGGAGCAGAGCAGGTTTGAAAAAGGATCTT  TCTTTTTCCTT-------------------------------------------------------------------------------------------------------------------------------------------------AAATGGGAGCAGAGCAGGTTTGAAAAAGGATCTT  TCTTTTTCCTT-------------------------------------------------------------------------------------------------------------------------------------------------AAATGGGAGCAGAGCAGGTTTGAAAAAGGATCTT  TCTTTTTCCTT-------------------------------------------------------------------------------------------------------------------------------------------------AAATGGGAGCAGAGCAGGTTTGAAAAAGGATCTT  TCTTTTTCCTT-------------------------------------------------------------------------------------------------------------------------------------------------AAATGGGAGCAGAGCAGGTTTGAAAAAGGATCTT  TCTTTTTCCTT-------------------------------------------------------------------------------------------------------------------------------------------------AAATGGGAGCAGAGCAGGTTTGAAAAAGGATCTT  TCTTTTTCCTT-------------------------------------------------------------------------------------------------------------------------------------------------AAATGGGAGCAGAGCAGGTTTGAAAAAGGATCTT  TATTTTTCCTT-------------------------------------------------------------------------------------------------------------------------------------------------AAATGGGAGCAGAGCAGGTTTGAAAAAGGATCTT  TATTTTTCCTT-------------------------------------------------------------------------------------------------------------------------------------------------AAATGGGAGCAGAGCAGGTTTGAAAAAGGATCTT  TATTTTTCCTT-------------------------------------------------------------------------------------------------------------------------------------------------AAATGGGAGCAGAGCAGGTTTGAAAAAGGATCTT  TCTTTTTCCTT-------------------------------------------------------------------------------------------------------------------------------------------------AAATGGGAGCAGAGCAGGTTTGAAAAAGGATCTT  TATTTTTCCTT-------------------------------------------------------------------------------------------------------------------------------------------------AAATGGGAGCAGAGCAGGTTTGAAAAAGGATCTT  TCTTTTTCCTT-------------------------------------------------------------------------------------------------------------------------------------------------AAATGGGAGCAGAGCAGGTTTGAAAAAGGATCTT  TCTTTTTCCTT-------------------------------------------------------------------------------------------------------------------------------------------------AAATGGGAGCAGAGCAGGTTTGAAAAAGGATCTT  TCTTTTTCCTT-------------------------------------------------------------------------------------------------------------------------------------------------AAATGGGAGCAGAGCAGGTTTGAAAAAGGATCTT  TCTCTTTCCTT-------------------------------------------------------------------------------------------------------------------------------------------------AAATGGGAGCAGAGCAGGTTTGAAAAAGGATCTT  TCTTTTTCCTT-------------------------------------------------------------------------------------------------------------------------------------------------AAATGGGAGCAGAGCAGGTTTGAAAAAGGATCTT  TCTTTTTCCTT-------------------------------------------------------------------------------------------------------------------------------------------------AAATGGGAGCAGAGCAGGTTTGAAAAAGGATCTT  TCTTTTTCCTT-------------------------------------------------------------------------------------------------------------------------------------------------AAATGGGAGCAGAGCAGGTTTGAAAAAGGATCTT  TCTTTTTCCTT-------------------------------------------------------------------------------------------------------------------------------------------------AAATGGGAGCAGAGCAGGTTTGAAAAAGGATCTT  TCTTTTTCCTT-------------------------------------------------------------------------------------------------------------------------------------------------AAATGGGAGCAGAGCAGGTTTGAAAAAGGATCTT  TCTTTTTCCTTTCCGCAGGGACCAGGAGATTGGATCTAGCCATAAGAGGAATGCTTGGTATAAATAAGCCACTTCTTGGTCTTCGACCCCCTAAGTCACTACGAGCGCCCCCGATCAGTGCAATGGGATGTGGCTATTTATCTATCTCTTGAATCGAAATGGGAGCAGAGCAGGTTTGAAAAAGGATCTT  TCTTTTTCCTTTCTGCAGGGACCAGGAGATTGGATCTAGCCATAAGAGGAATGCTTGGTATAAATAAGCCGCTTATTGGTCTTCGACCCCCTAAGTCACTACGAGCGCCCCCGATCAGTGCAATGGGATGTGGCTATTTATCTATCTCTTGACTCGAAATAGGAGCAGAGCAGGTTTGAAAAAGGATCTT |  | Homoplasy |
| 164 | Schedonorus arundinaceus Schedonorus arundinaceus[C] Schedonorus pratensis  Festuca altissima  Festuca ovina  Lolium multiflorum  Lolium perenne  Dactylis glomerata  Helictochloa hookeri  Deschampsia antarctica  Poa palustris  Phleum alpinum  Briza sp  Puccinellia nuttalliana  Ammophila breviligulata  Agrostis stolonifera  Hierochloe odorata  Anthoxanthum odoratum  Phalaris arundinacea  Torreyochloa  Avena sativa  Trisetum cernuum  Aegilops cylindrica  Aegilops geniculata  Aegilops speltoides  Aegilops tauschii  Triticum aestivum  Triticum monococcum aegilopoides Triticum monococcum (modified) Triticum urartu (modified)  Hordeum jubatum  Hordeum vulgare  Hordeum vulgare spontaneum Secale cereale  Bromus vulgaris  Brachypodium distachyon  Diarrhena obovata  Melica mutica  Melica subulata  Oryzopsis asperifolia Achnatherum hymenoides Ampelodesmos mauritanica Piptochaetium avenaceum  Phaenosperma globosum Brachyelytrum aristosum  Bambusa bambosa | 113366  IRb  trnA-UGC | trnA-UGC | ATTCTTAAGTTTAAGATCAAAG  ATTCTTAAGTTTAAGATCAAAG  ATTCTTAAGTTTAAGATCAAAG  ATTCTTAAGTTTAAGATCAAAG  ATTCTTAAGTTTAAGATCAAAG  ATTCTTAAGTTTAAGATCAAAG  ATTCTTAAGTTTAAGATCAAAG  ATTCTTAAGTTTAAGATCAAAG  ATTCTTAAGTTTAAGATCAAAG  ATTCTTAAGTTTAAGATCAAAG  ATTCTTAAGTTTAAGATCAAAG  ATTCTTAAGTTTAAGATCAAAG  ATTCTTAAGTTTAAGATCAAAG  ATTCTTAAGTTTAAGATCAAAG  ATTCTTAAGTTTAAGATCAAAG  ATTCTTAAGTTTAAGATCAAAG  ATTCTTAAGTTTAAGATCAAAG  ATTCTTAAGTTTAAGATCAAAG  ATTCTTAAGTTTAAGATCAAAG  ATTCTTAAGTTTAAGATCAAAG  ATTCTTAAGTTTAAGATCAAAG  ATTCTTAAGTTTAAGATCAAAG  ATTCTTAA------GATCAAAG  ATTCTTAA------GATCAAAG  ATTCTTAA------GATCAAAG  ATTCTTAA------GATCAAAG  ATTCTTAA------GATCAAAG  ATTCTTAA------GATCAAAG  ATTCTTAA------GATCAAAG  ATTCTTAA------GATCAAAG  ATTCTTAAGTTTAAGATCAAAG  ATTCTTAAGTTTAAGATCAAAG  ATTCTTAAGTTTAAGATCAAAG  ATTCTTAAGTTTAAGATCAAAG  ATTCTTAAGTTTAAGATCAAAG  ATTCTTAAGTTTAAGATCAAAG  ATTCTTAAGTTTAAGATCAAAG  ATTCTTAAGTTTAAGATCAAAG  ATTCTTAAGTTTAAGATCAAAG  ATTCTTAAGTTTAAGATCAAAG  ATTCTTAAGTTTAAGATCAAAG  ATTCTTAAGTTTAAGATCAAAG  ATTCTTAAGTTTAAGATCAAAG  ATTCTTAAGTTTAAGATCAAAG  ATTCTTAAGTTTAAGATCAAAG  ATTCTTAAGTTTAAGATCAAAG |  | Aegilops+Triticum |
| 165 | Schedonorus arundinaceus Schedonorus arundinaceus[C] Schedonorus pratensis  Festuca altissima  Festuca ovina  Lolium multiflorum  Lolium perenne  Dactylis glomerata  Helictochloa hookeri  Deschampsia antarctica  Poa palustris  Phleum alpinum  Briza sp  Puccinellia nuttalliana  Ammophila breviligulata  Agrostis stolonifera  Hierochloe odorata  Anthoxanthum odoratum  Phalaris arundinacea  Torreyochloa  Avena sativa  Trisetum cernuum  Aegilops cylindrica  Aegilops geniculata  Aegilops speltoides  Aegilops tauschii  Triticum aestivum  Triticum monococcum aegilopoides Triticum monococcum (modified) Triticum urartu (modified)  Hordeum jubatum  Hordeum vulgare  Hordeum vulgare spontaneum Secale cereale  Bromus vulgaris  Brachypodium distachyon  Diarrhena obovata  Melica mutica  Melica subulata  Oryzopsis asperifolia Achnatherum hymenoides Ampelodesmos mauritanica Piptochaetium avenaceum  Phaenosperma globosum Brachyelytrum aristosum  Bambusa bambosa | 113741  IRb  IGS |  | TTCGATCTATGATTTCGCATT  TTCGATCTATGATTTCGCATT  TTCGATCTATGATTTCGCATT  TTCGATCTATGATTTCGCATT  TTCGATCTATGATTTCGAATT  TTCGATCTATGATTTCGCATT  TTCGATCTATGATTTCGCATT  TTCGATCTATGATTTCGCATT  TTCGATCTATGATTTCGCATT  TTCGATCTATGATTTTTCATT  TTCGATCTATGATTTCGCATT  TTCGATCTATGATTTCGCATT  TTCGATCTATGATTTCGCATT  TTCGATTTATGATTTCGCATT  TTCGATCTATGATTTCGCATT  TTCGATCTATGATTTCGCATT  TTCGATCTATGATTTCGCATT  TTCGATCTATGATTTCGGATT  TTTGATCTATGATTTCGCATT  TTCGATCTATGATTTCGCATT  TTCGATC------TTCGCATT  TTCGATC------TTCGCATT  TTCGATCTATGATTTCACATT  TTCGATCTATGATTTCACATT  TTCGATCTATGATTTCACATT  TTCGATCTATGATTTCACATT  TTCGATCTATGATTTCACATT  TTCGATCTATGATTTCACATT  TTCGATCTATGATTTCACATT  TTCGATCTATGATTTCACATT  TTCGATCTATGATTTCACATT  TTCGATCTATGATTTCACATT  TTCGATCTATGATTTCACATT  TTCGATCTATGATTTCACATT  TTCGATCTATGATTTCACATT  TTCGATCTATGATTTCGCATT  TTCGATCTATGATTTCGCATT  TTCGATCTATGATTTCGCATT  TTCGATCTATGATTTCGCATT  TTCGATCTATGATTTCGCATT  TTCGATCTATGATTTCGCATT  TTCGATCTATGATTTCGCATT  TTCGATCTATGATTTCGCATT  TTCGATCTATGATTTCGCATT  TTCGATCTATGATTTCGCATT  TTCGATCTATGATTTCGCATT |  | Aveninae+Koelerinae |
| 166 | Schedonorus arundinaceus Schedonorus arundinaceus[C] Schedonorus pratensis  Festuca altissima  Festuca ovina  Lolium multiflorum  Lolium perenne  Dactylis glomerata  Helictochloa hookeri  Deschampsia antarctica  Poa palustris  Phleum alpinum  Briza sp  Puccinellia nuttalliana  Ammophila breviligulata  Agrostis stolonifera  Hierochloe odorata  Anthoxanthum odoratum  Phalaris arundinacea  Torreyochloa  Avena sativa  Trisetum cernuum  Aegilops cylindrica  Aegilops geniculata  Aegilops speltoides  Aegilops tauschii  Triticum aestivum  Triticum monococcum aegilopoides Triticum monococcum (modified) Triticum urartu (modified)  Hordeum jubatum  Hordeum vulgare  Hordeum vulgare spontaneum Secale cereale  Bromus vulgaris  Brachypodium distachyon  Diarrhena obovata  Melica mutica  Melica subulata  Oryzopsis asperifolia Achnatherum hymenoides Ampelodesmos mauritanica Piptochaetium avenaceum  Phaenosperma globosum Brachyelytrum aristosum  Bambusa bambosa | 117723  IRb | IRb | TTAGG---TATGCT  TTAGG---TATGCT  TTAGG---TATGCT  TTAGG---TATGCT  TTAGG---TATGCT  TTAGG---TATGCT  TTAGG---TATGCT  TTAGG---TATGCT  TTAGG---TATGCT  TTAGG---TATGCT  TTAGG---TATGCT  TTAGG---TATGCT  TTAGG---TATGCT  TTAGG---TATGCT  TTAGG---TATGCT  TTAGG---TATGCT  TTAGG---TATGCT  TTAGG---TATGCT  TTAGG---TATGCT  TTAGG---TATGCT  TTAGG---TATGCT  TTAGG---TATGCT  TTAGG---TATGCT  TTAGG---TATGCT  TTAGG---TATGCT  TTAGG---TATGCT  TTAGG---TATGCT  TTAGG---TATGCT  TTAGG---TATGCT  TTAGG---TATGCT  TTAGG---TATGCT  TTAGG---TATGCT  TTAGG---TATGCT  TTAGG---TATGCT  TTAGG---TATGCT  TTAGG---TATGCT  TTAGG---TATGCT  TTGGGTATTATGCT  TTGGGTATTATGCT  TTAGG---TATGCT  TTAGG---TATGCT  TTAGG---TATGCT  TTAGG---TATGCT  TTGGG---TATGCT  TTGGG---TATGCT  TTGGG---TATGCT | ssmp | Melica |
| 167 | Schedonorus arundinaceus Schedonorus arundinaceus[C] Schedonorus pratensis  Festuca altissima  Festuca ovina  Lolium multiflorum  Lolium perenne  Dactylis glomerata  Helictochloa hookeri  Deschampsia antarctica  Poa palustris  Phleum alpinum  Briza sp  Puccinellia nuttalliana  Ammophila breviligulata  Agrostis stolonifera  Hierochloe odorata  Anthoxanthum odoratum  Phalaris arundinacea  Torreyochloa  Avena sativa  Trisetum cernuum  Aegilops cylindrica  Aegilops geniculata  Aegilops speltoides  Aegilops tauschii  Triticum aestivum  Triticum monococcum aegilopoides Triticum monococcum (modified) Triticum urartu (modified)  Hordeum jubatum  Hordeum vulgare  Hordeum vulgare spontaneum Secale cereale  Bromus vulgaris  Brachypodium distachyon  Diarrhena obovata  Melica mutica  Melica subulata  Oryzopsis asperifolia Achnatherum hymenoides Ampelodesmos mauritanica Piptochaetium avenaceum  Phaenosperma globosum Brachyelytrum aristosum  Bambusa bambosa | 117887  IRb  IGS |  | CAATTCTTT--------------ATTCTTACATTA  CAATTCTTT--------------ATTCTTACATTA  CAATTCTTT--------------ATTCTTACATTA  CAATTCTTT--------------ATTCTTACATTA  CAATTCTTT--------------ATTCTTACATTA  CAATTCTTT--------------ATTCTTACATTA  CAATTCTTT--------------ATTCTTACATTA  CAATTCTTT--------------ATTCTTACATTA  CAATTCTTT--------------ATTCTTACATTA  CAATTCTTT--------------ATTCTTACATTA  CAATTCTTT--------------ATTCTTACATTA  CAATTCTTT--------------ATTCTTACATTA  CAATTCTTT--------------ATTCTTACATTA  CAATTCTTT--------------ATTCTTACATTA  CAATTCTTT--------------ATTCTTACATTA  CAATTCTTT--------------ATTCTTACATTA  CAATTCTTT--------------ATTCTTACATTA  CAATTCTTT--------------ATTCTTACATTA  CAATTCTTT--------------ATTCTTACATTA  CAATTCTTT--------------ATTCTTACATTA  CAATTCTTT--------------ATTCTTACATTA  CAATTCTTT--------------ATTCTTACATTA  CAATTCTTTATTCTTACATTAAAATTCTTACATTA  CAATTCTTTATTCTTACATTAAAATTCTTACATTA  CAATTCTTTATTCTTACATTAAAATTCTTACATTA  CAATTCTTTATTCTTACATTAAAATTCTTACATTA  CAATTCTTTATTCTTACATTAAAATTCTTACATTA  CAATTCTTTATTCTTACATTAAAATTCTTACATTA  CAATTCTTTATTCTTACATTAAAATTCTTACATTA  CAATTCTTTATTCTTACATTAAAATTCTTACATTA  CAATTCTTTATTCTTACATTAAAATTCTTACATTA  CAATTCTTTATTCTTACATTAAAATTCTTACATTA  CAATTCTTTATTCTTACATTAAAATTCTTACATTA  CAATTCTTTATTCTTACATTAAAATTCTTACATTA  CAATTCTTTATTCTTACATTAAAATTCTTACATTA  CAATTCTTT--------------ATTCTTACATTA  CAATTCTTT--------------ATTCTTACATTA  CAATTCTTT--------------ATTCTTACATTA  CAATTCTTT--------------ATTCTTACATTA  CAATTCTTT--------------ATTCTTACATTA  CAATTCTTT--------------ATTCTTACATTA  CAATTCTTT--------------ATTCTTACATTA  CAATTCTTT--------------ATTCTTACATTA  CAATTCTTT--------------ATTCTTACATTA  CAATTCTTT--------------ATTCTTACATTA  CAATTCTTT--------------ATTCTTACATTA | Ssmp | Bromeae+Triticeae |
| 168 | Schedonorus arundinaceus Schedonorus arundinaceus[C] Schedonorus pratensis  Festuca altissima  Festuca ovina  Lolium multiflorum  Lolium perenne  Dactylis glomerata  Helictochloa hookeri  Deschampsia antarctica  Poa palustris  Phleum alpinum  Briza sp  Puccinellia nuttalliana  Ammophila breviligulata  Agrostis stolonifera  Hierochloe odorata  Anthoxanthum odoratum  Phalaris arundinacea  Torreyochloa  Avena sativa  Trisetum cernuum  Aegilops cylindrica  Aegilops geniculata  Aegilops speltoides  Aegilops tauschii  Triticum aestivum  Triticum monococcum aegilopoides Triticum monococcum (modified) Triticum urartu (modified)  Hordeum jubatum  Hordeum vulgare  Hordeum vulgare spontaneum Secale cereale  Bromus vulgaris  Brachypodium distachyon  Diarrhena obovata  Melica mutica  Melica subulata  Oryzopsis asperifolia Achnatherum hymenoides Ampelodesmos mauritanica Piptochaetium avenaceum  Phaenosperma globosum Brachyelytrum aristosum  Bambusa bambosa | 118415  IRb  Ycf1 exon 1 | Ycf1 exon 1 | TCTTTTCTAT--------TTAGTAGTCTAAG  TCTTTTCTAT--------TTAGTAGTCTAAG  TCTTTTCTAT--------TTAGTAGTCTAAG  TCTTTTCTAT--------TTAGTAGTCTAAG  TCTTTTCTAT--------TTAGTAGTCTAAG  TCTTTTCTAT--------TTAGTAGTCTAAG  TCTTTTCTAT--------TTAGTAGTCTAAG  TCTTTTCTAT--------TTAGTAGTCTAAG  TCTTTTCTAT--------TTAGTAGTCTAAG  TCTTTTCTAT--------TTAGTAGTCTAAG  TCTTTTCTAT--------TTAGTAGTCTAAG  TCTTTTCTAT--------TTAGTAGTCTAAG  TCTTTTCTAT--------TTAGTAGTCTAAG  TCTTTTCTAT--------TTAGTAGTCTAAG  TCTTTTCTAT--------TTAGTAGTCTAAG  TCTTTTCTAT--------TTAGTAGTCTAAG  TCTTTTCTAT--------TTAGTAGTCTAAG  TCTTTTCTAT--------TTAGTAGTCTAAG  TCTTTTCTAT--------TTAGTAGTCTAAG  TCTTTTCTAT--------TTAGTAGTCTAAG  TCTTTTCTAT--------TTAGTAGTCTAAG  TCTTTTCTAT--------TTAGTAGTCTAAG  TCTTTTCTAT--------TTAGTAGTCTAAG  TCTTTTCTAT--------TTAGTAGTCTAAG  TCTTTTCTAT--------TTAGTAGTCTAAG  TCTTTTCTAT--------TTAGTAGTCTAAG  TCTTTTCTAT--------TTAGTAGTCTAAG  TCTTTTCTAT--------TTAGTAGTCTAAG  TCTTTTCTAT--------TTAGTAGTCTAAG  TCTTTTCTAT--------TTAGTAGTCTAAG  TCTTTTCTAT--------TTAGTAGTCTAAG  TCTTTTCTAT--------TTAGTAGTCTAAG  TCTTTTCTAT--------TTAGTAGTCTAAG  TCTTTTCTAT--------TTAGTAGTCTAAG  TCTTTTCTAT--------TTAGTAGTCTAAG  TCTTTTCCAT--------TTAGTAGTCTAAG  TCTTTTCTAT--------TTAGTAGTCTAAG  TCTTTTCTATTTATCTATTTAGTAGTCTAAG  TCTTTTCTATTTATCTATTTAGTAGTCTAAG  TCTTTTCTAT--------TTAGTAGTCTAAG  TCTTTTCTAT--------TTAGTAGTCTAAG  TCTTTTCTAT--------TTAGTAGTCTAAG  TCTTTTCTAT--------TTAGTAGTCTAAG  TCTTTTCTAT--------TTAGTAGTCTAAG  TGTTTTCTAT--------TTAGTAGTCTAAG  TCTTTTCTAC--------TTAGTAGTCTAAG |  | Melica |
| 169 | Schedonorus arundinaceus Schedonorus arundinaceus[C] Schedonorus pratensis  Festuca altissima  Festuca ovina  Lolium multiflorum  Lolium perenne  Dactylis glomerata  Helictochloa hookeri  Deschampsia antarctica  Poa palustris  Phleum alpinum  Briza sp  Puccinellia nuttalliana  Ammophila breviligulata  Agrostis stolonifera  Hierochloe odorata  Anthoxanthum odoratum  Phalaris arundinacea  Torreyochloa  Avena sativa  Trisetum cernuum  Aegilops cylindrica  Aegilops geniculata  Aegilops speltoides  Aegilops tauschii  Triticum aestivum  Triticum monococcum aegilopoides Triticum monococcum (modified) Triticum urartu (modified)  Hordeum jubatum  Hordeum vulgare  Hordeum vulgare spontaneum Secale cereale  Bromus vulgaris  Brachypodium distachyon  Diarrhena obovata  Melica mutica  Melica subulata  Oryzopsis asperifolia Achnatherum hymenoides Ampelodesmos mauritanica Piptochaetium avenaceum  Phaenosperma globosum Brachyelytrum aristosum  Bambusa bambosa | 119443  IRb |  | TCTTTTCTAT--------TTAGTAGTCTAAG  TCTTTTCTAT--------TTAGTAGTCTAAG  TCTTTTCTAT--------TTAGTAGTCTAAG  TCTTTTCTAT--------TTAGTAGTCTAAG  TCTTTTCTAT--------TTAGTAGTCTAAG  TCTTTTCTAT--------TTAGTAGTCTAAG  TCTTTTCTAT--------TTAGTAGTCTAAG  TCTTTTCTAT--------TTAGTAGTCTAAG  TCTTTTCTAT--------TTAGTAGTCTAAG  TCTTTTCTAT--------TTAGTAGTCTAAG  TCTTTTCTAT--------TTAGTAGTCTAAG  TCTTTTCTAT--------TTAGTAGTCTAAG  TCTTTTCTAT--------TTAGTAGTCTAAG  TCTTTTCTAT--------TTAGTAGTCTAAG  TCTTTTCTAT--------TTAGTAGTCTAAG  TCTTTTCTAT--------TTAGTAGTCTAAG  TCTTTTCTAT--------TTAGTAGTCTAAG  TCTTTTCTAT--------TTAGTAGTCTAAG  TCTTTTCTAT--------TTAGTAGTCTAAG  TCTTTTCTAT--------TTAGTAGTCTAAG  TCTTTTCTAT--------TTAGTAGTCTAAG  TCTTTTCTAT--------TTAGTAGTCTAAG  TCTTTTCTAT--------TTAGTAGTCTAAG  TCTTTTCTAT--------TTAGTAGTCTAAG  TCTTTTCTAT--------TTAGTAGTCTAAG  TCTTTTCTAT--------TTAGTAGTCTAAG  TCTTTTCTAT--------TTAGTAGTCTAAG  TCTTTTCTAT--------TTAGTAGTCTAAG  TCTTTTCTAT--------TTAGTAGTCTAAG  TCTTTTCTAT--------TTAGTAGTCTAAG  TCTTTTCTAT--------TTAGTAGTCTAAG  TCTTTTCTAT--------TTAGTAGTCTAAG  TCTTTTCTAT--------TTAGTAGTCTAAG  TCTTTTCTAT--------TTAGTAGTCTAAG  TCTTTTCTAT--------TTAGTAGTCTAAG  TCTTTTCCAT--------TTAGTAGTCTAAG  TCTTTTCTAT--------TTAGTAGTCTAAG  TCTTTTCTATTTATCTATTTAGTAGTCTAAG  TCTTTTCTATTTATCTATTTAGTAGTCTAAG  TCTTTTCTAT--------TTAGTAGTCTAAG  TCTTTTCTAT--------TTAGTAGTCTAAG  TCTTTTCTAT--------TTAGTAGTCTAAG  TCTTTTCTAT--------TTAGTAGTCTAAG  TCTTTTCTAT--------TTAGTAGTCTAAG  TGTTTTCTAT--------TTAGTAGTCTAAG  TCTTTTCTAC--------TTAGTAGTCTAAG |  | Melica |
| 170 | Schedonorus arundinaceus Schedonorus arundinaceus[C] Schedonorus pratensis  Festuca altissima  Festuca ovina  Lolium multiflorum  Lolium perenne  Dactylis glomerata  Helictochloa hookeri  Deschampsia antarctica  Poa palustris  Phleum alpinum  Briza sp  Puccinellia nuttalliana  Ammophila breviligulata  Agrostis stolonifera  Hierochloe odorata  Anthoxanthum odoratum  Phalaris arundinacea  Torreyochloa  Avena sativa  Trisetum cernuum  Aegilops cylindrica  Aegilops geniculata  Aegilops speltoides  Aegilops tauschii  Triticum aestivum  Triticum monococcum aegilopoides Triticum monococcum (modified) Triticum urartu (modified)  Hordeum jubatum  Hordeum vulgare  Hordeum vulgare spontaneum Secale cereale  Bromus vulgaris  Brachypodium distachyon  Diarrhena obovata  Melica mutica  Melica subulata  Oryzopsis asperifolia Achnatherum hymenoides Ampelodesmos mauritanica Piptochaetium avenaceum  Phaenosperma globosum Brachyelytrum aristosum  Bambusa bambosa | 120921  ndhF | ndhF | TTTTACTTGATCTAAAAAGAAAAAGTATTTTT  TTTTACTTGATCTAAAAAGAAAAAGTATTTTT  TTTTACTTGATCTAAAAAGAAAAAGTATTTTT  TTTTACTTGATC------GAAAAAGTACTTTT  TTTTACTTGATC------GAAAAAATAATTTT  TTTTACTTGATCTAAAAAGAAAAAGTATTTTT  TTTTACTTGATCTAAAAAGAAAAAGTATTTTT  TTTTACTTGATC------GAAAAAGTACTTTT  TTTTACTTGATC------AAAAAAGTGCTTTT  TTTTACTTGATC------GAAAAAGTGCTTTT  TCTAACTTGATC------GAAAAAGTACTTTT  TTTGACTTGATC------GAAAAAGTACTTTT  TTTTACTTTATT------AAAAAATTCCTTTT  TTTTACTTGATC------GAAAAAGTACTTTT  TTTTACTTTATC------AAAAAATTCCTTTT  TTTTACTTTACC------AAAAAATGCCTTTT  TTTTACTTTATC------AAAAAAGTCCTTTT  TTTTACTTTATC------AAAAAATCCCTTTT  TTTTACTTTATC------GAAAAATTCCTTTT  TTTTACTTTATC------AAAAAAGTCCTTTT  TTTTATTTTATC------AAAAAAGTACGAAA  TTTTACTTTATA------AAAAAAGTCCGAAA  TTTTACTTGATC------TAAAAATTCTTTTT  TTTTACTTGATC------TAAAAATTCTTTTT  TTTTACTTGATC------TAAAAATTCTTTTT  TTTTACTTGATC------TAAAAATTCTTTTT  TTTTACTTGATC------TAAAAATTCTTTTT  TTTTACTTGATC------TAAAAATTCTTTTT  TTTTACTTGATC------TAAAAATTCTTTTT  TTTTACTTGATC------TAAAAATTCTTTTT  TTTTACTTGATC------TAAAAATTCTTTTT  TTTTACTTGATC------CAAAAATTCTTTTT  TTTTACTTGATC------CAAAAATTCTTTTT  TTTTATTTGATC------TAAAAATTCTTTTT  TTTTACTTGATC------AAAAATTTTTTTTT  TTTTACTTGATA------AAAAAAGTTCTTTT  TTTTACTTGATC------AAAAAAGGACTTTT  TTTTACTCGATC------AAAAAAGTCCTTTT  TTTTACTCGATC------AAAAAAGTCCTTTT  TTTTACTCGATC------AAAAAAGTCCTTTT  TTTTACTTGATC------AAAAAAGTCCTTTT  TTTTACTCGATC------AAAAAAGTATTTTT  TTTTACTCGATC------AAAAAAGTCCTTTT  TTTTACTTGATC------AAAAAAGACCCTTT  TTTCACTTGATC------GAAAAAGGACTTTT  TTTTACTTGATC------CAAAAAAGACTTTT |  | Lolium+Schedonorus |
| 171 | Schedonorus arundinaceus Schedonorus arundinaceus[C] Schedonorus pratensis  Festuca altissima  Festuca ovina  Lolium multiflorum  Lolium perenne  Dactylis glomerata  Helictochloa hookeri  Deschampsia antarctica  Poa palustris  Phleum alpinum  Briza sp  Puccinellia nuttalliana  Ammophila breviligulata  Agrostis stolonifera  Hierochloe odorata  Anthoxanthum odoratum  Phalaris arundinacea  Torreyochloa  Avena sativa  Trisetum cernuum  Aegilops cylindrica  Aegilops geniculata  Aegilops speltoides  Aegilops tauschii  Triticum aestivum  Triticum monococcum aegilopoides Triticum monococcum (modified) Triticum urartu (modified)  Hordeum jubatum  Hordeum vulgare  Hordeum vulgare spontaneum Secale cereale  Bromus vulgaris  Brachypodium distachyon  Diarrhena obovata  Melica mutica  Melica subulata  Oryzopsis asperifolia Achnatherum hymenoides Ampelodesmos mauritanica Piptochaetium avenaceum  Phaenosperma globosum Brachyelytrum aristosum  Bambusa bambosa | 125546  IGS |  | TAGATTAGAAAATGGATTCAATTCGA  TAAATTAGAAAATGGATTCAATTCGA  TAGATTAGAAAATGGATTCAATTCGA  TAGATTAGAAAATGGATTCAATTCGA  TAGATTAGAAAATGGATTCAATTCGA  TAGATTATAAAATGGATTCAATTCGA  TAGATTATAAAATGGATTCAATTCGA  TAGATTAGAAACTGGATTCAATTCGA  TAGATTAGAAAATGGATTCAATTCGA  TAGATTAGAAAATGGATTCAATTCGA  TAGATTAGAAAATGGATTCAATTCGA  TAGATTAGAAAATGGATTCAATTCGA  TAGATTAGAAAATGGATTCAATTCGA  TAGATTATAAAATGGATTCAATTC-A  TAGATTAGAAAATGGATTCAATTCGA  TAGATTAGAAAATGGATTCAATTCGA  TAGATTAGAAAATGGATTCAATTCGA  TAGATTAGAAAATGGATTCAATTCGA  TAGATTAGAAAATGGATTCAATTCGA  TAGATTATAAAATGGATTCAATTCGA  TAGATTAGAAAATGGATTCAATTCGA  TAGATTAGAAAATGGATTCAATTCGA  TAGATTAGAAA------TCAATTCGA  TAGATTAGAAA------TCAATTCGA  TAGATTAGAAA------TCAATTCGA  TAGATTAGAAA------TCAATTCGA  TAGATTAGAAA------TCAATTCGA  TAGATTAGAAA------TCAATTCGA  TAGATTAGAAA------TCAATTCGA  TAGATTAGAAA------TCAATTCGA  TAGATTAGAAA------TCAATTCGA  TAGATTAGAAA------TCAATTCGA  TAGATTAGAAA------TCAATTCGA  TAGATTAGAAA------TCAATTCGA  TAGATTAGAAA------TCAATTCGA  TAGATTATAAAATGGATTCAATTCGA  TAGATTATAAAATGGATTCAATTCGA  TAGATTATAAAATGGATTCAATTCGA  TAGATTATAAAATGGATTCAATTCGA  TAGATTAGAAAATAGATTCAATTCAA  TAGATTAGAAAATAGATTCAATTCGA  TAGATTAGAAAATAGATTCAATTCGA  TAGATTAGAAAATAGATTCAATTCGA  TAGATTAGAAAATGAATTTAATTCGA  TAGATTATAAAATGGATTCAATTCGA  TAGATTAGAAAATGGATTCAATTCGA |  | Bromeae+Triticeae |
| 172 | Schedonorus arundinaceus Schedonorus arundinaceus[C] Schedonorus pratensis  Festuca altissima  Festuca ovina  Lolium multiflorum  Lolium perenne  Dactylis glomerata  Helictochloa hookeri  Deschampsia antarctica  Poa palustris  Phleum alpinum  Briza sp  Puccinellia nuttalliana  Ammophila breviligulata  Agrostis stolonifera  Hierochloe odorata  Anthoxanthum odoratum  Phalaris arundinacea  Torreyochloa  Avena sativa  Trisetum cernuum  Aegilops cylindrica  Aegilops geniculata  Aegilops speltoides  Aegilops tauschii  Triticum aestivum  Triticum monococcum aegilopoides Triticum monococcum (modified) Triticum urartu (modified)  Hordeum jubatum  Hordeum vulgare  Hordeum vulgare spontaneum Secale cereale  Bromus vulgaris  Brachypodium distachyon  Diarrhena obovata  Melica mutica  Melica subulata  Oryzopsis asperifolia Achnatherum hymenoides Ampelodesmos mauritanica Piptochaetium avenaceum  Phaenosperma globosum Brachyelytrum aristosum  Bambusa bambosa | 128711  ndhD | ndhD | GGAACCATAACCATATAAGCCCAT  GGAACCATAACCATATAAGCCCAT  GGAACCATAACCATATAAGCCCAT  GGAACC------ATATAAGCCCAT  GGAACC------ATATAAGCCCAT  GGAACCATAACCATATAAGCCCAT  GGAACCATAACCATATAAGCCCAT  GGAACC------ATATAAGCCCAT  GGAACC------ATATAAGCCCAT  GGAACC------ATATAAGCCCAT  GGAACC------ATATAAGCCCAT  GGAACC------ATATAAGCCCAT  GGAACC------ATATAAGCCCAT  AGAACC------ATATAAGCCCAT  GGAACC------ATATAAGCCCAT  GGAACC------ATATAAGCCCAT  GGAACC------ATATAAGCCCAT  GGAACC------ATATAAGCCGAT  GGAACC------ATATAAGCCCAT  GGAACC------ATATAAGCCCAT  GGAACC------ATATAAGCCCAT  GGAACC------ATATAAGCCCAT  GGAACC------ATATAAGCCCAT  GGAACC------ATATAAGCCCAT  GGAACC------ATATAAGCCCAT  GGAACC------ATATAAGCCCAT  GGAACC------ATATAAGCCCAT  GGAACC------ATATAAGCCCAT  GGAACC------ATATAAGCCCAT  GGAACC------ATATAAGCCCAT  GGAACC------ATATAAGCCCAT  GGAACC------GTATAAGCCCAT  GGAACC------GTATAAGCCCAT  GGAACC------ATATAAGCCCAT  GGAACC------GTATAAGCCCAT  GGAACC------GTACAAGCCCAT  GGAACC------ATATAAGCCCAT  GGAACC------ATATAAGCCCAT  GGAACC------ATATAAGCCCAT  GGAACC------ATATAAGCCCAT  GGAACC------ATATAAGCCCAT  GGAACC------ATATAAGCCCAT  GGAACC------ATATAAGCCCAT  GGAACC------ATATAAGCCCAT  GGAACC------ATATAAGCCCAT  GGAACC------ATATAAGCCCAT |  | Lolium+Schedonorus |
| 173 | Schedonorus arundinaceus Schedonorus arundinaceus[C] Schedonorus pratensis  Festuca altissima  Festuca ovina  Lolium multiflorum  Lolium perenne  Dactylis glomerata  Helictochloa hookeri  Deschampsia antarctica  Poa palustris  Phleum alpinum  Briza sp  Puccinellia nuttalliana  Ammophila breviligulata  Agrostis stolonifera  Hierochloe odorata  Anthoxanthum odoratum  Phalaris arundinacea  Torreyochloa  Avena sativa  Trisetum cernuum  Aegilops cylindrica  Aegilops geniculata  Aegilops speltoides  Aegilops tauschii  Triticum aestivum  Triticum monococcum aegilopoides Triticum monococcum (modified) Triticum urartu (modified)  Hordeum jubatum  Hordeum vulgare  Hordeum vulgare spontaneum Secale cereale  Bromus vulgaris  Brachypodium distachyon  Diarrhena obovata  Melica mutica  Melica subulata  Oryzopsis asperifolia Achnatherum hymenoides Ampelodesmos mauritanica Piptochaetium avenaceum  Phaenosperma globosum Brachyelytrum aristosum  Bambusa bambosa | 129397  IGS |  | GTTTTT------TGTAACGT  GTTTTT------TGTAACGT  GTTTTT------TGTAACGT  GTTTTT------TGTAACGT  GTTTTT------TGGAACGT  GTTTTT------TGTAACGT  GTTTTT------TGTAACGT  GTTTTT------TGTAACGT  GTTTTT------TGTAACGT  GTTTTT------TGTAACGT  GTTTTT------TGTAACGT  GTTTTT------TGTAACGT  GTTTTT------TGTAACGT  GTTTTT------TGTAACGT  GTTTTT------TGTAACGT  GTTTTT------TGTAACGT  GTTTTT------TGTAACGT  GTTTTT------TGTAACGT  GTTTTT------TGTAACGT  GTTTTT------TGTAACGT  GTTTTT------TGTAACGT  GTTTTT------TGTAACGT  GTTTTT------TGTAACGT  GTTTTT------TGTAACGT  GTTTTT------TGTAACGT  GTTTTT------TGTAACGT  GTTTTT------TGTAACGT  GTTTTT------TGTAACGT  GTTTTT------TGTAACGT  GTTTTT------TGTAACGT  GTTTTT------TGTAACGT  GTTTTTTGTTTTTGTAACGT  GTTTTTTGTTTTTGTAACGT  GTTTTT------TGTAACGT  GTTTTT------TGTAACGT  GTTTTT------TGTAACGT  GTTTTT------TGTAACGT  GTTTTT------TGTAACCT  GTTTTT------TGTAACCT  GTTTTT------TGTAACGT  GTTTTT------TGTAACGT  GTTTTT------TGTAACGT  GTTTTT------TGTAACGT  GTTTTT------TGTAACGT  ATTATT------TGTAACGT  GTTTTT------TGTAACGT |  | Hordeum vulgare |
| 174 | Schedonorus arundinaceus Schedonorus arundinaceus[C] Schedonorus pratensis  Festuca altissima  Festuca ovina  Lolium multiflorum  Lolium perenne  Dactylis glomerata  Helictochloa hookeri  Deschampsia antarctica  Poa palustris  Phleum alpinum  Briza sp  Puccinellia nuttalliana  Ammophila breviligulata  Agrostis stolonifera  Hierochloe odorata  Anthoxanthum odoratum  Phalaris arundinacea  Torreyochloa  Avena sativa  Trisetum cernuum  Aegilops cylindrica  Aegilops geniculata  Aegilops speltoides  Aegilops tauschii  Triticum aestivum  Triticum monococcum aegilopoides Triticum monococcum (modified) Triticum urartu (modified)  Hordeum jubatum  Hordeum vulgare  Hordeum vulgare spontaneum Secale cereale  Bromus vulgaris  Brachypodium distachyon  Diarrhena obovata  Melica mutica  Melica subulata  Oryzopsis asperifolia Achnatherum hymenoides Ampelodesmos mauritanica Piptochaetium avenaceum  Phaenosperma globosum Brachyelytrum aristosum  Bambusa bambosa | 129409  IGS |  | TGTAACGT----ATCAATAAGA  TGTAACGT----ATCAATAAGA  TGTAACGT----ATCAATAAGA  TGTAACGT----ATCAATAAGA  TGGAACGT----ATCAATAAGA  TGTAACGT----ATCAATAAGA  TGTAACGT----ATCAATAAGA  TGTAACGT----ATCAATAAGA  TGTAACGT----ATCAATAAGA  TGTAACGT----ATCAATAAGA  TGTAACGT----ATCAATAAGA  TGTAACGT----ATCAATAAGA  TGTAACGT----ATCAATAAGA  TGTAACGT----ATCAATAAGA  TGTAACGTATCAATCAATAAGA  TGTAACGTATCAATCAATAAGA  TGTAACGT----ATCAATAAGA  TGTAACGT----ATCAATAAGA  TGTAACGT----ATCAATAAGA  TGTAACGT----ATCAATAAGA  TGTAACGT----ATCAATAAGA  TGTAACGT----ATCAATAAGA  TGTAACGT----ATCAATAAGA  TGTAACGT----ATCAATAAGA  TGTAACGT----ATCAATAAGA  TGTAACGT----ATCAATAAGA  TGTAACGT----ATCAATAAGA  TGTAACGT----ATCAATAAGA  TGTAACGT----ATCAATAAGA  TGTAACGT----ATCAATAAGA  TGTAACGT----ATCAATAAGA  TGTAACGT----ATCAATAAGA  TGTAACGT----ATCAATAAGA  TGTAACGT----ATCAATAAGA  TGTAACGT----ATCAATAAGA  TGTAACGT----ATCAATAAGA  TGTAACGT----ATCAATAAGA  TGTAACCT----ATCAATAAGA  TGTAACCT----ATCAATAAGA  TGTAACGT----ATCAATAAGA  TGTAACGT----ATCAATAAGA  TGTAACGT----ATCAATAAGA  TGTAACGT----ATCAATAAGA  TGTAACGT----ATCAATAAGA  TGTAACGT----ATCAATAAGC  TGTAACGT----ATCAATAAGA | ssmp | Agrostidinae |
| 175 | Schedonorus arundinaceus Schedonorus arundinaceus[C] Schedonorus pratensis  Festuca altissima  Festuca ovina  Lolium multiflorum  Lolium perenne  Dactylis glomerata  Helictochloa hookeri  Deschampsia antarctica  Poa palustris  Phleum alpinum  Briza sp  Puccinellia nuttalliana  Ammophila breviligulata  Agrostis stolonifera  Hierochloe odorata  Anthoxanthum odoratum  Phalaris arundinacea  Torreyochloa  Avena sativa  Trisetum cernuum  Aegilops cylindrica  Aegilops geniculata  Aegilops speltoides  Aegilops tauschii  Triticum aestivum  Triticum monococcum aegilopoides Triticum monococcum (modified) Triticum urartu (modified)  Hordeum jubatum  Hordeum vulgare  Hordeum vulgare spontaneum Secale cereale  Bromus vulgaris  Brachypodium distachyon  Diarrhena obovata  Melica mutica  Melica subulata  Oryzopsis asperifolia Achnatherum hymenoides Ampelodesmos mauritanica Piptochaetium avenaceum  Phaenosperma globosum Brachyelytrum aristosum  Bambusa bambosa | 129939  IGS |  | TAGCCAA-----TAAATTGGC  TAGCCAA-----TAAATTGGC  TAGCCAA-----TAAATTGGC  TAGCCAA-----TAAATTGGC  TAGCCAA-----TAAATTGGC  TAGCCAA-----TAAATTGGC  TAGCCAA-----TAAATTGGC  TAGCCAA-----TAAATTGGC  TAGCCAA-----TAAATTGGC  TAGCCAA-----TAAATTGGC  TAGCCAA-----TAAATTGGC  TAGCCAA-----TAAATTGGC  TAGCCAA-----TAAATTGGC  TAGCCAA-----TAAATTGGC  TAGCCAA-----TAAATTGGC  TAGCCAA-----TAAATTGGC  TAGCCAA-----TAAATTGGC  TAGCCAA-----TAAATTGGC  TAGCCAA-----TAAATTGGC  TAGCCAA-----TAAATTGGC  TAGCCAA-----TAAATTGGC  TAGCCAA-----TAAATTGGC  TAGCCAA-----TAAATTGGC  TAGCCAA-----TAAATTGGC  TAGCCAA-----TAAATTGGC  TAGCCAA-----TAAATTGGC  TAGCCAA-----TAAATTGGC  TAGCCAA-----TAAATTGGC  TAGCCAA-----TAAATTGGC  TAGCCAA-----TAAATTGGC  TAGCCAATTTATTTTATTGGC  TAGCCAATTTATTTTATTGGC  TAGCCAATTTATTTTATTGGC  TAGCCAA-----TAAATTGGC  TAGCCAA-----TAAATTGGC  TCGCCAA-----TAAATTGGC  TAGCCAA-----TAAATTGGC  TAGCTAA-----TAAATAGCC  TAGCTAA-----TAAATAGGC  TAGCCAA-----TAAATGGGC  TAGCCAA-----TAAATTGGC  TAGCCAA-----TAAATTGGC  TAGCCAA-----TAAATTGGC  TAGCCAA-----AAAATAGGC  TAGCCAA-----TAAATTGGC  TAGCCAA-----TAAATTGGC |  | Hordeum vulgare |
| 176 | Schedonorus arundinaceus Schedonorus arundinaceus[C] Schedonorus pratensis  Festuca altissima  Festuca ovina  Lolium multiflorum  Lolium perenne  Dactylis glomerata  Helictochloa hookeri  Deschampsia antarctica  Poa palustris  Phleum alpinum  Briza sp  Puccinellia nuttalliana  Ammophila breviligulata  Agrostis stolonifera  Hierochloe odorata  Anthoxanthum odoratum  Phalaris arundinacea  Torreyochloa  Avena sativa  Trisetum cernuum  Aegilops cylindrica  Aegilops geniculata  Aegilops speltoides  Aegilops tauschii  Triticum aestivum  Triticum monococcum aegilopoides Triticum monococcum (modified) Triticum urartu (modified)  Hordeum jubatum  Hordeum vulgare  Hordeum vulgare spontaneum Secale cereale  Bromus vulgaris  Brachypodium distachyon  Diarrhena obovata  Melica mutica  Melica subulata  Oryzopsis asperifolia Achnatherum hymenoides Ampelodesmos mauritanica Piptochaetium avenaceum  Phaenosperma globosum Brachyelytrum aristosum  Bambusa bambosa | 130012  IGS |  | CAATGAAT------TAAA  CAATGAAT------TAAA  CAATGAAT------TACA  CAATGAAT------TACA  CAATGAAT------TACA  CAATGAAT------TACA  CAATGAAT------TACA  CAATGAAT------TACA  CAATGAAT------TACA  CAATGAAT------TACA  CAATGAAT------TACA  CAATGAAT------TACA  CAATGAAT------TAAA  CAATGAAT------TACA  CAATGAAT------TACA  CAATGAAT------TACA  CAATGAAT------TACA  CAATGAAT------TACA  CAATCAAT------TACA  CAATGAAT------TACA  CAATGAAT------TACA  CAATGAAT------TACA  CAATGAATTACAAATACA  CAATGAATTACAAATAAA  CAATGAATTCCAAATACA  CAATGAATTACAAATACA  CAATGAATTACAAATACA  CAATGAATTAAAAATACA  CAATGAATTAAAAATACA  CAATGAATTACAAATACA  CAATGAATTACAAATACA  CAATGAATTACAAATACA  CAATGAATTACAAATACA  CAATGAATTACAAATACA  CAATGAAT------TACA  CAATGAAT------TAGA  CAATGAAT------TACA  CAATGAAT------TACA  CAATGAAT------TACA  CAATGAAT------TACA  CAATGAAT------TACA  CAATGAAT------TACA  CAATGAAT------TACA  CAATGCAT------TACA  CAATGAAT------TGCA  CAATGAAT------TGCA |  | Triticeae |
| 177 | Schedonorus arundinaceus Schedonorus arundinaceus[C] Schedonorus pratensis  Festuca altissima  Festuca ovina  Lolium multiflorum  Lolium perenne  Dactylis glomerata  Helictochloa hookeri  Deschampsia antarctica  Poa palustris  Phleum alpinum  Briza sp  Puccinellia nuttalliana  Ammophila breviligulata  Agrostis stolonifera  Hierochloe odorata  Anthoxanthum odoratum  Phalaris arundinacea  Torreyochloa  Avena sativa  Trisetum cernuum  Aegilops cylindrica  Aegilops geniculata  Aegilops speltoides  Aegilops tauschii  Triticum aestivum  Triticum monococcum aegilopoides Triticum monococcum (modified) Triticum urartu (modified)  Hordeum jubatum  Hordeum vulgare  Hordeum vulgare spontaneum Secale cereale  Bromus vulgaris  Brachypodium distachyon  Diarrhena obovata  Melica mutica  Melica subulata  Oryzopsis asperifolia Achnatherum hymenoides Ampelodesmos mauritanica Piptochaetium avenaceum  Phaenosperma globosum Brachyelytrum aristosum  Bambusa bambosa | 130026  IGS |  | TAAAAA-------AATTCCTTT  TAAAAA-------AATTCCTTT  TACAAA-------AATTCCTTT  TACAAA-------AATTCCTTT  TACAAA-------AATTCCTTT  TACAAA-------AATTCCTTT  TACAAA-------AATTCCTTT  TACAAA-------AATTCTTTT  TACAAA-------AATCCCTTT  TACAAA-------AATTCCTTT  TACAAA-------AATTCCTTT  TACAAA-------AATTCCTTT  TAAAAA-------AATTCATTT  TACAAA-------AATTCCTTT  TACAAA-------AATTCATTT  TACAAA-------AATTCATTT  TACAAA-------AATGCATTT  TACAAA-------AATACATTT  TACAAA-------AATTCATTT  TACAAA-------AATTCATTT  TACAAA-------AATTCATTT  TACAAA-------AATTCATTT  TACAAA-------AATTCATTT  TAAAAA-------AATTCATTT  TACAAA-------AATTCATTT  TACAAA-------AATTCATTT  TACAAA-------AATTCATTT  TACAAA-------AATTCATTT  TACAAA-------AATTCATTT  TACAAA-------AATTCATTT  TACAAA-------AATTCATTT  TACAAA-------AATTCATTT  TACAAA-------AATTCATTT  TACAAA-------AATTCATTT  TACAAA-------AATTCATTT  TAGAAA-------AATTCATTT  TACAAA-------AATTCATTT  TACAAAAATTTACAATTTATAT  TACAAAAATTTACAATTTATAT  TACAAA-------AATTCATTT  TACAAA-------AATTCATTT  TACAAA-------AATTCATTT  TACAAA-------AATTCAT--  TACAAA-------AATTCAA-T  TGCAAA-------AATGCAA-T  TGCAAA-------AATTCAA-T |  | Melica |
